# RNA Demethylase ALKBH5 Prevents Lung Cancer Progression by Regulating EMT and Stemness *via* Regulating p53

**DOI:** 10.3389/fonc.2022.858694

**Published:** 2022-04-22

**Authors:** Xiangli Liu, Ziyi Wang, Qiwei Yang, Xiaohai Hu, Qiang Fu, Xinyu Zhang, Wenya Li

**Affiliations:** Department of Thoracic Surgery, The First Affiliated Hospital of China Medical University, Shenyang, China

**Keywords:** nonsmall-cell lung cancer (NSCLC), N6-methyladenosine (m^6^A) methylation, cancer stem-like cells (CSCs), ALKBH5, epithelial and mesenchymal transition (EMT)

## Abstract

**Background:**

Although N6-methyladenosine (m^6^A) RNA methylation is the most abundant reversible methylation of mRNA, which plays a critical role in regulating cancer processing, few studies have examined the role of m^6^A in nonsmall-cell lung cancer-derived cancer stem-like cells (CSCs).

**Methods:**

CSCs were enriched by culturing NSCLC cells in a serum-free medium, and stem factors, including CD24, CD44, ALDH1, Nanog, Oct4, and Sox2 were detected by Western blot. ALKBH5 expression was measured by employing a tissue array. Global m^6^A methylation was measured after ALKBH5 knockdown. Malignances of CSCs were detected by performing CCK-8 assay, invasion assay, cell cycle analysis, and tumor formation *in vitro* and *in vivo*.

**Results:**

m^6^A demethylase ALKBH5 is highly expressed in CSCs derived from NSCLC. Knockdown of ALKBH5 increased global m^6^A level, and also increased E-cadherin, decreased stem hallmarkers, Nanog and Oct4, and inhibited stemness of CSCs. In lung carcinoma, ALKBH5 is found to be positively correlated with p53 by using Gene Expression Profiling Interactive Analysis (GEPIA) online tool. P53 transcriptionally regulates ALKBH5 and subsequently regulates the global m^6^A methylation level. Knockdown of p53 or inhibition of p53’s transcriptional activity by addition of its specific inhibitor PFT-α decreased expression of ALKBH5 and CSCs’ malignancies, including proliferation, invasion, and tumor formation ability, indicating that p53 may partially regulate CSC’s malignancies *via* ALKBH5. Furthermore, we also found p53 transcriptionally regulates PRRX1, which is consistent with our previous report.

**Conclusion:**

Collectively, our findings indicate the pivotal role of ALKBH5 in CSCs derived from NSCLC and highlight the regulatory function of the p53/ALKBH5 axis in modulating CSC progression, which could be a promising therapeutic target for NSCLC.

## Introduction

This small group of cells, known as cancer stem cells (CSCs), is characteristic of stem cells. The ability of CSCs to self-renew, multidifferentiate, transfer (1), and evade drug-induced cell death is mainly due to their dormant stem-like properties (2), which are characterized by clinical recurrence; the diffuse tumor cells remained inactive for long periods of time. This may occur in the early stages of the disease or after treatment interventions. Activation of these dormant cells contributes to tumor growth and recurrence. The self-protection of CSCs is achieved through asymmetric cell division cycles in which CSC populations are retained, resulting in heterogeneous tumor populations of CSCs and nonstem-like cancer cells (3). These nonstem-like cancer cells experienced rapid symmetric cell proliferation, making them susceptible to traditional cancer treatments and preserving the cancer stem cell population, vindicating treatment failure and cancer recurrence. These important clinical findings further stimulate strong interest in the further study of CSCs and their involvement in the treatment of drug-resistant lung cancer alternatives. CSCs exhibit high drug resistance and toughness due to prolonged telomere duration, initiation of apoptotic pathways, enhanced membrane transport protein activity, and enhanced mobility and metastasis.

The complete treatment of cancer depends on revealing its origin. Lung CSCs identify and demonstrate resistance to various lung cancer treatment regimens. These include conventional therapies, biomolecules, and targeted therapies. The elimination of lung cancer stem cells during therapeutic interventions is critical because it prevents tumor expansion, recurrence, and metastasis. Although little is known about the biology of pulmonary embolism, various markers of pulmonary embolism have been distinguished and considered. These markers were incorporated but not limited to ALDH1, CD133, side groups (Hoechst negative), CD44, CD87, and CD117. These markers are associated with chemoresistance in the treatment of various first-line diseases. Therefore, it is widely believed that CSC is closely related to pathological features, resulting in a poor clinical prognosis.

N6-methyladenosine (m^6^A) is the most common internal chemical modification in eukaryotes. In mammals, m^6^A installed by m^6^A methyltransferase METTL3 and METTL14 is erased by fat mass and obesity-related proteins (FTO) or α-ketoglutaric acid-dependent dioxygenase homologs 5 (ALKBH5) (4–7). The effects of MRNA m^6^A modification on cellular processes include changes in RNA stability (6, 8), translation efficiency (9, 10), secondary structure (11), subcellular localization (12), alternative polyadenylation, and splicing (13). m^6^A methyltransferase is critical for the speed and differentiation of mouse embryonic stem cells and circadian clock. FTO is known to regulate fat production and energy homeostasis (14). ALKBH5 is overexpressed in testis but downregulated in the heart and brain, affecting nuclear RNA output and metabolism, gene expression, and mouse fertility (7). ALKBH5 knockout mice are viable, but significant changes in the expression of key genes required for spermatogenesis and maturation indicate a spermatogenesis disorder, although the overall increase in m^6^A levels in the testes is modest (7). These studies suggest that key gene expression changes, which are sensitive to the function of m^6^A regulators, can cause significant phenotypic changes. So far, however, the biological significance and key target genes of these m^6^A modulators in human cancer remain elusive.

Regulation of ALKBH5 is manifested by multiple tumors (15, 16). A previous report shows when ALKBH5 is reduced in PC, the combination of P53 with ALKBH5 promoter was confirmed by genome analysis, microarray verification, and luciferase analysis, combined with bioinformatics prediction, indicating that P53 was transcriptionally activated on ALKBH5 gene. As a well-investigated transcriptional activator, p53 mutated about half of the malignant diseases and may explain the downregulation of ALKBH5 expression.

The molecular mechanism of RNA M6 methylation in regulating malignancies of different kinds of cancer has only recently begun to be elucidated. Dominissini and colleagues tried to explain the roles of m^6^A methylation *via* regulating p53 signaling pathways, DNA mismatch repair, and RNA degradation (17). It is also reported that more than 7,000 human genes were sequenced using m^6^A and found that silencing m^6^A methyltransferase regulates the p53 signaling pathway-mediated apoptosis. Gabbert and Martin also reported that in gastric cancer, m^6^A modification may exert critical regulatory roles *via* regulating p53 signaling pathway (18, 19).

In recent years, the carcinogenic or antitumor function of paired related homeobox 1 (PRRX1) has been reported in many kinds of tumors. In glioma and pancreatic cancer, PRRX1 is overexpressed in tumor-initiating cells and plays a regulatory role in tumor invasion and metastasis. In breast, lung, or hepatocellular carcinoma, PRRX1 inhibits the self-renewal and stem cell support of tumor-initiating cells (20, 21). In the field of sarcoma research, mice that eventually develop osteosarcoma p53 and Rb lack PRRX1-positive cells or osteoblasts (22–25), suggesting that PRRX1-positive cells play a key role in the development of osteosarcoma. However, the function of PRRX1 in human osteosarcoma has not been determined. In our previous report, it is presented that PRRX1 regulates the stemness phenotype and epithelial–mesenchymal transition (EMT) in CSCs enriched from nonsmall cell lung cancer (NSCLC) (26). However, the exact role of PRRX1 in regulating NSCLC is still largely unknown.

Here, we examined the correlation between m^6^A methylation regulated by ALKBH5 and malignancies in human NSCLC-derived CSCs. We further investigated the modification of m^6^A methylation on p53 using human NSCLC cell lines A549 and PC-9.

## Material and Methods

### Cell Culture and Enrichment of CSCs

Nonsmall cell lung cancer (NSCLC) cell lines A549 and PC-9 were all purchased from the American Type Culture Collection (ATCC). Cells were routinely maintained in Dulbecco’s modified Eagle’s medium (DMEM) supplemented with 10% fetal bovine serum (FBS, Gibco, Ca, USA) and antibiotics (50 U/ml penicillin and 50 μg/ml streptomycin, Gibco) at 37°C in a humidified atmosphere containing 5% CO_2_. To enrich CSCs from either cells, viable cells were seeded at a 6-well plate and cultured in DMEM/Ham Nutrient Mixture F-12 (F-12) (1:1) supplemented with epidermal growth factor (EGF, 20 ng/ml, Sigma-Aldrich, USA), human fibroblast growth factor basic (hFGFb, 10 ng/ml, Sigma-Aldrich, USA), and 2% B27 (Life Technologies, USA) for 14 days. Every 3 days, the medium was half-refreshed with the addition of relative supplements. For storage, cells were collected and suspended in DMEM/F-12 without the addition of serum and frozen in liquid nitrogen until use.

### Cell Viability Analysis

Cells were suspended and adjusted to a concentration of 1 × 106 cells/ml. For each well of a 96-well plate, 2,500 cells were seeded and allowed to attach to the bottom of the plate overnight. To analyze cell viability, the Cell Counting Kit-8 (CCK-8, Sigma-Aldrich, St. Louis, MO, USA)-prepared solution was employed and 10 µl of CCK-8 solution was added into each well for a 4-h coincubation at 37°C away from light. OD450 was detected by a microplate reader (Synergy 2 Multi-Mode Microplate Reader, BioTek, Winooski, VT, USA) to determine the cell viability.

### Reverse-Transcription Quantitative PCR

One microgram of total RNA was reverse transcripted following the instruction of the manufacturer, and complementary DNA (cDNA) was used as a template by using Reverse Transcriptase Kit (RIBOBIO, Guangzhou, China). The primers were listed as follow: CD24 sense primer 5′-CTCCTACCCACGCAGATTTATTC-3′ and antisense primer 5′-AGAGTGAGACCACGAAGAGAC-3′; CD44 sense primer 5′-CTGCCGCTTTGCAGGTGTA-3′ and antisense primer 5′-CATTGTGGGCAAGGTGCTATT-3′; ALDH1 sense primer 5′-GCACGCCAGACTTACCTGTC-3′ and antisense primer 5′-CCTCCTCAGTTGCAGGATTAAAG-3′; Nanog sense primer 5′-TTTGTGGGCCTGAAGAAAACT-3′ and antisense primer 5′-AGGGCTGTCCTGAATAAGCAG-3′; Oct4 sense primer 5′-CTGGGTTGATCCTCGGACCT-3′ and antisense primer 5′-CCATCGGAGTTGCTCTCCA-3′; Sox2 sense primer 5′–3′ and antisense primer 5′–3′; METTL3 sense primer 5′-TTGTCTCCAACCTTCCGTAGT-3′ and antisense primer 5′-CCAGATCAGAGAGGTGGTGTAG-3′; METTL14 sense primer 5′-AGTGCCGACAGCATTGGTG-3′ and antisense primer 5′-GGAGCAGAGGTATCATAGGAAGC-3′; YTHDF1 sense primer 5′-ACCTGTCCAGCTATTACCCG-3′ and antisense primer 5′-TGGTGAGGTATGGAATCGGAG-3′; WTAP sense primer 5′-CTTCCCAAGAAGGTTCGATTGA-3′ and antisense primer 5′-TCAGACTCTCTTAGGCCAGTTAC-3′; FTO sense primer 5′-ACTTGGCTCCCTTATCTGACC-3′ and antisense primer 5′–3′; ALKBH5 sense primer 5′-TGTGCAGTGTGAGAAAGGCTT-3′ and antisense primer 5′-CCACCAGCTTTTGGATCACCA-3′; β-actin sense primer 5′-CATGTACGTTGCTATCCAGGC-3′ and antisense primer 5′-CTCCTTAATGTCACGCACGAT-3′. PCR was then carried out using PowerUp SYBR™ Green Master Mix (Thermo Scientific, Waltham, MA, USA) following the manufacturer’s instructions. mRNA levels were normalized against β-actin mRNA and expressed relative to the control conditions.

### Western Blot

To detect specific protein levels, Western blot was carried out by using primary antibodies, which were all rabbit monoclonal and bought from Abcam (Cambridge, Britain). The primary antibodies used was listed as followed: anti-CD24 (Cat. No.: ab202073); anti-CD44 (Cat. No.: 189524); anti-ALDH1 (Cat. No.: ab227984); anti-Nanog (Cat. No.: ab109250); anti-Oct4 (Cat. No.: ab200834); anti-Sox2 (Cat. No.: ab92494); anti-β-actin (Cat. No.: ab115777); anti-METTL3 (Cat. No.: ab195352); anti-METTL14 (Cat. No.: ab252562); anti-YTHDF1 (Cat. No.: ab252346); anti-WTAP (Cat. No.: ab195380); anti-FTO (Cat. No.: ab126605); anti-ALKBH5 (Cat. No.: ab195377); anti-E-cadherin (Cat. No.: ab231303); anti-p53 (Cat. No.: ab32389); anti-PRRX1 (Cat. No.: ab211292). All antibodies were diluted at 1:1,000. Goat anti-rabbit IgG H&L antibody (HRP ladled, 1:10,000, #ab7090) was used as a secondary antibody. Blot bands were quantified *via* densitometry with Image J software (National Institutes of Health Baltimore, MD, USA). β-Actin was used as an internal reference.

### Detection of m^6^A Methylation Level

To quantitatively measure the m^6^A methylation level, total RNA was extracted from tissue samples using TRIzol reagent (Thermo Scientific, USA) following the manufacturer’s instruction, and then m^6^A RNA methylation quantification kit (Thermo Scientific, USA) was employed for this purpose by following manufacturer’s instruction. The percentage of m^6^A-methylated mRNA in the total mRNA was calculated to access the m^6^A methylation level. Briefly, 200 ng mRNA of each sample was loaded in the assay well. A capture antibody specific for m^6^A (Synaptic Systems, catalog No. 202003, at dilution of 1:2,000) was then added to the wells. Two-hour incubation later, wells were washed three times using washing buffer, and a detection antibody (Abcam, Catalog. No. ab6747, at dilution of 1:5,000) was added to each well. The developer solution was added to each well following the wash steps to remove any liquid while protecting samples from light. The color was developed and captured using a 450-nm wavelength optical meter.

### GEPIA Analysis

The online database gene expression profiling interactive analysis (GEPIA) (http://gepia.cancer-pku.cn/index.html) was used to investigate differential expression analysis, profiling according to pathological stages, patient survival analysis, and correlation analysis

### PI Staining and Flow Cytometric Analysis

Cells were collected and adjusted to a concentration of 1 × 106 cells/ml and then fixed using 70% anhydrous ethanol overnight, stained with 50 μg/ml propidium iodide (PI, Sigma) for 30 min at 4°C, and then assessed with a Beckman Navios. The cell phase is represented by G0–G1, S, and G2–M.

### Invasion

To assess invasive capacity, a 24-well Transwell insert system (Corning, NY) prelaid with Matrigel was used. For each well, 1 × 104 cells were plated in the top chamber containing a Matrigel-coated membrane (60 µg of Matrigel for each well). In the lower chamber, a medium containing 10% FBS was filled to be used as a chemoattractant. After 48-h incubation, cells on the lower surface of the membrane were fixed with methanol and stained with 0.1% crystal violet. The number of cells invading the membrane was counted under a microscope (Olympus, Tokyo, Japan).

### Colony Formation

Cells at 2.5 × 103 were seeded in 0.3% low melting soft agar laid on 0.6% low melting soft agar support for 3-week incubation. Colonies were fixed with methanol and stained with 0.1% crystal violet in 20% methanol for 30 min. Each assay was performed in triplicate.

### Tissue Microarray of ISH

#### Clinical Colorectal Cancer Tissue Microarray

Lung adenocarcinoma tissue microarrays (HLugS120CS01) and lung squamous cell carcinoma (HLuGA150CS03) were purchased from Outdo Biotech (Shanghai Outdo Biotech Co. Ltd., China). Completed clinicopathology data were collected for further analysis. The EnVision+detetion system (Dako) was employed to perform immunohistochemistry following the manufacturer’s instructions. A semiquantitative scoring criterion for IHC of ALKBH5 staining was used (week or moderated was considered negative; strong was considered positive). Two independent pathologists, blinded to the clinicopathological information, performed the identification.

### Human Protein Antibody Membrane Array Analysis

Fifteen different proteins were embedded on a human Protein Antibody Membrane Array (Cat. No.: ab211066, Abcam) according to the manufacturer’s instructions. The experiments were performed following the manufacturer’s instructions. Briefly, 650 μg total protein of each sample was used for 2-h incubation with membrane. Detection of proteins was performed by incubation with horseradish peroxidase(HRP)-conjugated streptavidin for 2 h. Signal intensities were detected by chemiluminescence, and the membranes (*n* = 2) were briefly exposed to x-ray films (GE Healthcare, Munich, Germany) for 30 s.

### RNA Knockdown

To knockdown an inverted and self-complementary hairpin DNA oligonucleotide, encoding a short-hairpin RNA-targeting ALKBH5 mRNA was chemically synthesized (RIBOBIO, Guangzhou, China), aligned, and cloned into the lentiviral vector pLL3.7 (RIBOBIO, Guangzhou, China) that coexpresses the fluorescent protein GFP. As a control, we used shRNA-targeting Luciferase mRNA. Oligos used to construct the shRNA-targeting ALKBH5 a were as follows: 5′-CCTCATAGTCGCTGCGCTCG-3′; 5′-ATAGTTGTCCCGGGACGTCA-3′ (reverse). Oligos used as control shRNA were as follows: 5′-TTCTCCGAACGTGTCACGA-3′ (forward); 5′-ACGTGACACGTTCGGAGAATT-3′ (reverse).

### Animal Experiment

All the animal experiments were conducted according to the ethics committee. All procedures in this section were approved by the Medical Ethics Committee of the Shanghai Outdo Biotech Company and performed according to the ethical guidelines (ethics No.: YB M-05-02). Four-week-old female BALB/c nude mice were purchased from Dashuo Experimental Animal Company (Chengdu, China) and raised in the SPF animal facilities.

Cells at 1 × 106 were subcutaneously injected into the mice similarly to nude mice. Twenty-eight days later, grafted tumors were collected and morphologically analyzed. The proliferating capacity was measured by Ki67 staining.

### Statistical Analysis

Each experiment was performed at least three times. The software GraphPad Prism software was used for data analysis. Statistical analyses were performed using ANOVA (equal variance) or Welch’s ANOVA (unequal variance). A statistically significant difference among groups was defined as P < 0.05.

## Results

### ALKBH5 Is Upregulated in CSCs Compared With Those in Parental Cells and Leads to a Decrease in Global m^6^A Methylation

**Figure 1:**
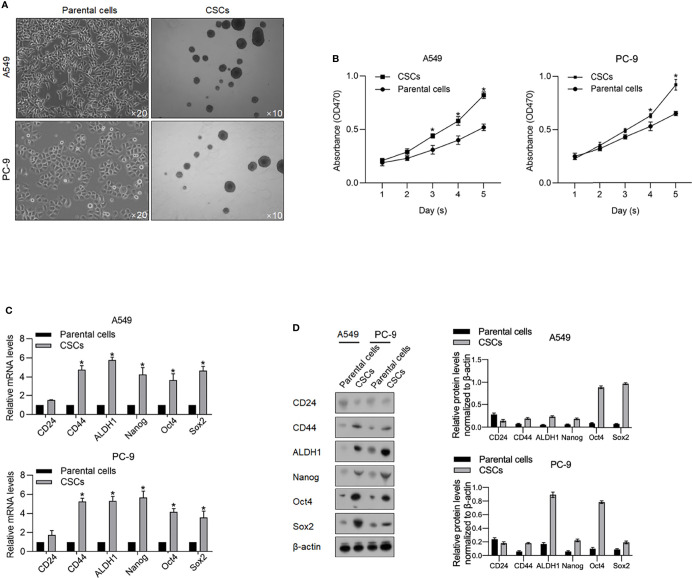
Enrichment of CSCs from A549 and PC-9 cells. **(A)** After being cultured in a serum-free medium, spheres derived from A549 and PC-9 were imaged at an amplification of ×10. **(B)** By performing CCK-8 assay, the cell viability of CSCs and their parental cells from days 1 to 5 was measured. ^*^ *p* < 0.05 vs. the parental cell group. The mRNA **(C)** and protein levels **(D)** of stemness hallmarkers, including CD24, CD44, ALDH1, Nanog, Oct4, and Sox2 were measured by performing RT-qPCR and Western blot analysis. ^*^ *p* < 0.05 vs. parental cell group.

Aiming to investigate the functional role of m^6^A methylation in CSCs derived from lung adenocarcinoma cells, we firstly enriched spheres from A549 and PC9 cells by culturing in a serum-free medium (SFM) (**Figure 1A**). Ten days after being cultured in SFM, spheres (diameter >20 µm) were observed suspended in a medium, which is a characteristic of CSCs. Collected spheres present more vigorous viability from days 1 to 5 in a serum-supplemented medium (**Figure 1B**). We then accessed stem factors, including CD24, CD44, ALDH1, Nanog, Oct4, and Sox2 and expectedly found all these stem factors (**Figures 1C, D**).

**Figure 2:**
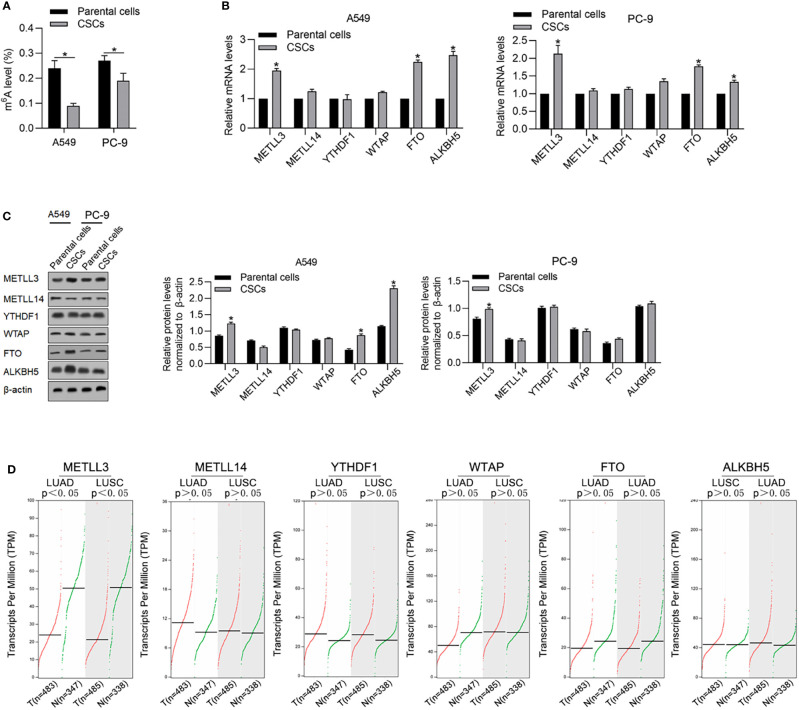
CSCs present a relatively lower m^6^A methylation level compared with their parental cells. **(A)** Global m^6^A methylation was measured in CSCs and its parental cells of A549 and PC-9. ^*^ *p* < 0.05 vs. parental cells. m^6^A methylation-relative gene expressions including METLL3, METLL14, YTHDF1, WTAP, FTO, and ALKBH5 were measured in mRNA **(B)** and protein levels **(C)**. **(D)** By employing the GEPIA database analysis tool, the relative mRNA levels of METLL3, METLL14, YTHDF1, WTAP, FTO, and ALKBH5 were compared between tumor samples and nontumor samples.

By considering the critical roles of m^6^A methylation in regulating CSCs, we tried to determine the expression levels of m^6^A methylation in CSCs compared with that in relevant PCs. As shown in **Figure 2A**, CSCs present a relatively low level of m^6^A methylation compared with that in relevant PCs. We then measured m^6^A methylation regulatory genes, including METTL3, METLL14, YTHDF1, FTO, WTAP, and ALKBH5 by reverse-transcription quantitative PCR (RT-qPCR) and found that METTL3, FTO, and ALKBH5 were comparatively higher in CSCs normalized to PCs (**Figure 2B**). Consistent upregulation of METTL3, FTO, and ALKBH5 in CSCs were also observed by performing Western blot analysis (**Figure 2C**). By performing online database searching of the GEPIA database, although FTO and ALKBH5 present no obvious difference between tumor and adjacent tissue, METLL3 significantly decreased in tumor tissues compared with adjacent tissues (**Figure 2D**).

### ALKBH5 Is Upregulated in CSCs and Enhances Malignancies

**Figure 3:**
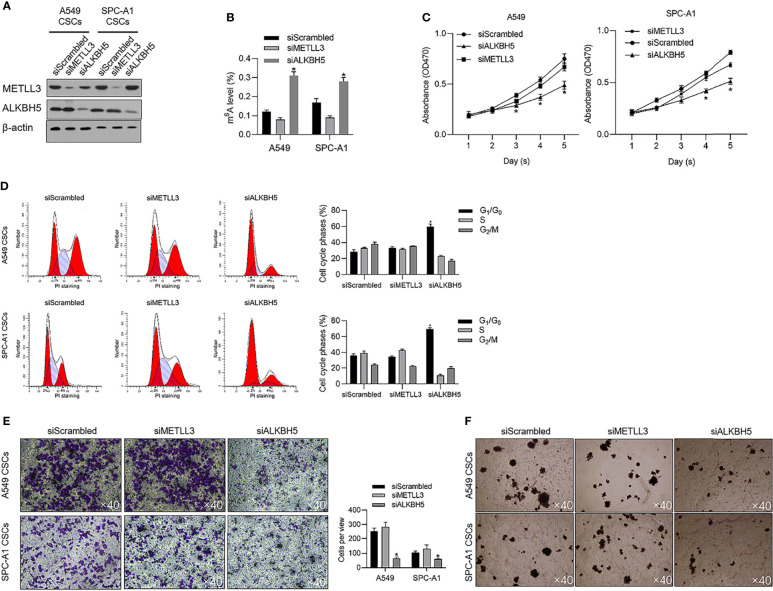
ALKBH5 is tightly associated with global m^6^A methylation level and promotes cell cycle progression and invasive capacity. **(A)** Efficient knockdown of METTL3 or ALKBH5 was confirmed by performing a Western blot analysis. **(B)** After METTL3 or ALKBH5 knockdown, the global m^6^A methylation was measured. **(C)** After METTL3 or ALKBH5 knockdown, cell viability from days 1 to 5 was measured by performing CCK-8 assay. ^*^ *p* < 0.05 vs. the siScrambled group. **(D)** After METTL3 or ALKBH5 knockdown, the cell cycle distribution was measured by performing PI staining followed by flow cytometric analysis. ^*^ *p* < 0.05 vs. siScrambled group. After METTL3 or ALKBH5 knockdown, invasive capacity **(E)** or tumor formation in soft agar **(F)** was respectively analyzed.

**Figure 4:**
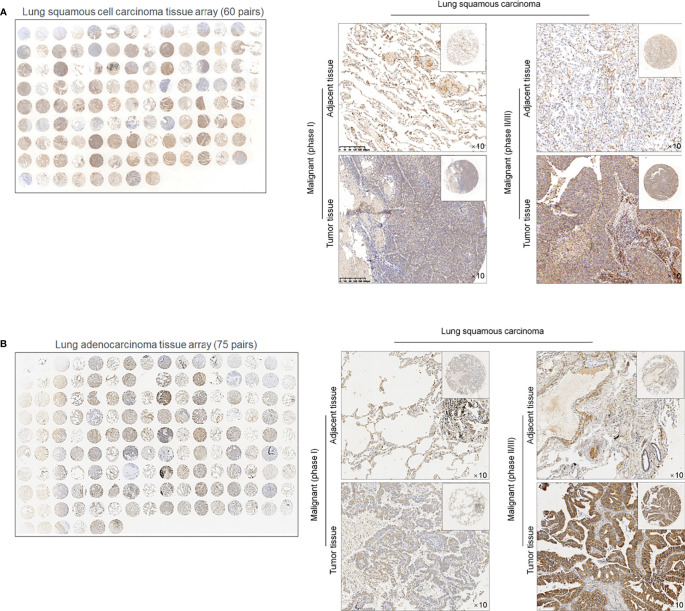
ALKBH5 protein is expressed relatively higher in lung cancer tissues compared to adjacent non-tumor tissues 60 paired lung squamous cell carcinoma (LUAD) tissue array **(A)** and 75 paried lung adenocarcinoma (LUSC) tissue array was employed for ISH staining of ALKBH5 **(B)**.

To evaluate the effects of METTL3 and ALKBH5 on m^6^A methylation in NSCLC CSCs, siRNAs targeting METTL3 or ALKBH5 were transfected efficiently into cells (**Figure 3A**). Seventy-two hours later after transfection, m^6^A methylation level was measured, and, expectedly, results presented that knockdown of ALBKH5 significantly increased m^6^A methylation, and oppositely, knockdown of METLL3 decreased m^6^A methylation (**Figure 3B**). Knockdown of ALKBH5, but not METLL3, significantly inhibited malignant behaviors of CSCs, including cell proliferation (**Figure 3C**), cell cycle promotion (**Figure 3D**), invasion (**Figure 3E**), and colony formation (**Figure 3F**). These results indicate that ALKBH5, but not METLL3, plays a critical regulatory role in malignancies *via* regulating m^6^A methylation in NSCLC CSCs. We then further employed a tissue array to evaluate the protein level of ALKBH5 in LUAD and LUSC. **Figures 4A, B** presents that, compared with adjacent tissues, tumor tissue presents obvious higher intensity of ALKBH5, which indicated that ALKBH5 may be upregulated in tumor tissues compared with adjacent tissues.

### ALKBH5 Is Critical for Maintaining Stemness in CSCs

**Figure 5:**
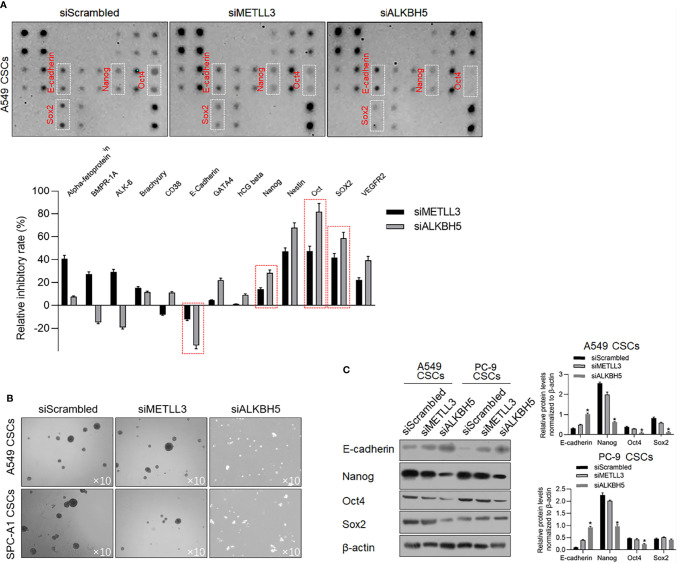
ALKBH5 knockdown increased E-cadherin and decreased stemness. **(A)** To detect the changes of human stem cell hallmarkers after ALKBH5 knockdown, a human stem cell antibody array was performed. **(B)** After METTL3 or ALKBH5 knockdown, sphere formation was measured. **(C)** To confirm the effects of METLL3 or ALKBH5 knockdown on E-cadherin, Nanog, Oct4, and Sox2, a Western blot analysis was performed. ^*^ *p* < 0.05 vs. siScrambled group.

To determine the effects of METLL3 and ALKBH5 on the stem-like property of NSCLC CSCs, a stem factor protein array was employed after METTL3 or ALKBH5 knockdown. After METTL3 and ALKBH5 knockdown, E-cadherin protein level was significantly increased, and Nanog, Oct4, and Sox2 were decreased significantly (**Figure 5A**), indicating that knockdown of METLL3 or ALKBH5 potentially regulates stemness of NSCLC CSCs. Sphere formation was further performed to confirm their role in regulating stemness, and, expectedly, results presented that knockdown of either METLL3 or ALKBH5 inhibited sphere formation (**Figure 5B**). Changes in E-cadherin, Nanog, Oct4, and Sox2 indicated that these two proteins, especially ALKBH5 are critical for maintaining stemness (**Figure 5C**). Knockdown of ALKBH5 significantly modified hallmarkers of EMT, including E-cadherin, Nanog, Oct4, and Sox2, indicating its critical role in maintaining the stemness of CSCs.

### P53 Transcriptionally Regulates ALKBH5 and Malignancies in CSCs

**Figure 6:**
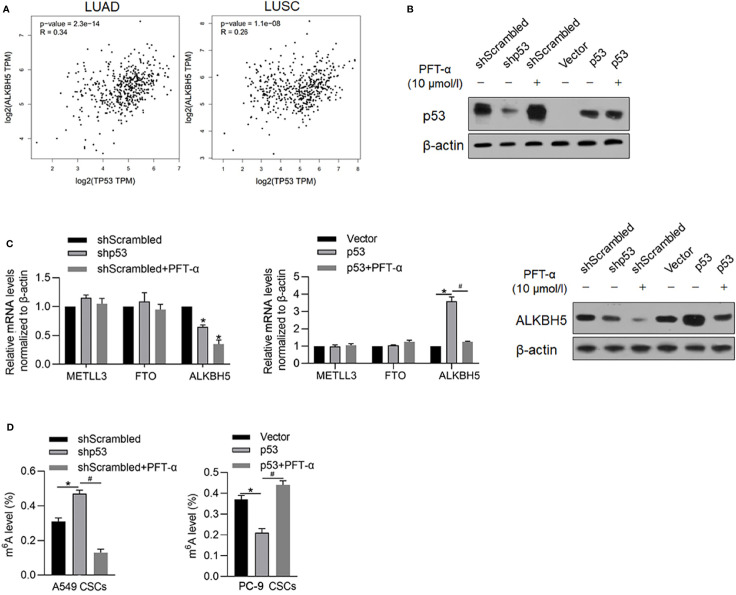
p53 transcriptionally regulates ALKBH5 and subsequently decreased m^6^A methylation. **(A)** GEPIA database analysis tool was used to compare the correlation between ALKBH5 and p53 in LUAD and LUSC. **(B)** In A549 and PC-9 CSCs, the efficiency of p53 knockdown or overexpression was measured by performing a Western blot analysis. **(C)** After modification of p53 with or without PFT-α, ALKBH5 mRNA and protein level were measured. ^*^ *p* < 0.05 vs. shScrambled group; ^#^ *p* < 0.05 vs. p53 group. **(D)** After modification of p53 with or without PFT-α, m^6^A methylation level was measured. ^*^ *p* < 0.05 vs. shScrambled group; ^#^ *p* < 0.05 vs. shp53 group (left panel); ^*^ *p* < 0.05, vs. vector group; ^#^ *p* < 0.05 vs. p53 group (right panel).

It is identified that ALKBH5 is a downstream target gene of p53, which is a key regulator of malignancies in various cancers. By searching the GEPIA database, it is illustrated that, both in LUAD and LUSA, mRNA levels of p53 are positively correlated with that of ALKBH5 (**Figure 6A**), which prompted us to identify the regulatory role of p53 on ALBKH5. To identify the effects of p53 on m^6^A methylation, we efficiently knocked down p53 in A549 (**Figure 6B**) and overexpressed p53 in PC-9, which is a p53-null cell line (**Figure 6B**). After efficient knockdown of p53, ALKBH5 mRNA was significantly downregulated (**Figure 6C**), which was also observed after blockage of p53’s transcriptional activity *via* inhibiting its DNA-binding activity (**Figure 6C**). We then further analyzed the m^6^A methylation level after interfering with p53 activity. Expectedly, both p53 knockdown and inhibition of p53’s transcriptional activity significantly increased the m^6^A methylation in NSCLC CSCs (**Figure 6D**).

**Figure 7:**
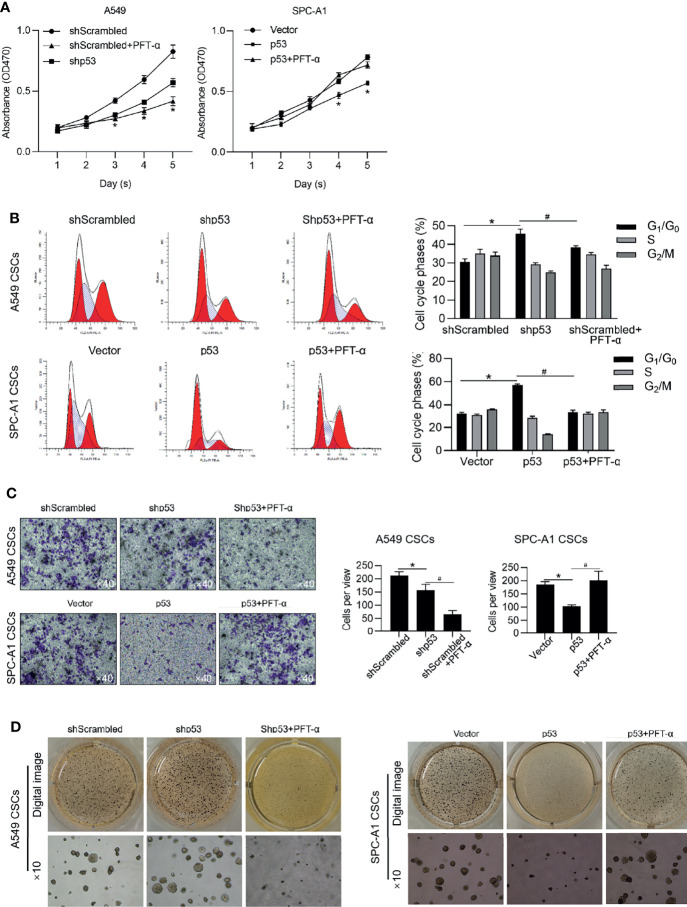
p53 inhibits malignancies *via* exerting transcriptional activity. In A549 CSCs, p53 was efficiently knockdown by transfecting shRNA targeting to p53 mRNA. In PC-9 CSCs, p53 was efficiently overexpressed by transfecting p53-coding plasmid. Cell viability was then analyzed by performing CCK-8 assay **(A)**. ^*^ *p* < 0.05 vs. shScrambled group (left panel); ^*^ *p* < 0.05 vs. vector group (right panel). **(B)** Cell cycle distribution was analyzed by performing PI staining followed by flow cytometry assay. ^*^ *p* < 0.05 vs. shScrambled group; ^#^ *p* < 0.05 vs. shp53 group (left panel); ^*^ *p* < 0.05 vs. vector group; ^#^ *p* < 0.05 vs. p53 group (right panel). **(C)** Cell invasion was analyzed by performing Transwell assay. ^*^ *p*<0.05 vs. shScrambled group; ^#^ *p* < 0.05, vs. shp53 group (left panel); ^*^ *p* < 0.05, vs. vector group; ^#^ *p* < 0.05 vs. p53 group (right panel). **(D)** Tumor formation in soft agar was performed. ^*^ *p* < 0.05 vs. shScrambled group; ^#^ *p* < 0.05 vs. shp53 group (left panel); ^*^ *p* < 0.05 vs. vector group; ^#^ *p* < 0.05 vs. p53 group (right panel).

We further analyzed the roles of p53 on malignancies of NSCLC CSCs *via* regulating p53 transcriptional activity. Expectedly, in A549 CSCs, knockdown of p53 slightly inhibited malignancies, including cell proliferation, entry of cell cycle, invasion, and tumor formation *in vitro* (**Figures 7A**–**D**), which were achieved by the addition of PFT-α, indicating that the roles of p53 in regulating malignancies were due to its transcriptional activity. In PC-9 CSCs, overexpression of p53 significantly inhibited malignancies in PC-9 CSCs, which were reversed by the addition of PFT-α, indicating that p53 may, at least, partially regulates malignancies *via* ALKBH5 in NSCLC CSCs (**Figures 7A**–**D**).

### P53 Potentially Regulates EMT and Stemness *via* PRRX1

**Figure 8:**
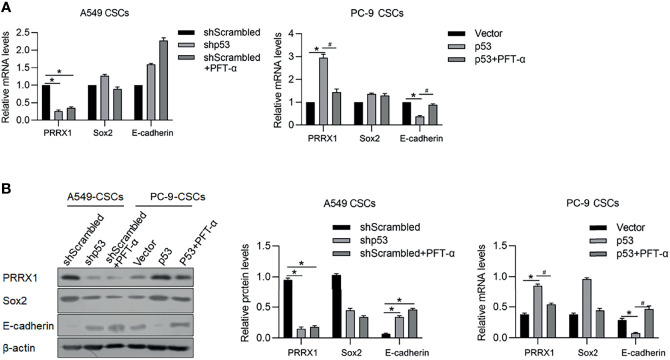
p53 potentially regulates PRRX1, Sox2, and E-cadherin *via* ALKBH5. In A549 CSCs, p53 was efficiently knockdown by transfecting shRNA targeting p53 mRNA. In PC-9 CSCs, p53 was efficiently overexpressed by transfecting p53-coding plasmid. mRNA **(A)** and protein **(B)** of PRRX1, Sox2, and E-cadherin were measured. ^*^ *p* < 0.05 shScrambled group (left panel); ^*^ *p* < 0.05 vs. vector group; ^#^ *p* < 0.05 vs. p53 group (right panel).

It is reported that p53 functions as a transcriptional factor of PRRX1 and E-cadherin by targeting their upstream promoter region. In our previous study, PRRX1 was observed to be a negative regulator of E-cadherin, which is a marker of EMT. These results prompted us to detect the regulatory roles of p53 on PRRX1 and E-cadherin. As the results presented, knockdown of p53 significantly decreased PRRX1 mRNA and oppositely increased E-cadherin mRNA in A549 CSCs (**Figure 8A**). Overexpression of p53 in PC-9 CSCs increased PRRX1 mRNA and decreased E-cadherin mRNA, which were reversed by the addition of PFT-a, indicating that p53’s transcriptional activity is critical for its regulatory roles on PRRX1 and E-cadherin (**Figure 8A**). These changes in protein levels were further confirmed by performing a Western blot analysis (**Figure 8B**). Taken together, p53 may act as a key regulator of PRRX1, ALKBH5, and E-cadherin.

**Figure 9:**
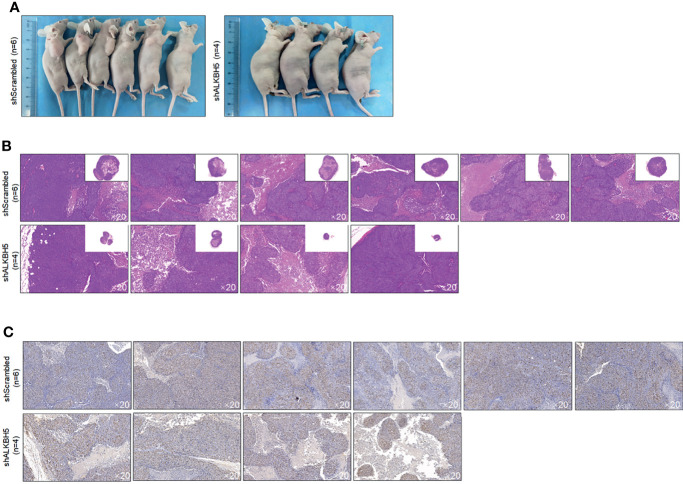
ALKBH5 knockdown decreased tumor formation in nude mice. **(A)** Volume of CSCs formed tumors in nude mice after seeding 5 × 10^5^ cells. Tumor formation was morphologically identified by H&E staining **(B)**, and cell proliferation marker Ki67 **(C)** were further measured.

To further confirm the roles of ALKBH5 in regulating tumor growth *in vivo*, A549 CSCs with knocked-down ALKBH5 were planted in nude mice. Twenty-eight days later, transplanted tumors in the ALKBH5 knockdown group were presented as significantly smaller than the control group (**Figures 9A, B**). By performing Ki67 staining to evaluate the proliferative capacity of tumors, expectedly, knockdown of Ki67 obviously decreased the Ki67-positive proportion in tumors (**Figure 9C**).

## Discussion

In this study, we presented that m^6^A demethylase ALKBH5, which is transcriptionally regulated by p53, modifies the epithelial and mesenchymal transition process and maintenance of stemness of cancer stem-like cells derived from NSCLC. In our previous report, it is presented that PRRX1 regulates the stemness phenotype and EMT of CSCs derived from NSCLC *via* stabilizing Sox2 (7). **Figures 5**, **8** present that downregulation of ALKBH5 or p53 significantly decreased Sox2 and increased E-cadherin protein levels, which is consistent with our previous findings and demonstrated the upstream regulating roles of ALKBH5 in modulating malignancies of CSCs derived from NSCLC.

PRRX1 is a member of the paired homeobox family, which plays an important role in tumors. EMT and mesenchymal–epithelial transition (MET) are two important mechanisms leading to tumor recurrence and metastasis. EMT can make tumor cells acquire the ability of invasion and migration, and promote the dissemination of tumor cells from the primary lesion, which is one of the initial steps of tumor recurrence and metastasis; MET can make tumor cells regain the ability of colonization and formation of tumor metastasis, then the cells resurge to complete tumor metastasis. At the same time, tumor IMT can make the tumor acquire stemness, and the two are correlated. PRRX1 is closely related to the occurrence of EMT, and it is an important transcription factor regulating EMT, which plays an important role in tumor recurrence and metastasis and tumor stemness maintenance (27). Studies have shown that overexpressed PRRX1 can induce EMT in hepatoma cells by activating the TGF-β pathway to promote β-catenin entry into the nucleus, while downregulation of Prrx1 can inhibit the expression of Slug and TGF-β-R2 by upregulating Pitx2-microRNA pathway, then relieve the inhibition of Slug on E-cadherin, thus leading to the occurrence of MET in hepatoma cells, which is closely related to metastasis and recurrence of liver cancer (28). The role of PRRX1 varies in tumors of different systems and types. In liver cancer, downregulation of PRRX1 can promote tumors to produce stem cell-like features (29). However, it is found that overexpression of PRRX1 is closely related to tumor EMT, tumor stemness, tumor metastasis, and prognosis in pancreatic cancer (30), colorectal cancer (26), and papillary thyroid cancer (31). The above differences may be related to the differences in PRRX1’s expression and function in different tumors.

In our previous report, main PRRX1 isoforms were investigated, and results found that PRRX1A, the main type of PRRX1 isoforms, promotes malignant behaviors *via* transcriptional activation of TGF-β depending on TGF-β/TGF-βR signaling pathway, and subsequently regulates EMT process (7). Meanwhile, PRRX1A tightly binds to and stabilizes Sox2, which may be the main cause of the decrease in stemness. Here, it is found that downregulation of ALKBH5, but not METLL3, significantly increased E-cadherin and decreased Oct4 and Sox2 proteins, which indicated its regulatory roles in the EMT process and maintenance of stemness. This indicates that ALKBH5 may be a direct regulator of PRRX1 and results in subsequent modification of EMT and stemness *via* PRRX1. However, we failed to evaluate the mRNA and protein levels of different isotypes of PRRX1, including PRRX1A and PRRX1B, which should be evaluated in further study.

m6A RNA methylation is involved in the phenotype of tumor stem cells by regulating gene expression. YTHDF2 is an m^6^A reader, which has been proved to be associated with the prognosis of patients with hepatocellular carcinoma. Some studies suggest that YTHDF2 can promote the stemness phenotype of hepatoma cells by regulating the m^6^A methylation of the Oct4 gene (32). At the same time, m^6^A methylation can enhance the stemness of hepatoma cells by upregulating LINC00106 (33). In addition, m^6^A methylation can also improve the stemness of hepatoma cells by regulating the stability of MGAT5 mRNA (34). In this study, we evaluated the protein levels of the main components of m^6^A methylation, including METLL3, METLL14, YTHDF1, WTAP, FTO, and ALKBH5. Only METTL3 and ALKBH5 are upregulated in CSCs compared with that in parental cells. Moreover, only knockdown of ALKBH5 significantly regulates m^6^A methylation, stemness, EMT, and malignancies together in NSCLC CSCs. ALKBH5 was reported to exert opposite roles in different kinds of cancers, indicating that it may regulate different m^6^A methylation profiles in a different context.

P53, as a kind of tumor suppressor protein, can maintain the balance between self-renewal and differentiation, thus maintaining the stability of the internal environment of organs. In recent years, some studies have shown that the gain-of-function (GOF) mutation of p53 further improves the stemness of cancer cells (35). In addition to posttranslational regulation of p53 protein’s stability and activity, p53 expression is also regulated by promoter methylation and m^6^A RNA methylation at the transcriptional and posttranscriptional levels. Studies have shown that a food-borne mycotoxin FA can reduce P53 expression in human liver cancer cells by reducing the expression level of m^6^A-p53 (36). In addition, the m^6^A demethylase ALKBH5 can activate PER1 *via* an m^6^A-ythdf2-dependent manner and reactivate the ATM-CHK2-P53 #CDC25C signaling pathway, inhibiting the growth of tumor cells. We presented that in LUAD and LUSC tissue samples, ALKBH5 is positively correlated with p53 and transcriptionally regulated by p53. Knockdown of p53 resulted in ALKBH5 decrease and global m^6^A methylation increase. These results demonstrated that ALKBH5 may be a downstream regulator of p53.

Our findings collectively show that ALKBH5 regulates EMT and maintenance of stemness of CSCs derived from NSCLC. P53 was found to act as an upstream regulator of ALKBH5 and exerts key roles in these processes. Targeting the p53-ALKBH5-PRRX1 axes may offer a promising therapeutic approach in curing metastatic NSCLC.

## Data Availability Statement

The original contributions presented in the study are included in the article/**Supplementary Material**. Further inquiries can be directed to the corresponding author.

## Ethics Statement

The animal study was reviewed and approved by the medical ethics committee of the Shanghai Outdo Biotech Company.

## Author Contributions

XL, ZW, and WL designed the experiments and performed molecular-related experiments in this study. QY, XH, and QF performed experiments on processing cells. QY, QF, and XZ are responsible for data collection and performed statistical analysis. XL and WL wrote the manuscript. WL revised the manuscript. All authors read and approved the final manuscript.

## Conflict of Interest

The authors declare that the research was conducted in the absence of any commercial or financial relationships that could be construed as a potential conflict of interest.

## Publisher’s Note

All claims expressed in this article are solely those of the authors and do not necessarily represent those of their affiliated organizations, or those of the publisher, the editors and the reviewers. Any product that may be evaluated in this article, or claim that may be made by its manufacturer, is not guaranteed or endorsed by the publisher.
