# Supplementary figures and images for: RNA Demethylase ALKBH5 Prevents Lung Cancer Progression by Regulating EMT and Stemness via Regulating p53 (part 1 of 2)
Source: Front Oncol. 2022 Apr 22;12:858694. doi: 10.3389/fonc.2022.858694 (PMC9076132; doi:10.3389/fonc.2022.858694)

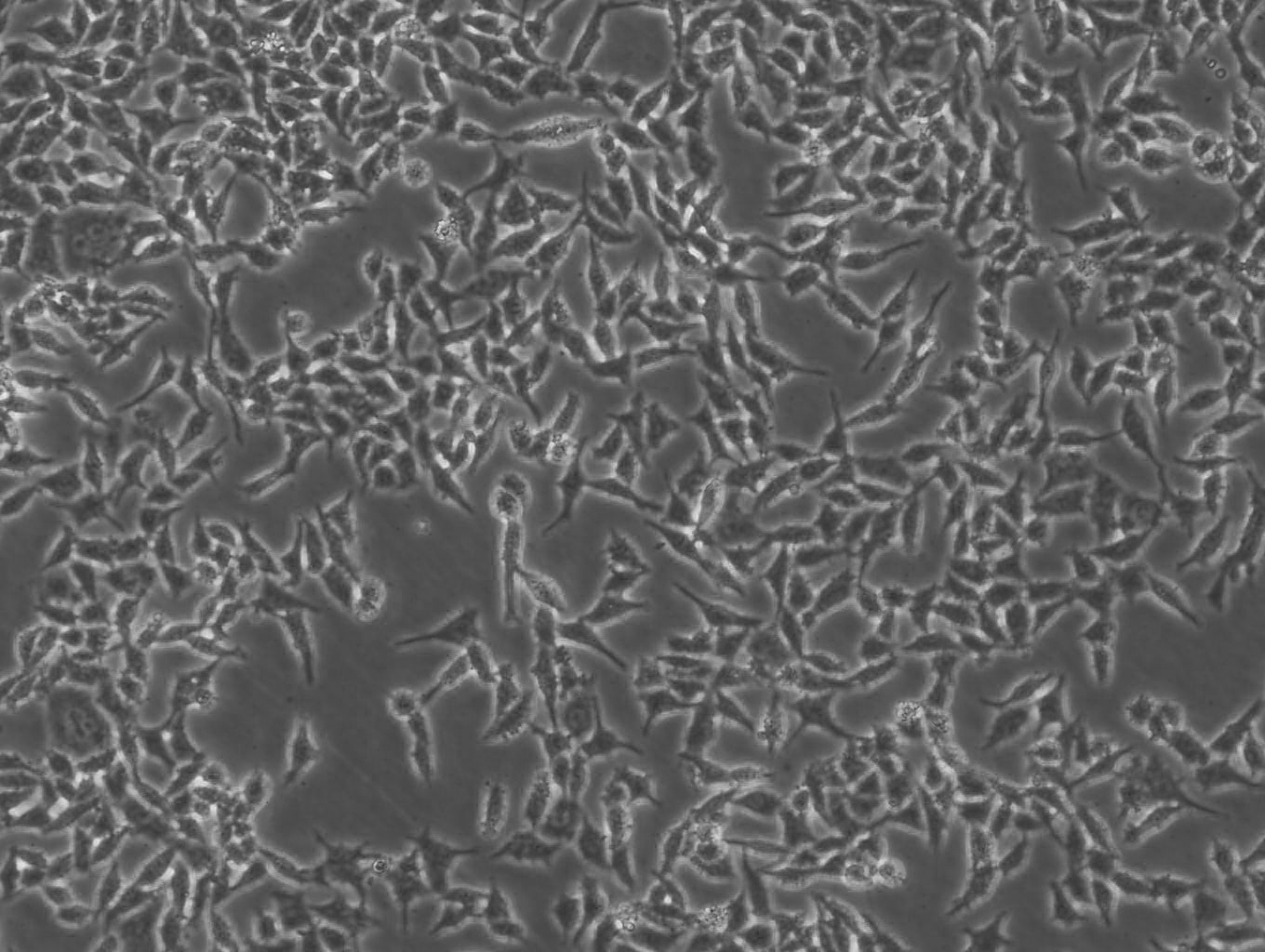

Supplement: Supplementary file 1 [file DataSheet_1.zip › fig 1a. A549 80% confluence.jpg]

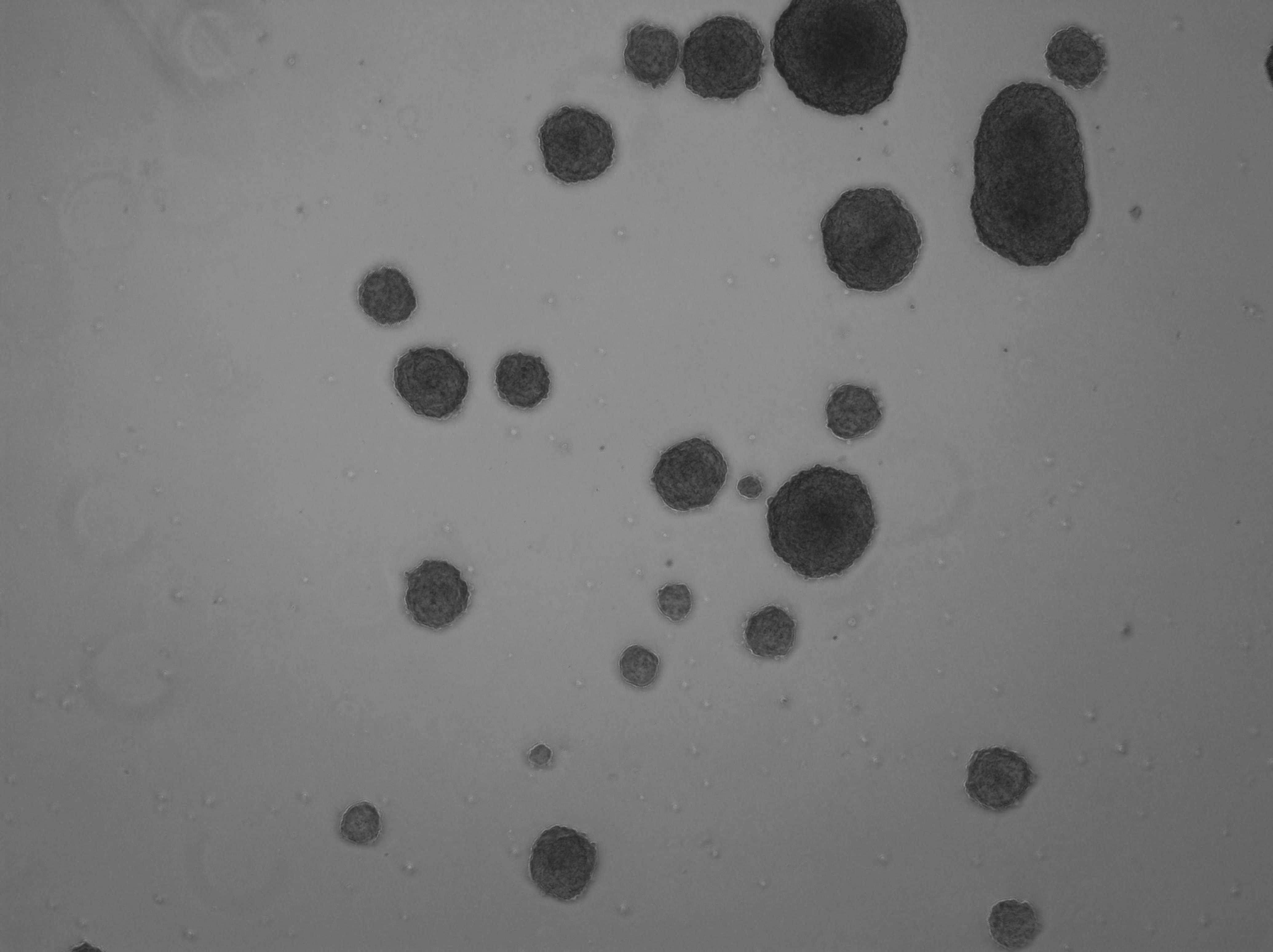

Supplement: Supplementary file 1 [file DataSheet_1.zip › fig 1a. A549 CSCs.jpg]

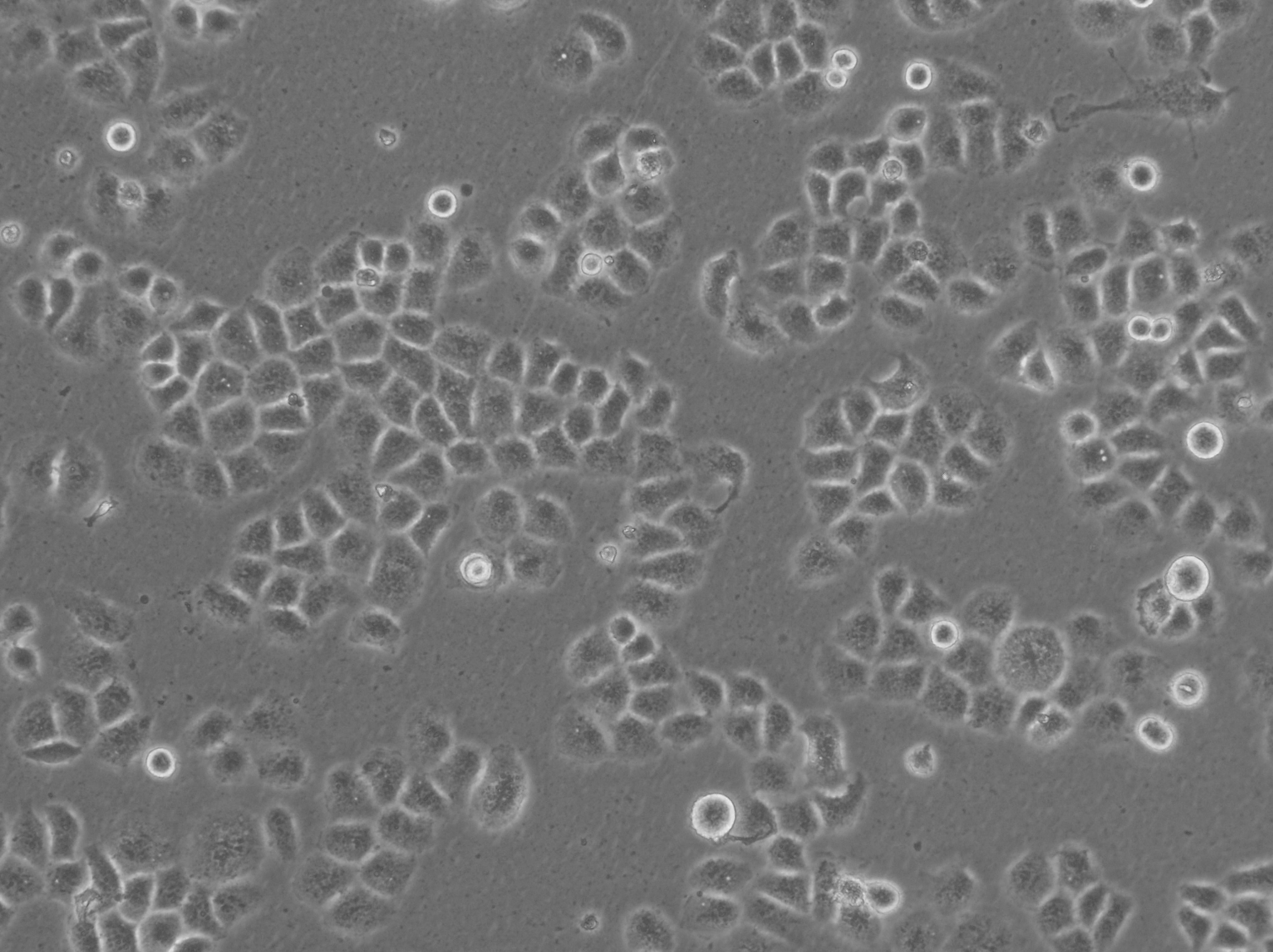

Supplement: Supplementary file 1 [file DataSheet_1.zip › fig 1a. PC-9 80%.jpg]

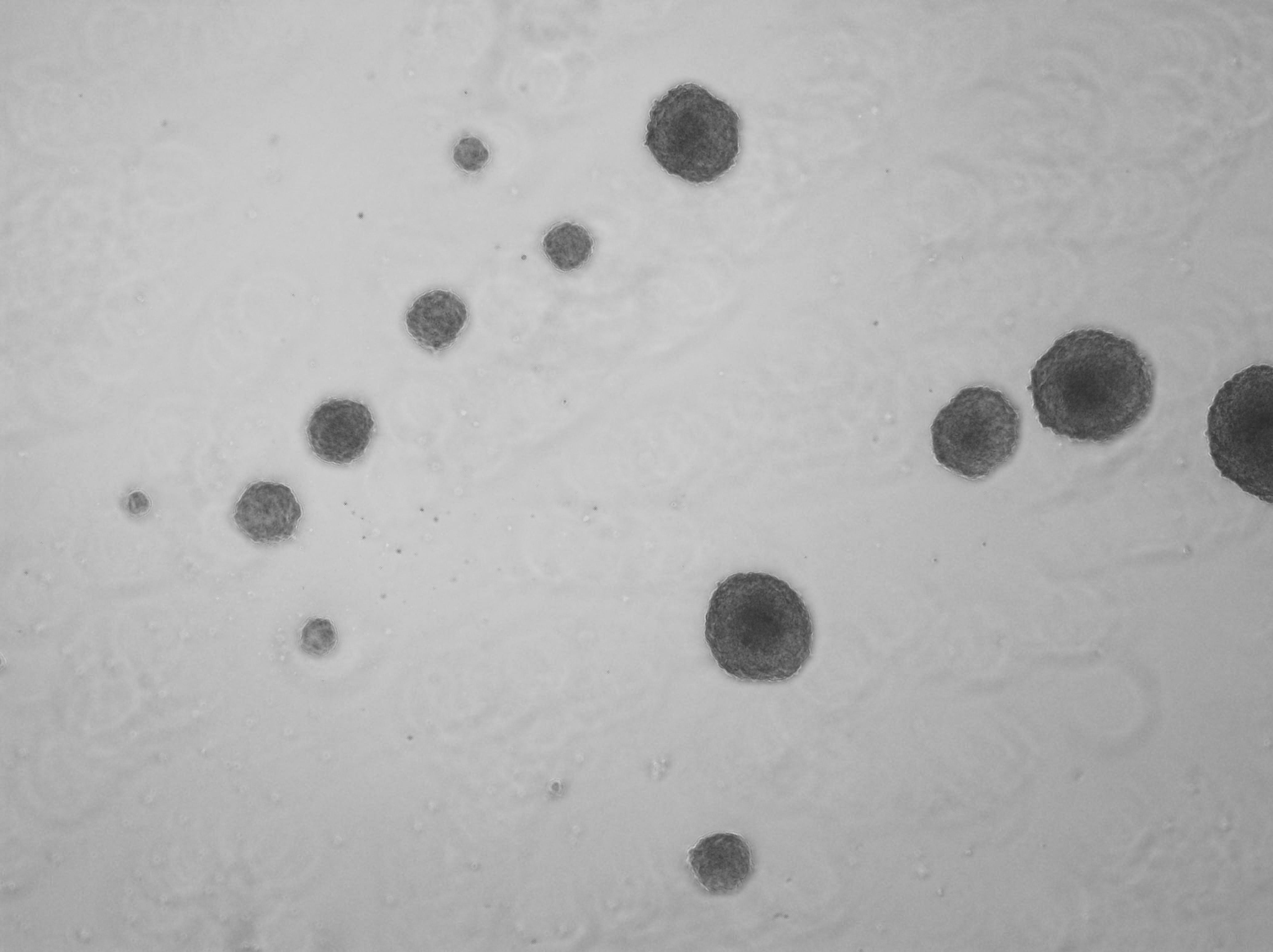

Supplement: Supplementary file 1 [file DataSheet_1.zip › fig 1a. PC-9 CSCs.jpg]

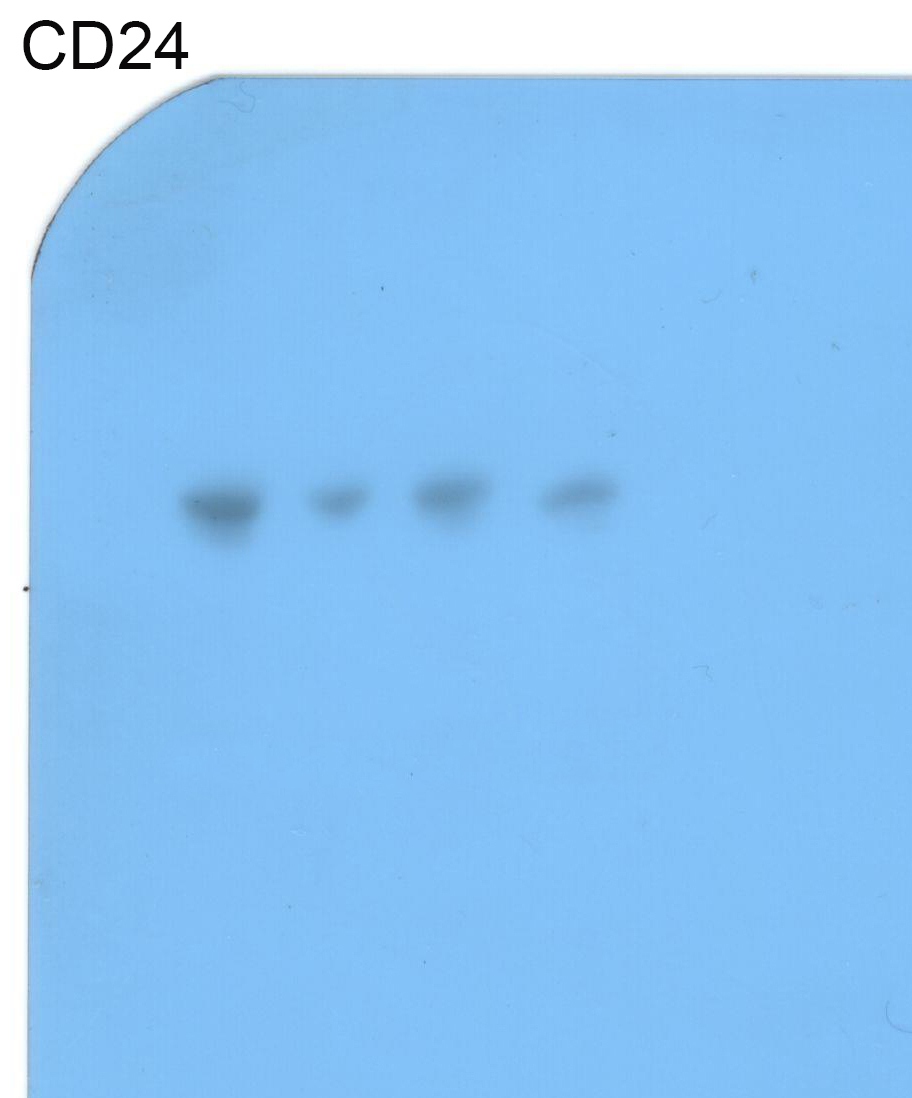

Supplement: Supplementary file 1 [file DataSheet_1.zip › fig 1d. CD24.jpg]

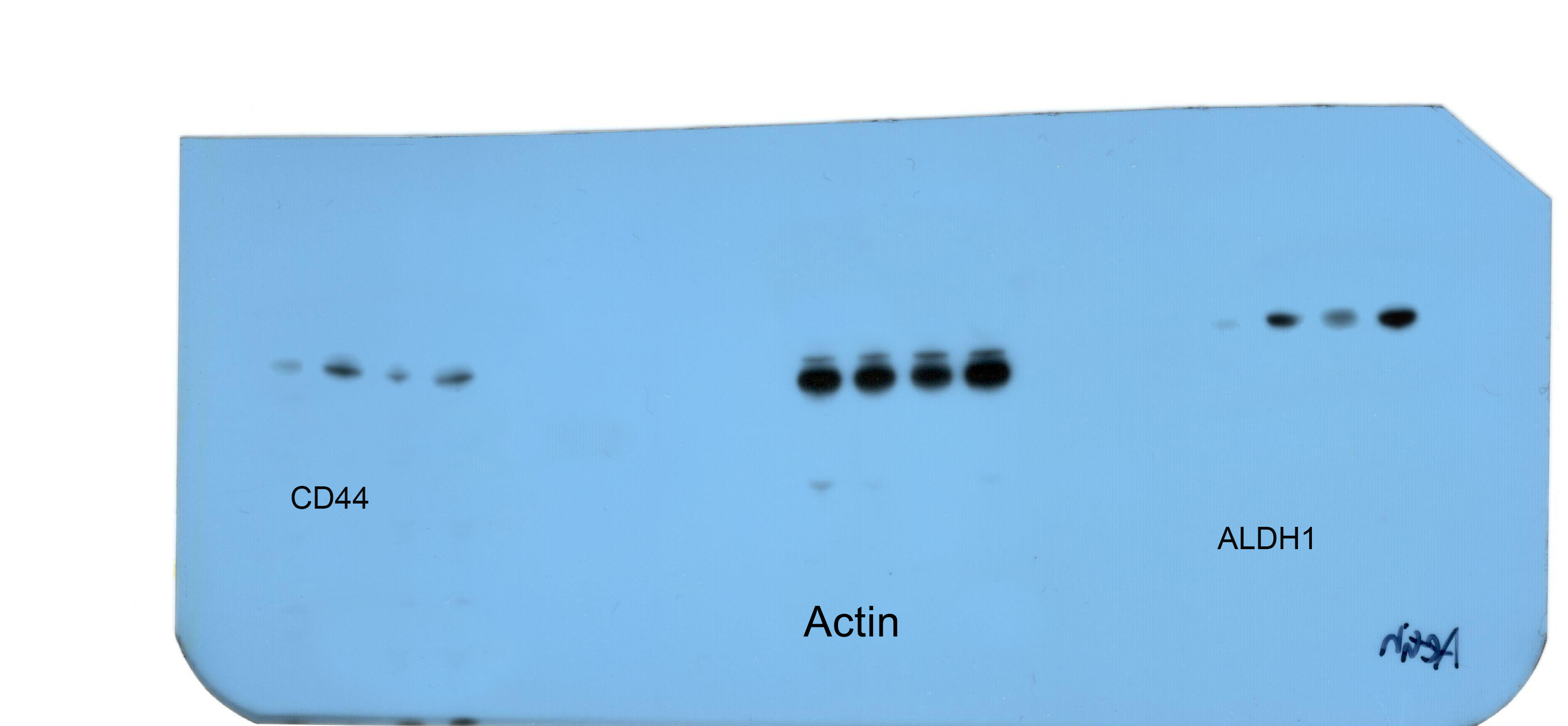

Supplement: Supplementary file 1 [file DataSheet_1.zip › fig 1d. CD44, actin, ALDH1.jpg]

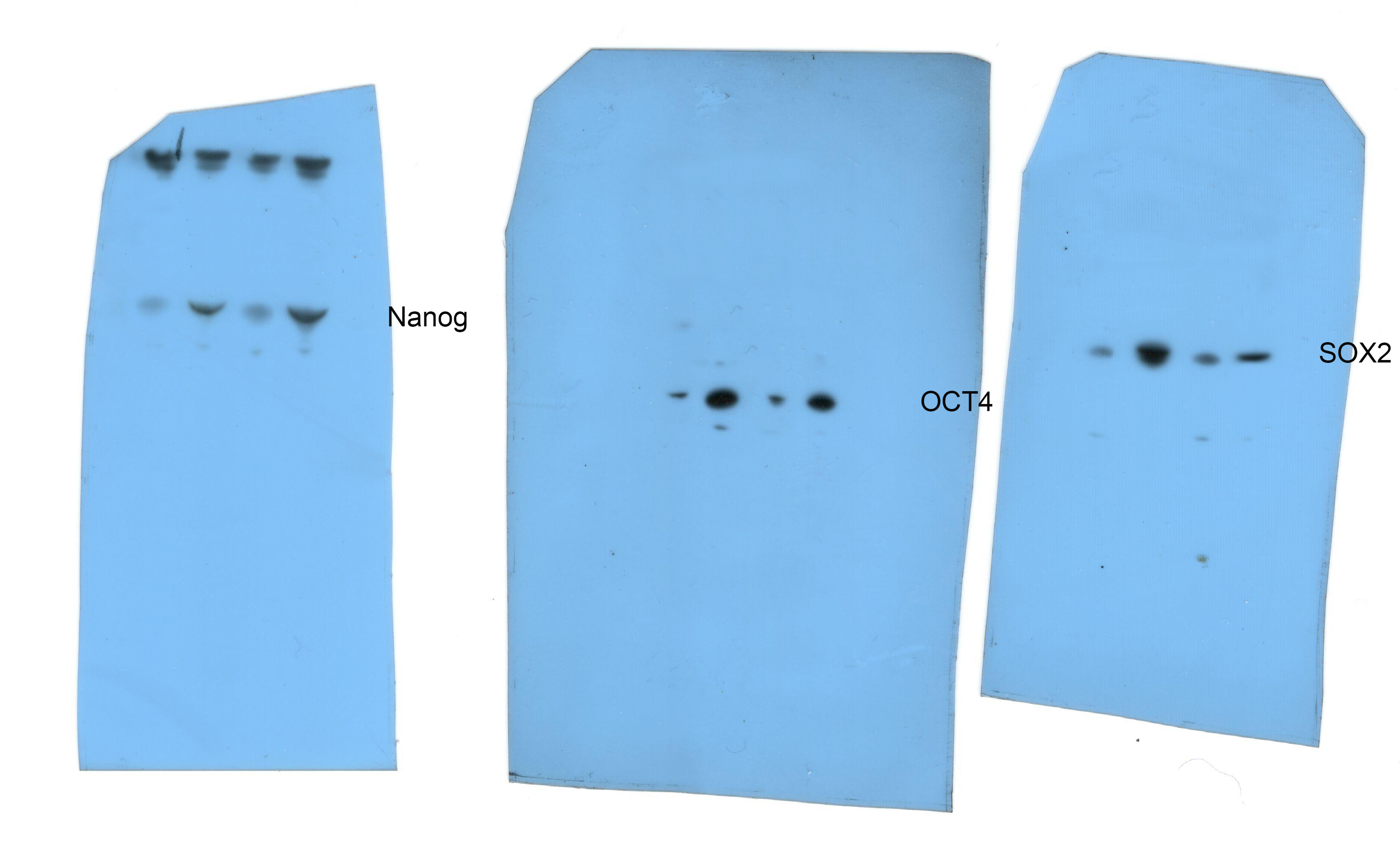

Supplement: Supplementary file 1 [file DataSheet_1.zip › fig 1d. nanog, oct4.jpg]

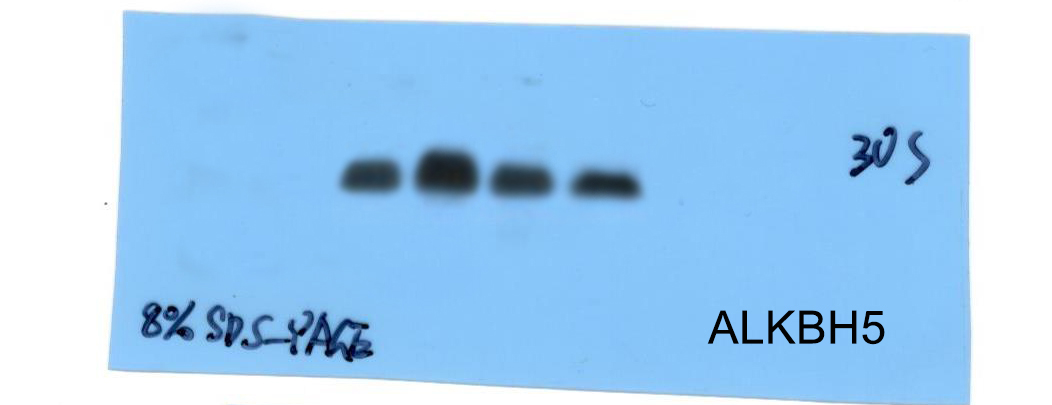

Supplement: Supplementary file 1 [file DataSheet_1.zip › fig 2c. ALKBH5.jpg]

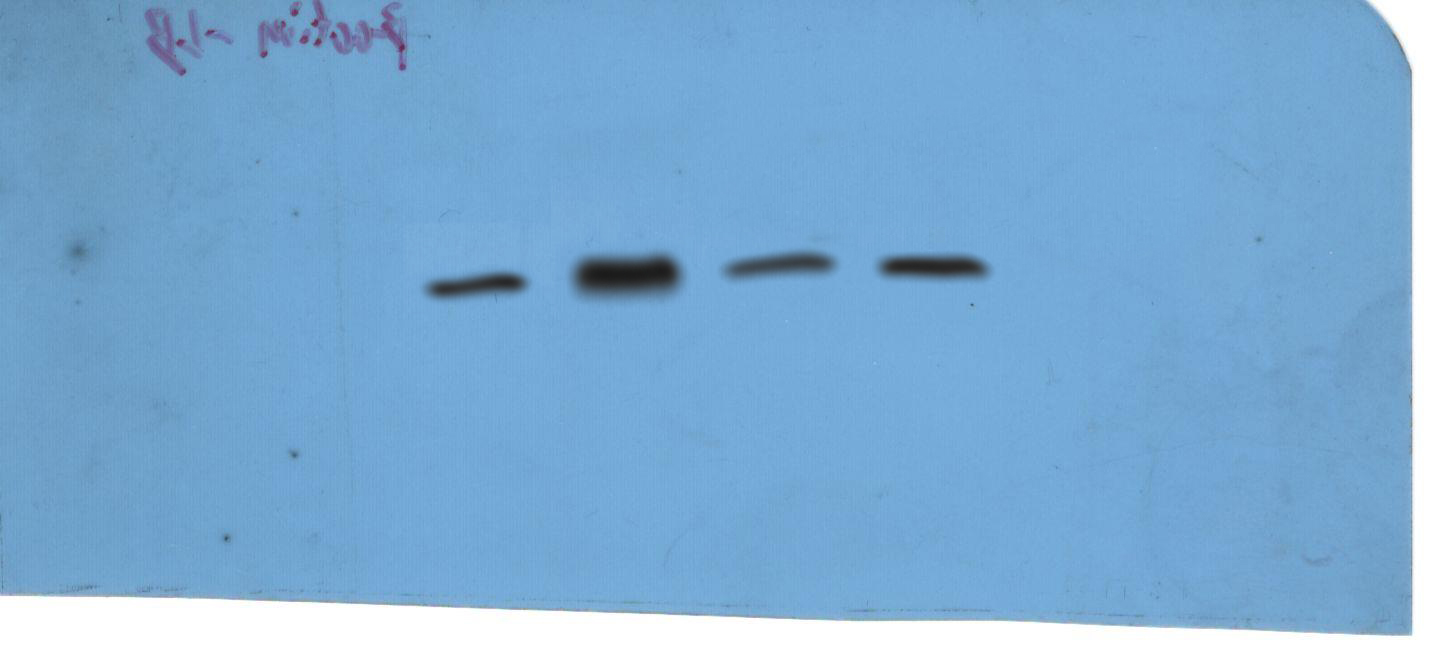

Supplement: Supplementary file 1 [file DataSheet_1.zip › fig 2c. FTO.jpg]

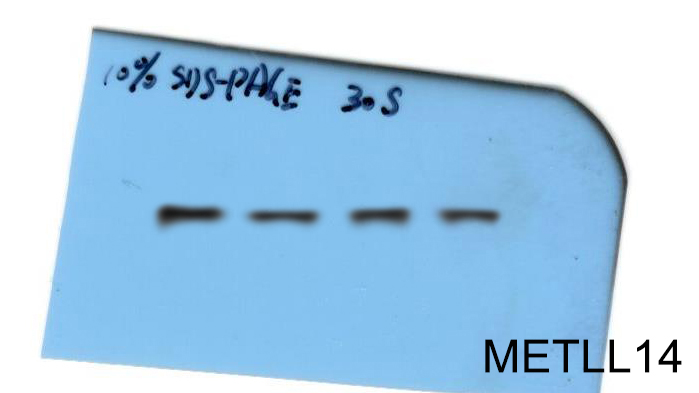

Supplement: Supplementary file 1 [file DataSheet_1.zip › fig 2c. METLL14.jpg]

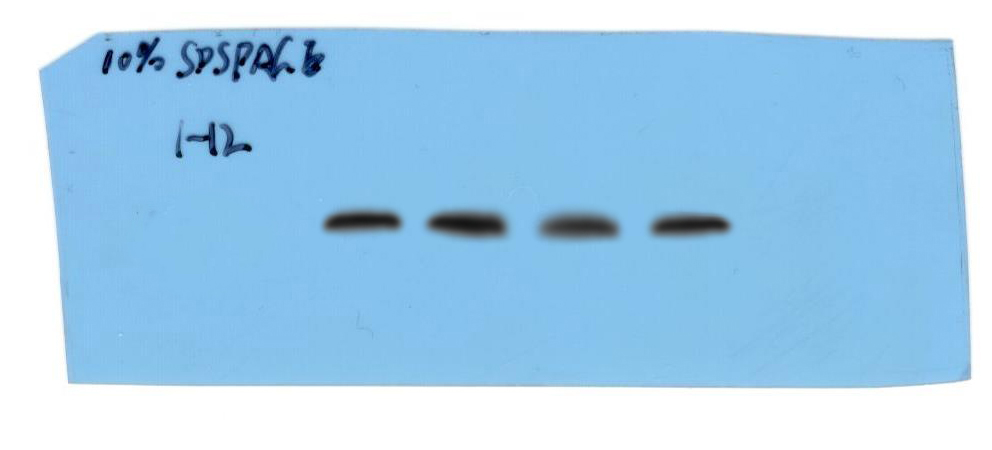

Supplement: Supplementary file 1 [file DataSheet_1.zip › fig 2c. WTAP.jpg]

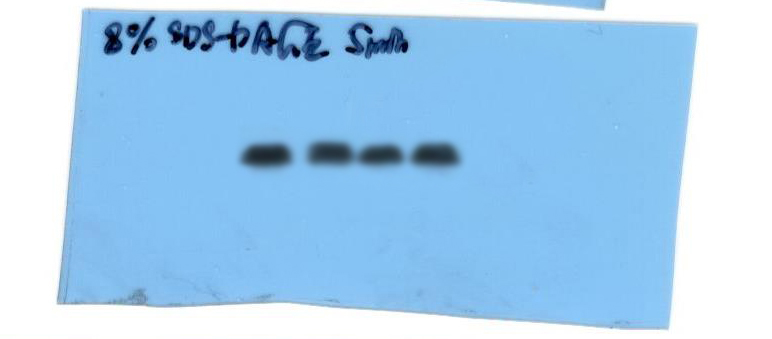

Supplement: Supplementary file 1 [file DataSheet_1.zip › fig 2c. YTHDF1.jpg]

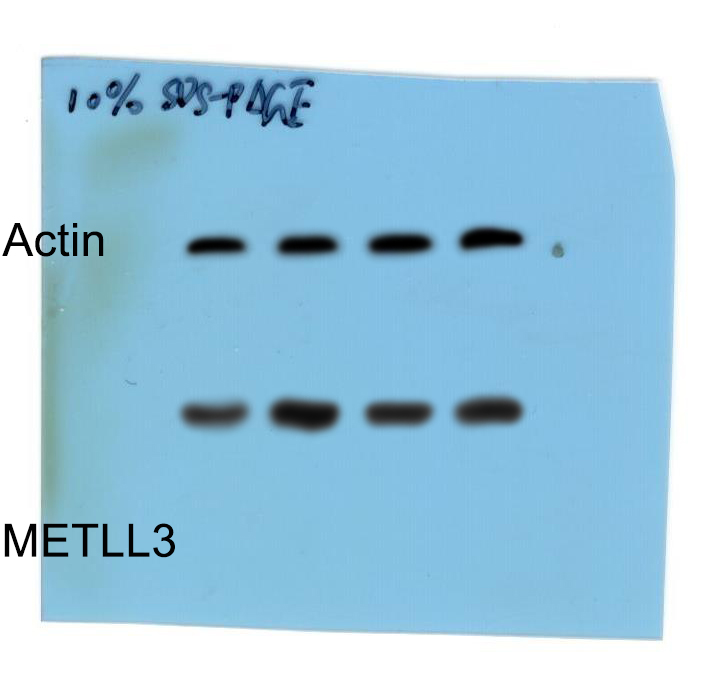

Supplement: Supplementary file 1 [file DataSheet_1.zip › fig 2c. actin, METLL3.jpg]

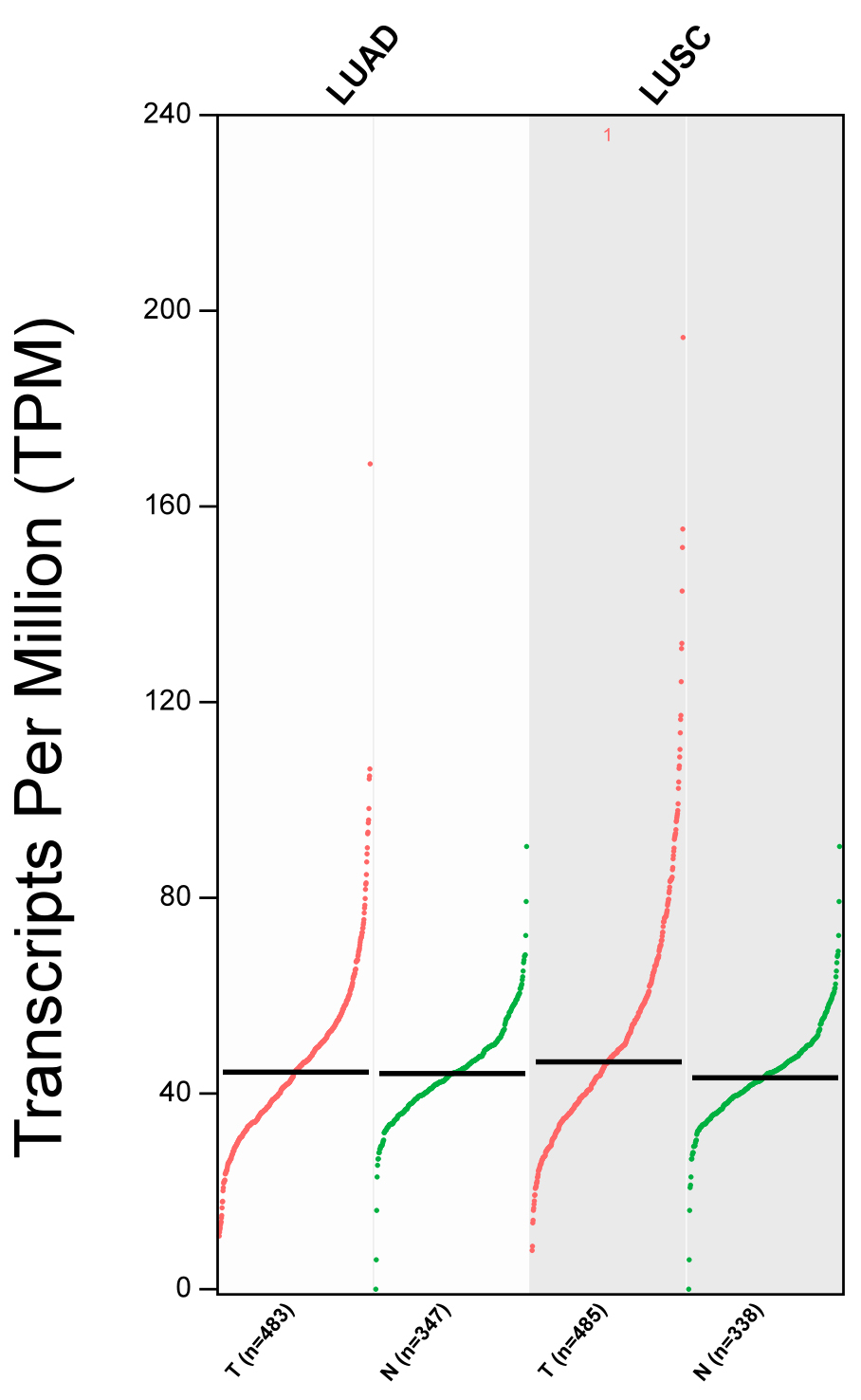

Supplement: Supplementary file 1 [file DataSheet_1.zip › fig 2d. ALKBH5 DEG.jpg]

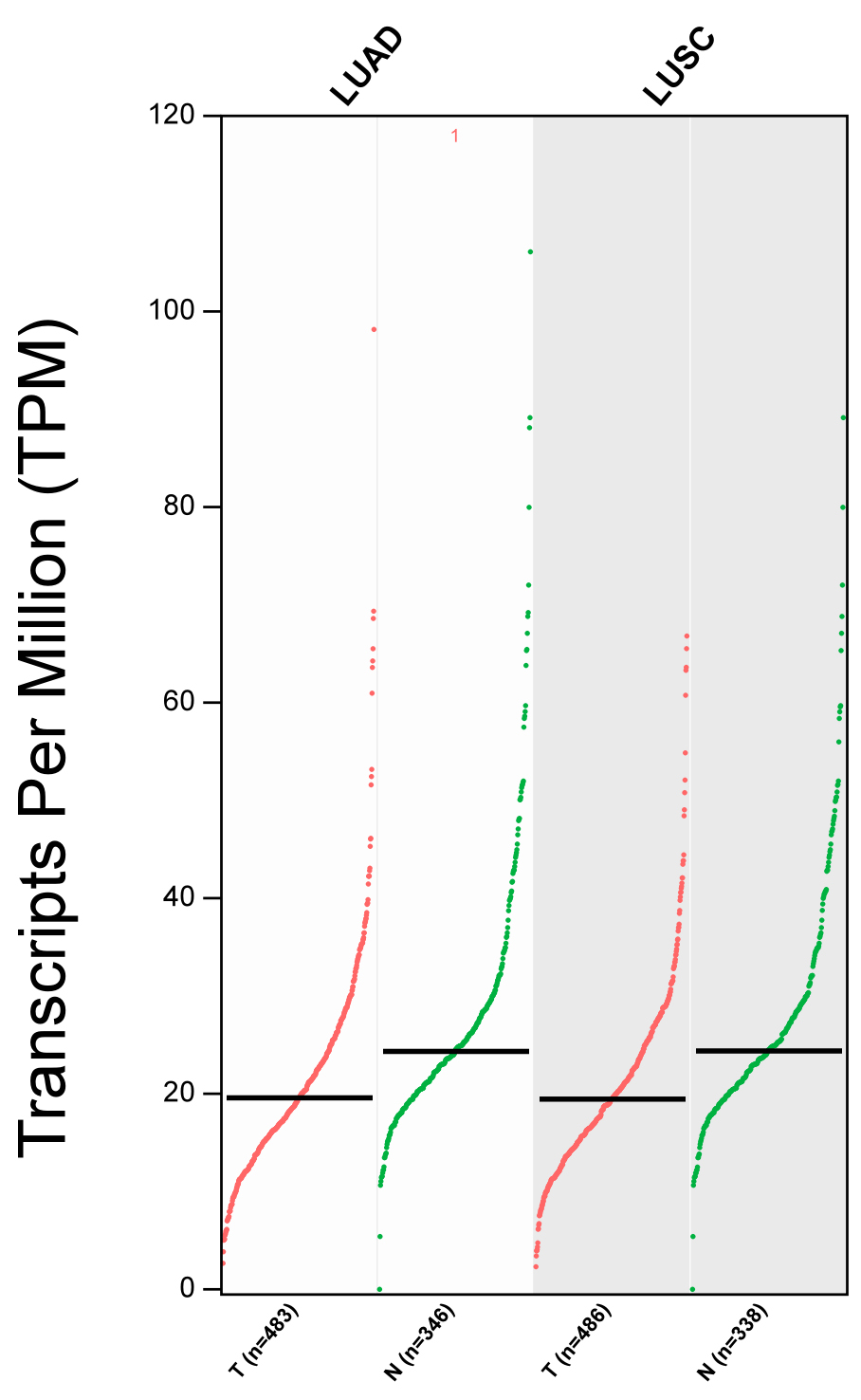

Supplement: Supplementary file 1 [file DataSheet_1.zip › fig 2d. FTO.jpg]

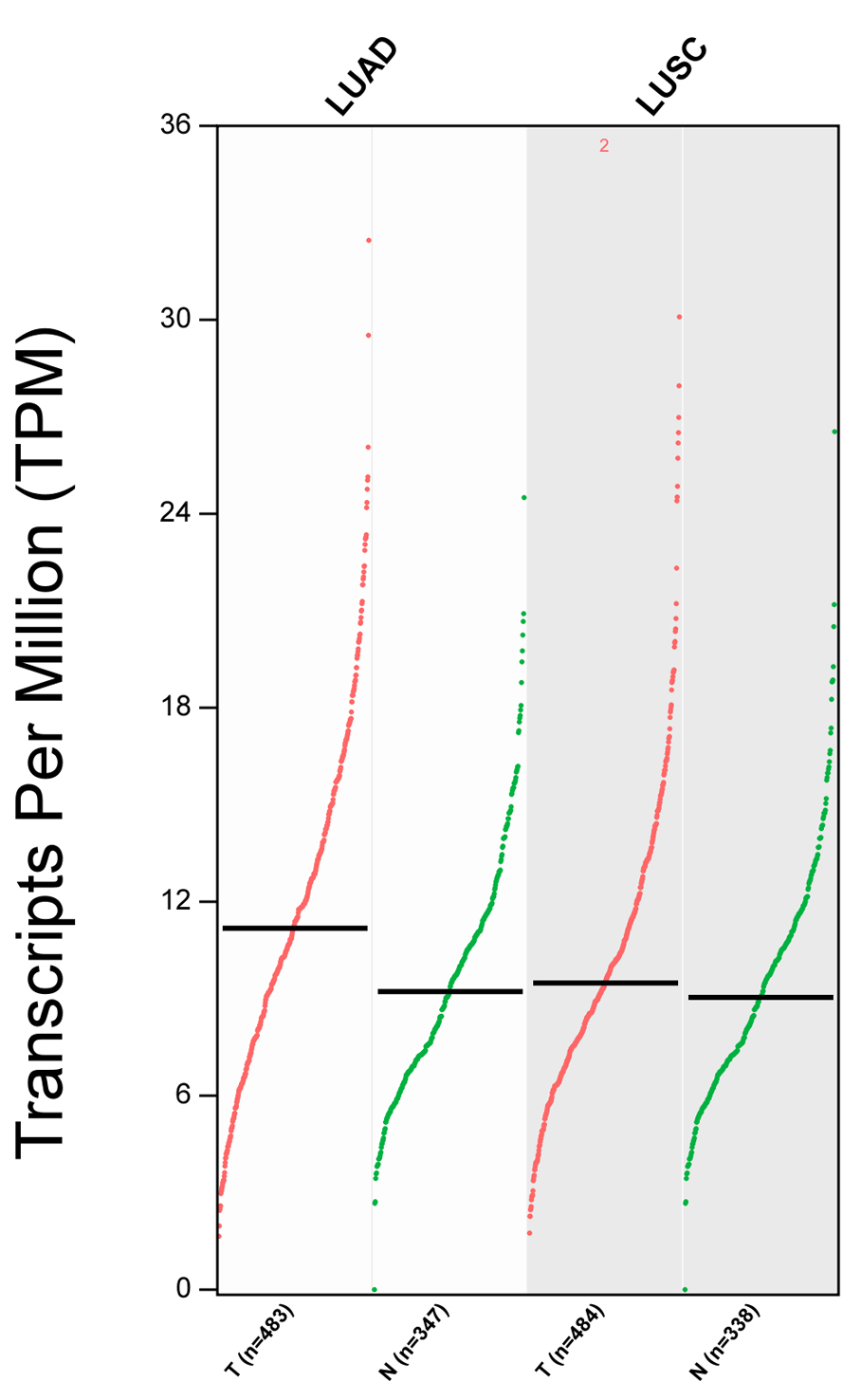

Supplement: Supplementary file 1 [file DataSheet_1.zip › fig 2d. METTL14 DEG.jpg]

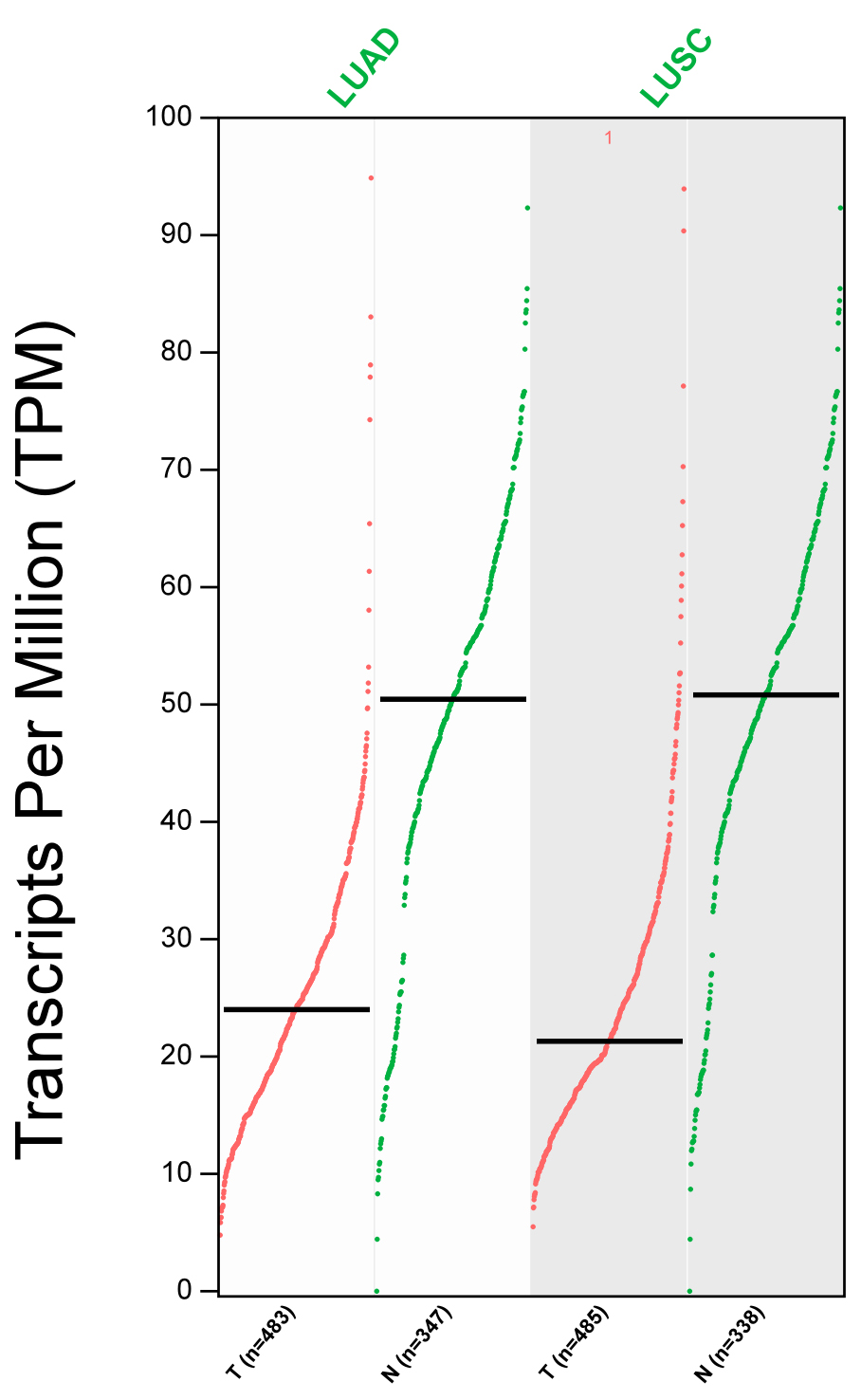

Supplement: Supplementary file 1 [file DataSheet_1.zip › fig 2d. METTL3 DEG.jpg]

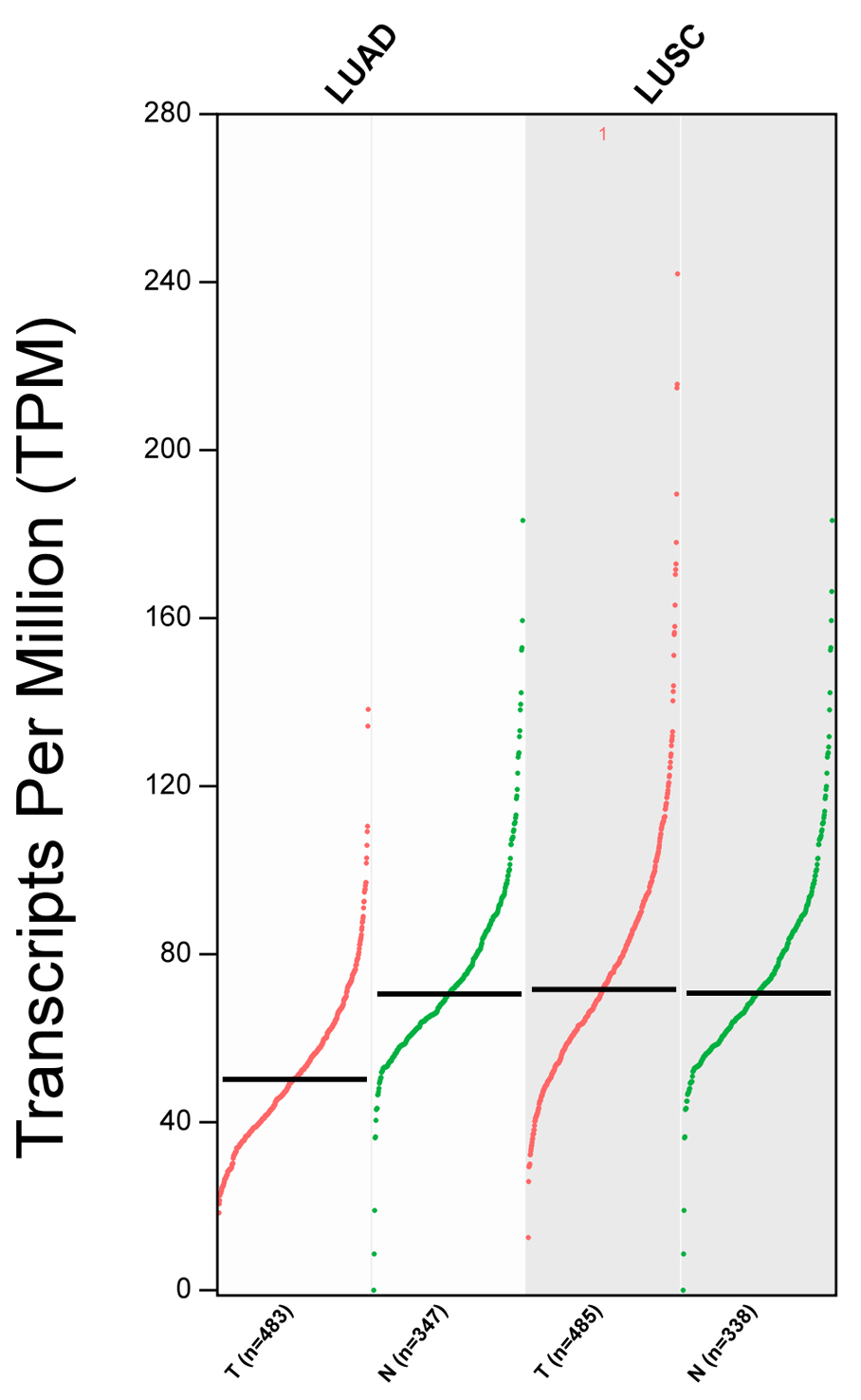

Supplement: Supplementary file 1 [file DataSheet_1.zip › fig 2d. WTAP DEG.jpg]

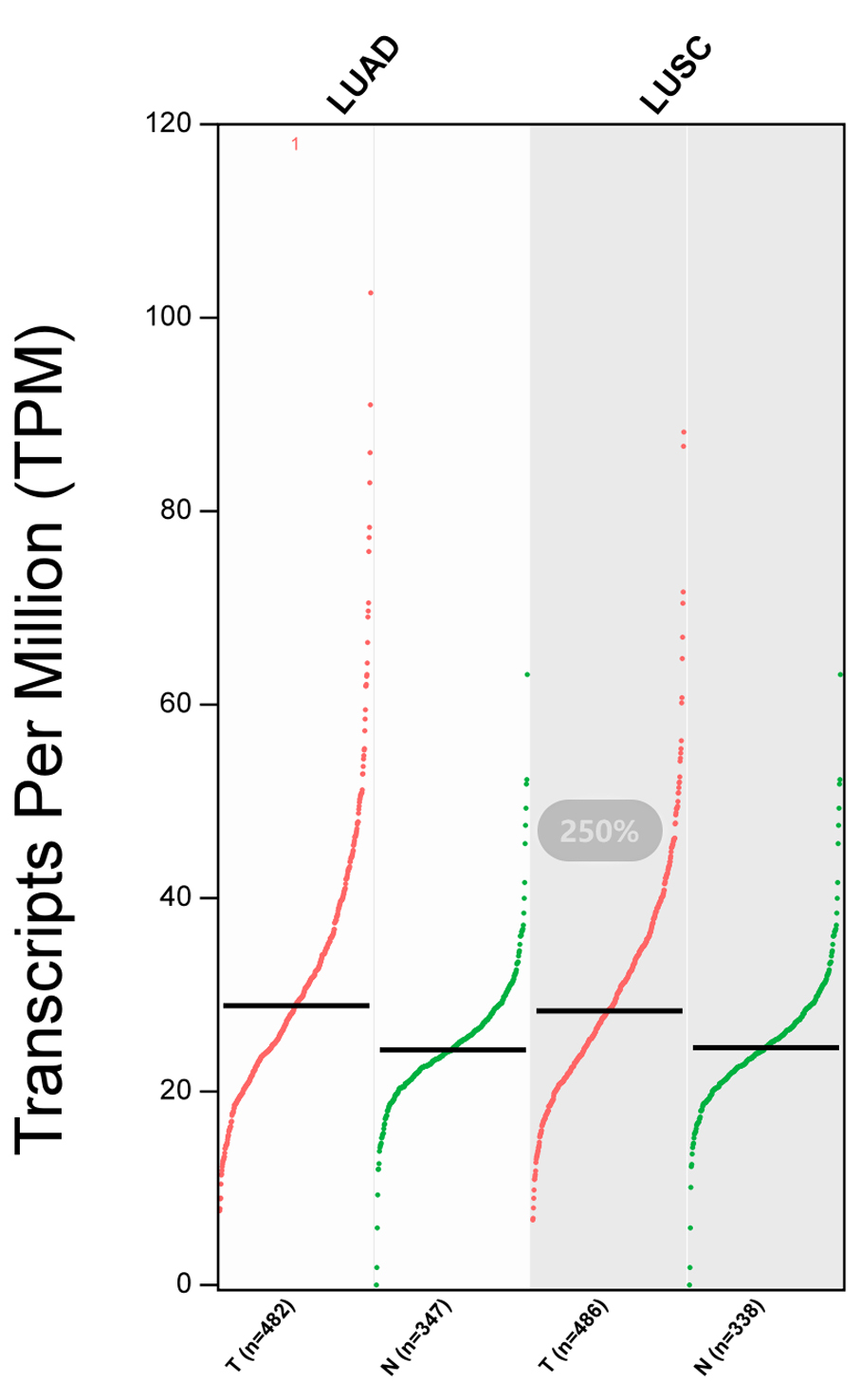

Supplement: Supplementary file 1 [file DataSheet_1.zip › fig 2d. YTHDF1 DEG.jpg]

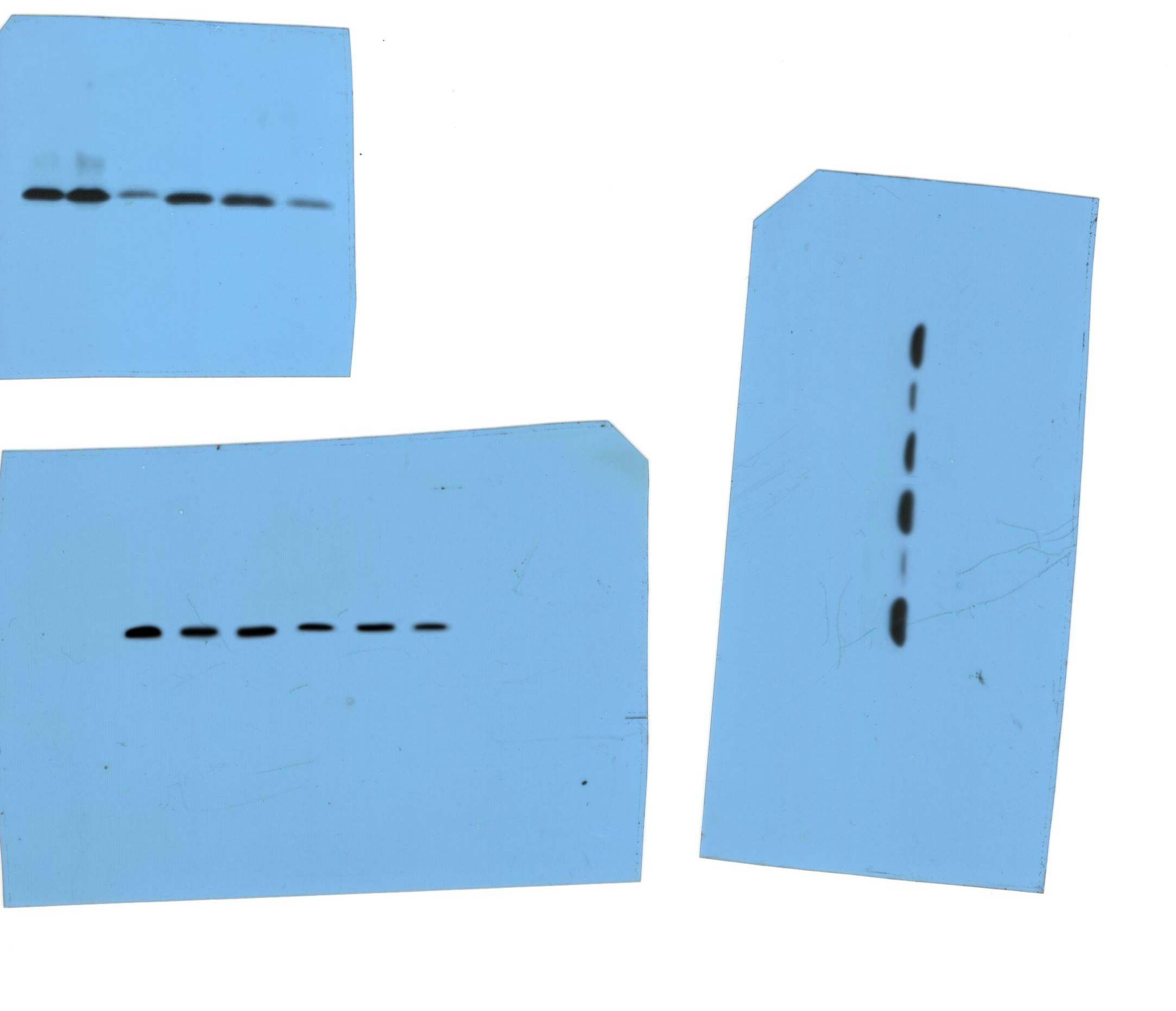

Supplement: Supplementary file 1 [file DataSheet_1.zip › fig 3a. Wb.jpg]

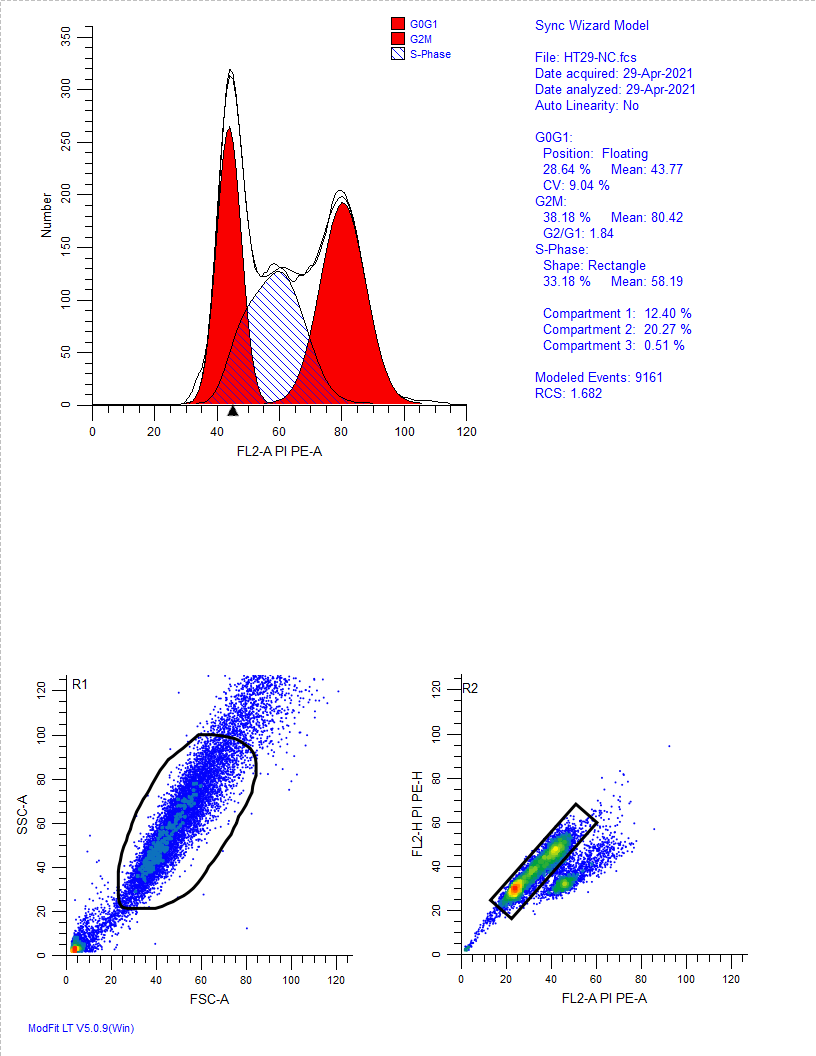

Supplement: Supplementary file 1 [file DataSheet_1.zip › fig 3d. A549 CSCs (1).tif]

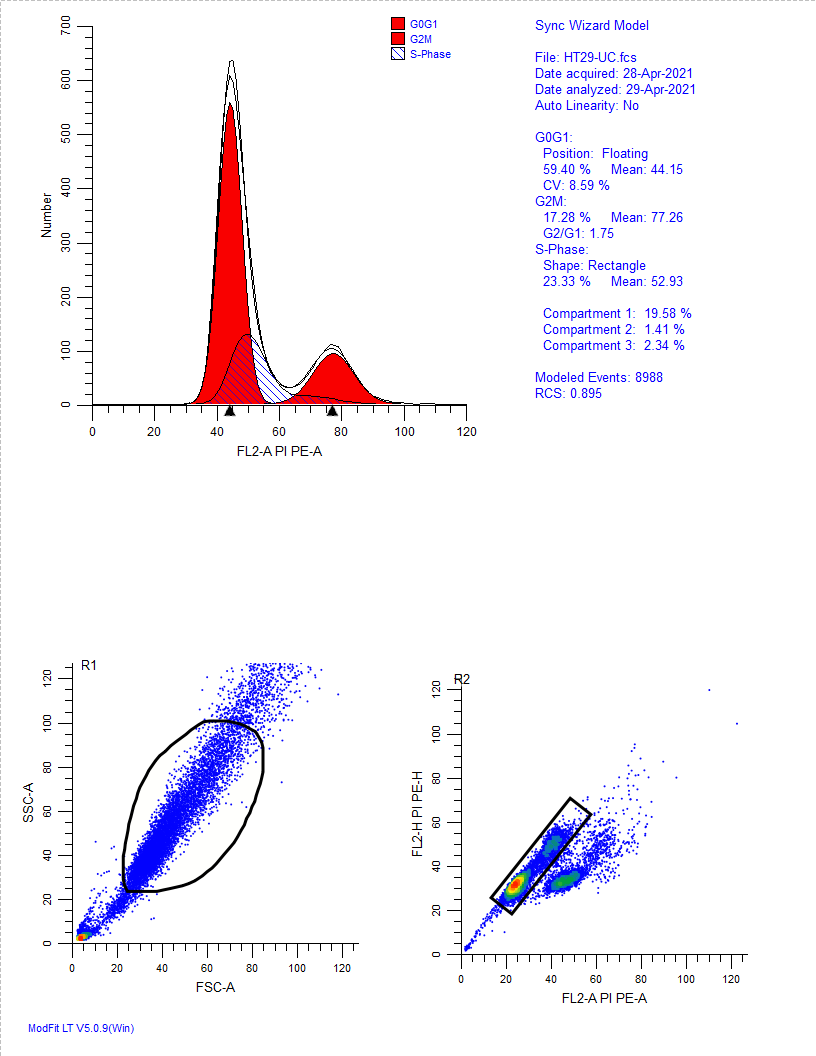

Supplement: Supplementary file 1 [file DataSheet_1.zip › fig 3d. A549 CSCs (2).tif]

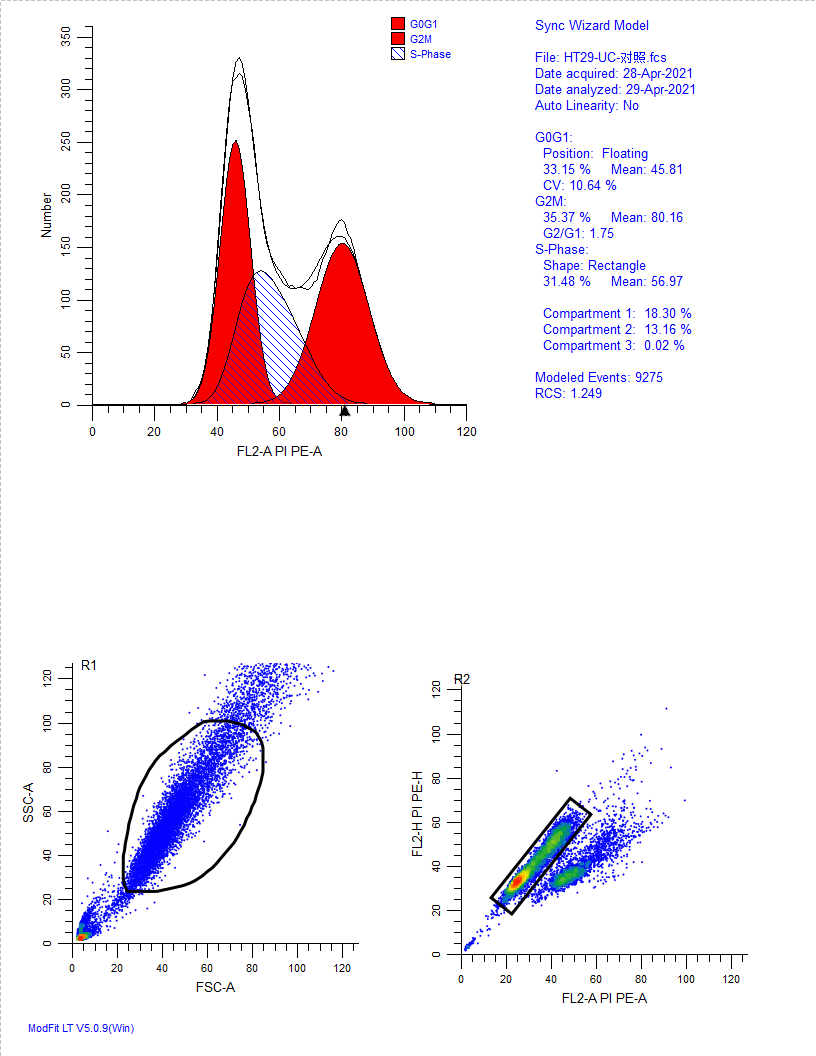

Supplement: Supplementary file 1 [file DataSheet_1.zip › fig 3d. A549 CSCs (3).tif]

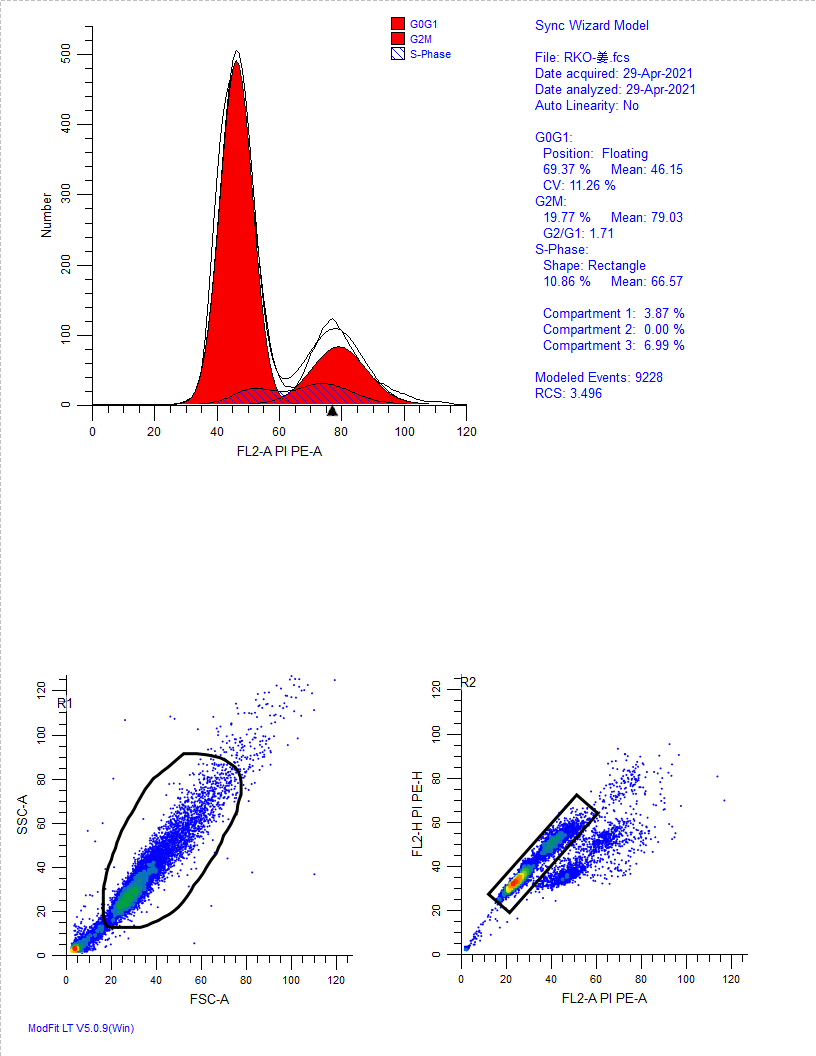

Supplement: Supplementary file 1 [file DataSheet_1.zip › fig 3d. PC-9 CSCs (1).tif]

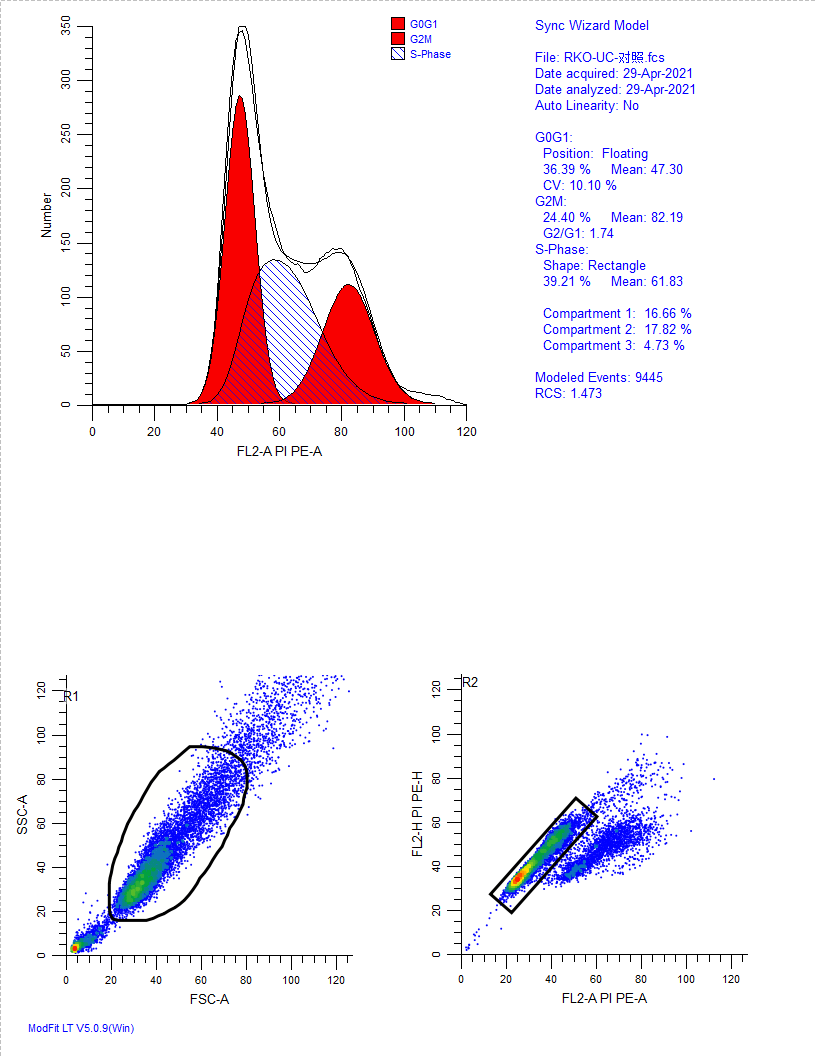

Supplement: Supplementary file 1 [file DataSheet_1.zip › fig 3d. PC-9 CSCs (2).tif]

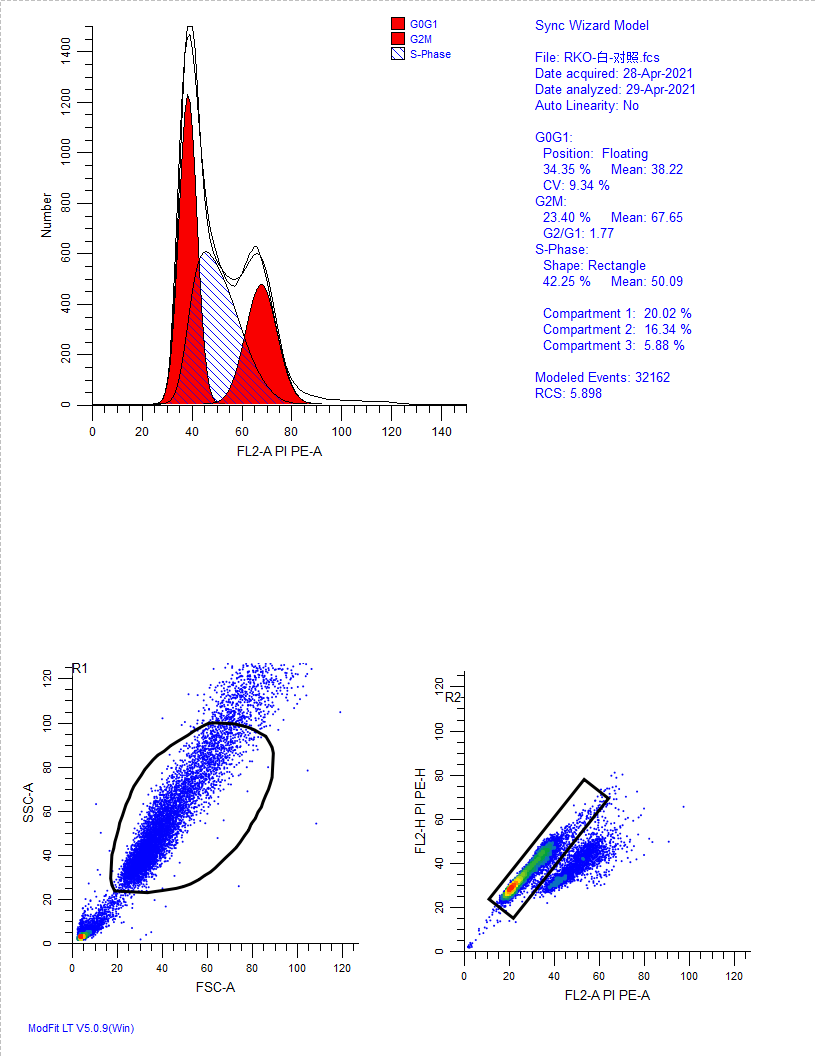

Supplement: Supplementary file 1 [file DataSheet_1.zip › fig 3d. PC-9 CSCs (3).tif]

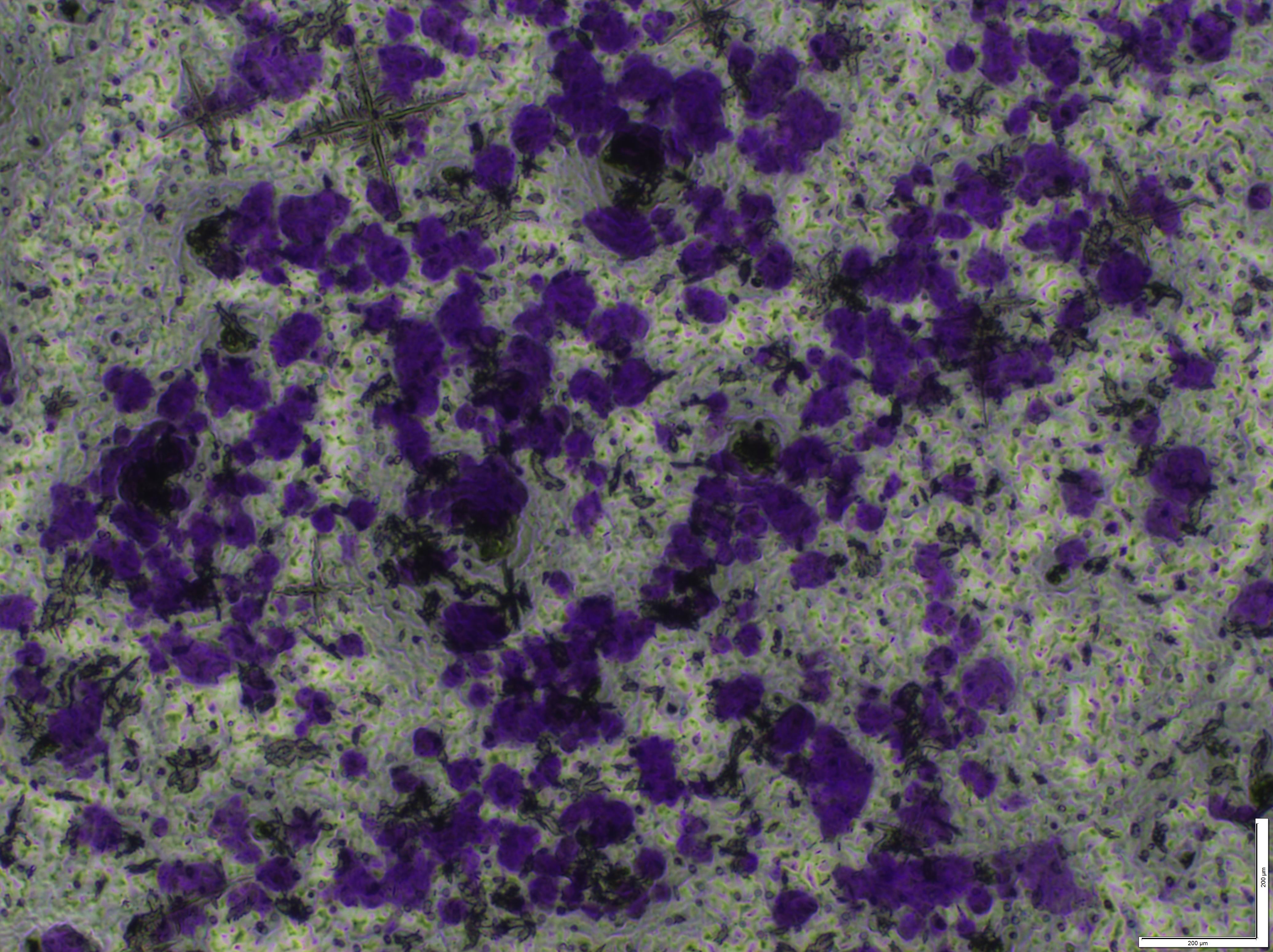

Supplement: Supplementary file 2 [file DataSheet_2.zip › fig 3e. A549 CSCs (1).jpg]

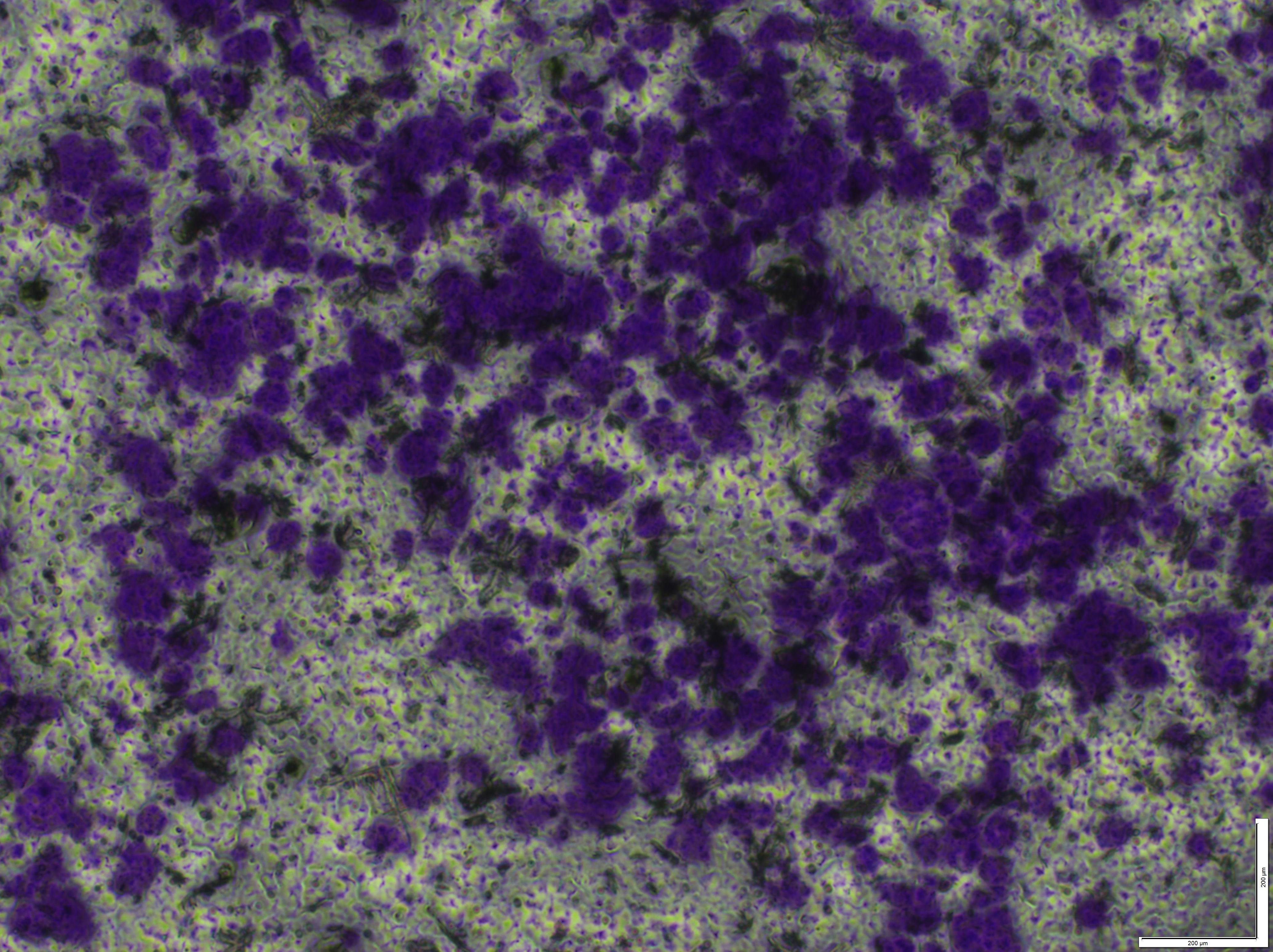

Supplement: Supplementary file 2 [file DataSheet_2.zip › fig 3e. A549 CSCs (2).jpg]

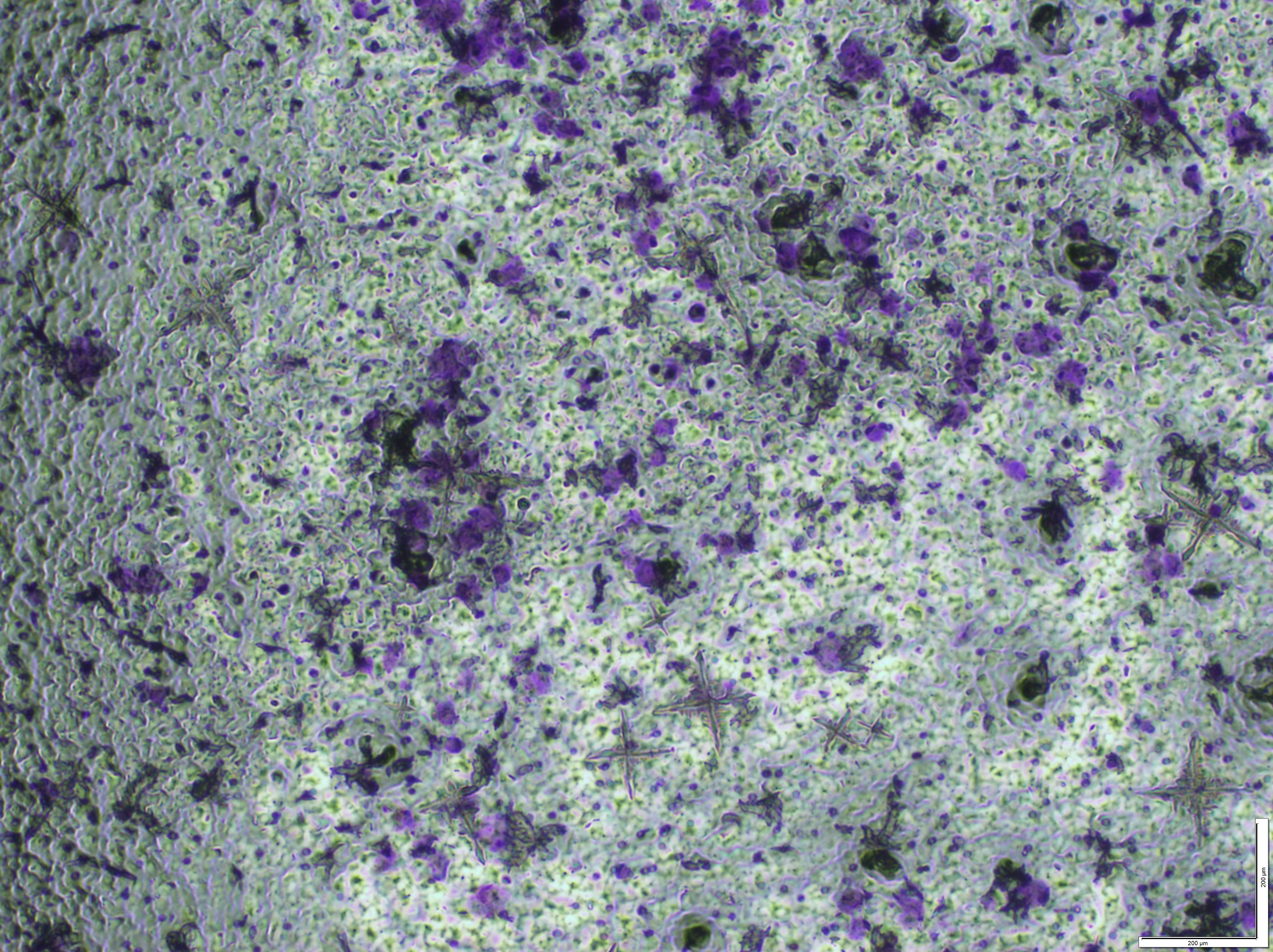

Supplement: Supplementary file 2 [file DataSheet_2.zip › fig 3e. A549 CSCs (3).jpg]

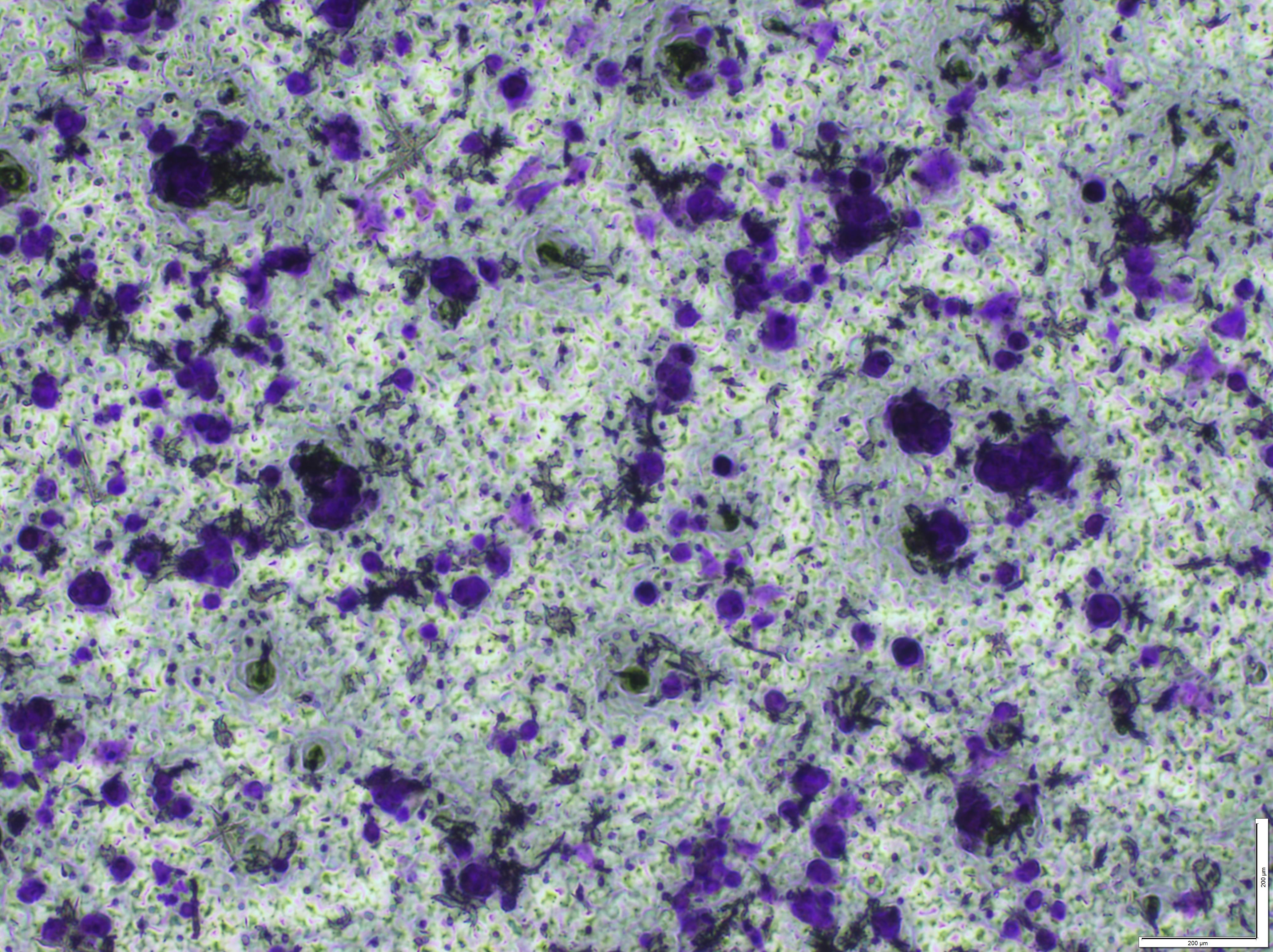

Supplement: Supplementary file 2 [file DataSheet_2.zip › fig 3e. PC-9 (1).jpg]

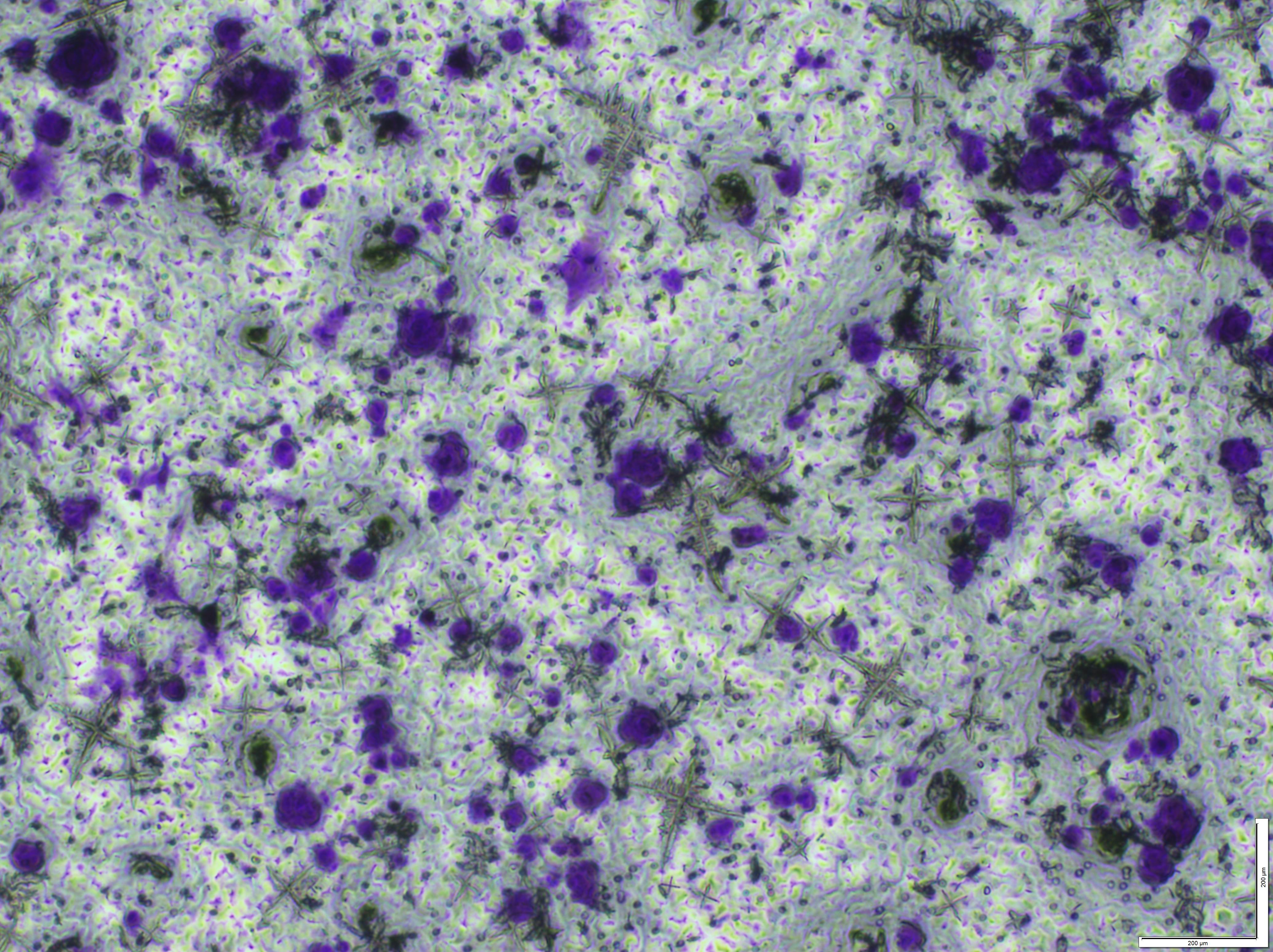

Supplement: Supplementary file 2 [file DataSheet_2.zip › fig 3e. PC-9 (2).jpg]

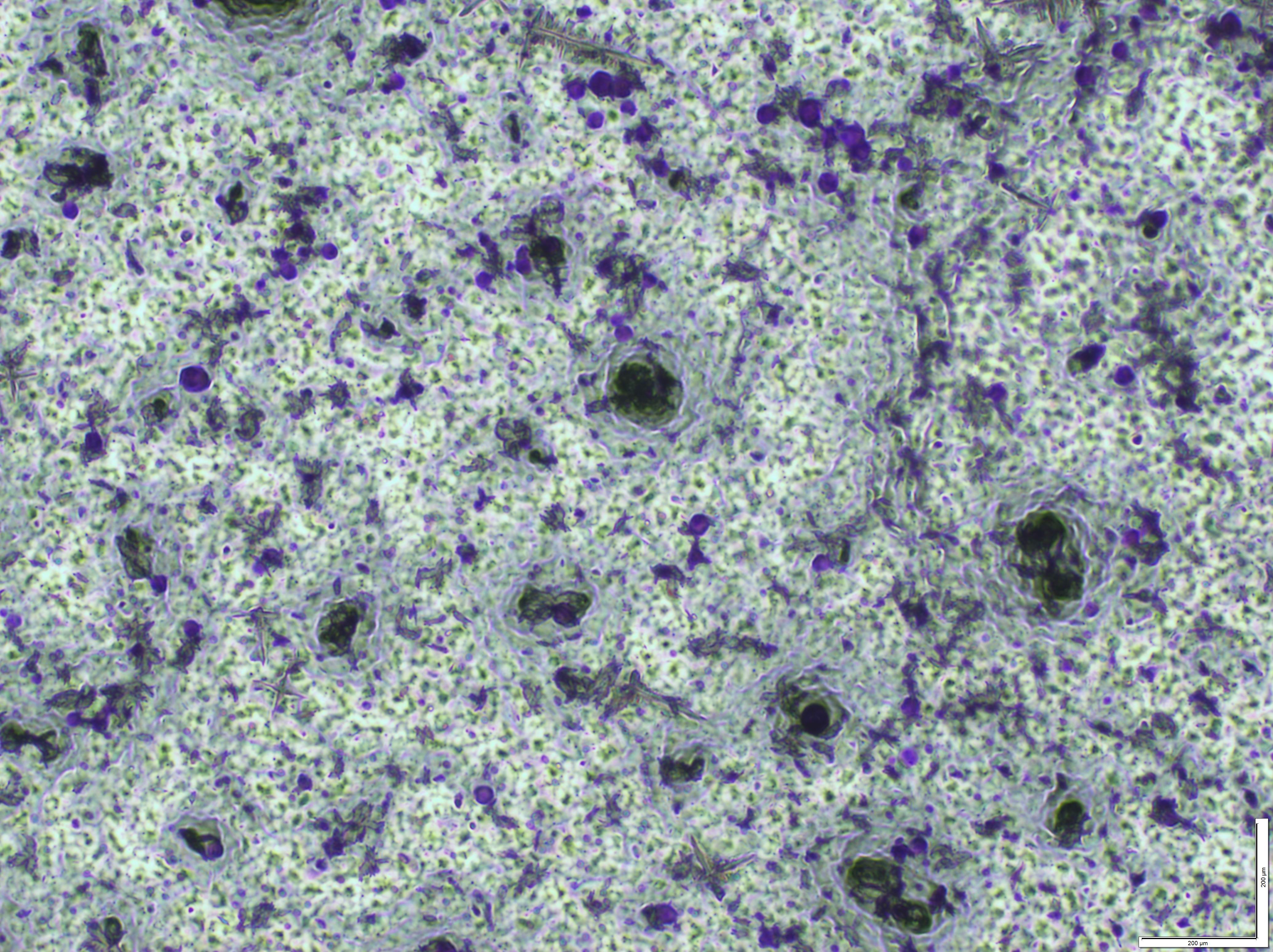

Supplement: Supplementary file 2 [file DataSheet_2.zip › fig 3e. PC-9 (3).jpg]

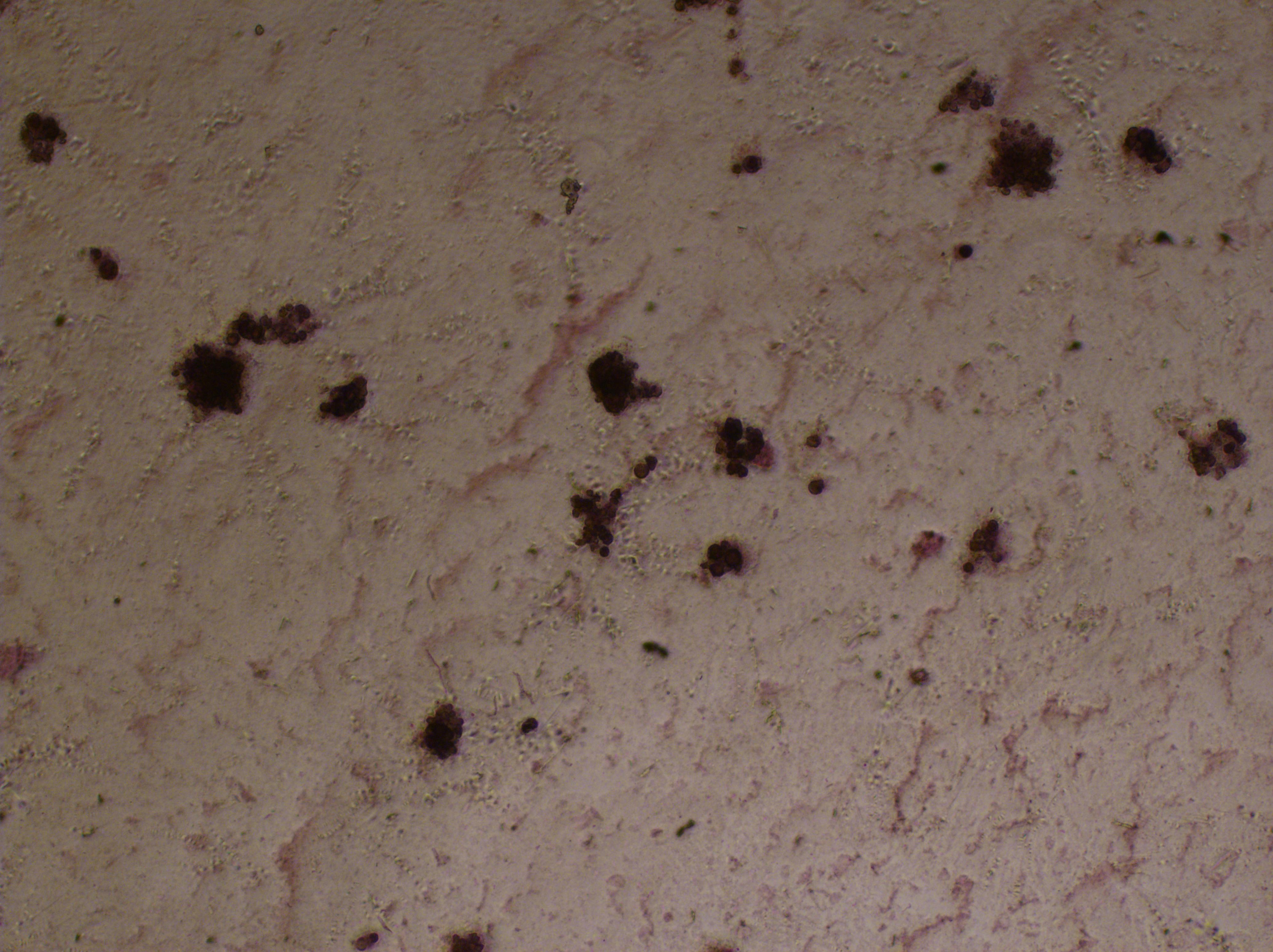

Supplement: Supplementary file 2 [file DataSheet_2.zip › fig 3f. A549 CSCs (1).jpg]

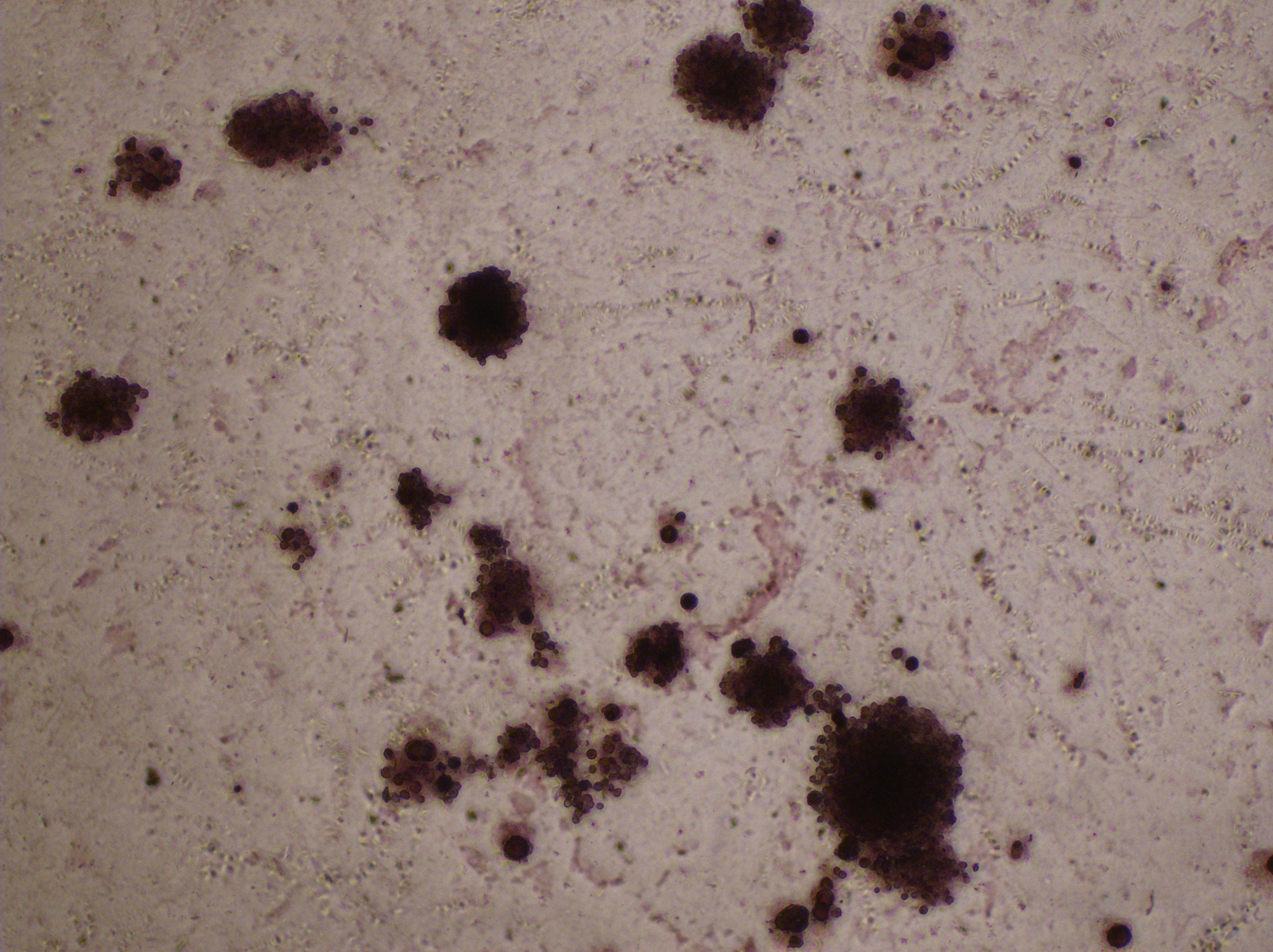

Supplement: Supplementary file 2 [file DataSheet_2.zip › fig 3f. A549 CSCs (2).jpg]

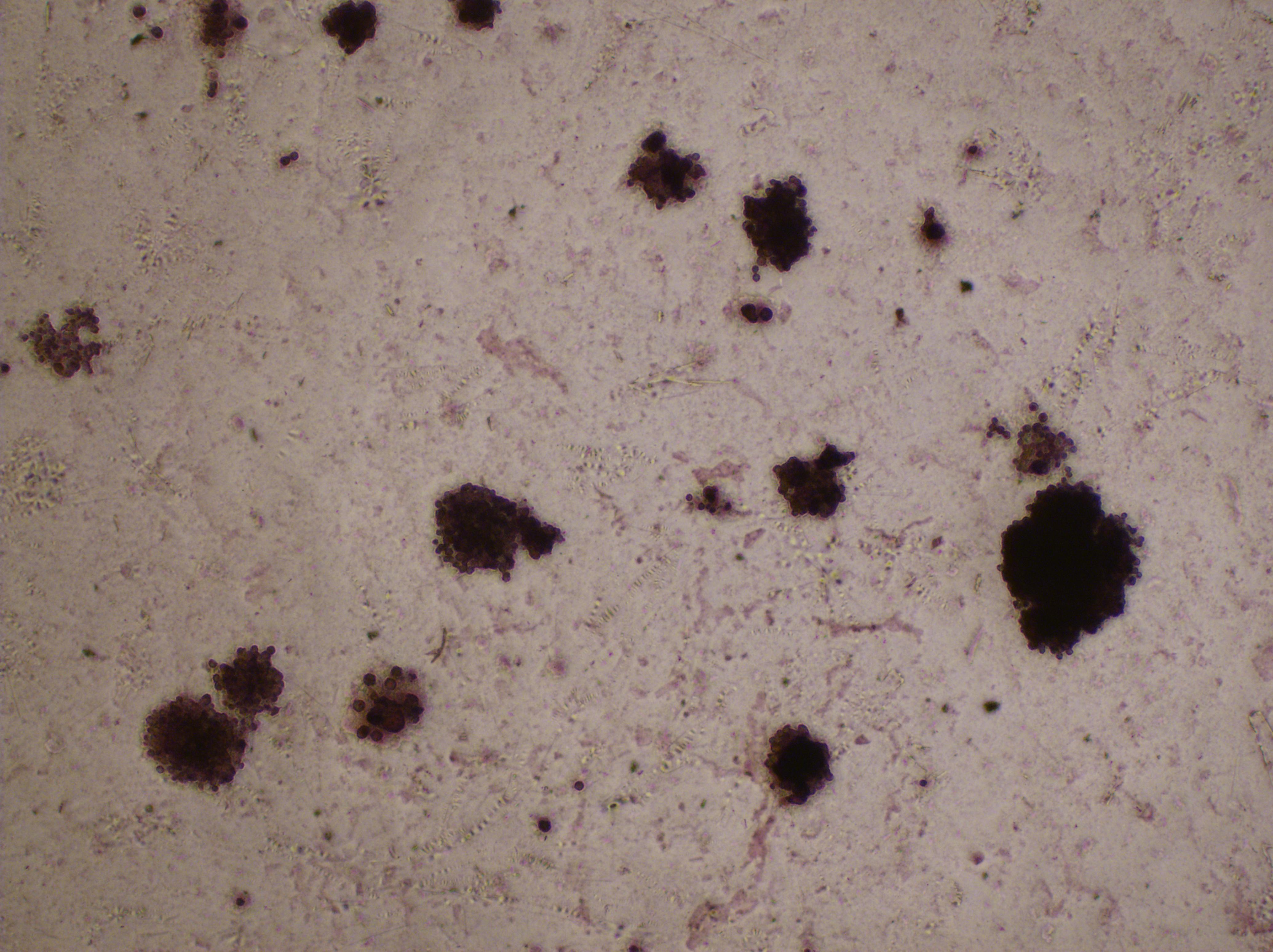

Supplement: Supplementary file 2 [file DataSheet_2.zip › fig 3f. A549 CSCs (3).jpg]

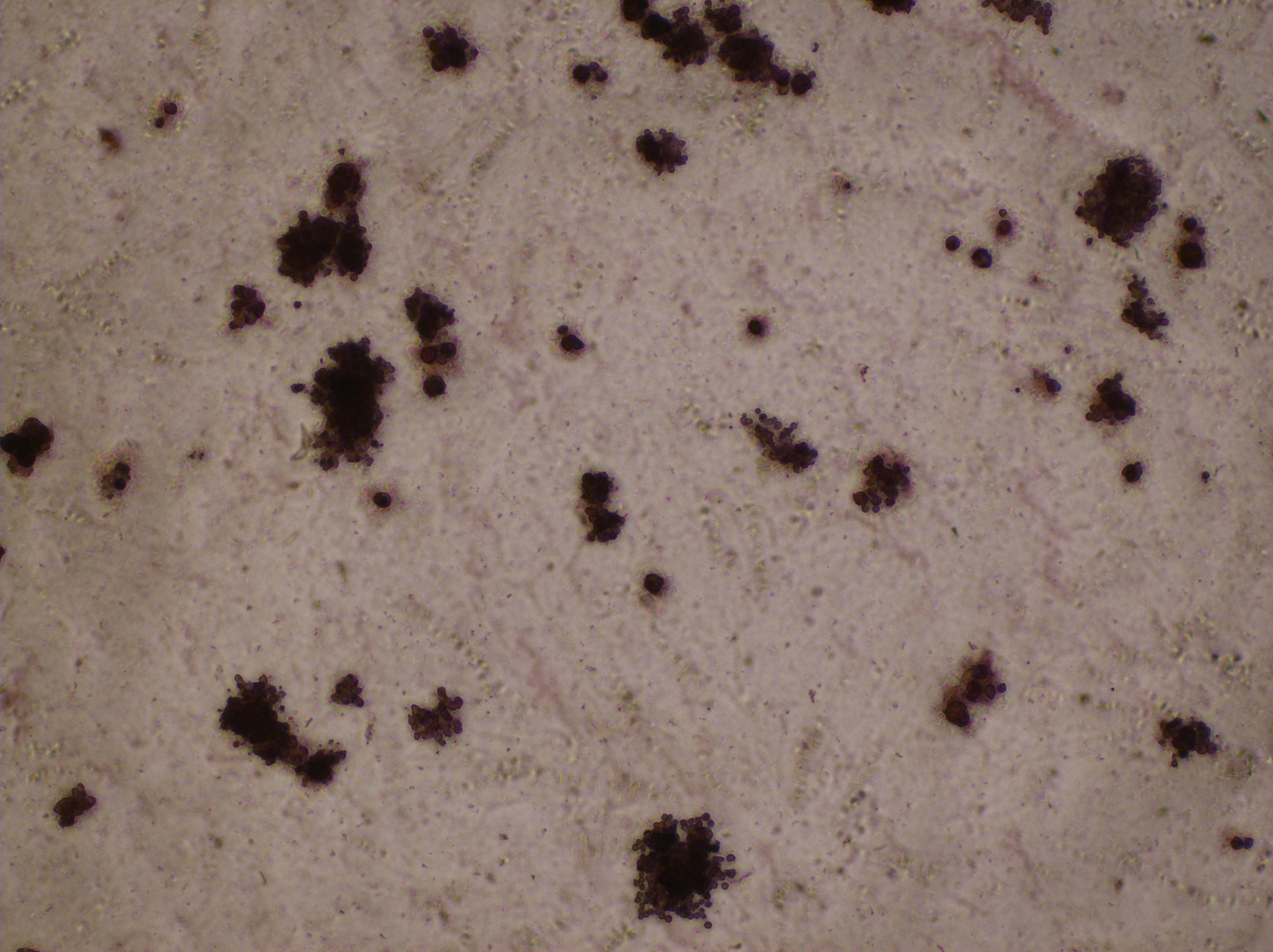

Supplement: Supplementary file 2 [file DataSheet_2.zip › fig 3f. PC-9 (1).jpg]

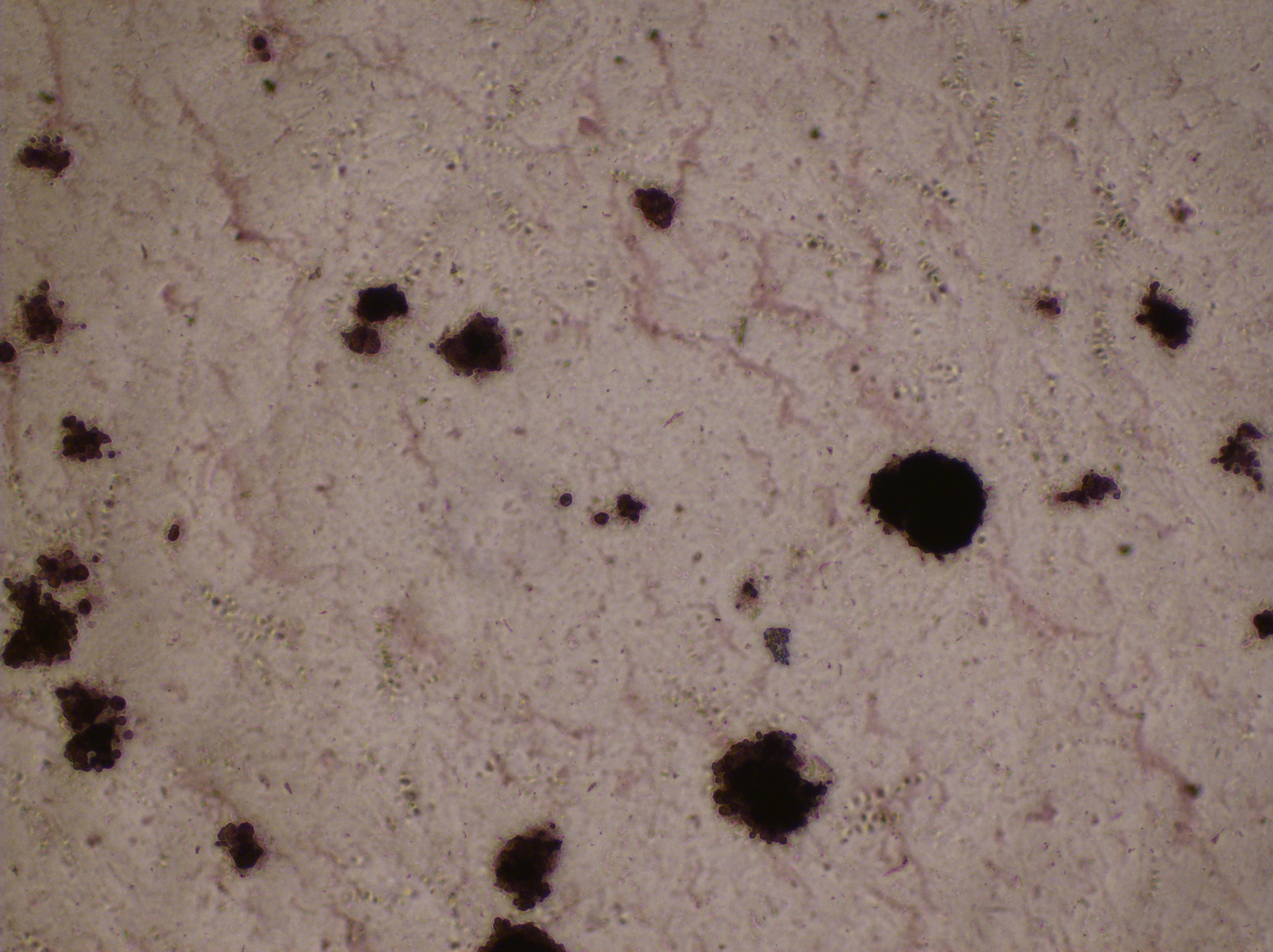

Supplement: Supplementary file 2 [file DataSheet_2.zip › fig 3f. PC-9 (2).jpg]

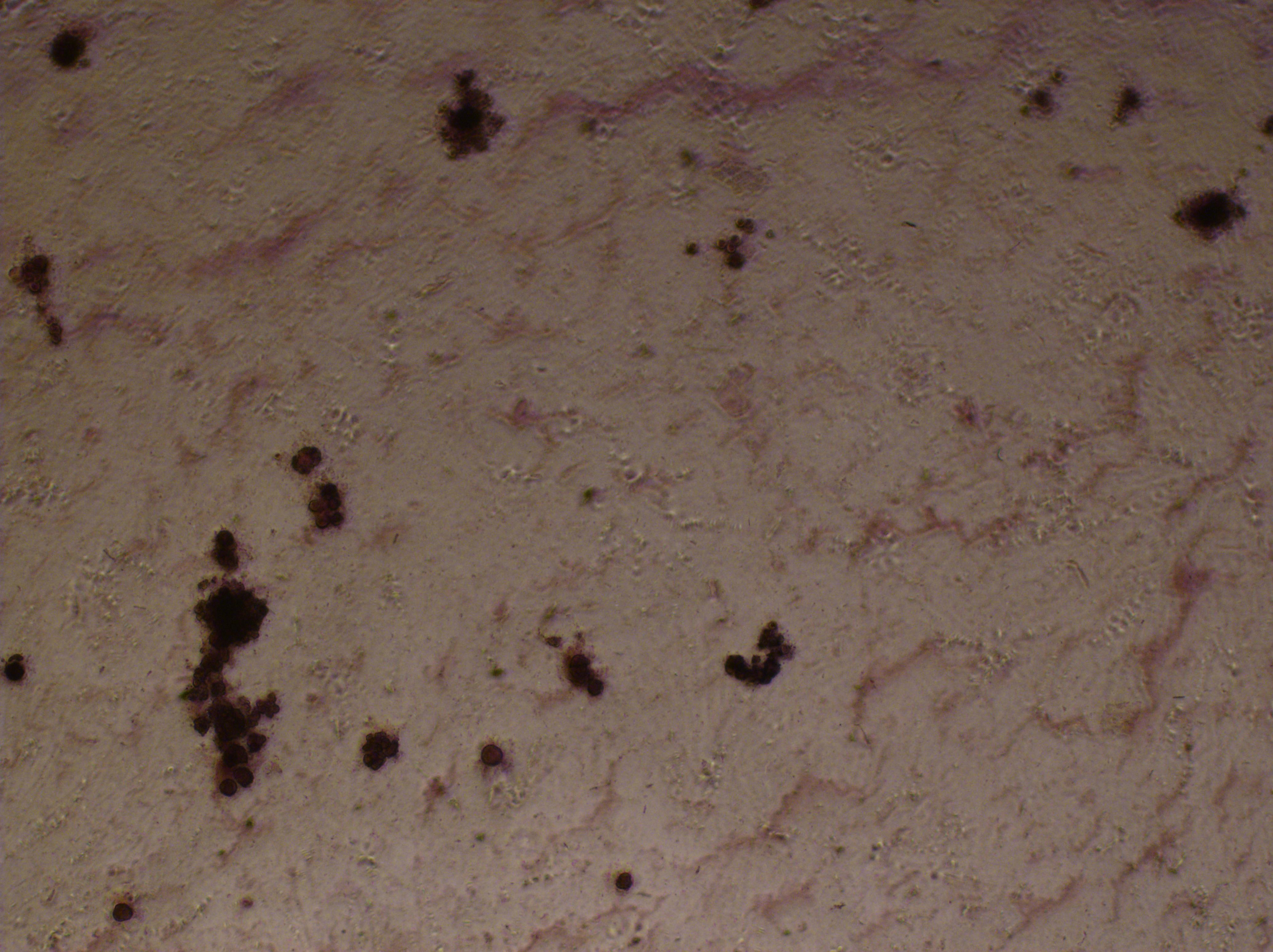

Supplement: Supplementary file 2 [file DataSheet_2.zip › fig 3f. PC-9 (3).jpg]

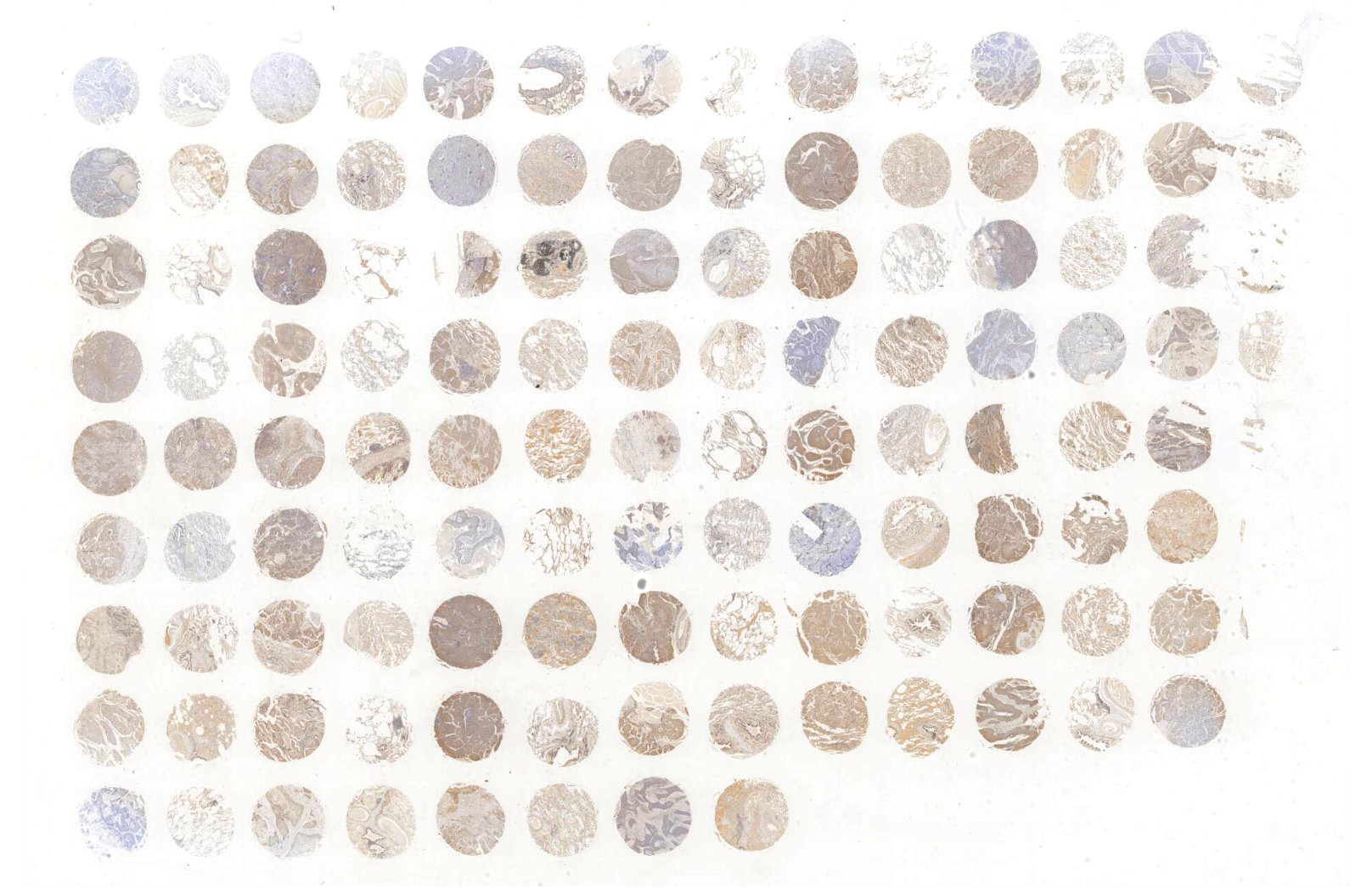

Supplement: Supplementary file 2 [file DataSheet_2.zip › fig 4a. ╖╬┴█░⌐ ALKBH5.jpg]

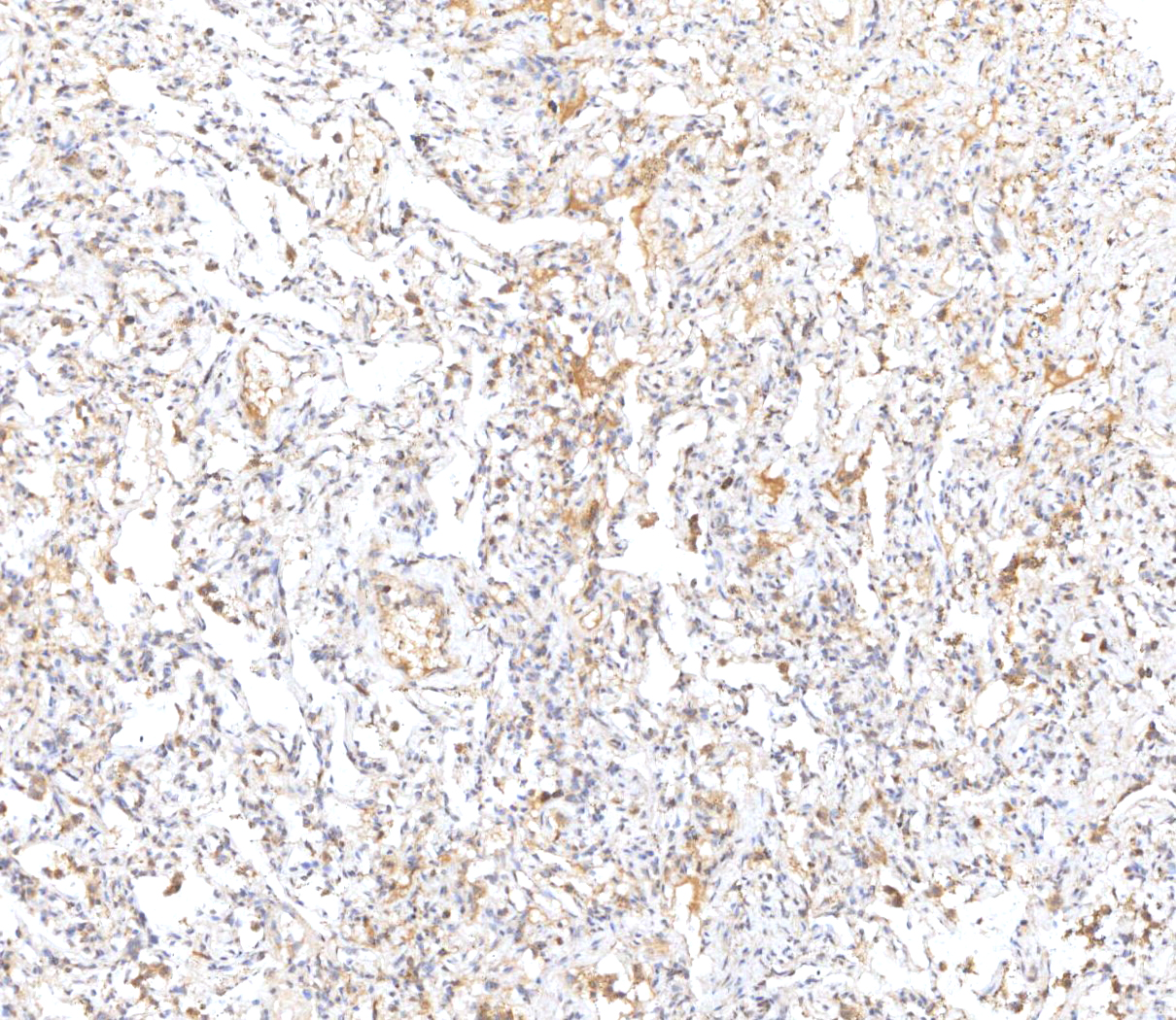

Supplement: Supplementary file 2 [file DataSheet_2.zip › fig 4a. ╖╬┴█░⌐ II, III╞┌ ALKBH5 12N 10í┴.jpg]

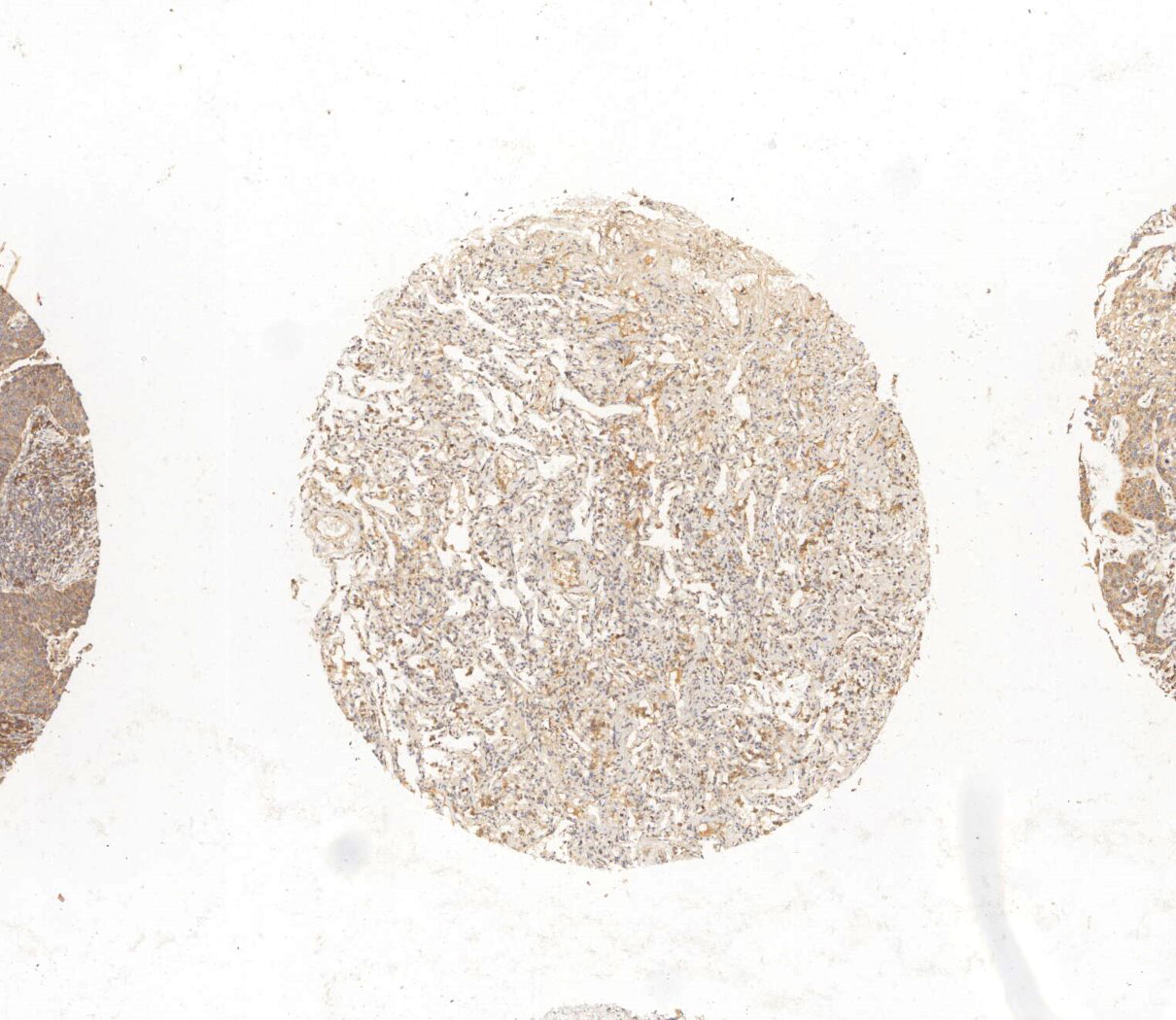

Supplement: Supplementary file 2 [file DataSheet_2.zip › fig 4a. ╖╬┴█░⌐ II, III╞┌ ALKBH5 12N 4í┴.jpg]

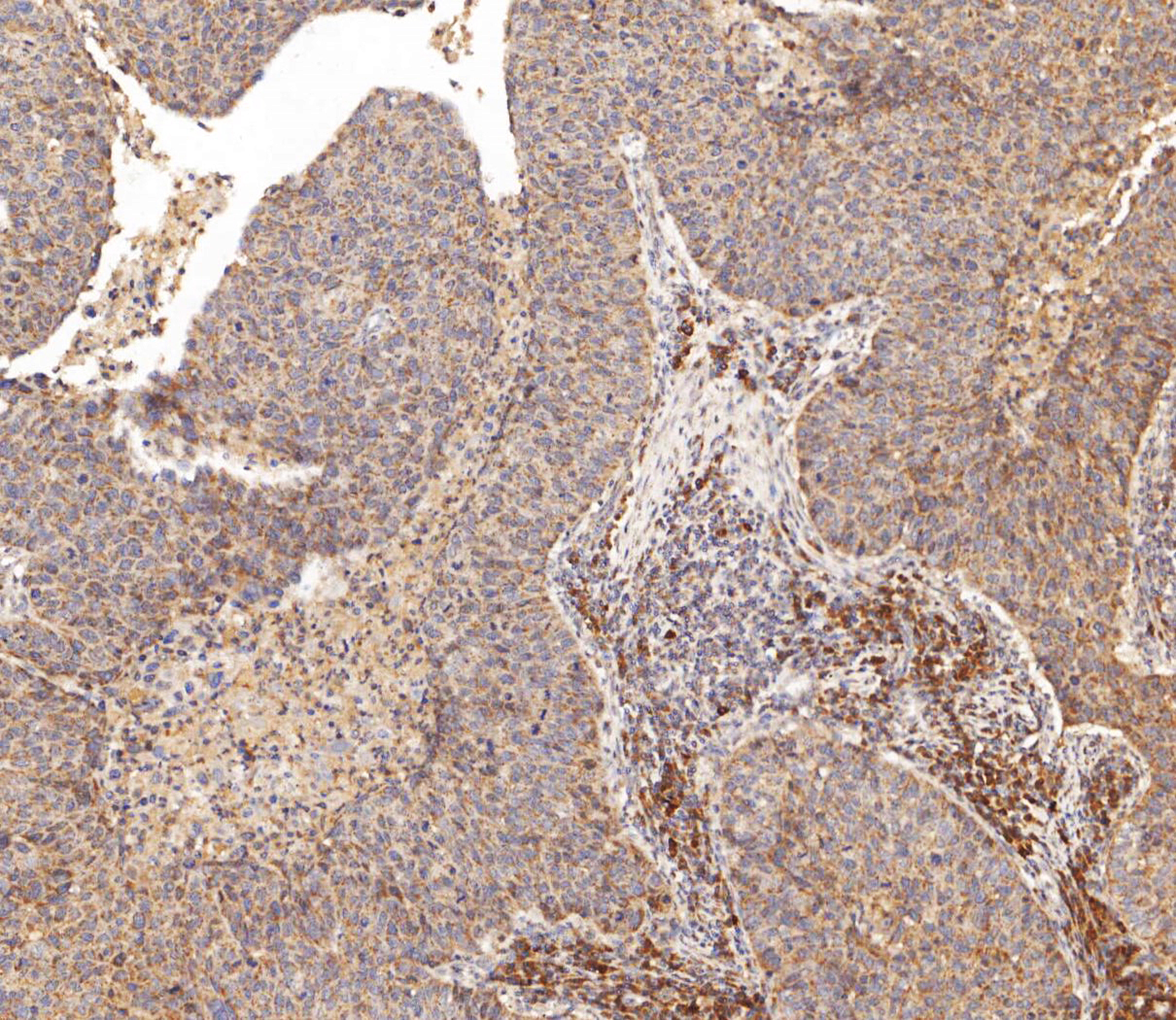

Supplement: Supplementary file 2 [file DataSheet_2.zip › fig 4a. ╖╬┴█░⌐ II, III╞┌ ALKBH5 12T 10í┴.jpg]

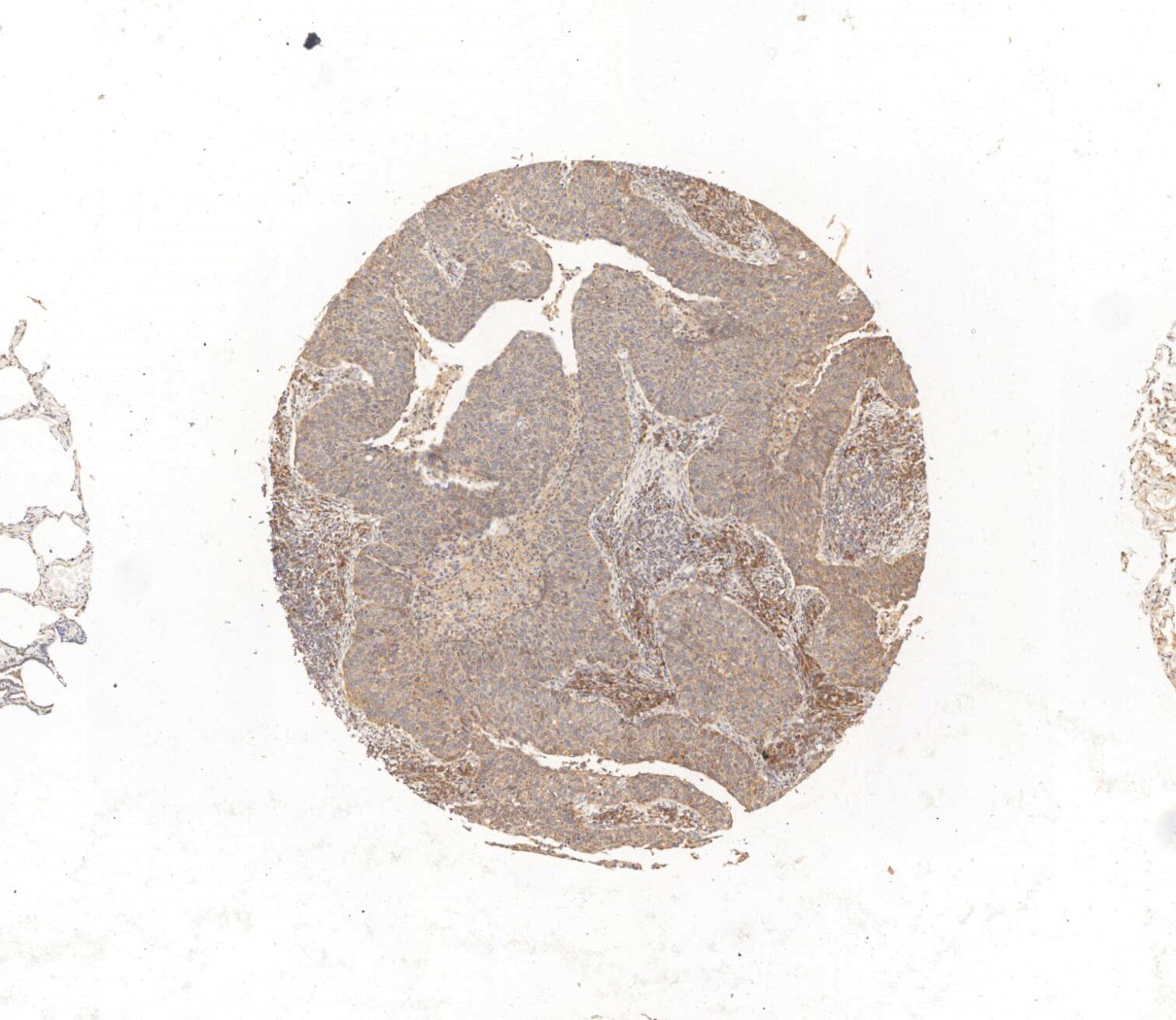

Supplement: Supplementary file 2 [file DataSheet_2.zip › fig 4a. ╖╬┴█░⌐ II, III╞┌ ALKBH5 12T 4í┴.jpg]

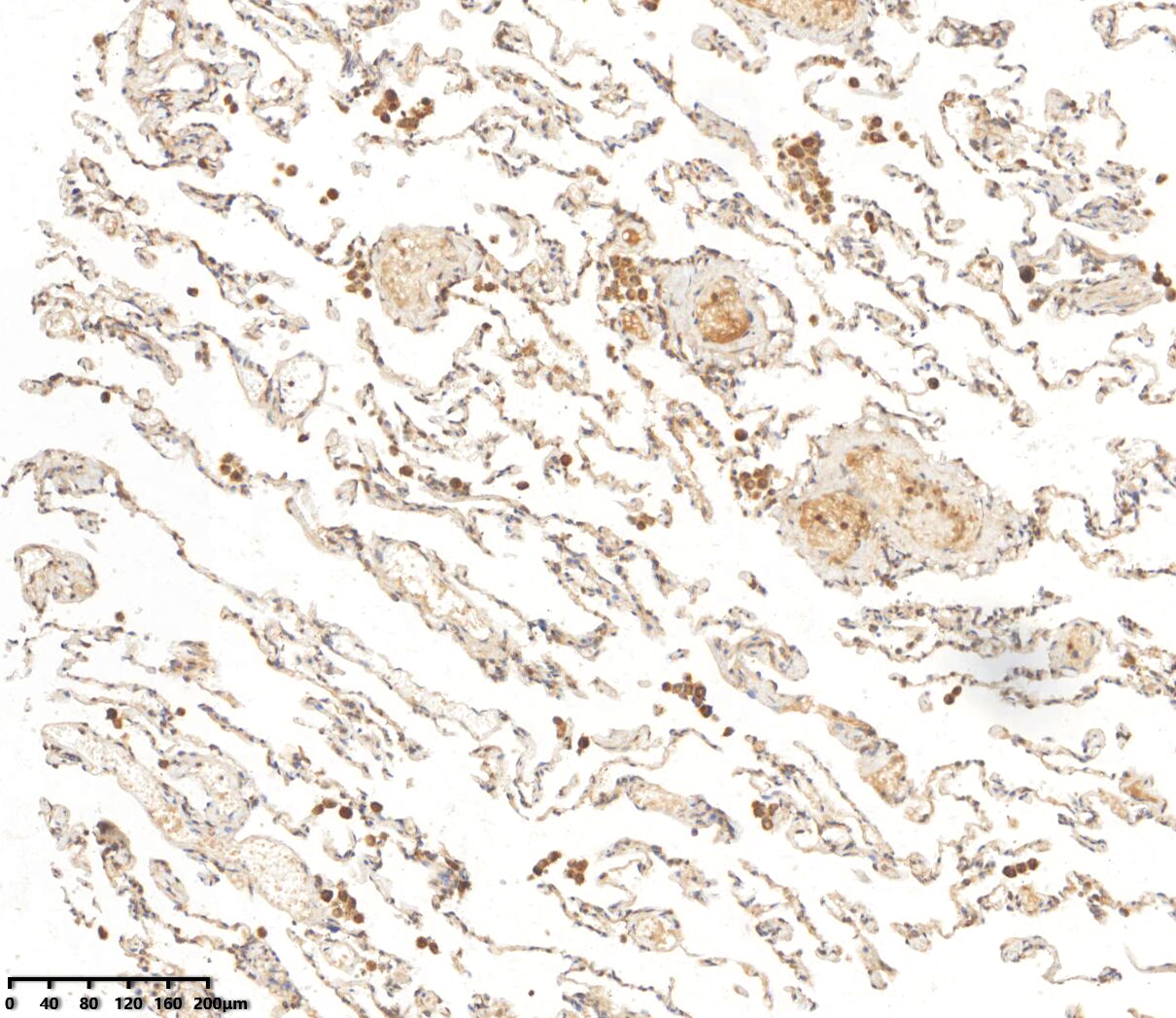

Supplement: Supplementary file 2 [file DataSheet_2.zip › fig 4a. ╖╬┴█░⌐ II╞┌ ALKBH5 20N 10í┴.jpg]

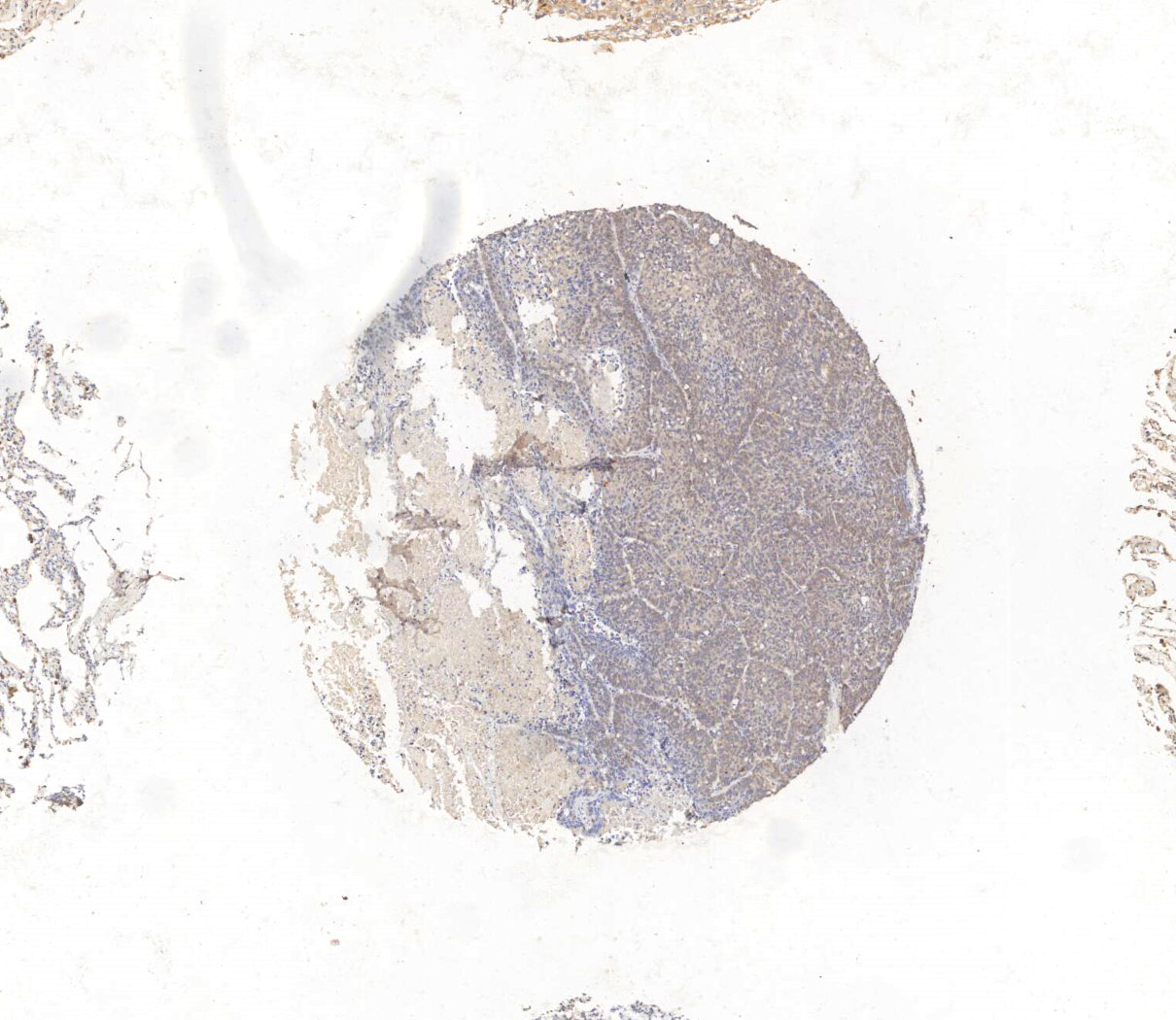

Supplement: Supplementary file 2 [file DataSheet_2.zip › fig 4a. ╖╬┴█░⌐ II╞┌ ALKBH5 20N 4í┴.jpg]

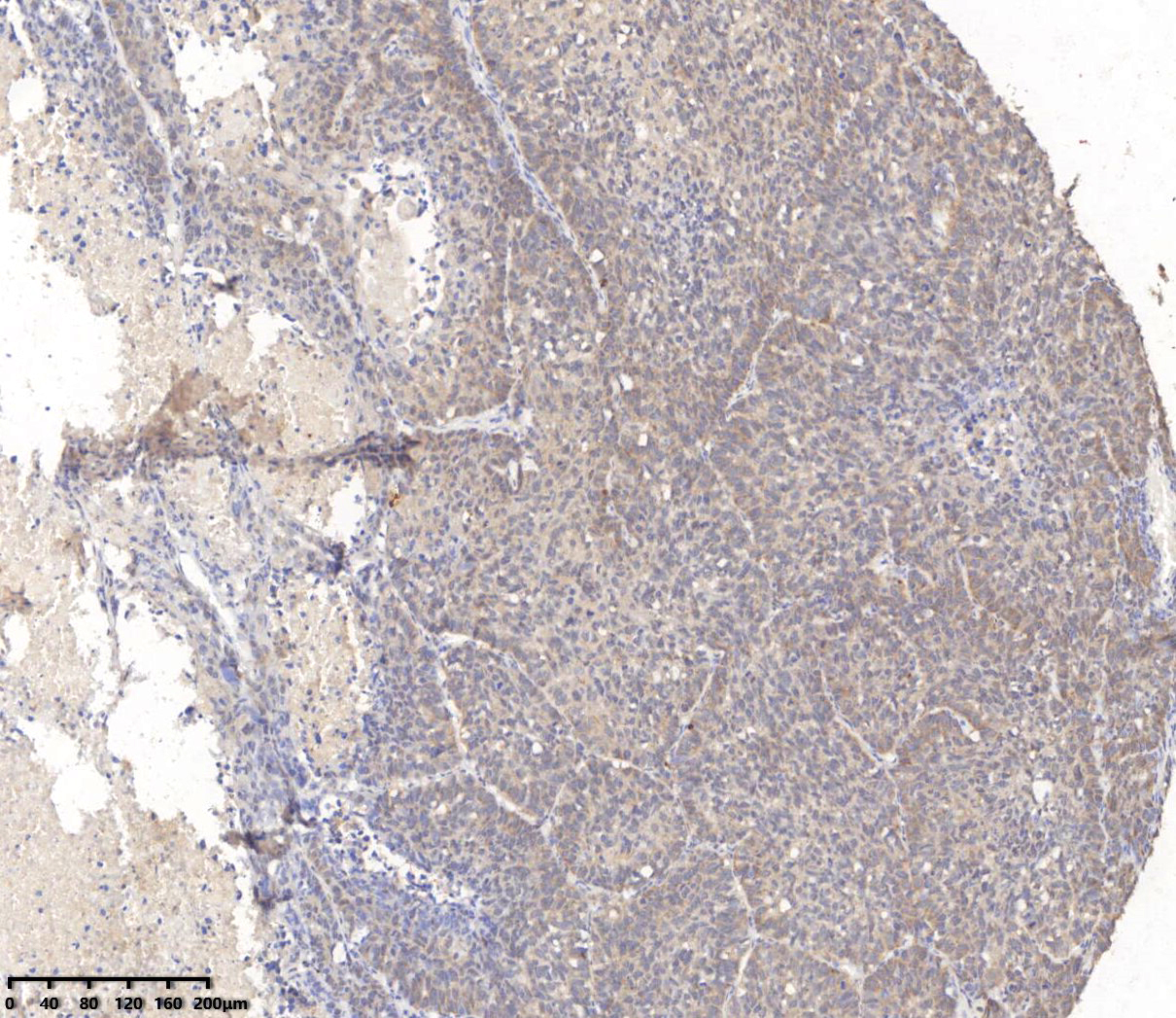

Supplement: Supplementary file 2 [file DataSheet_2.zip › fig 4a. ╖╬┴█░⌐ II╞┌ ALKBH5 20T 10í┴.jpg]

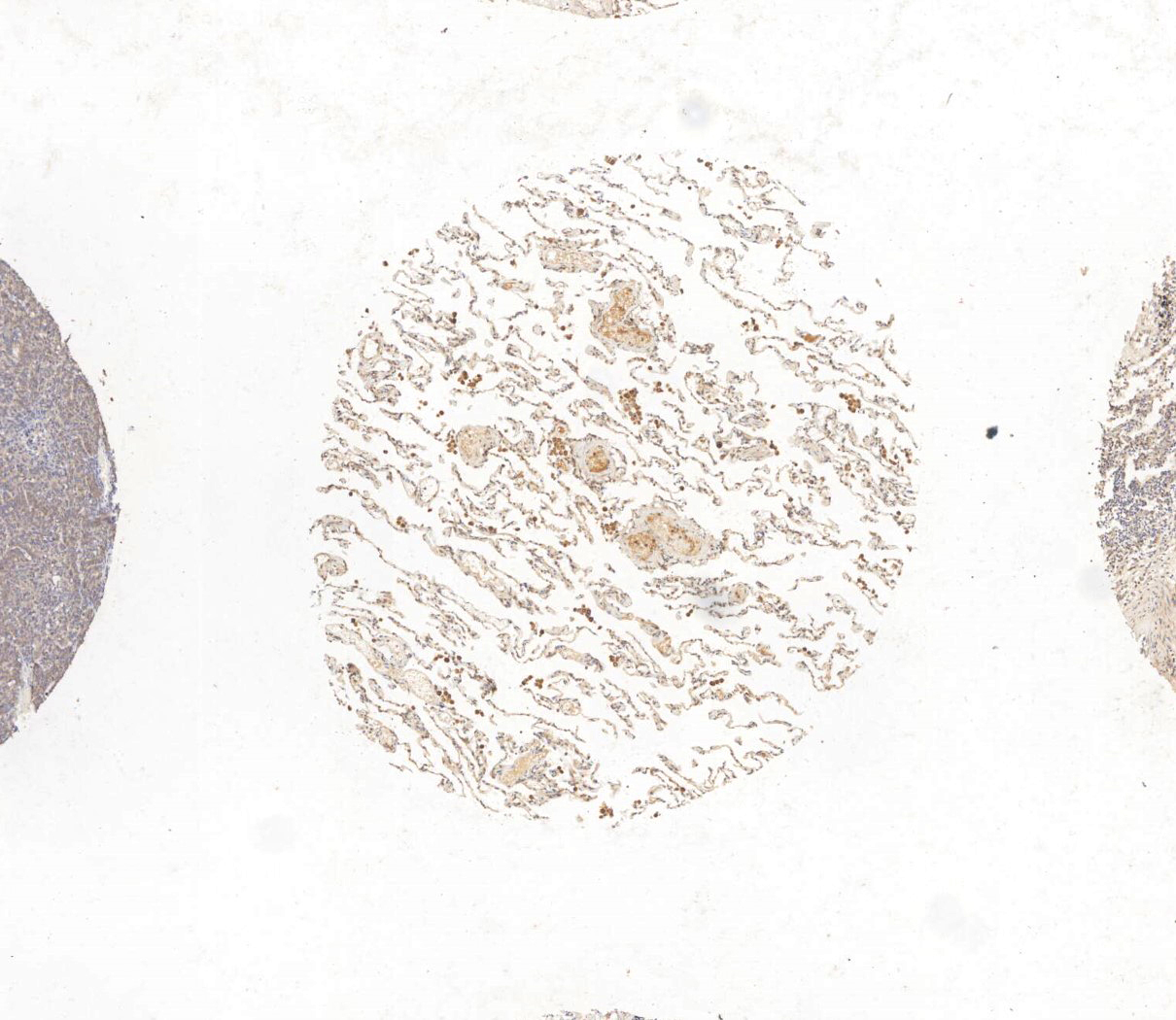

Supplement: Supplementary file 2 [file DataSheet_2.zip › fig 4a. ╖╬┴█░⌐ II╞┌ ALKBH5 20T 4í┴.jpg]

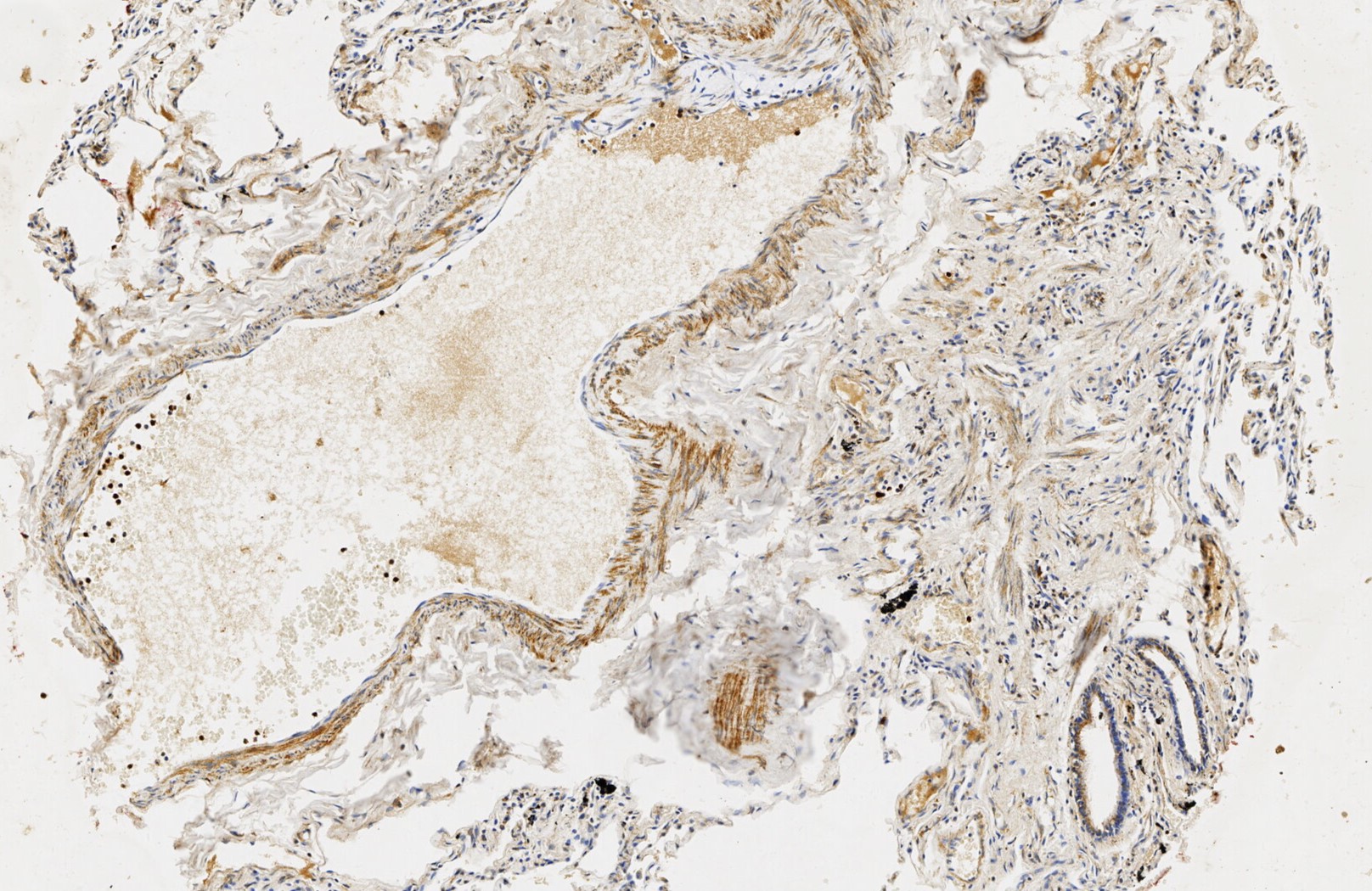

Supplement: Supplementary file 2 [file DataSheet_2.zip › fig 4b. 21N.jpg]

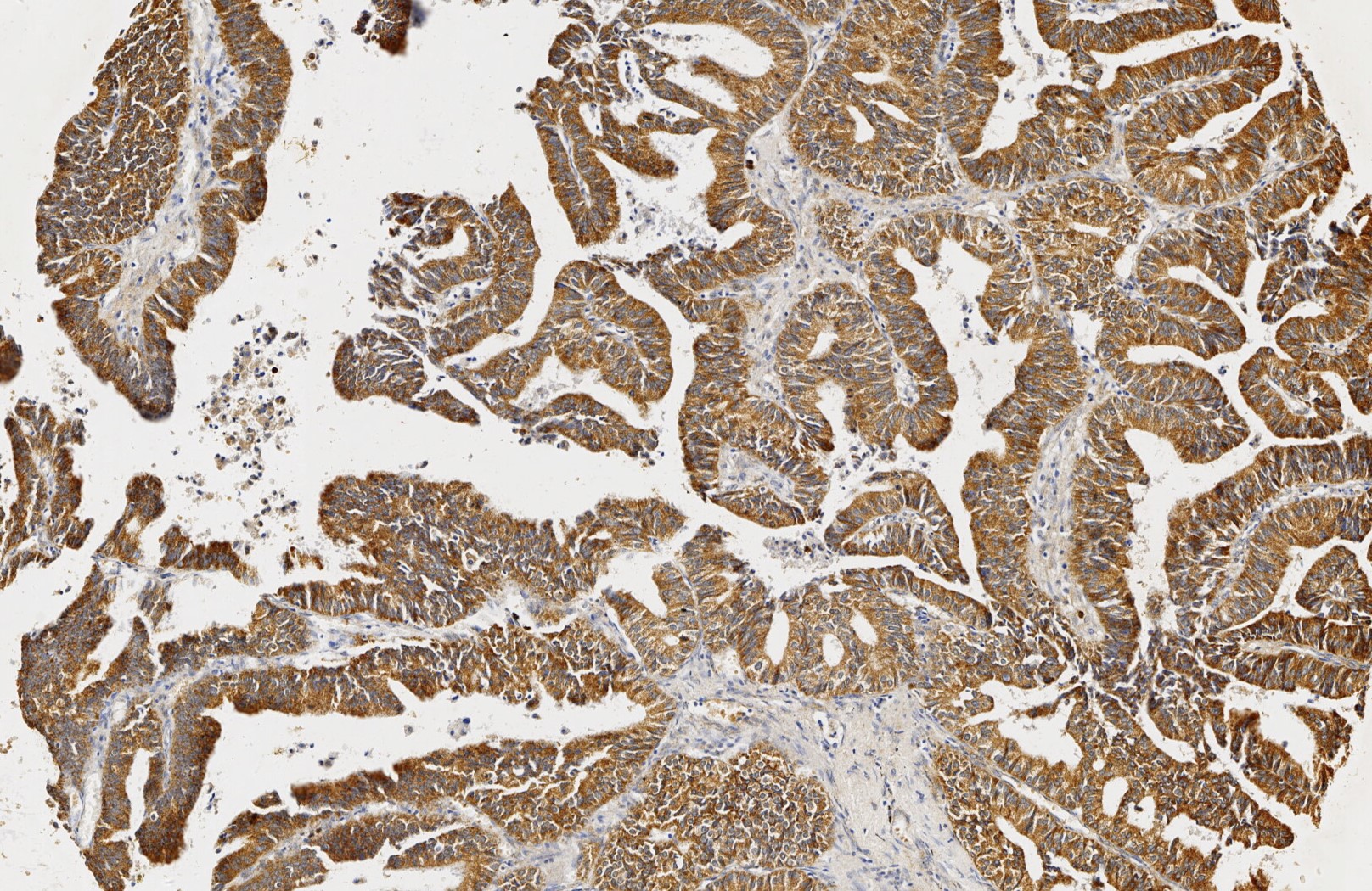

Supplement: Supplementary file 2 [file DataSheet_2.zip › fig 4b. 21T.jpg]

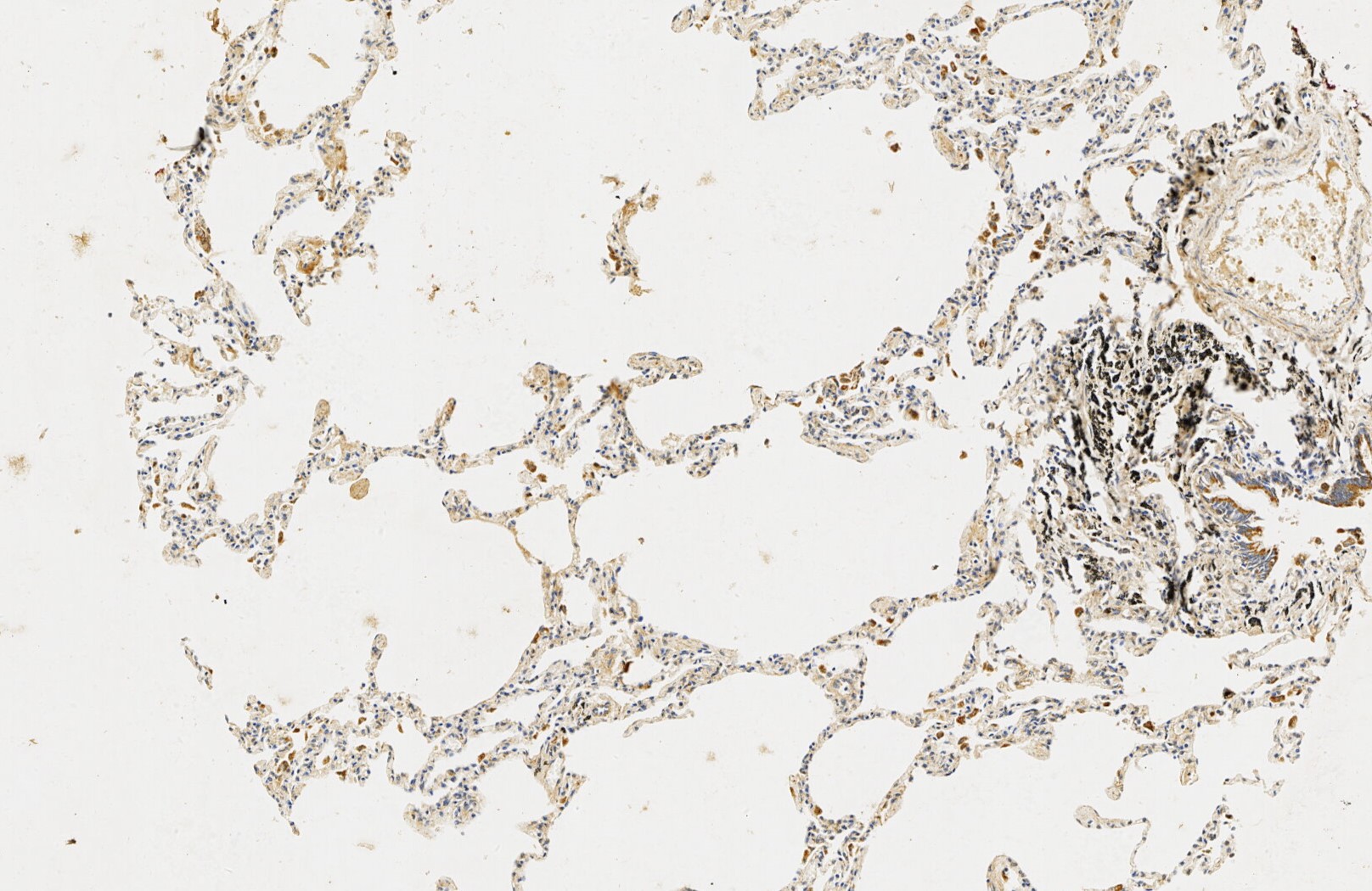

Supplement: Supplementary file 2 [file DataSheet_2.zip › fig 4b. 24N.jpg]

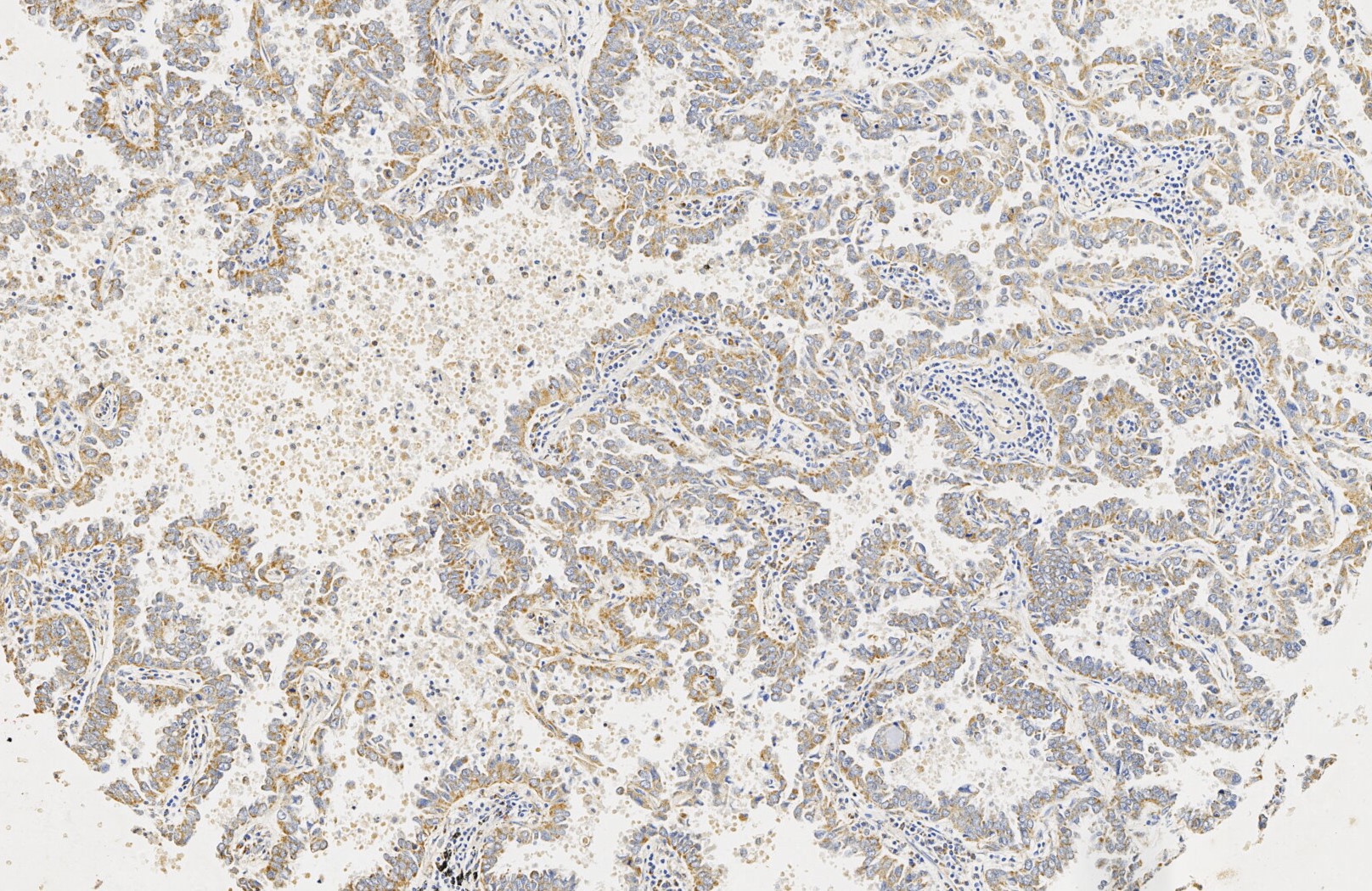

Supplement: Supplementary file 2 [file DataSheet_2.zip › fig 4b. 24T.jpg]

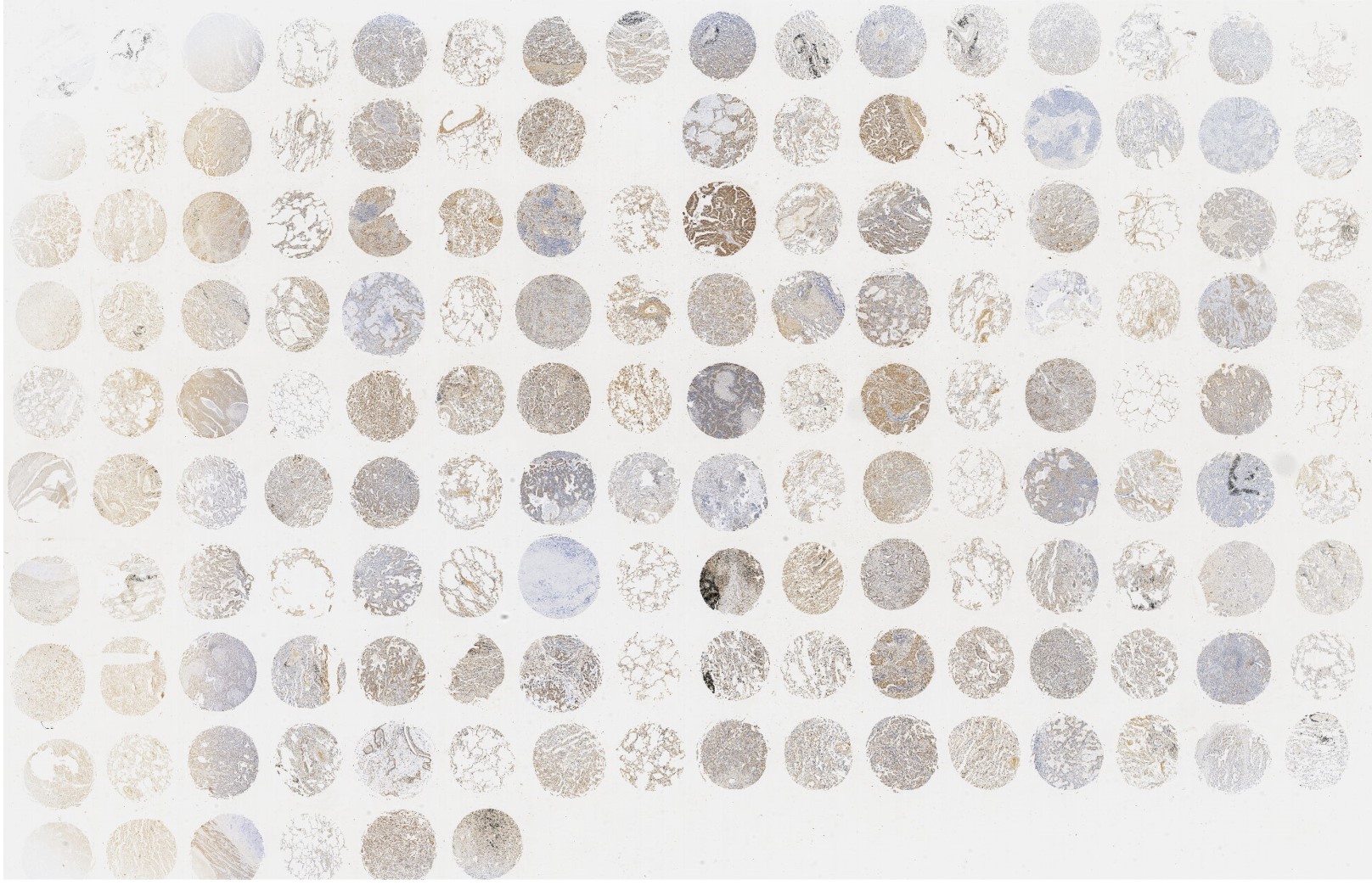

Supplement: Supplementary file 2 [file DataSheet_2.zip › fig 4b. ╖╬╧┘░⌐ALKBH5.jpg]

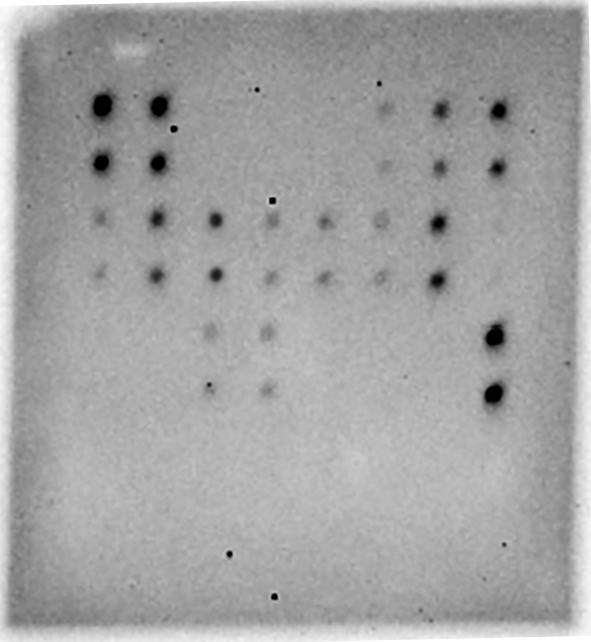

Supplement: Supplementary file 3 [file DataSheet_3.zip › fig 5a. siALKHB5.jpg]

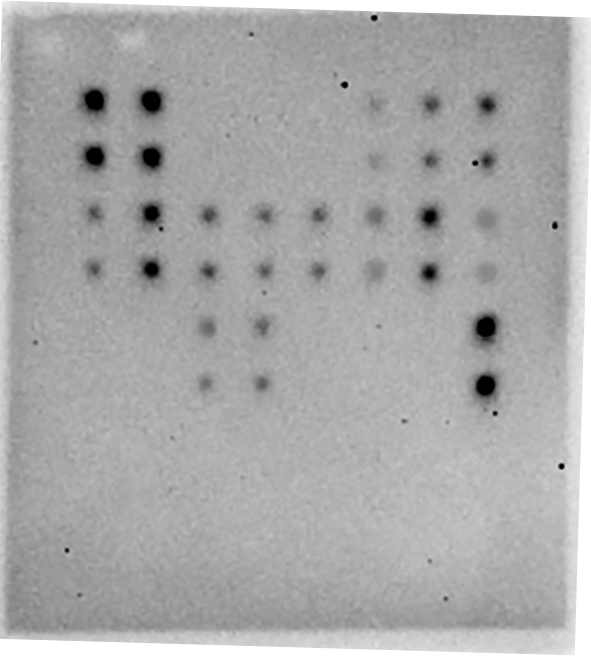

Supplement: Supplementary file 3 [file DataSheet_3.zip › fig 5a. siMETLL3.jpg]

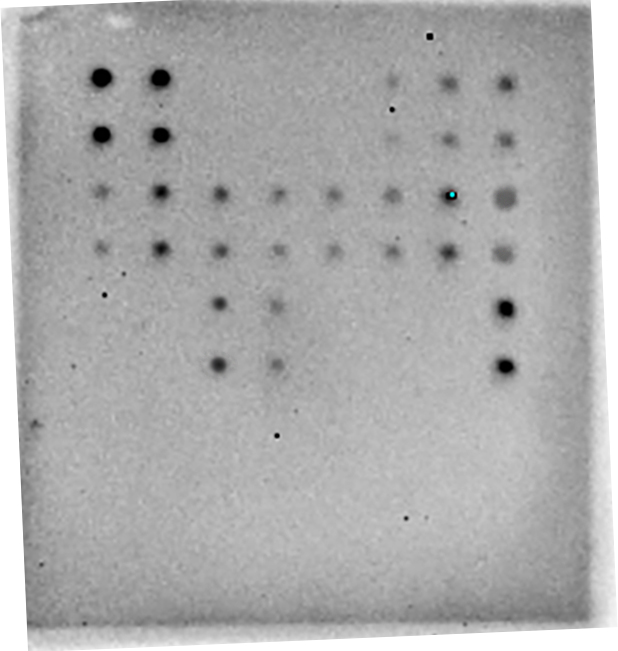

Supplement: Supplementary file 3 [file DataSheet_3.zip › fig 5a. siScrambled.jpg]

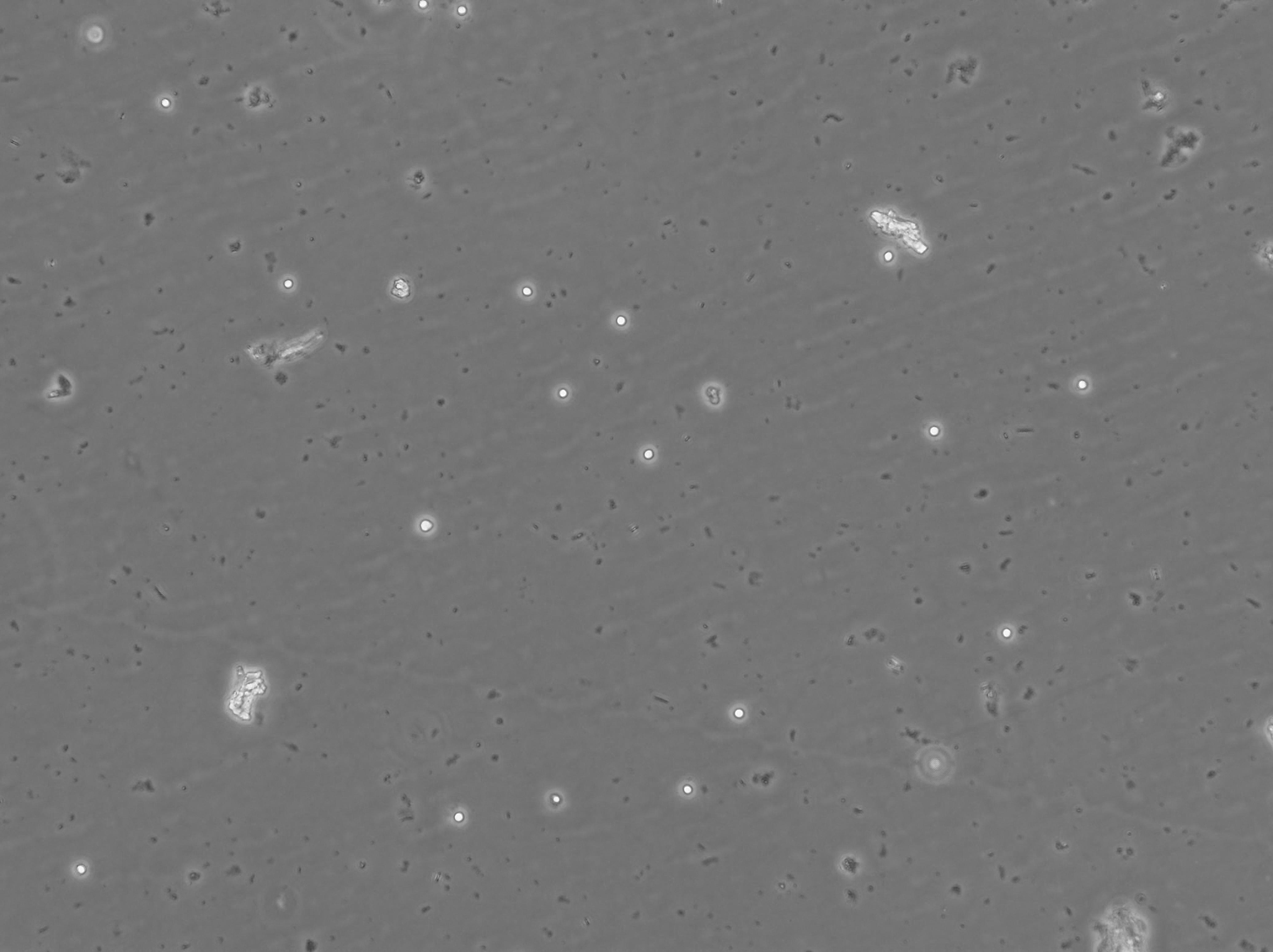

Supplement: Supplementary file 3 [file DataSheet_3.zip › fig 5b. siALKBH5 (1).jpg]

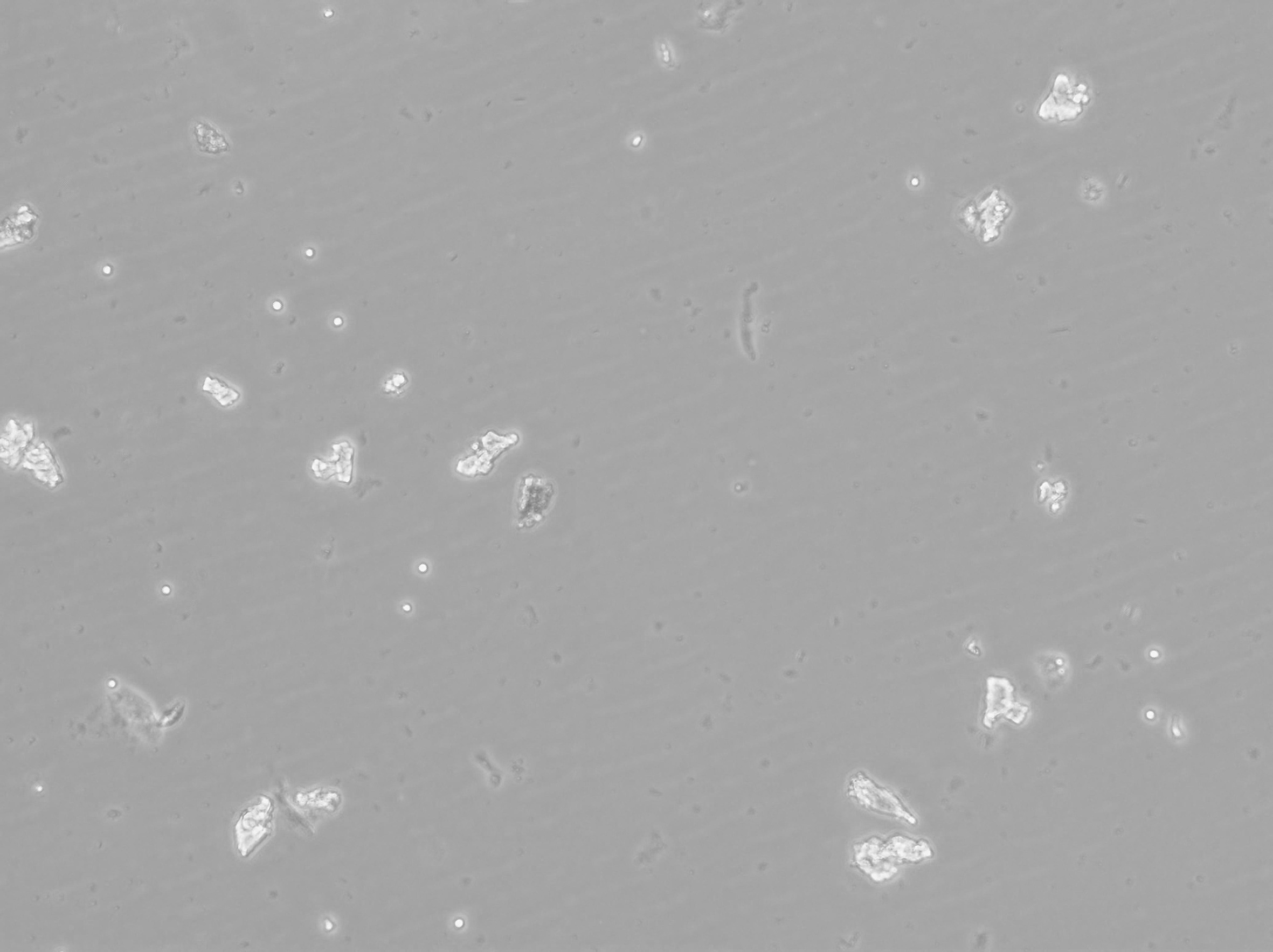

Supplement: Supplementary file 3 [file DataSheet_3.zip › fig 5b. siALKBH5 (2).jpg]

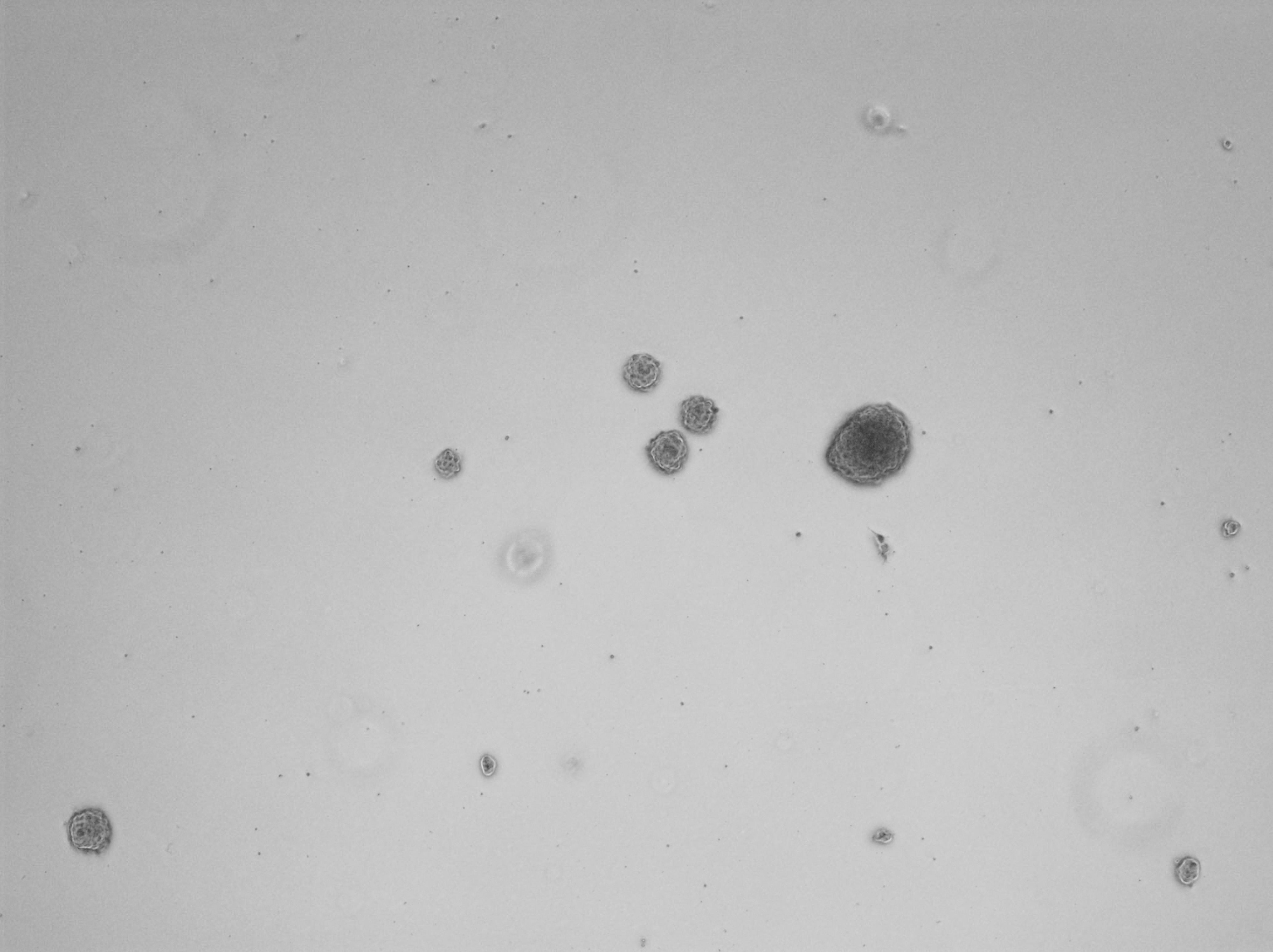

Supplement: Supplementary file 3 [file DataSheet_3.zip › fig 5b. siMETLL3 (1).jpg]

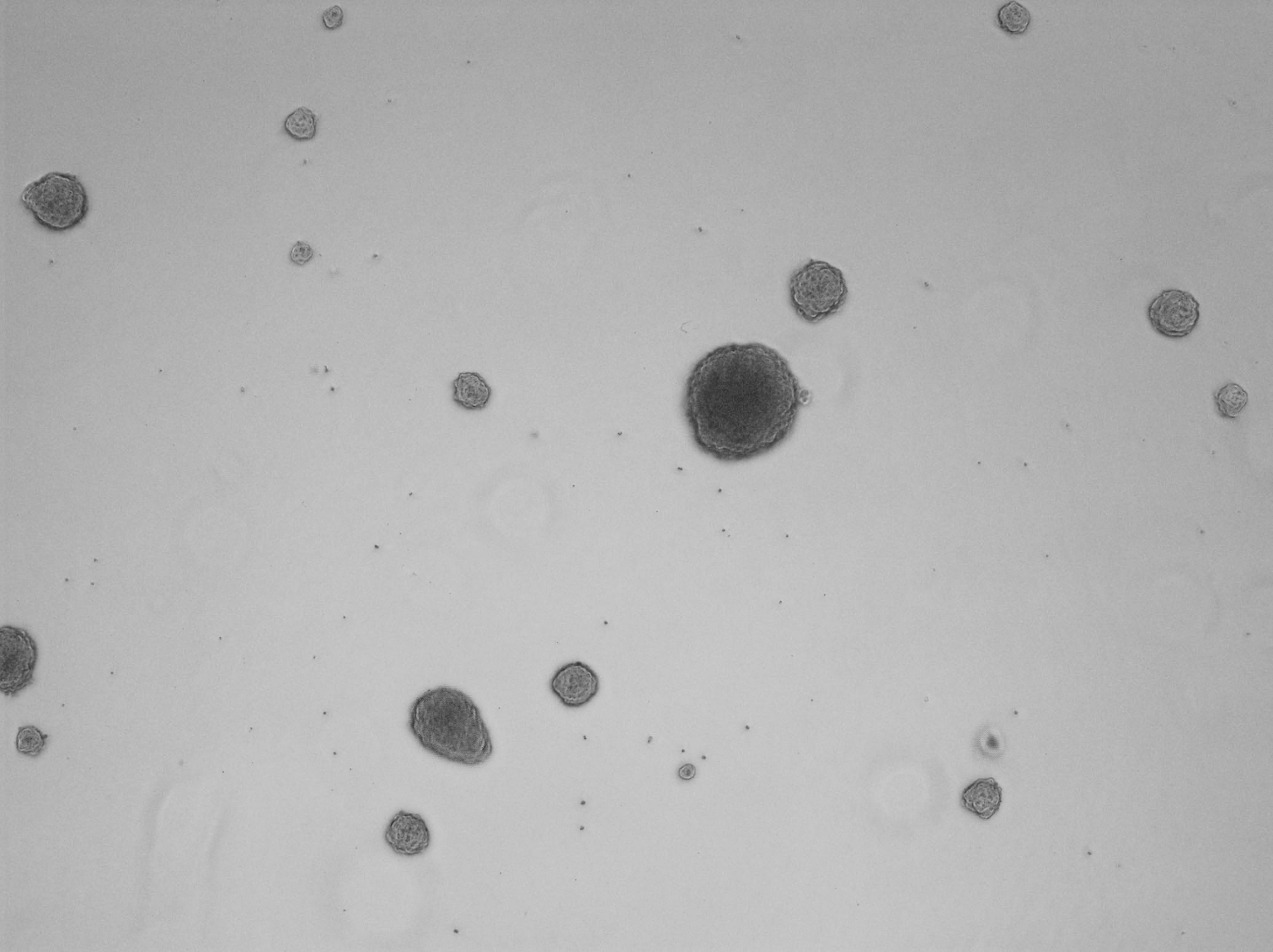

Supplement: Supplementary file 3 [file DataSheet_3.zip › fig 5b. siMETLL3 (2).jpg]

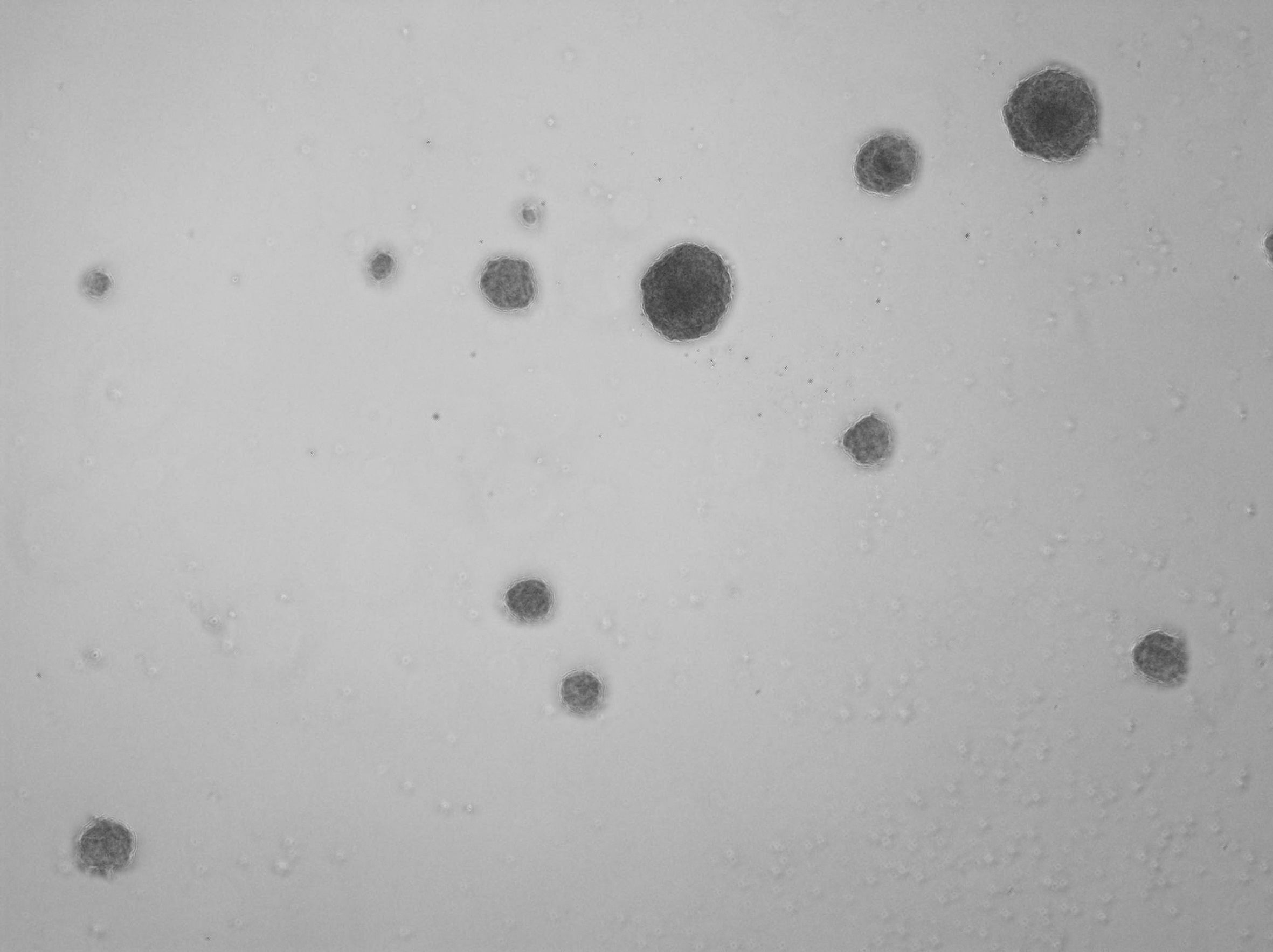

Supplement: Supplementary file 3 [file DataSheet_3.zip › fig 5b. siScrambled (1).jpg]

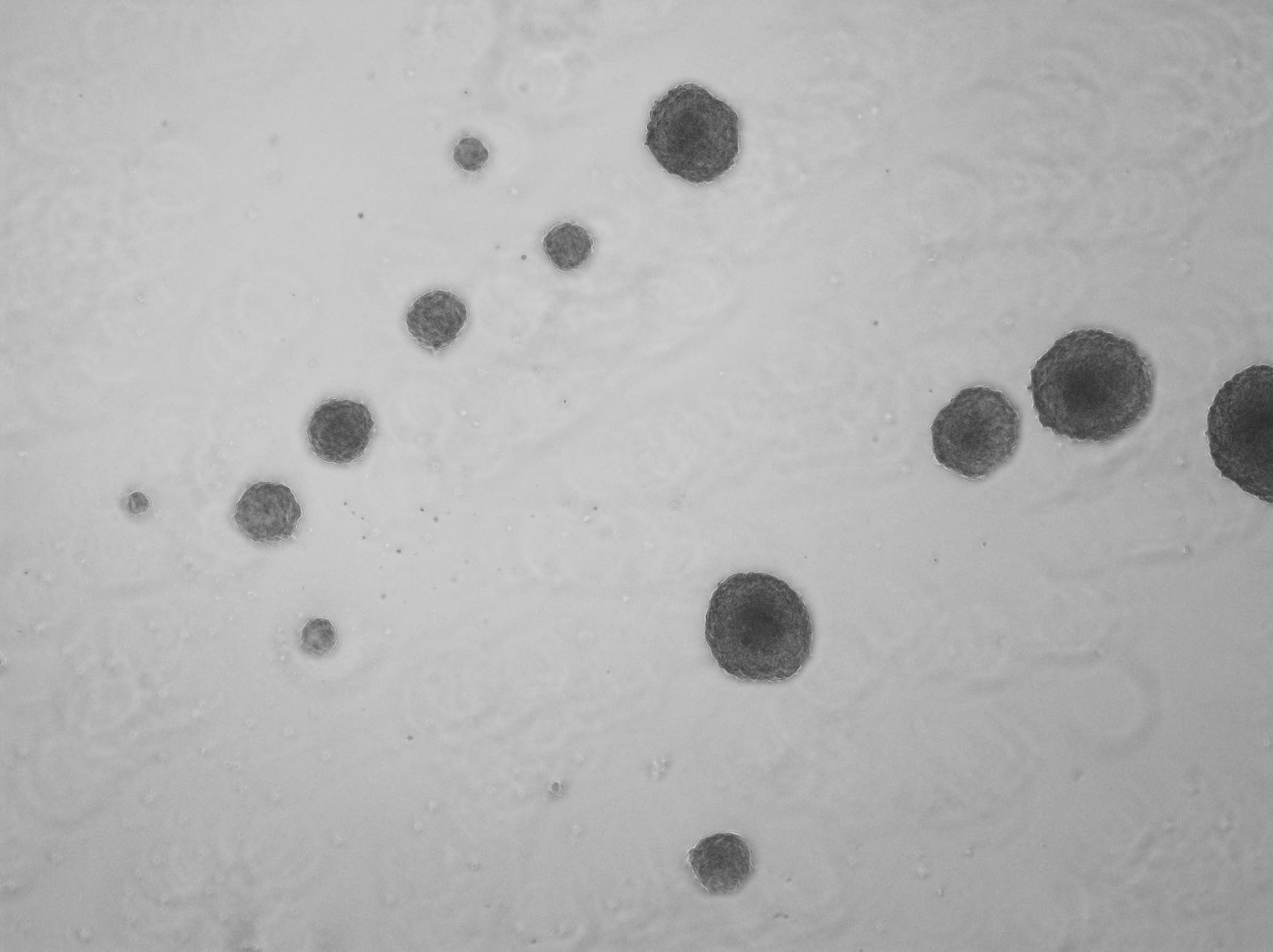

Supplement: Supplementary file 3 [file DataSheet_3.zip › fig 5b. siScrambled (2).jpg]

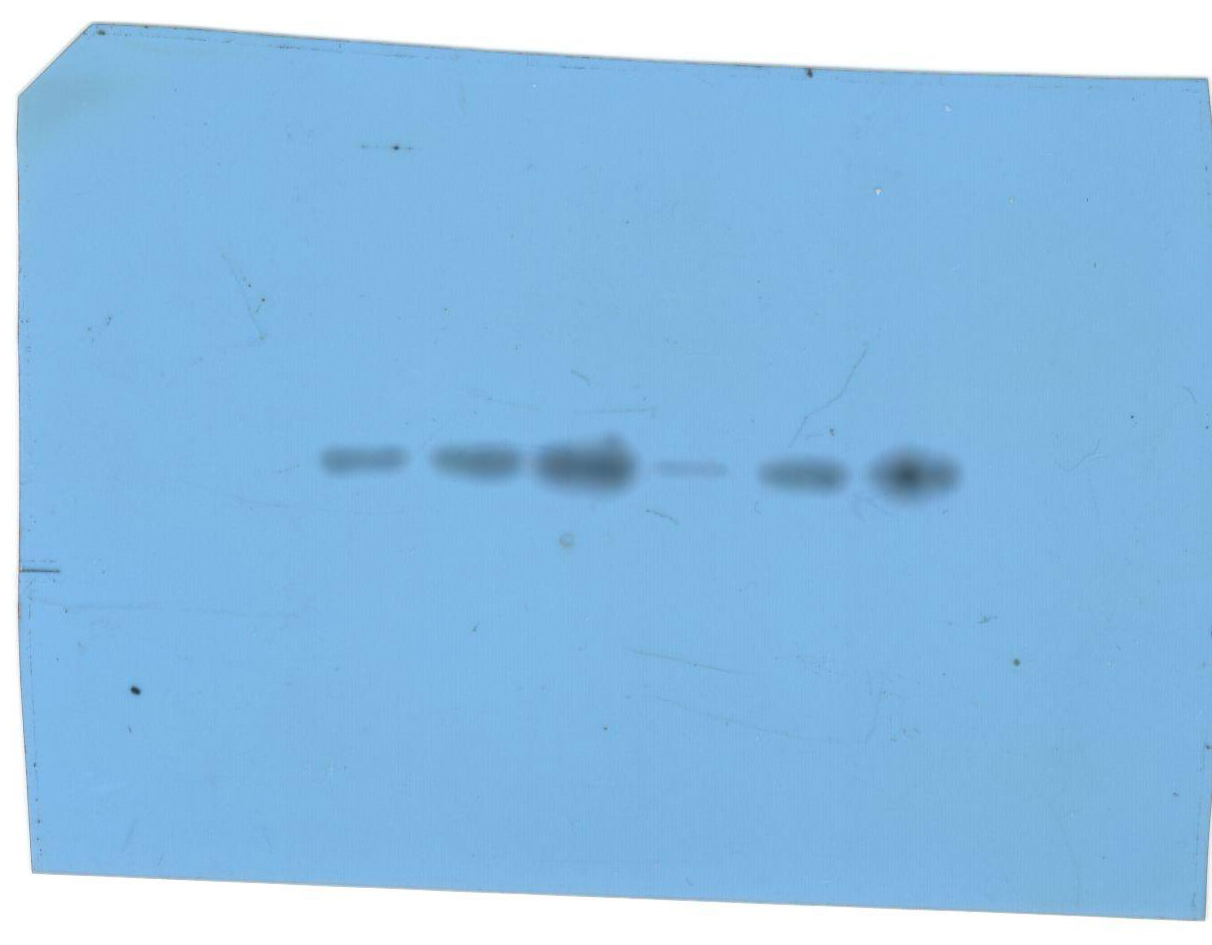

Supplement: Supplementary file 3 [file DataSheet_3.zip › fig 5c. E-cadherin.jpg]

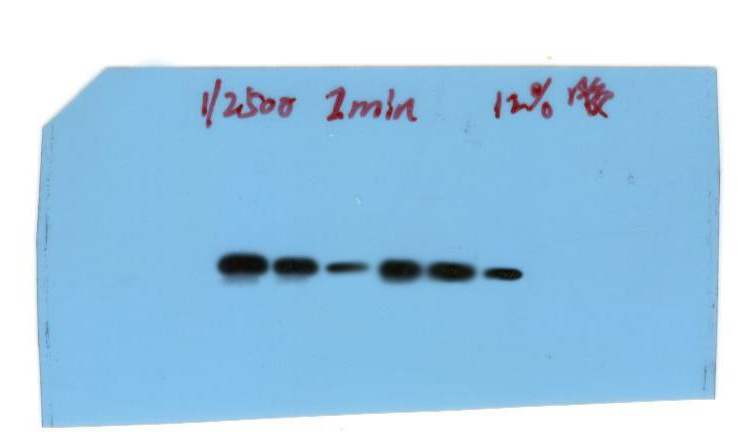

Supplement: Supplementary file 3 [file DataSheet_3.zip › fig 5c. Nanog.jpg]

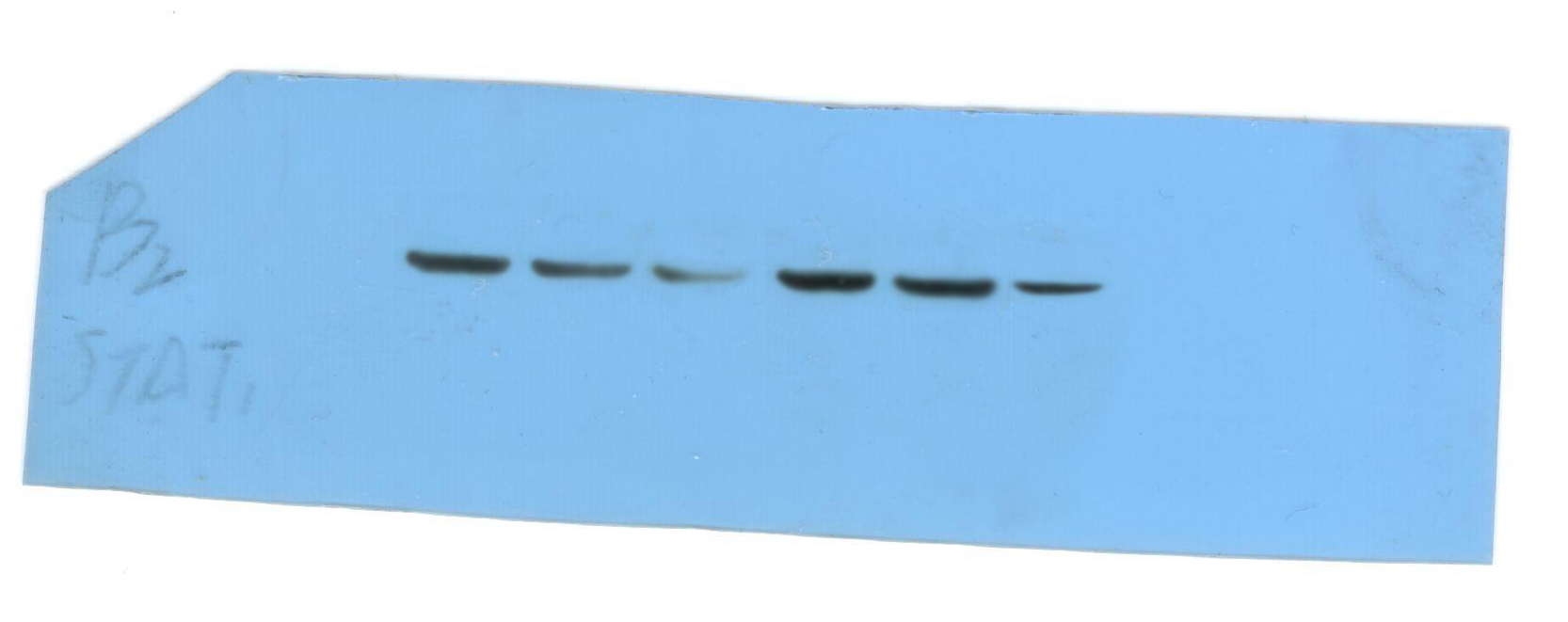

Supplement: Supplementary file 3 [file DataSheet_3.zip › fig 5c. Oc't4.jpg]

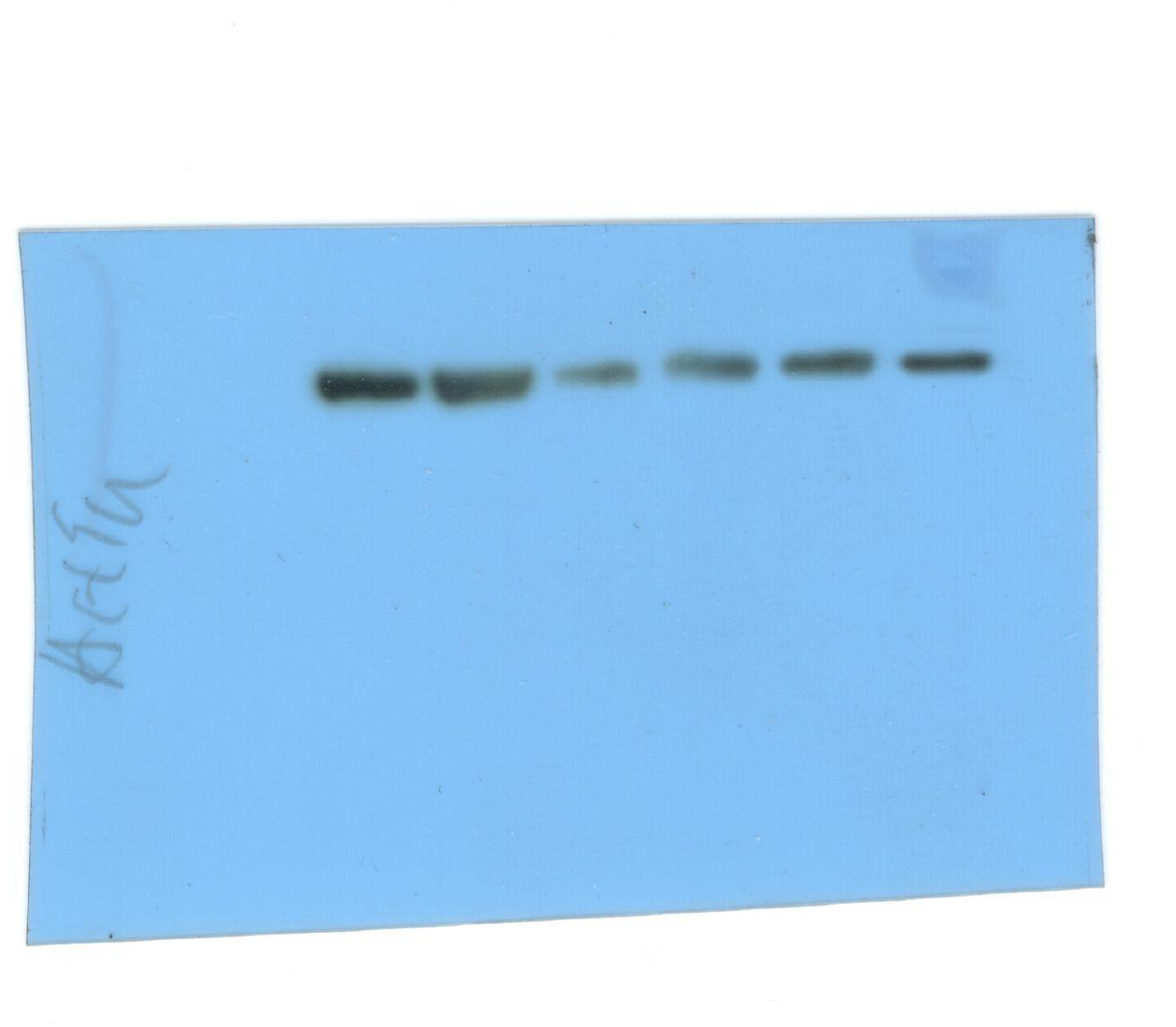

Supplement: Supplementary file 3 [file DataSheet_3.zip › fig 5c. Sox2.jpg]

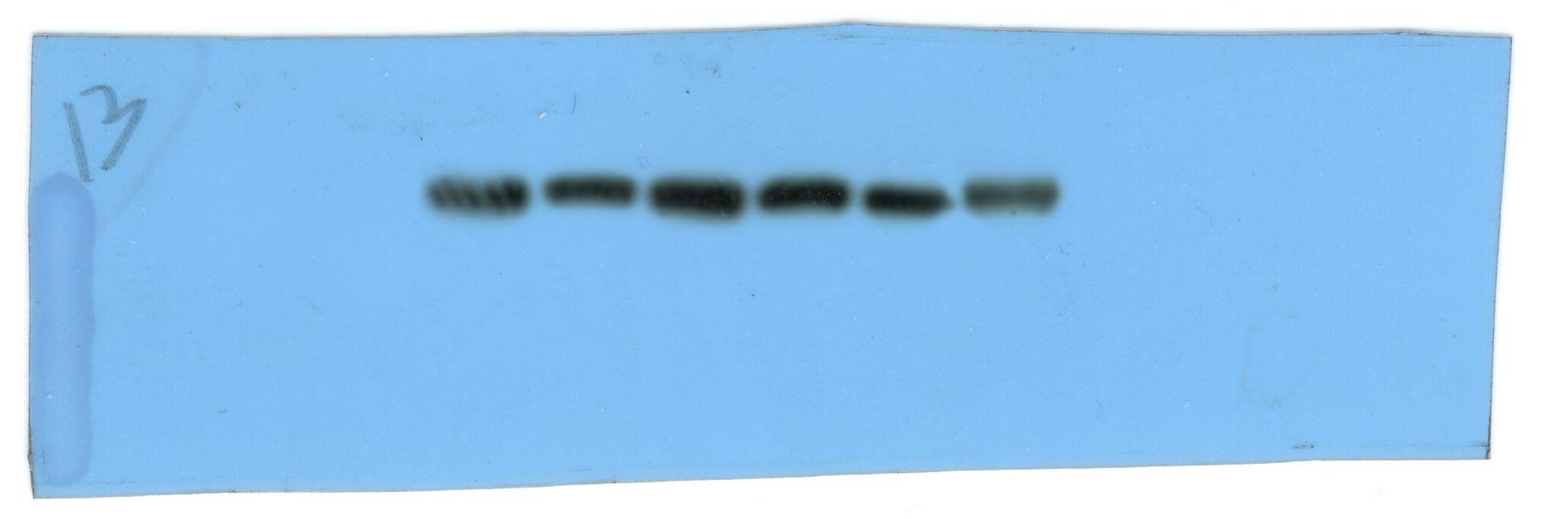

Supplement: Supplementary file 3 [file DataSheet_3.zip › fig 5c. actin.jpg]

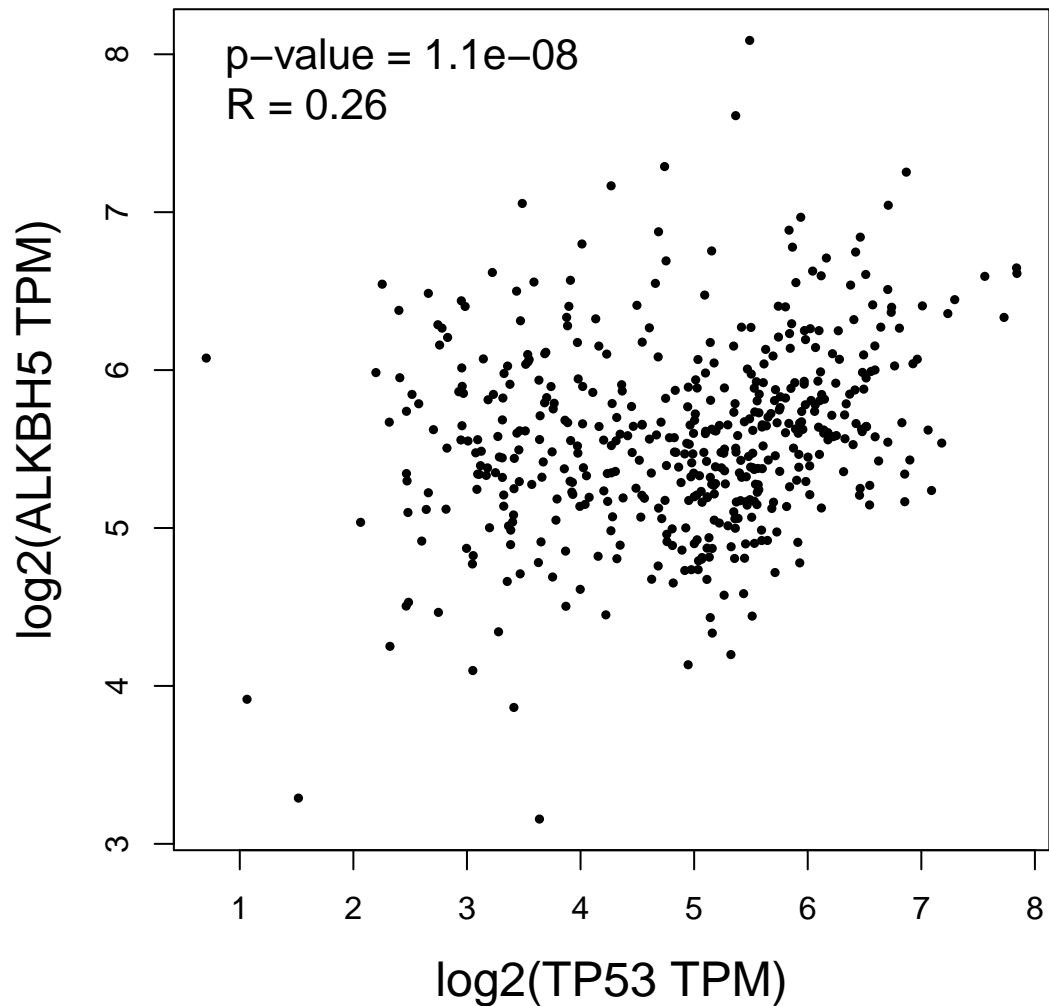

Supplement: Supplementary file 3 [file DataSheet_3.zip › fig 6a. TP53_ALKBH5_correlation_jtrgj.pdf]

p-value =  $2.3\text{e-}14$

R = 0.34

log<sub>2</sub>(ALKBH5 TPM)

7

6

5

4

2

3

4

6

7

log<sub>2</sub>(TP53 TPM)

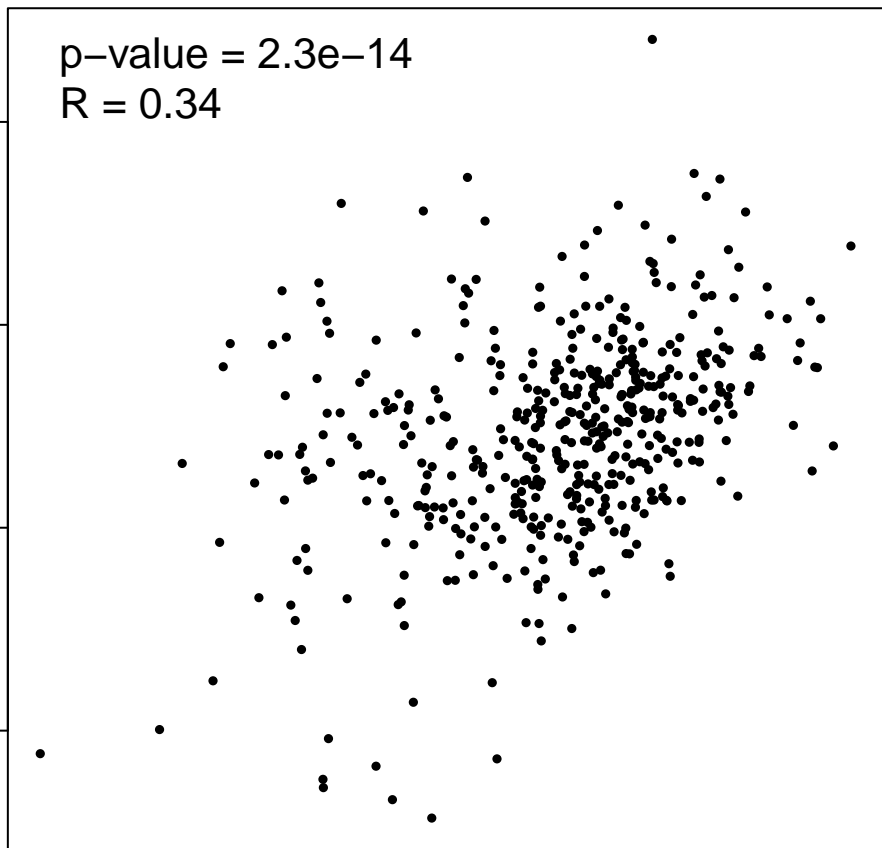

Supplement: Supplementary file 3 [file DataSheet_3.zip › fig 6a. TP53_ALKBH5_correlation_mmamr.pdf]

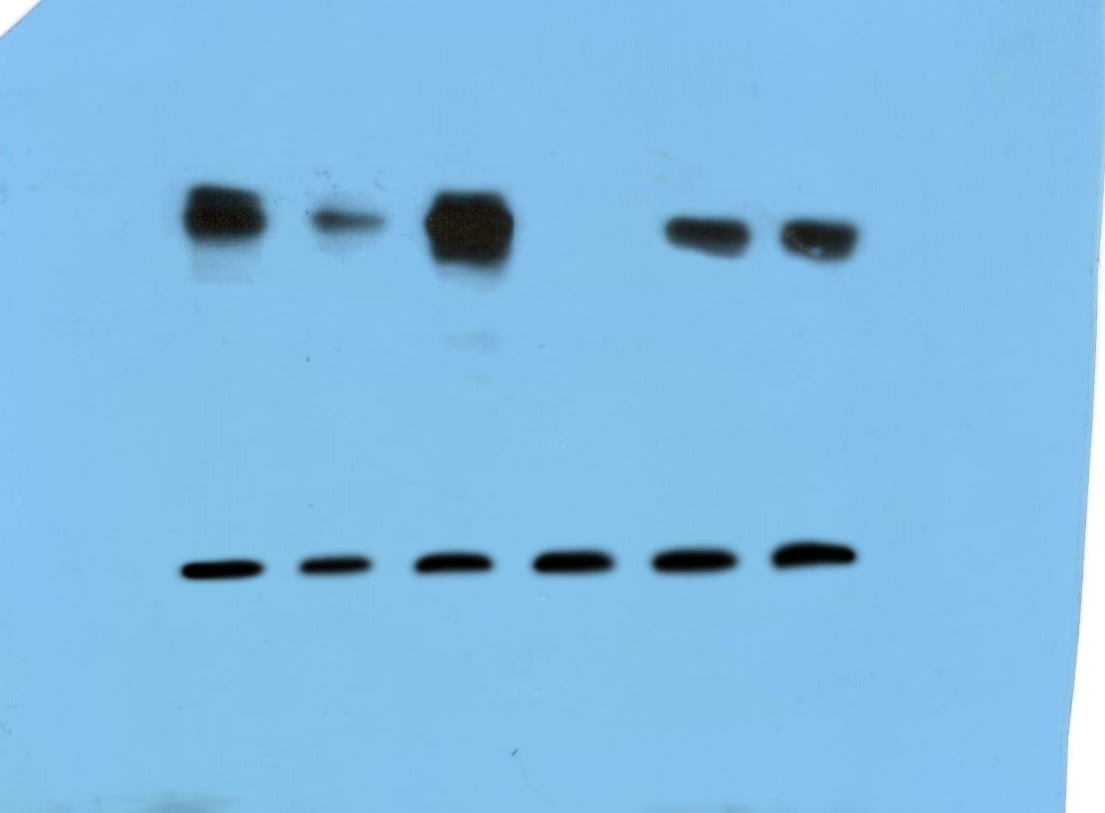

Supplement: Supplementary file 3 [file DataSheet_3.zip › fig 6b. p53, actin.jpg]

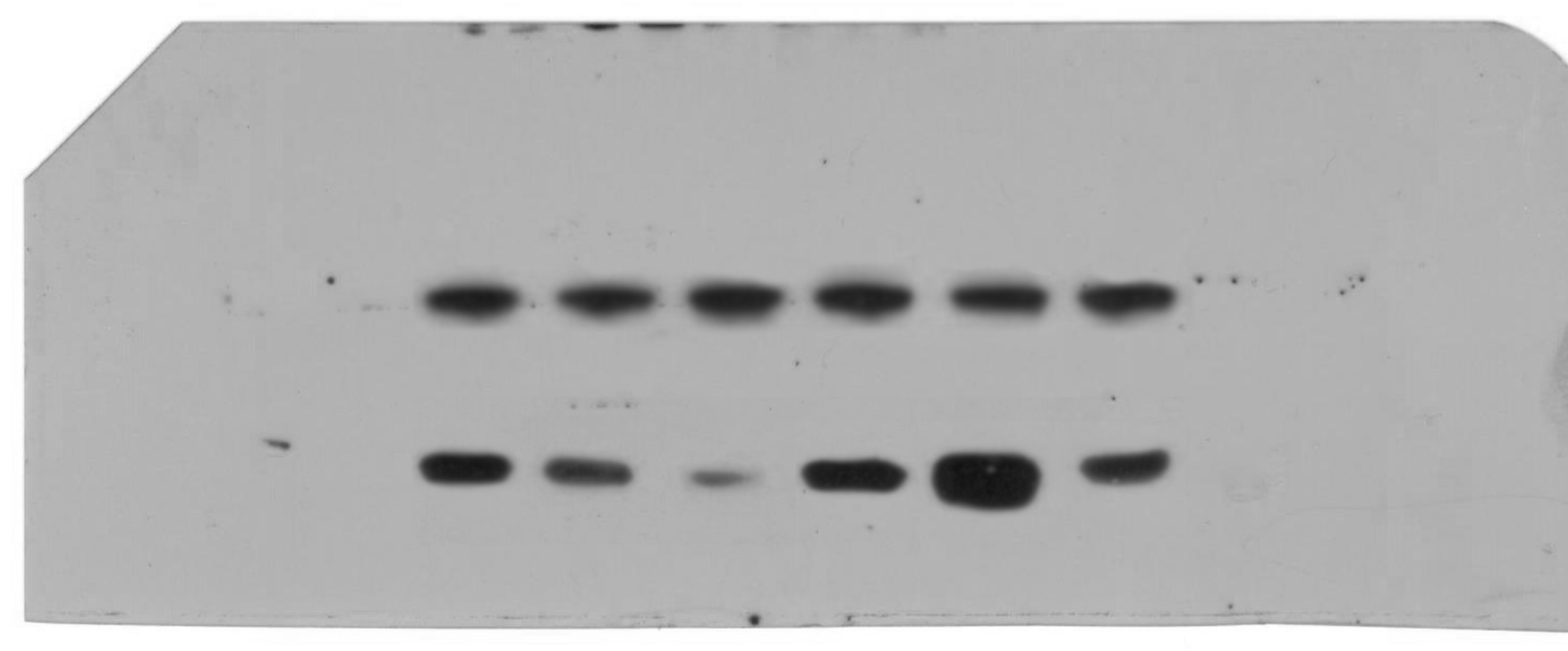

Supplement: Supplementary file 3 [file DataSheet_3.zip › fig 6c. wb.jpg]

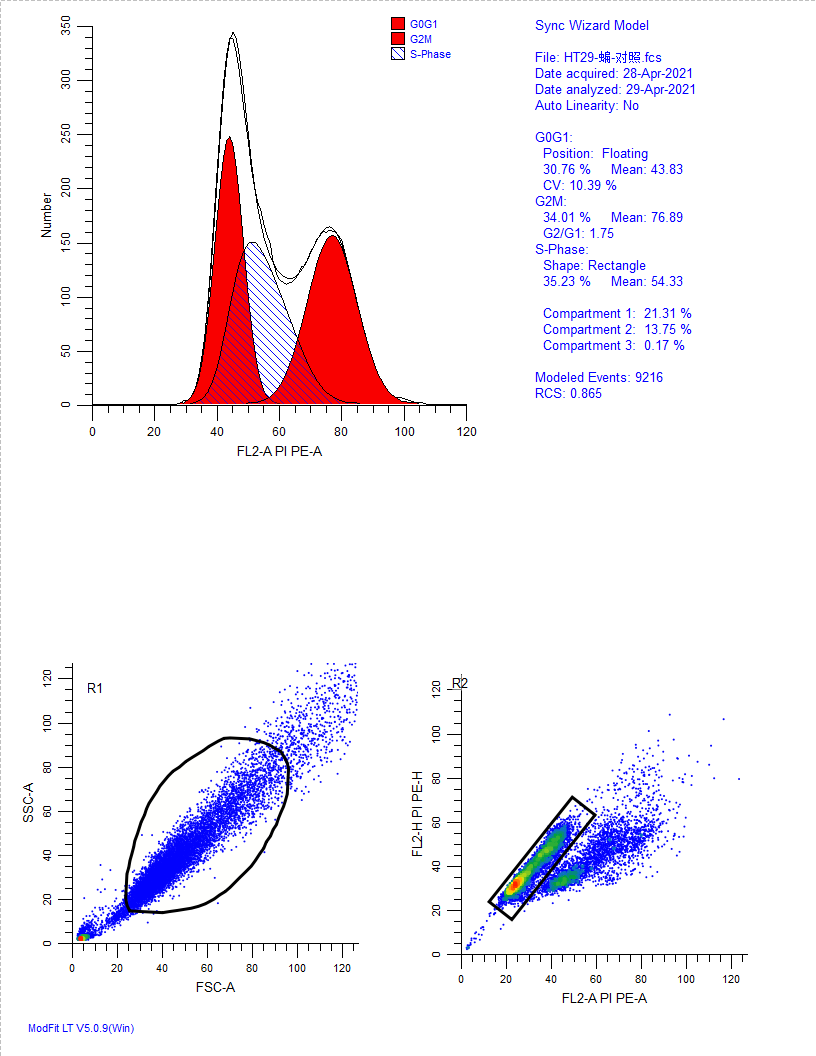

Supplement: Supplementary file 3 [file DataSheet_3.zip › fig 7b. A549 (1).tif]

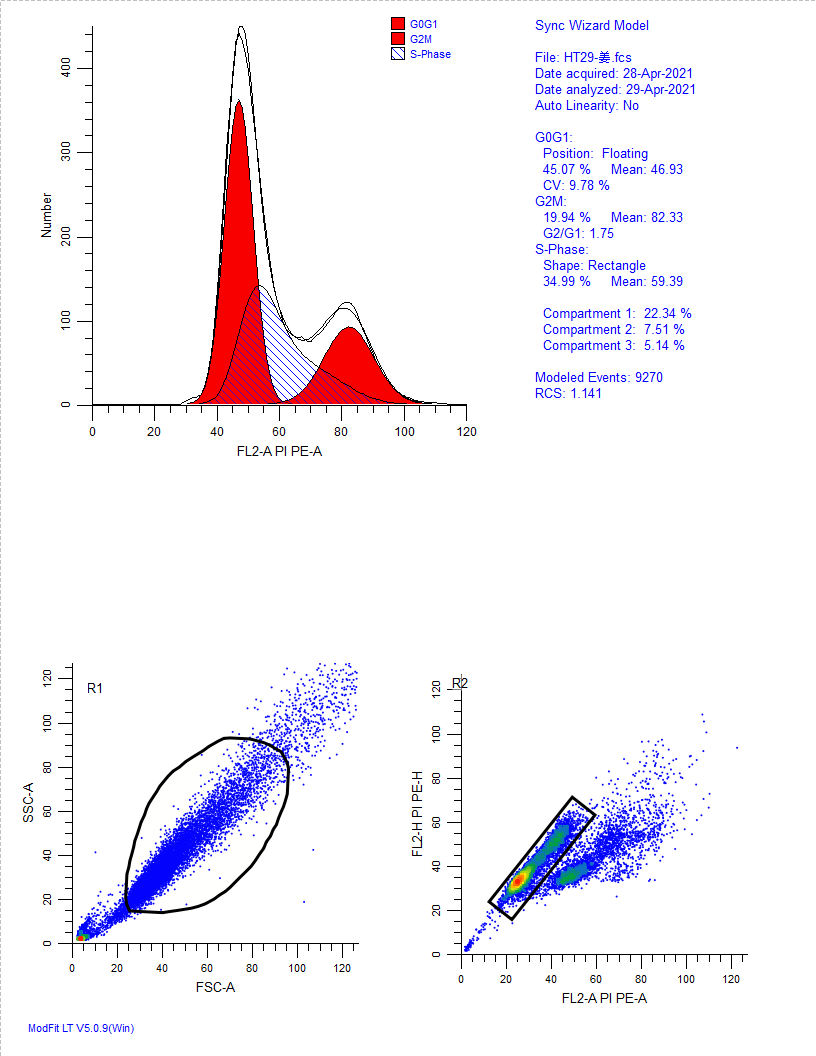

Supplement: Supplementary file 3 [file DataSheet_3.zip › fig 7b. A549 (2).tif]

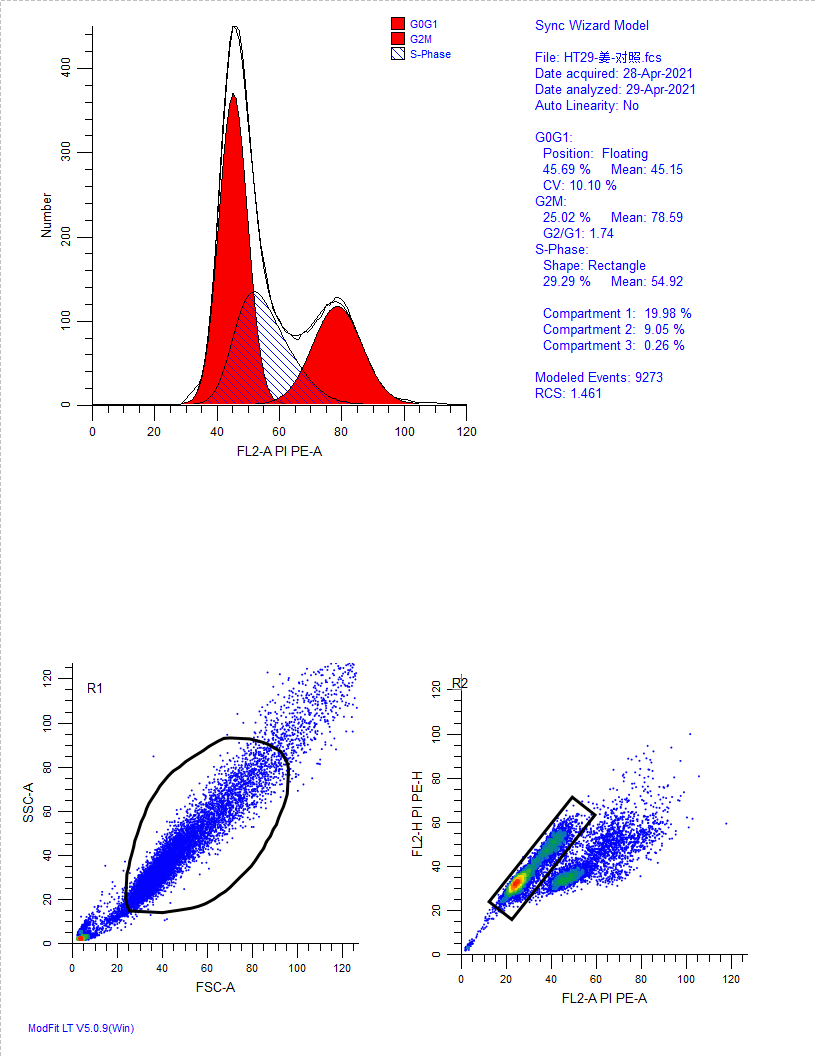

Supplement: Supplementary file 3 [file DataSheet_3.zip › fig 7b. A549 (3).tif]

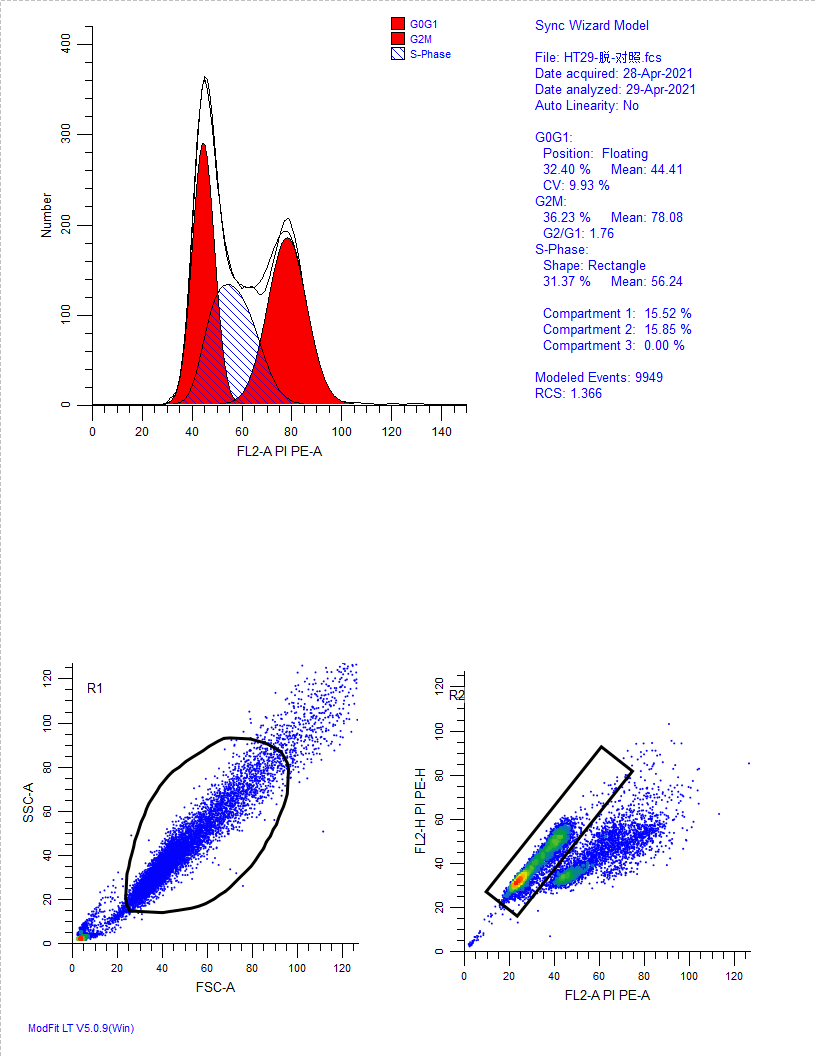

Supplement: Supplementary file 3 [file DataSheet_3.zip › fig 7b. PC-9 (1).tif]

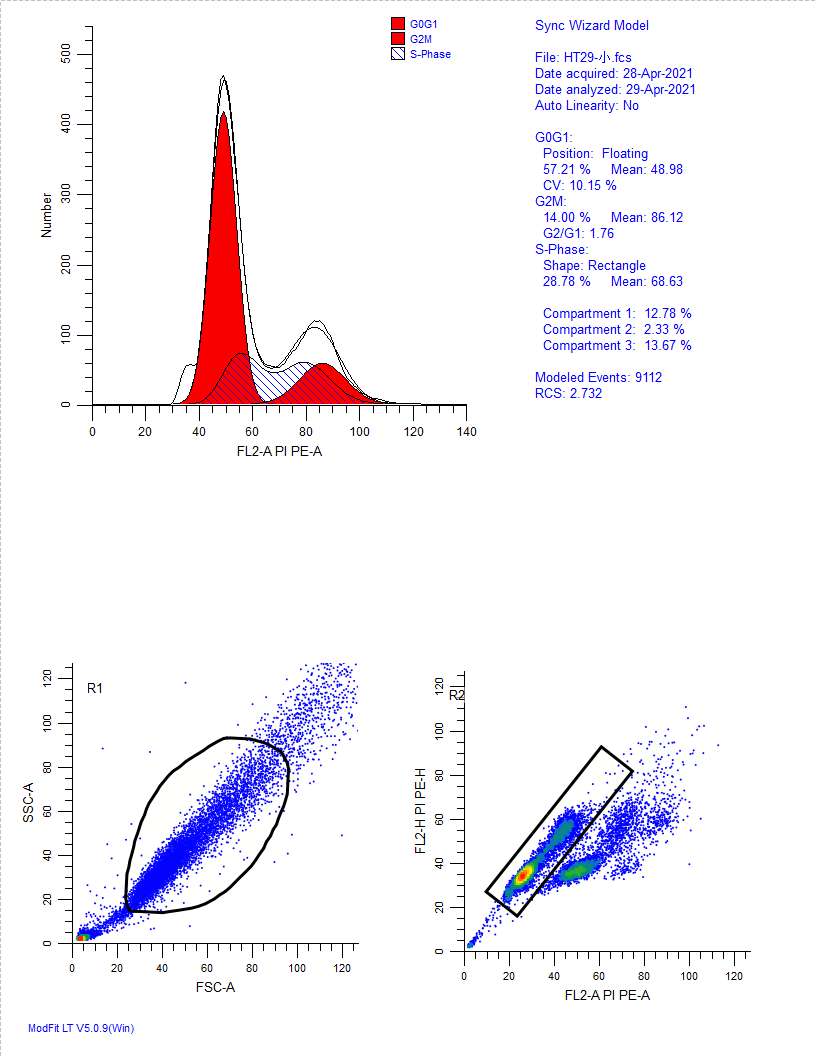

Supplement: Supplementary file 3 [file DataSheet_3.zip › fig 7b. PC-9 (2).tif]

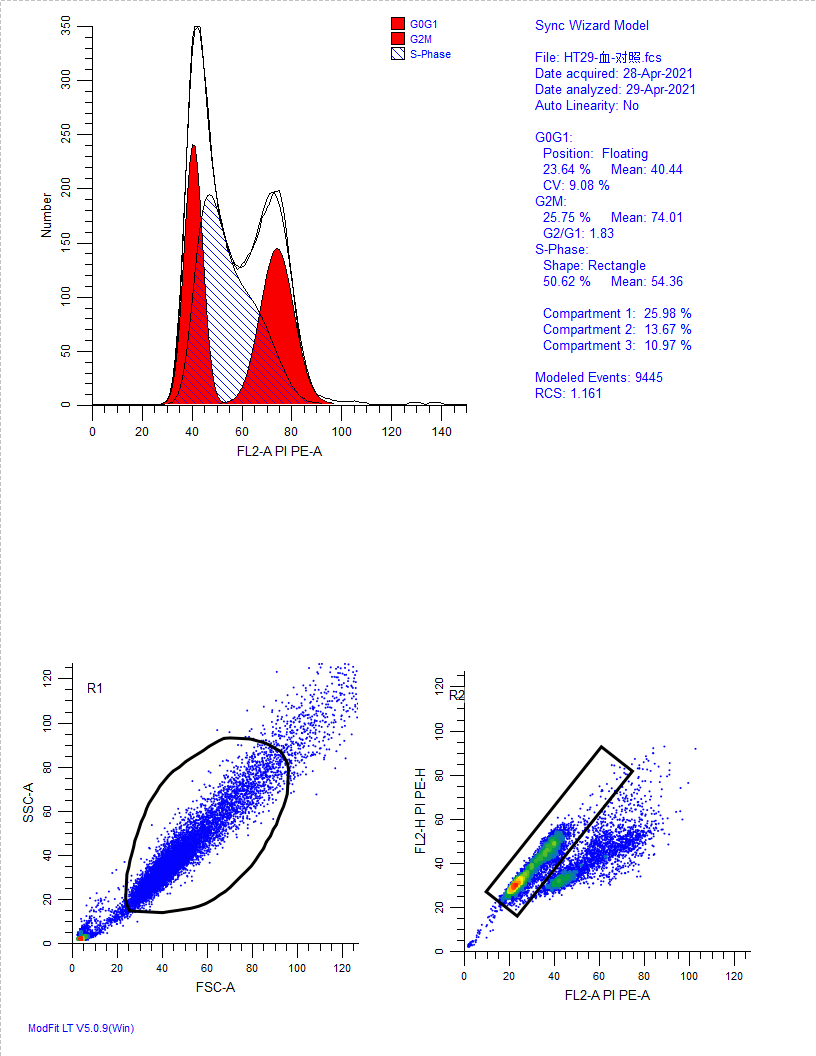

Supplement: Supplementary file 3 [file DataSheet_3.zip › fig 7b. PC-9 (3).tif]

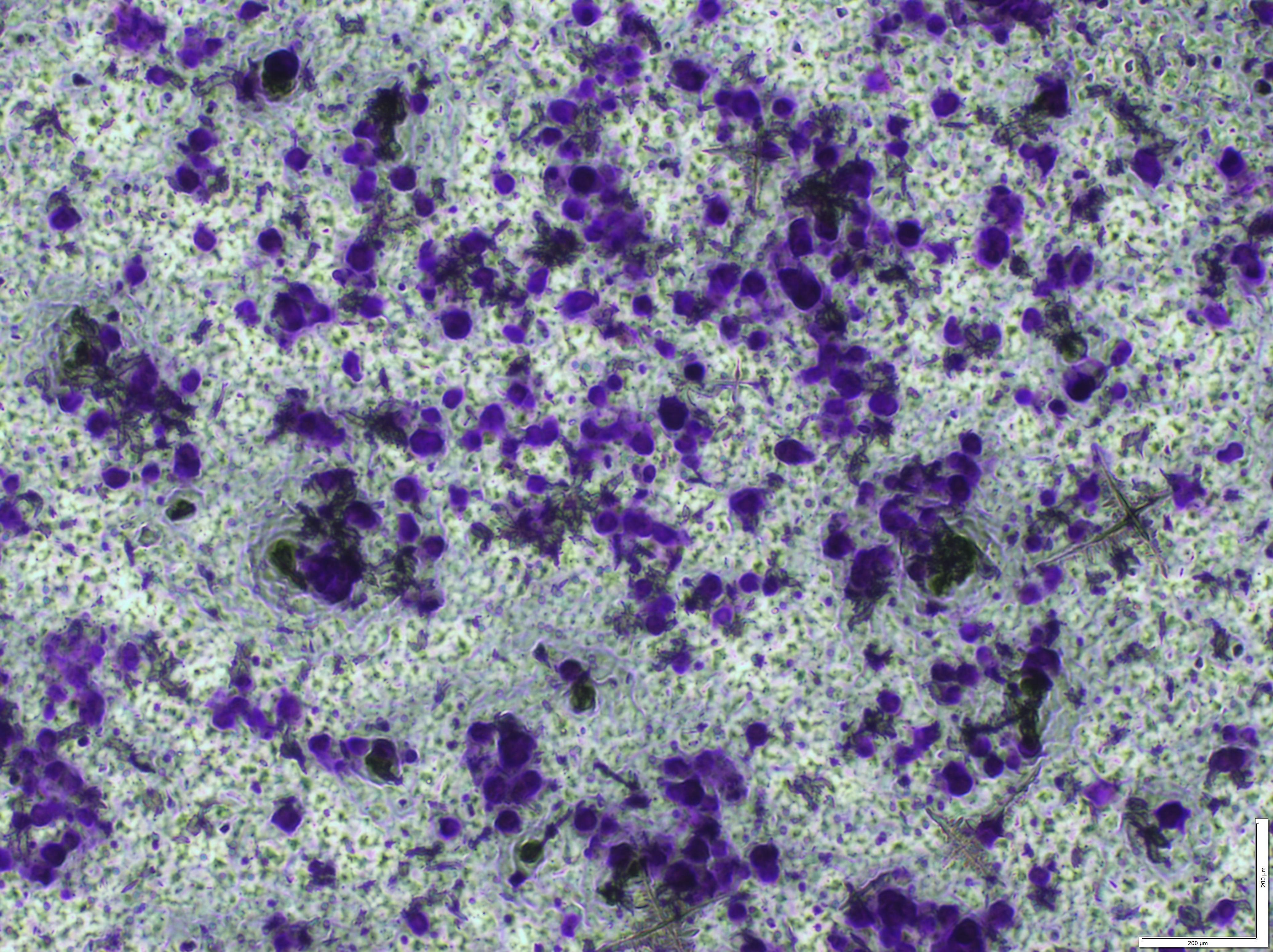

Supplement: Supplementary file 4 [file DataSheet_4.zip › fig 7c. A549 (1).jpg]

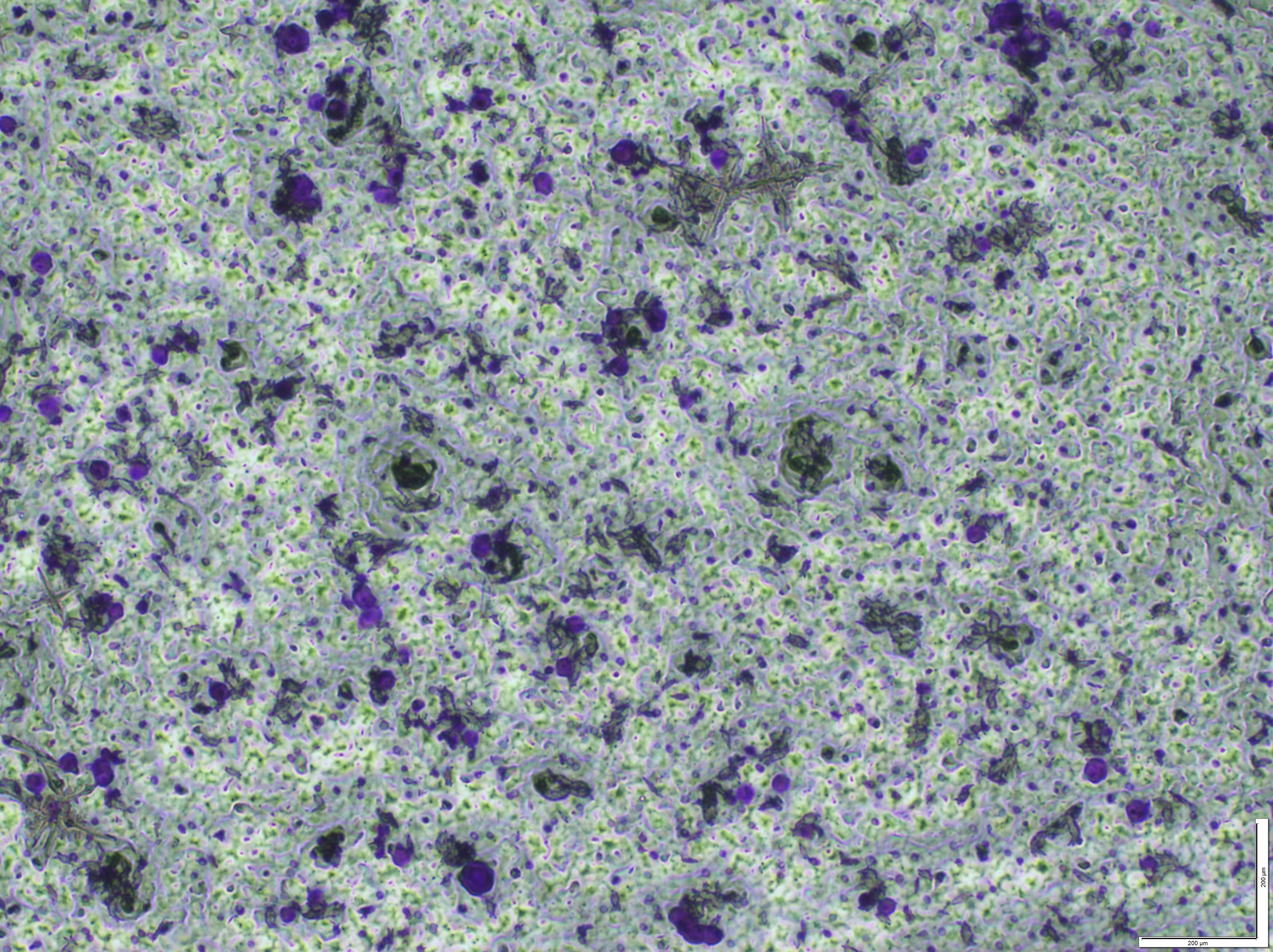

Supplement: Supplementary file 4 [file DataSheet_4.zip › fig 7c. A549 (2).jpg]

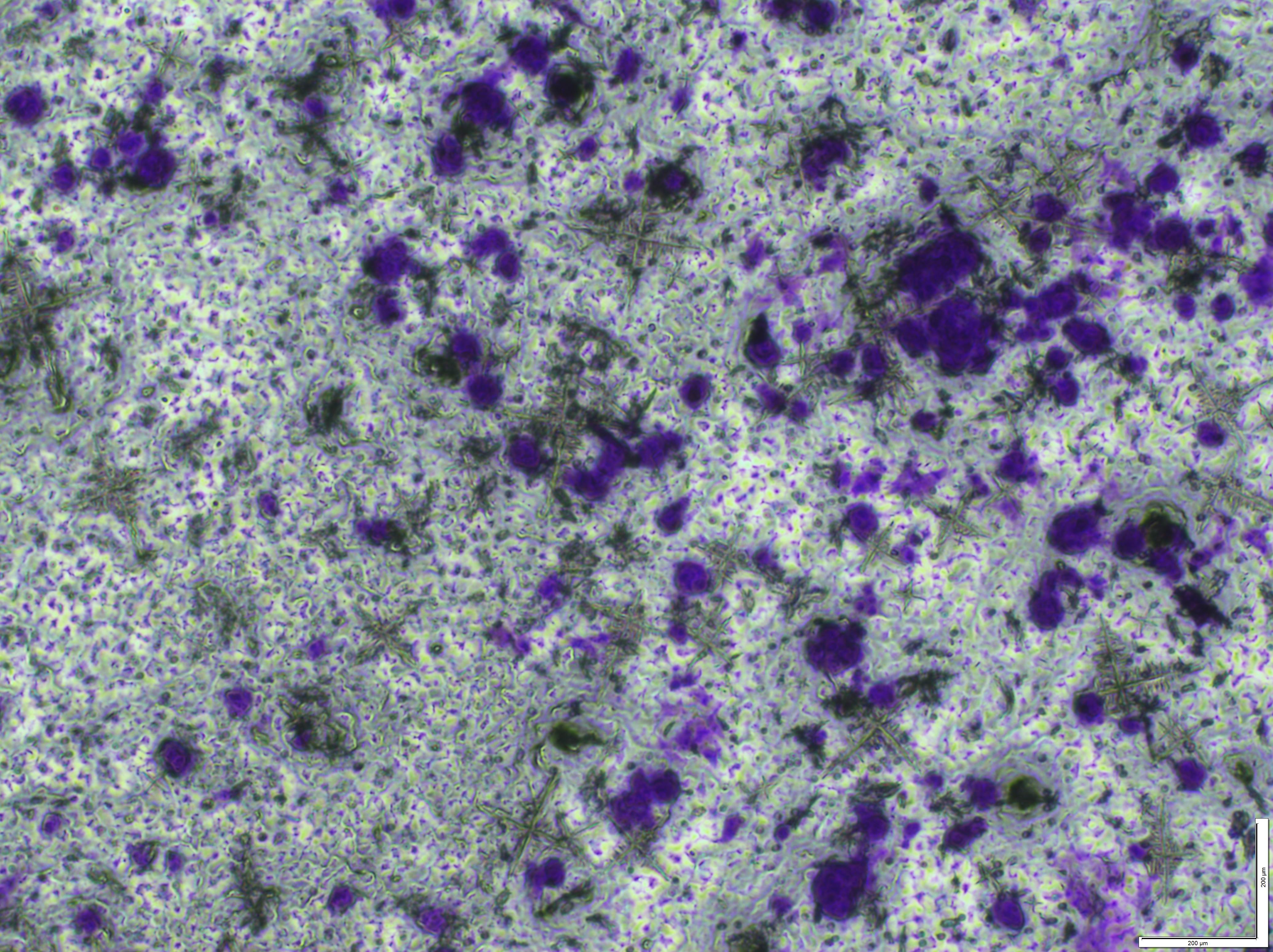

Supplement: Supplementary file 4 [file DataSheet_4.zip › fig 7c. A549 (3).jpg]

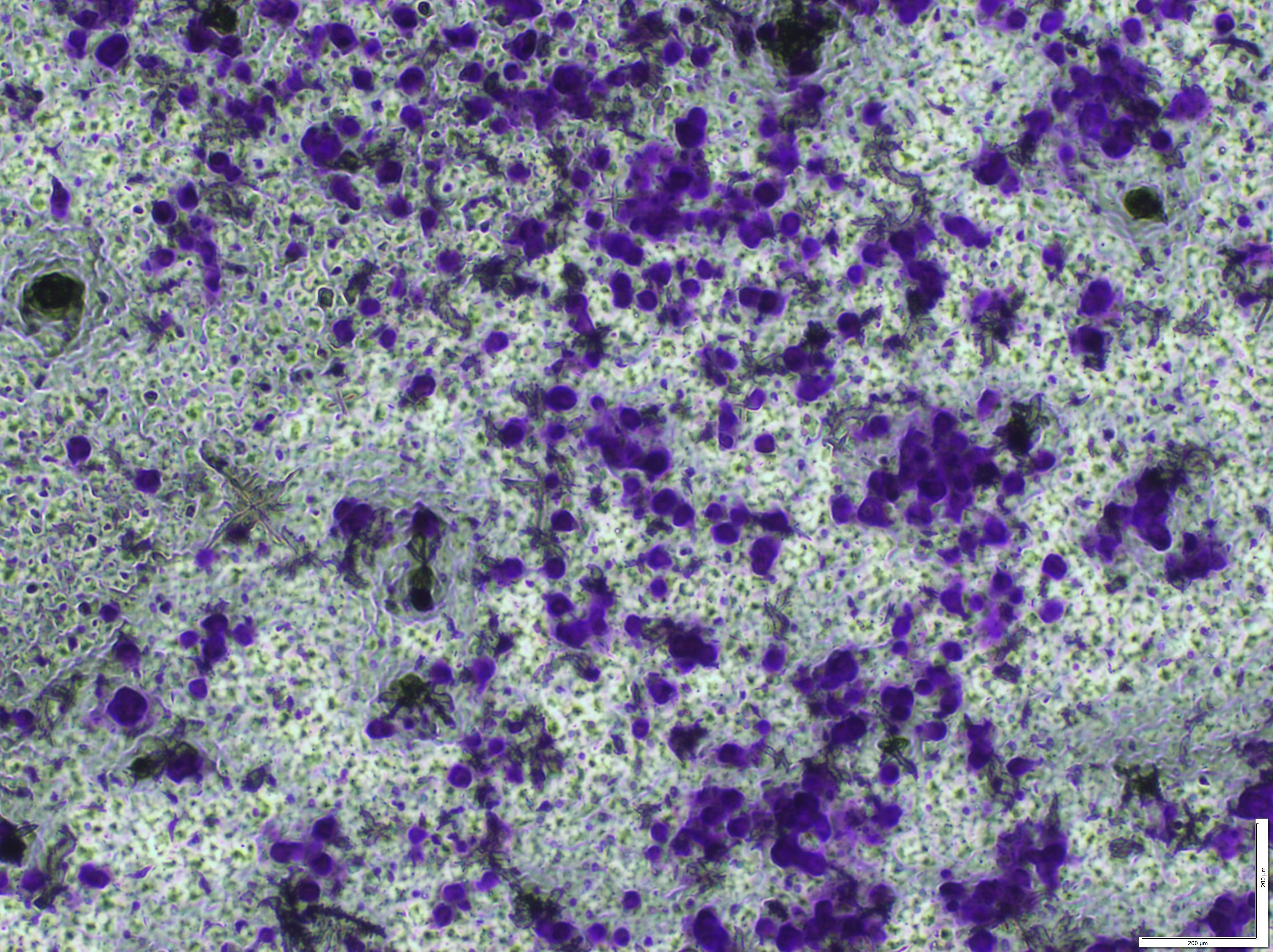

Supplement: Supplementary file 4 [file DataSheet_4.zip › fig 7c. PC-9 (1).jpg]

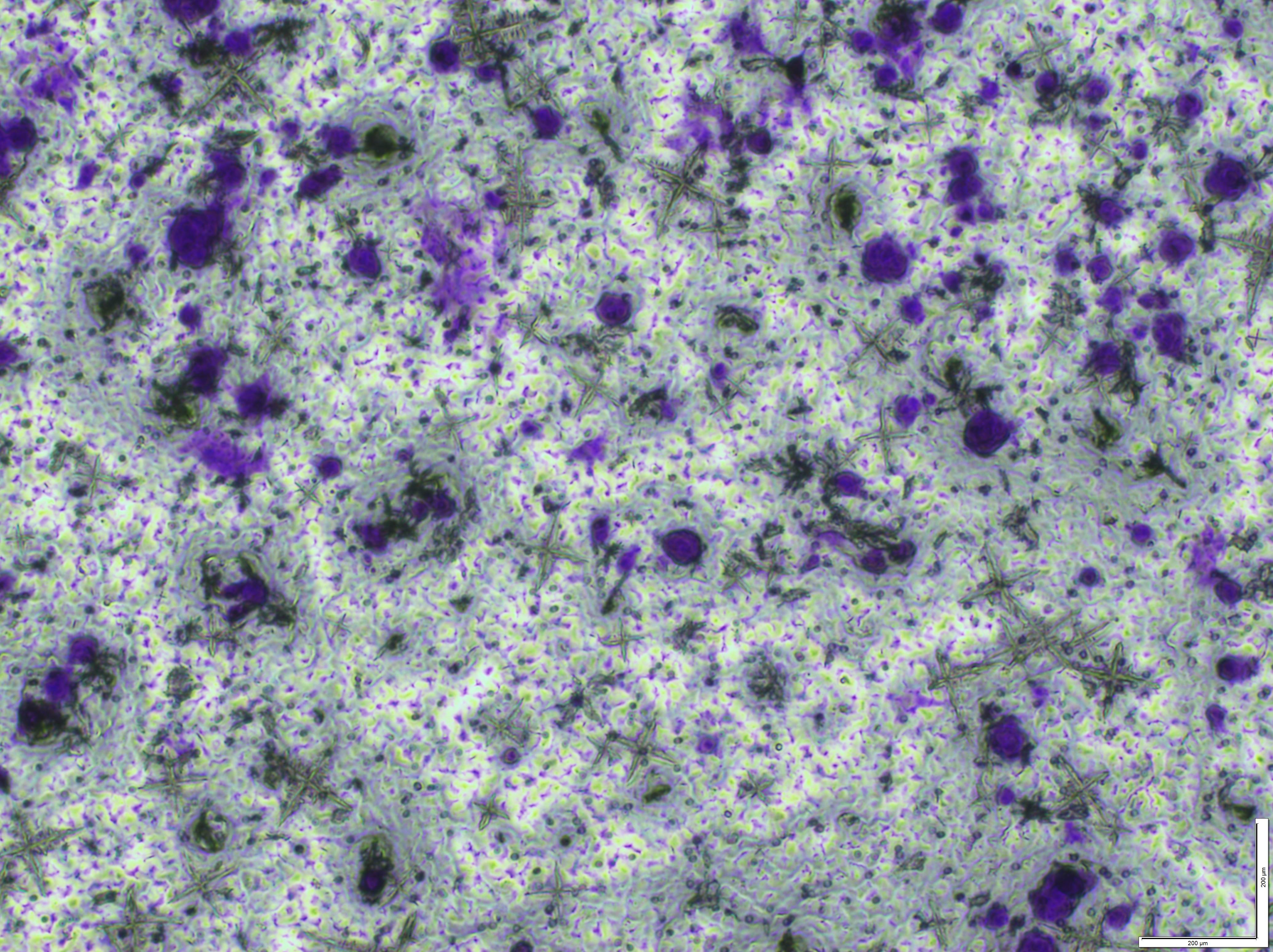

Supplement: Supplementary file 4 [file DataSheet_4.zip › fig 7c. PC-9 (2).jpg]

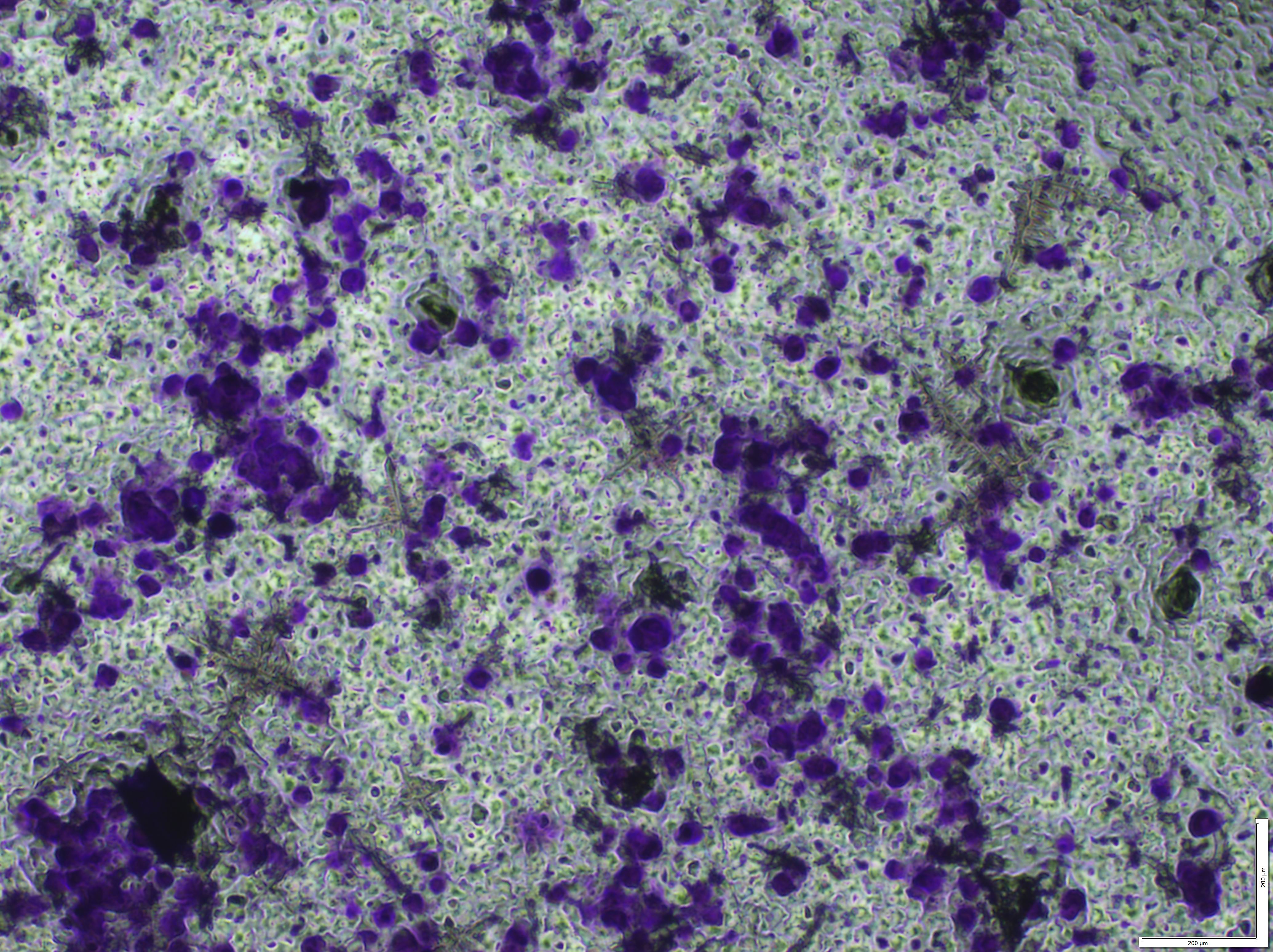

Supplement: Supplementary file 4 [file DataSheet_4.zip › fig 7c. PC-9 (3).jpg]

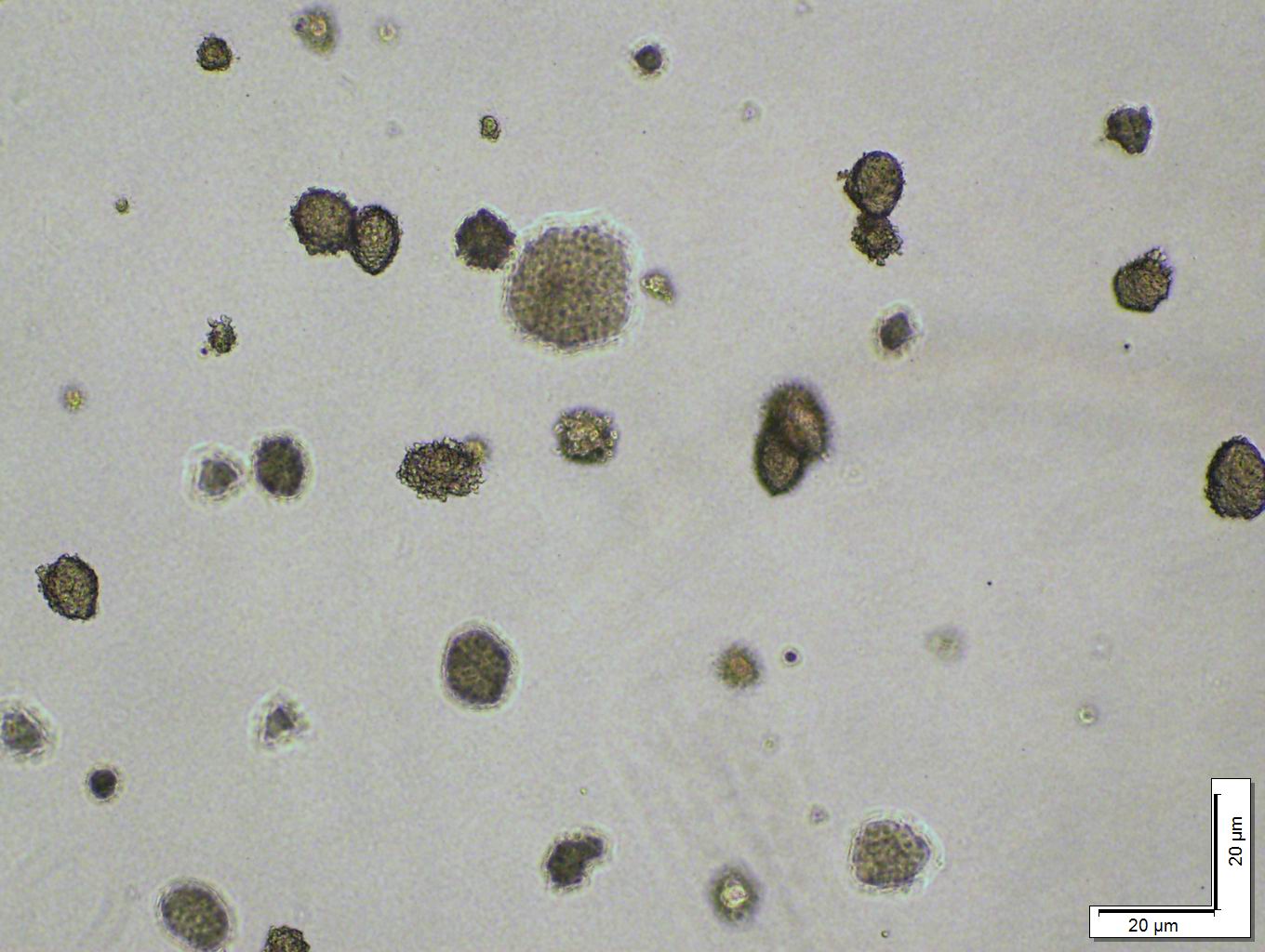

Supplement: Supplementary file 4 [file DataSheet_4.zip › fig 7d. A549 CSCs, shScrambled (1).jpg]

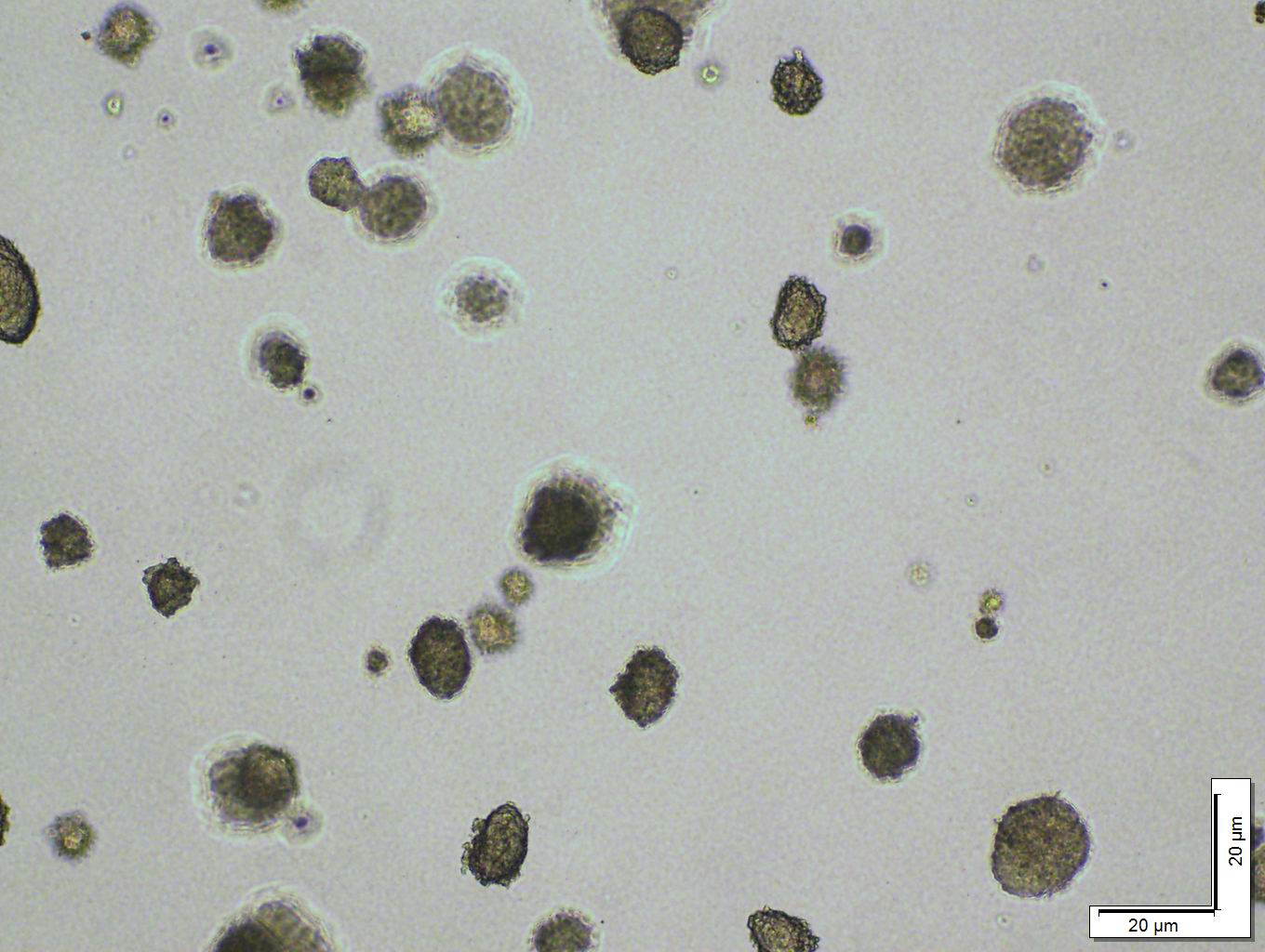

Supplement: Supplementary file 4 [file DataSheet_4.zip › fig 7d. A549 CSCs, shScrambled (2).jpg]

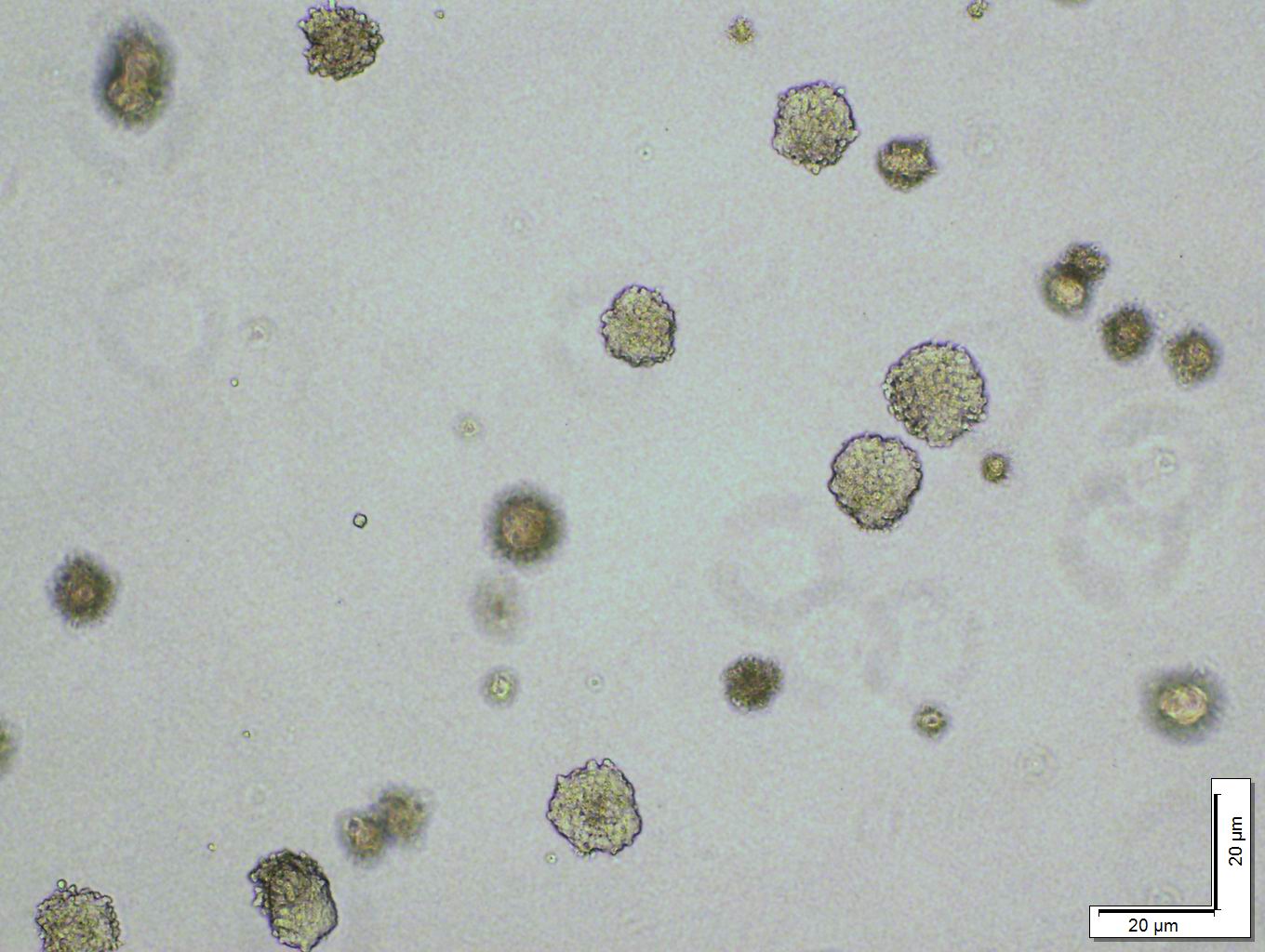

Supplement: Supplementary file 4 [file DataSheet_4.zip › fig 7d. A549 CSCs, shScrambled (3).jpg]

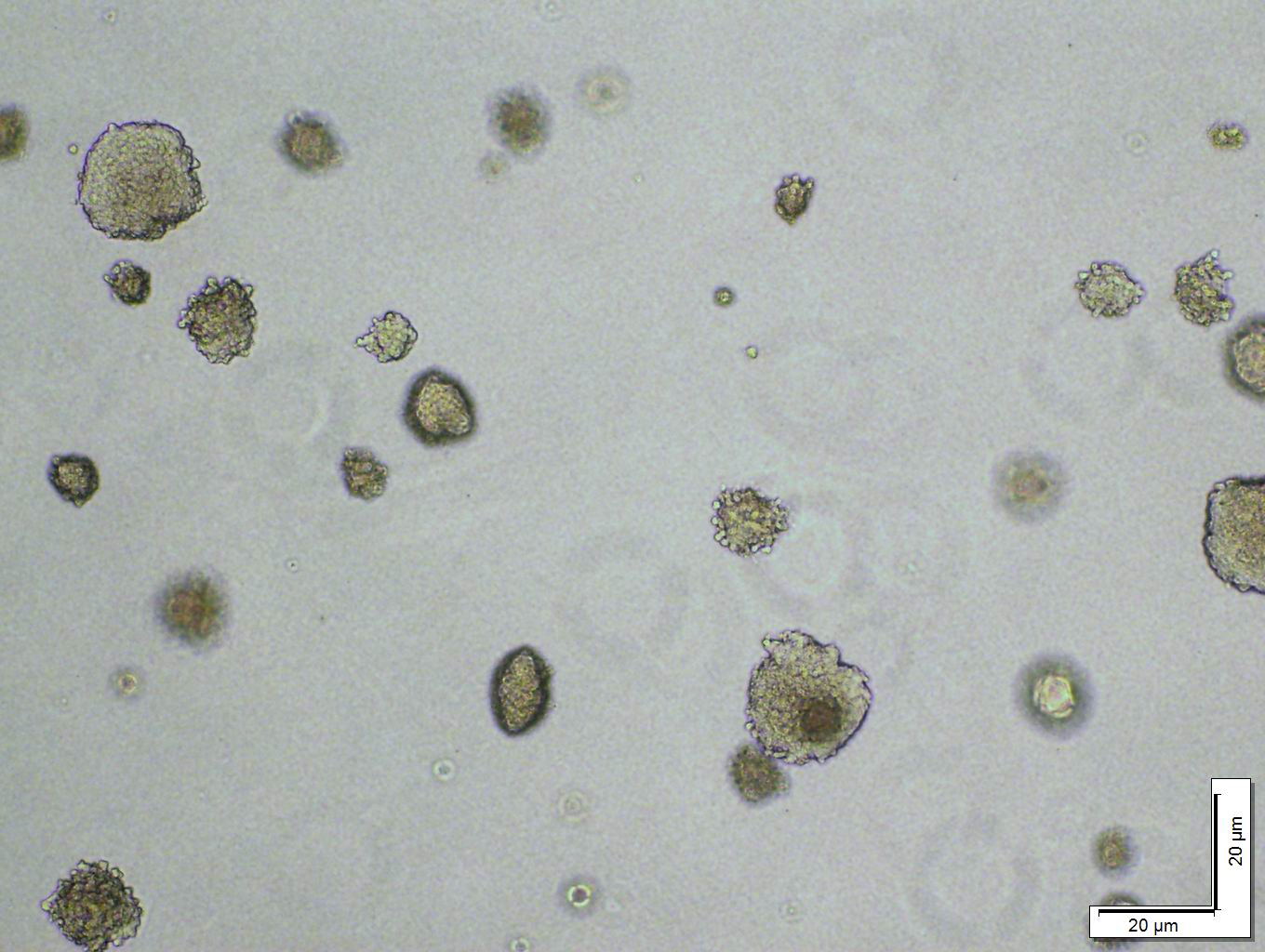

Supplement: Supplementary file 4 [file DataSheet_4.zip › fig 7d. A549 CSCs, shScrambled (4).jpg]

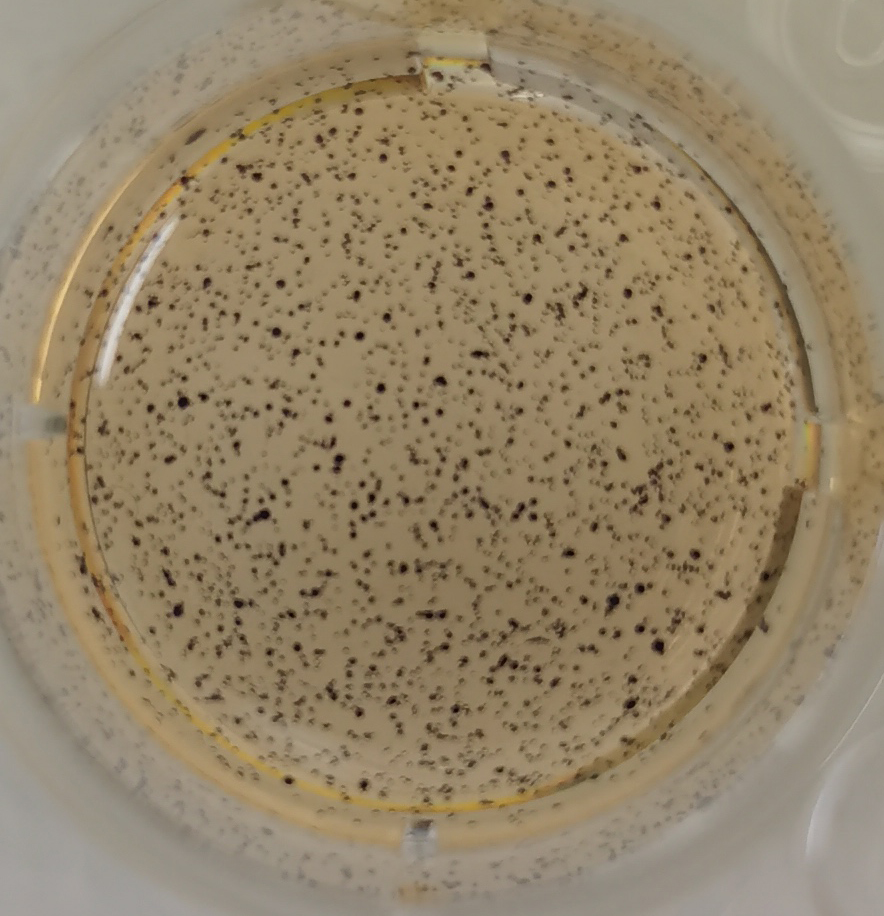

Supplement: Supplementary file 4 [file DataSheet_4.zip › fig 7d. A549 CSCs, shScrambled (5).jpg]

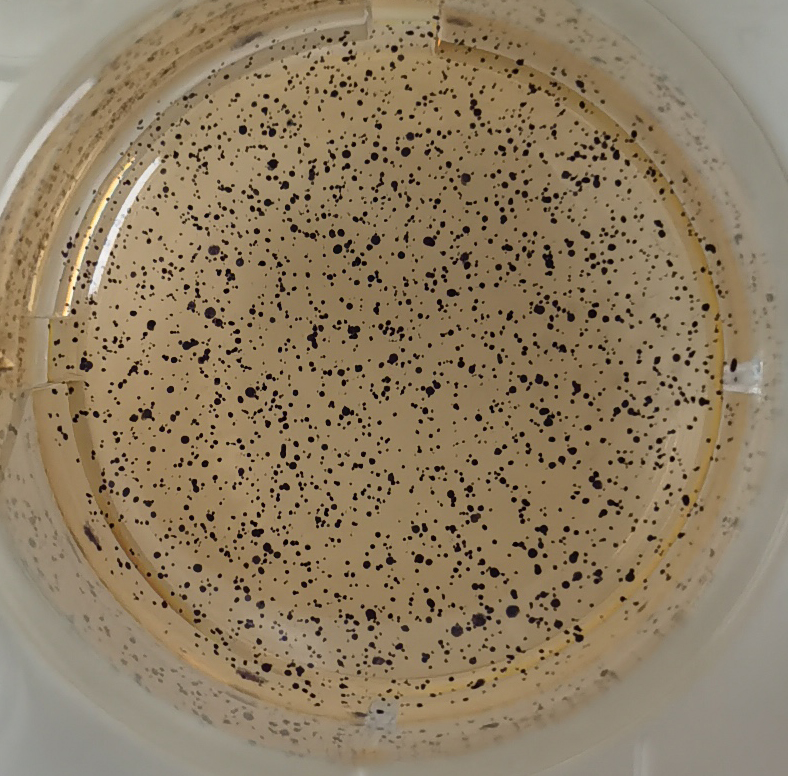

Supplement: Supplementary file 4 [file DataSheet_4.zip › fig 7d. A549 CSCs, shp53 (1).jpg]

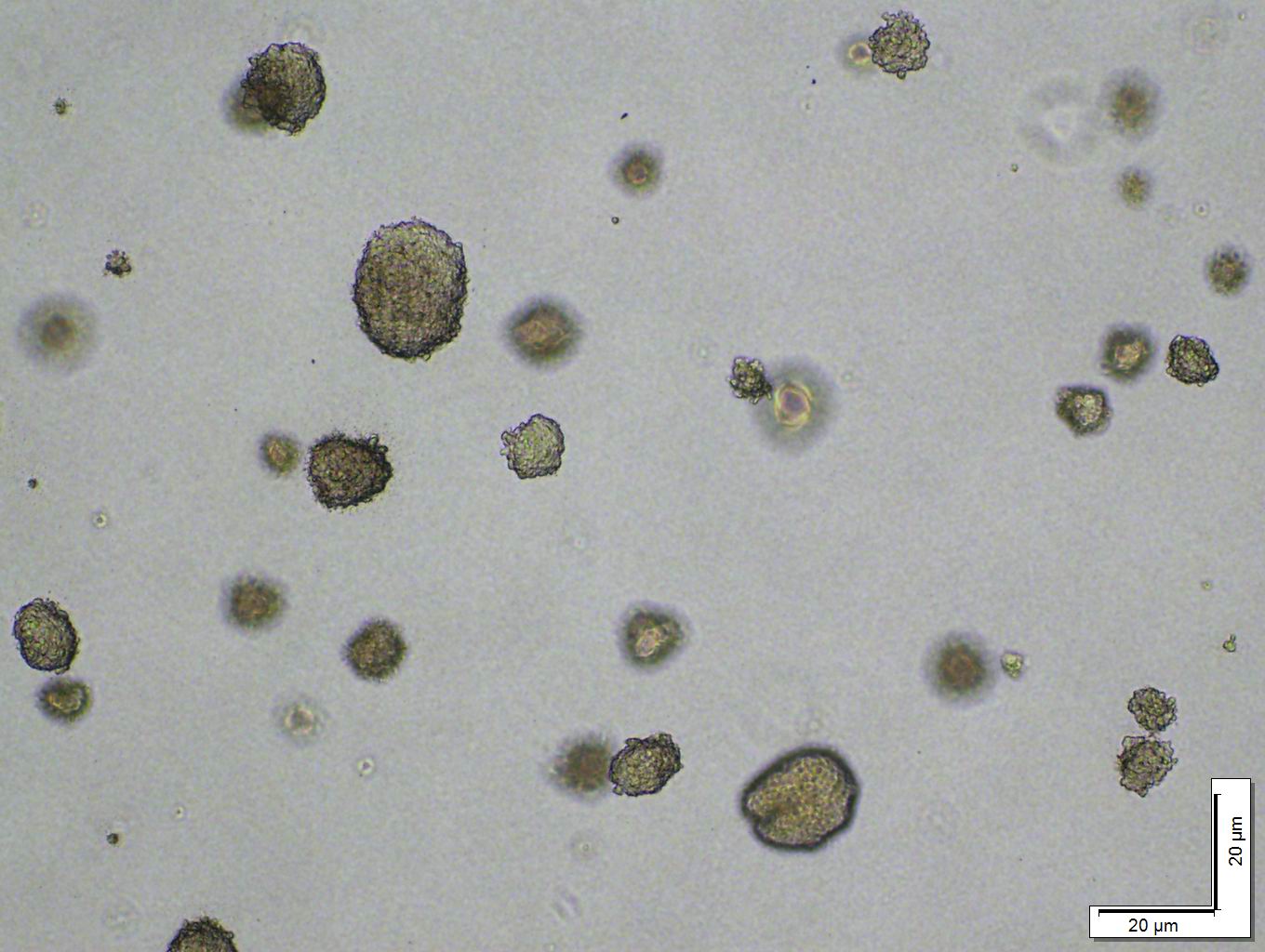

Supplement: Supplementary file 4 [file DataSheet_4.zip › fig 7d. A549 CSCs, shp53 (2).jpg]

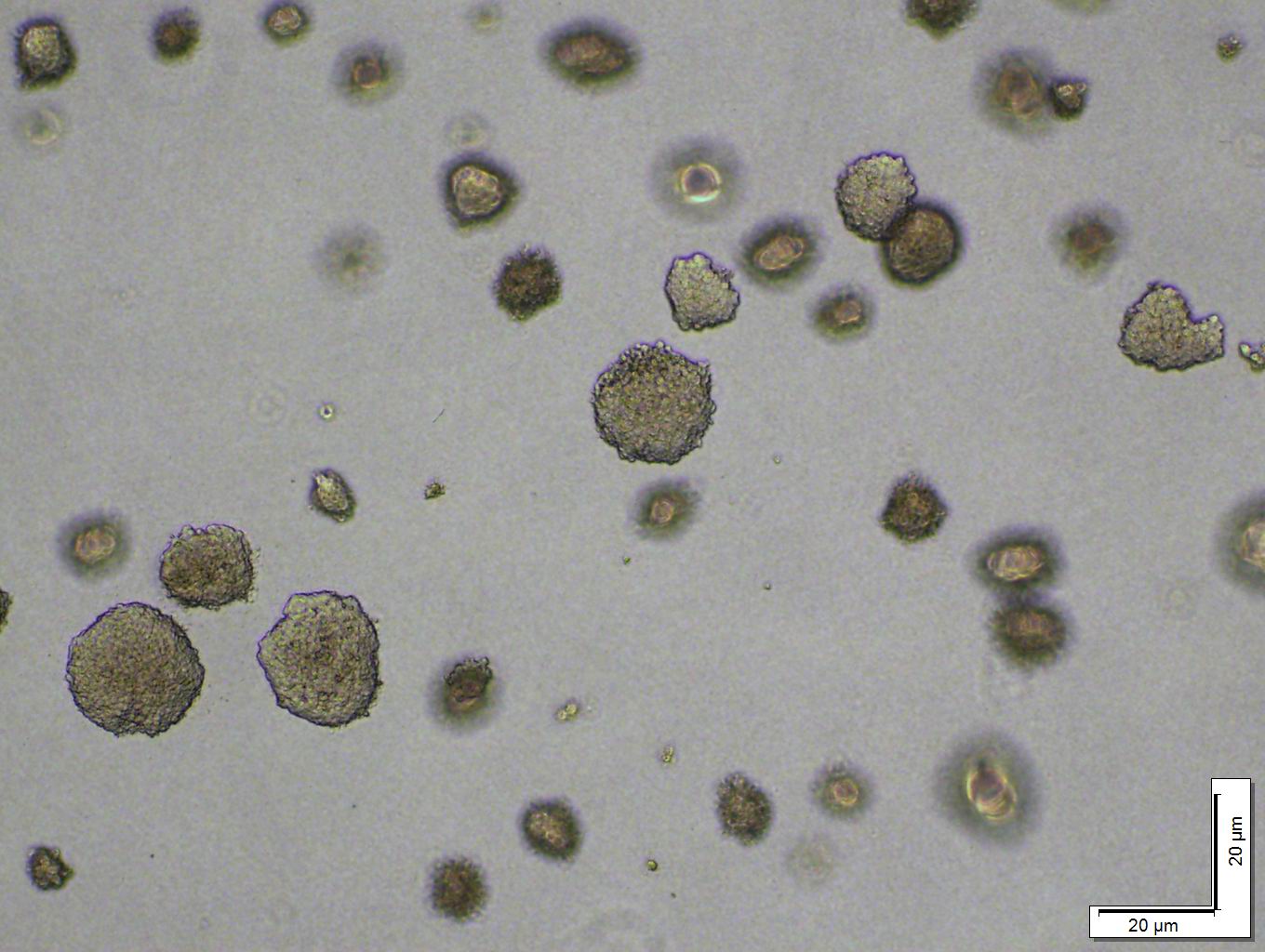

Supplement: Supplementary file 4 [file DataSheet_4.zip › fig 7d. A549 CSCs, shp53 (3).jpg]

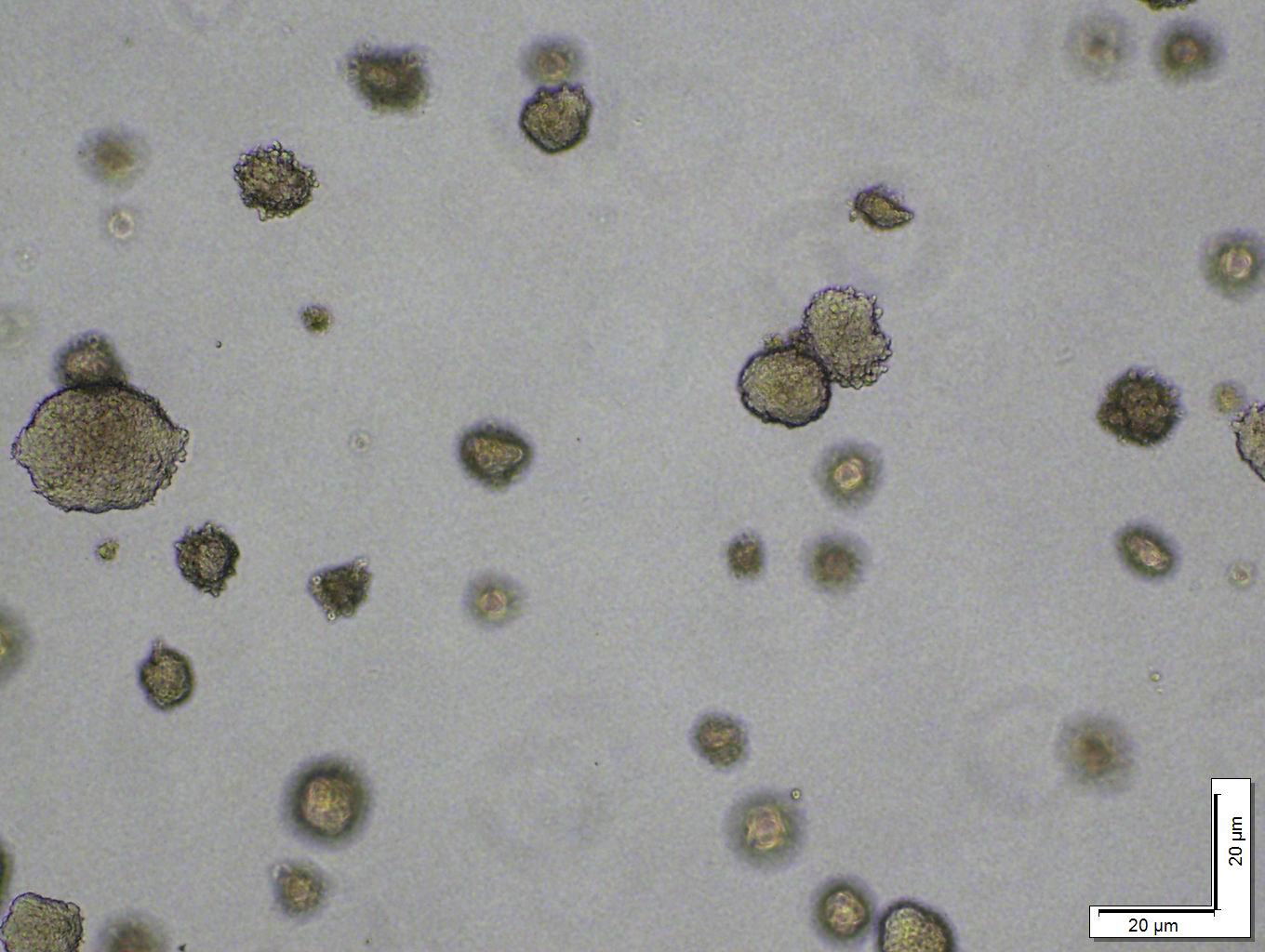

Supplement: Supplementary file 4 [file DataSheet_4.zip › fig 7d. A549 CSCs, shp53 (4).jpg]

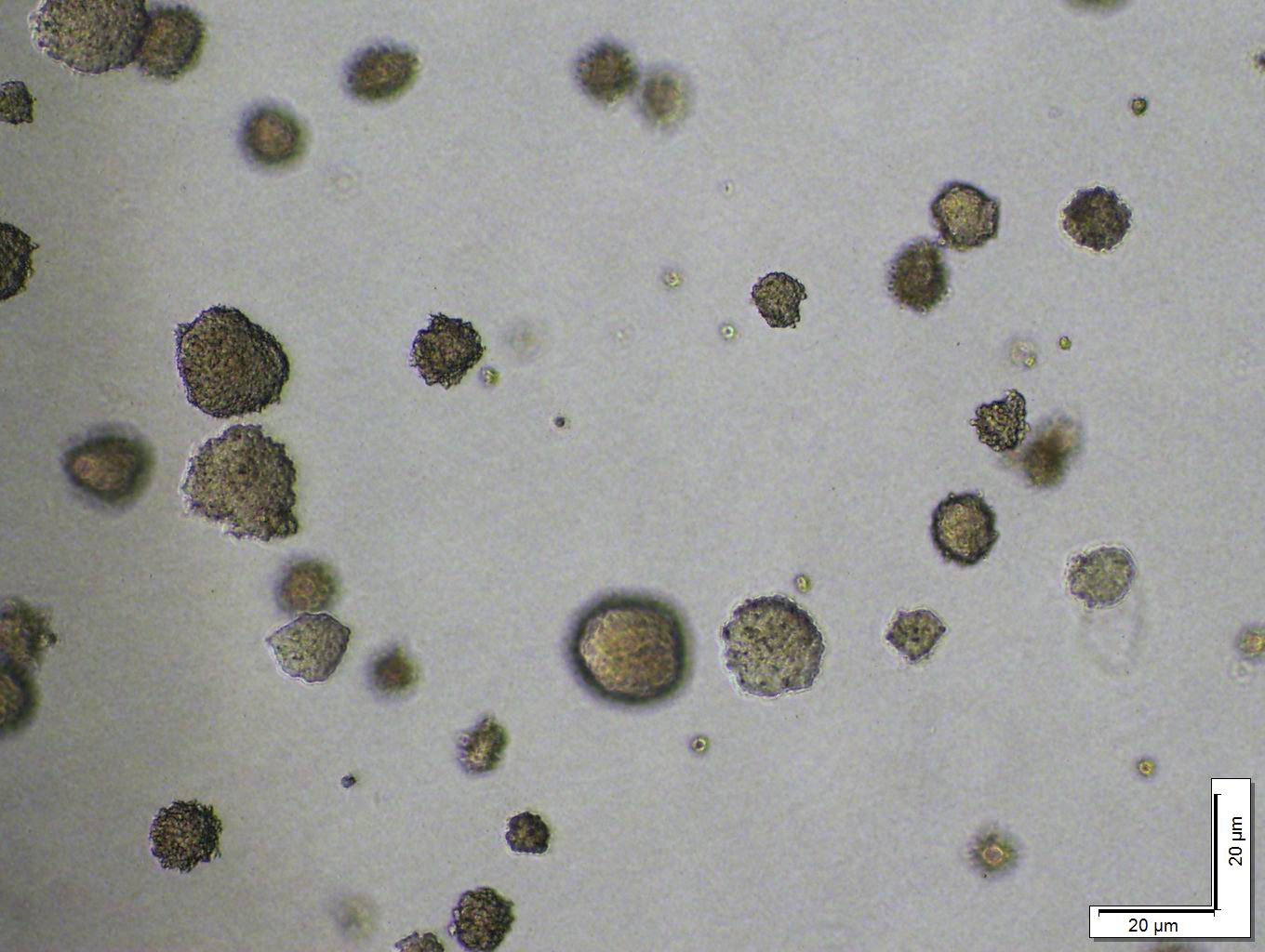

Supplement: Supplementary file 4 [file DataSheet_4.zip › fig 7d. A549 CSCs, shp53 (5).jpg]

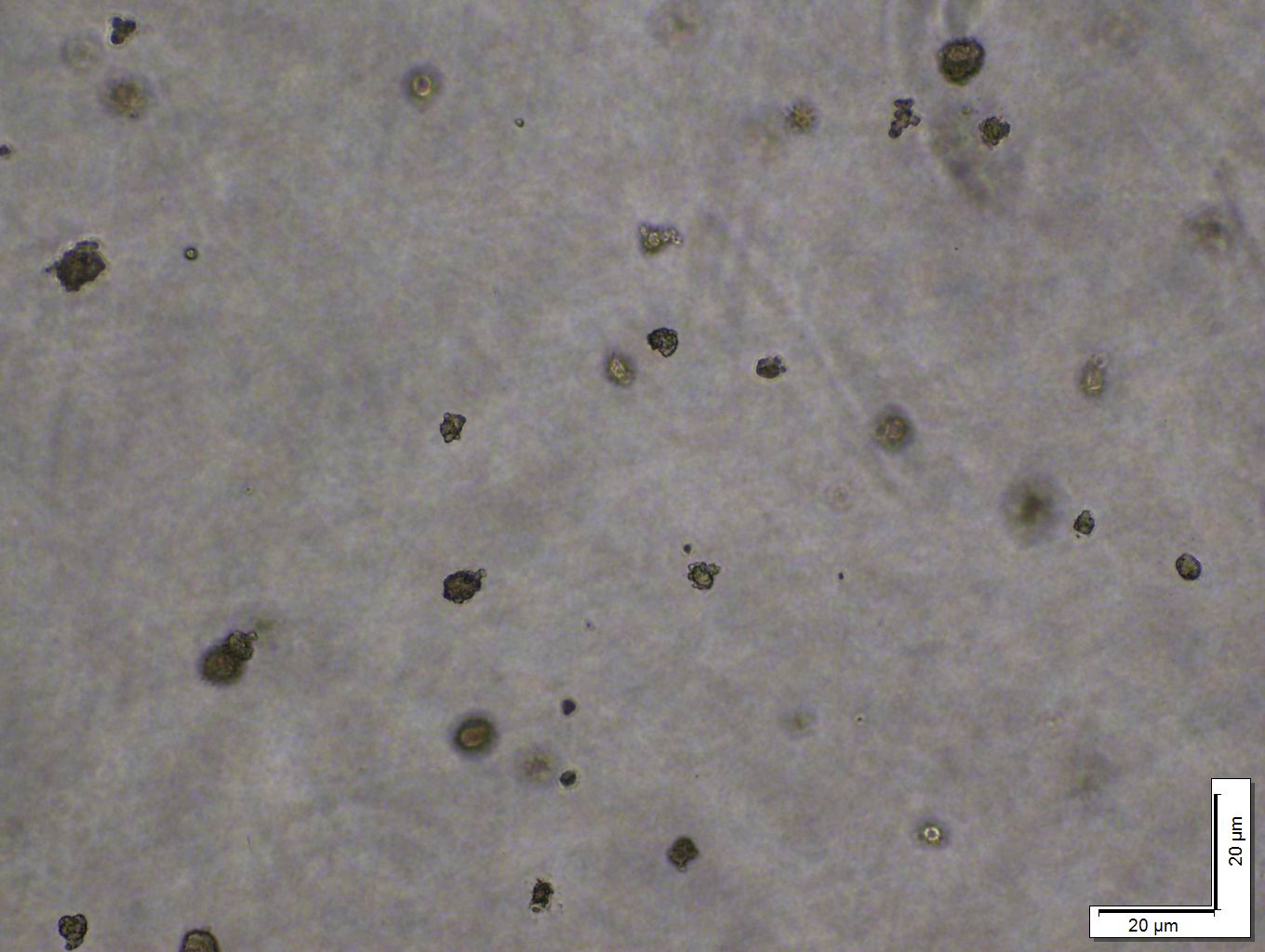

Supplement: Supplementary file 4 [file DataSheet_4.zip › fig 7d. A549 CSCs, shp53+PFT-A (1).jpg]

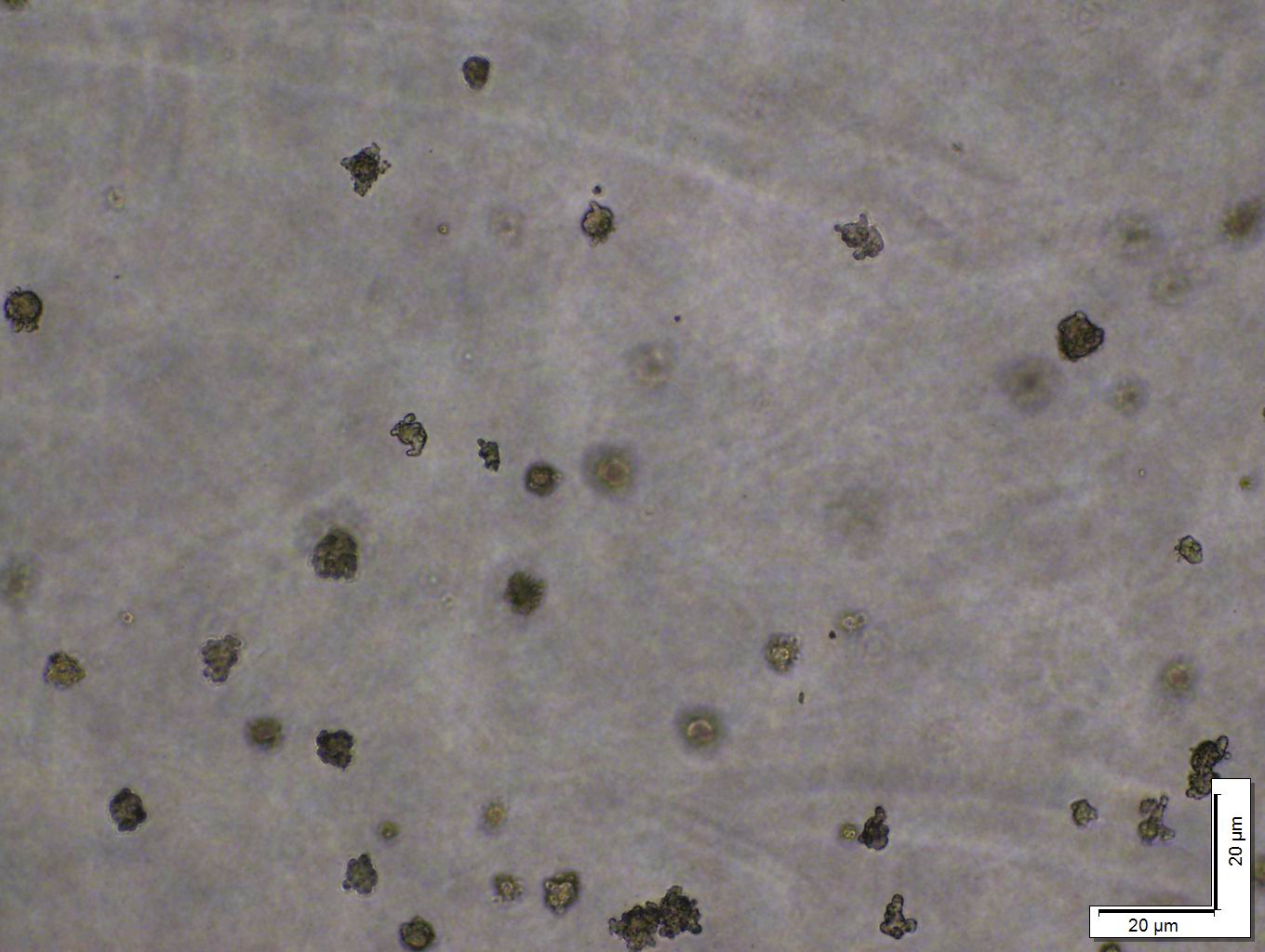

Supplement: Supplementary file 4 [file DataSheet_4.zip › fig 7d. A549 CSCs, shp53+PFT-A (2).jpg]

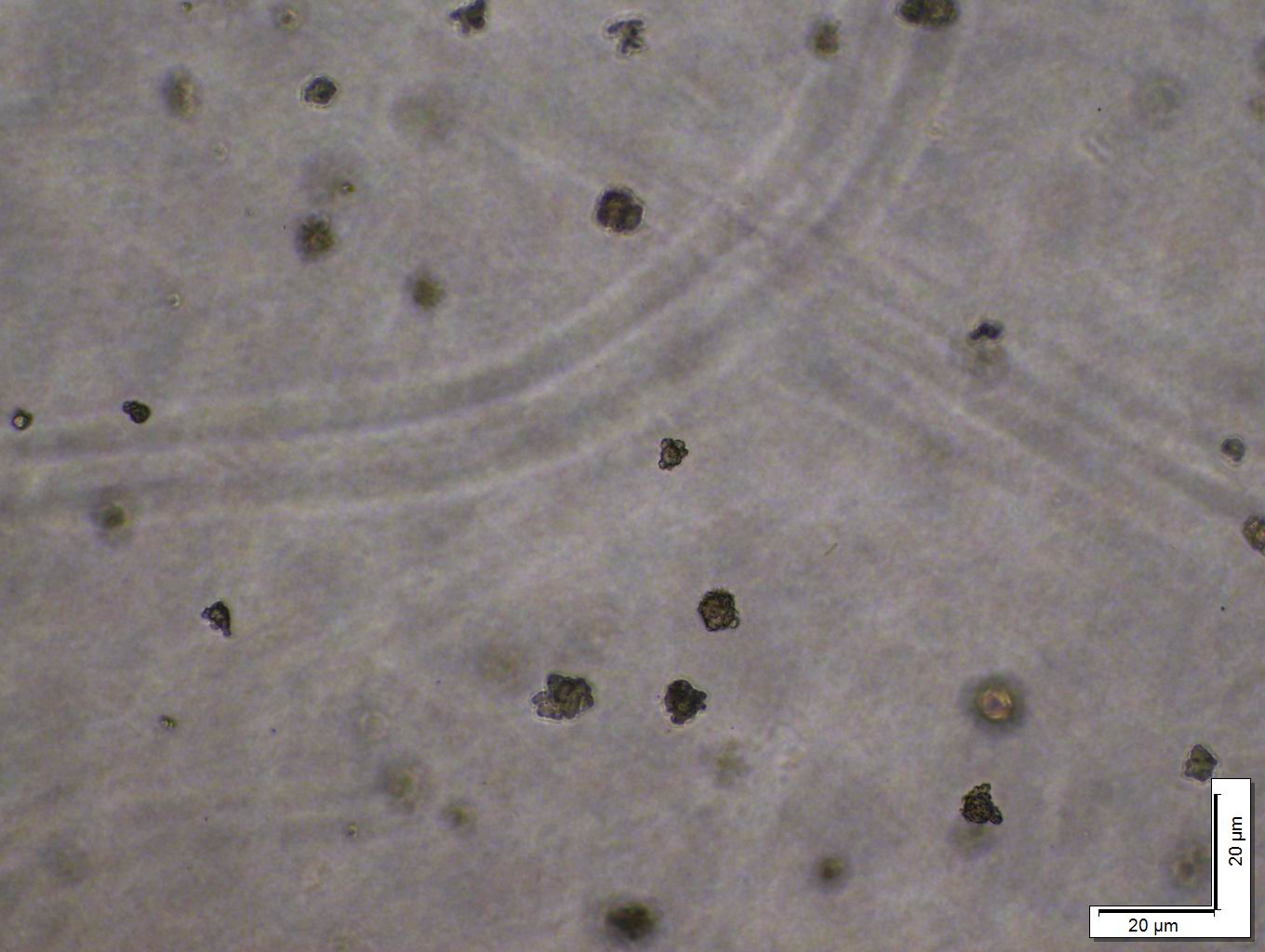

Supplement: Supplementary file 4 [file DataSheet_4.zip › fig 7d. A549 CSCs, shp53+PFT-A (3).jpg]

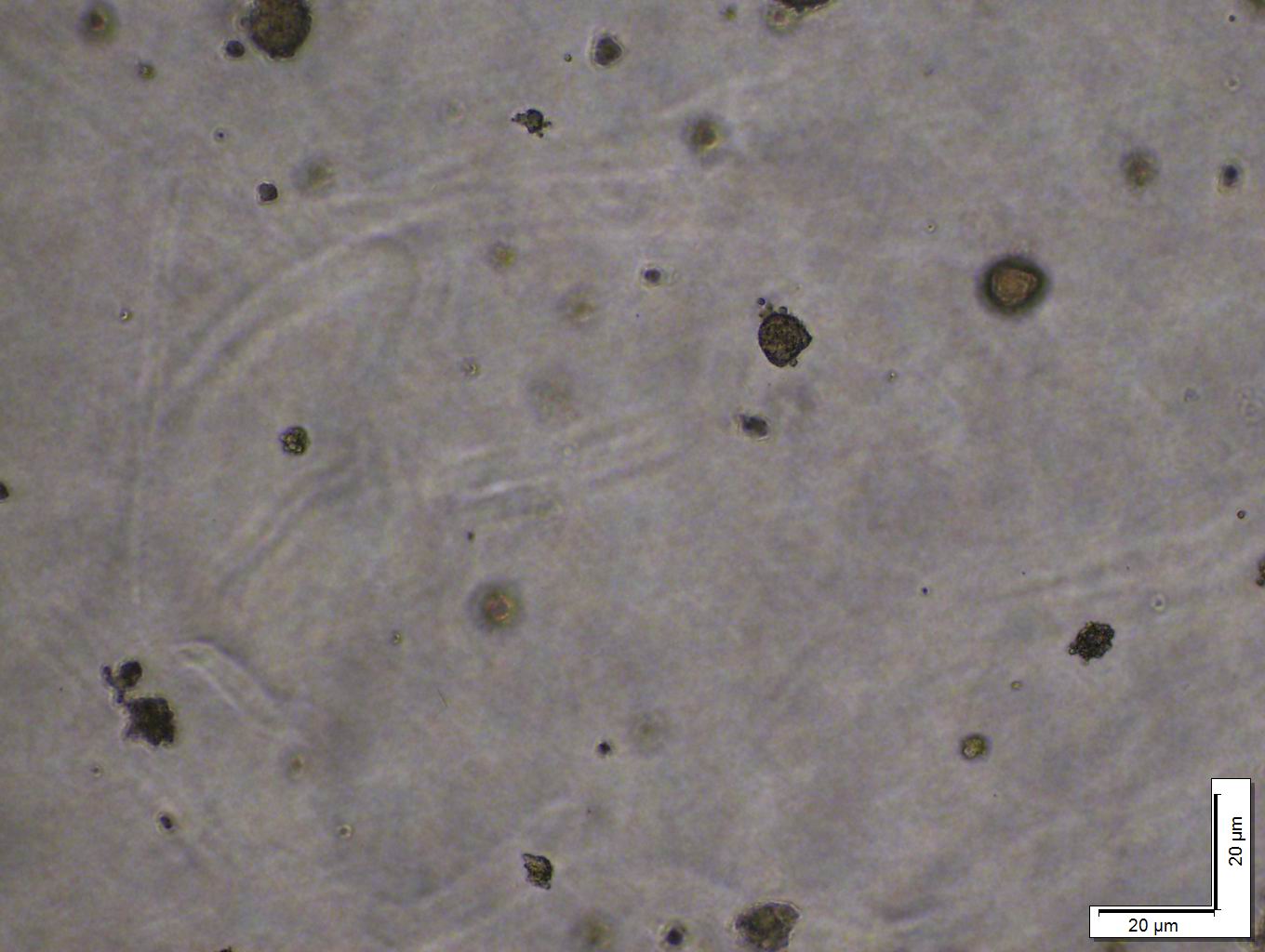

Supplement: Supplementary file 4 [file DataSheet_4.zip › fig 7d. A549 CSCs, shp53+PFT-A (4).jpg]

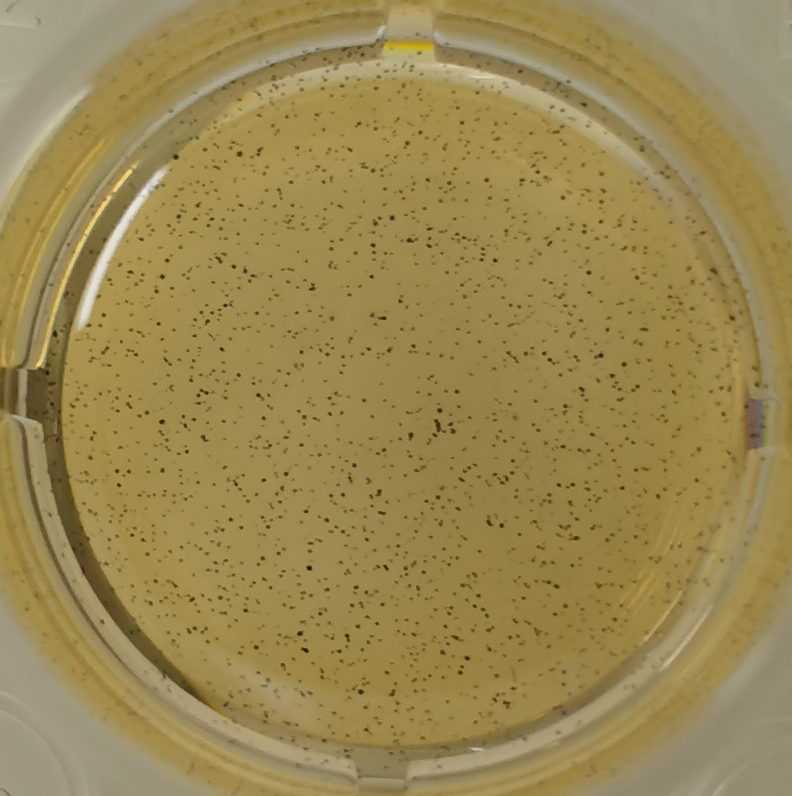

Supplement: Supplementary file 4 [file DataSheet_4.zip › fig 7d. A549 CSCs, shp53+PFT-A (5).jpg]

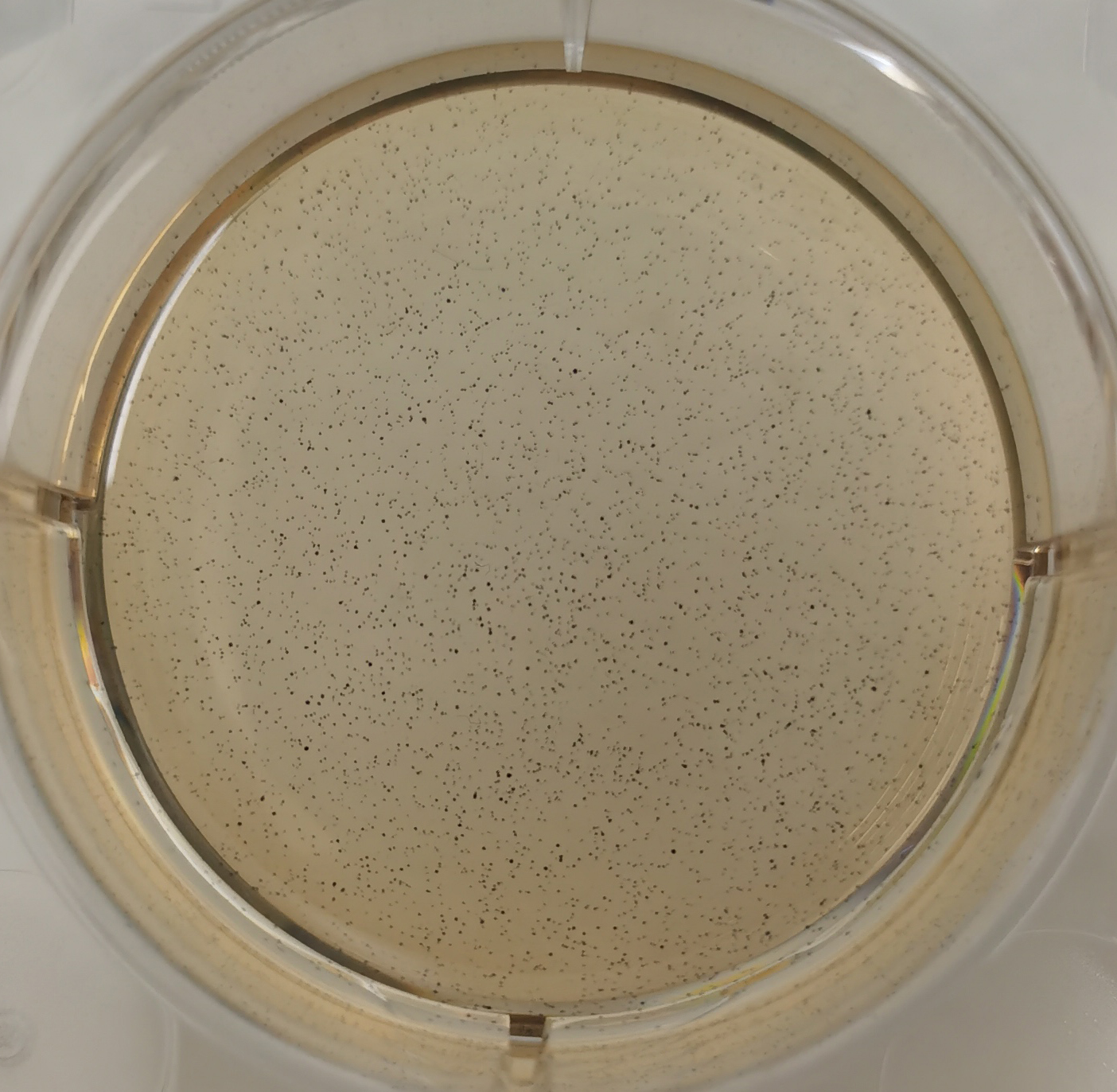

Supplement: Supplementary file 4 [file DataSheet_4.zip › fig 7d. PC-9, p53 (1).jpg]

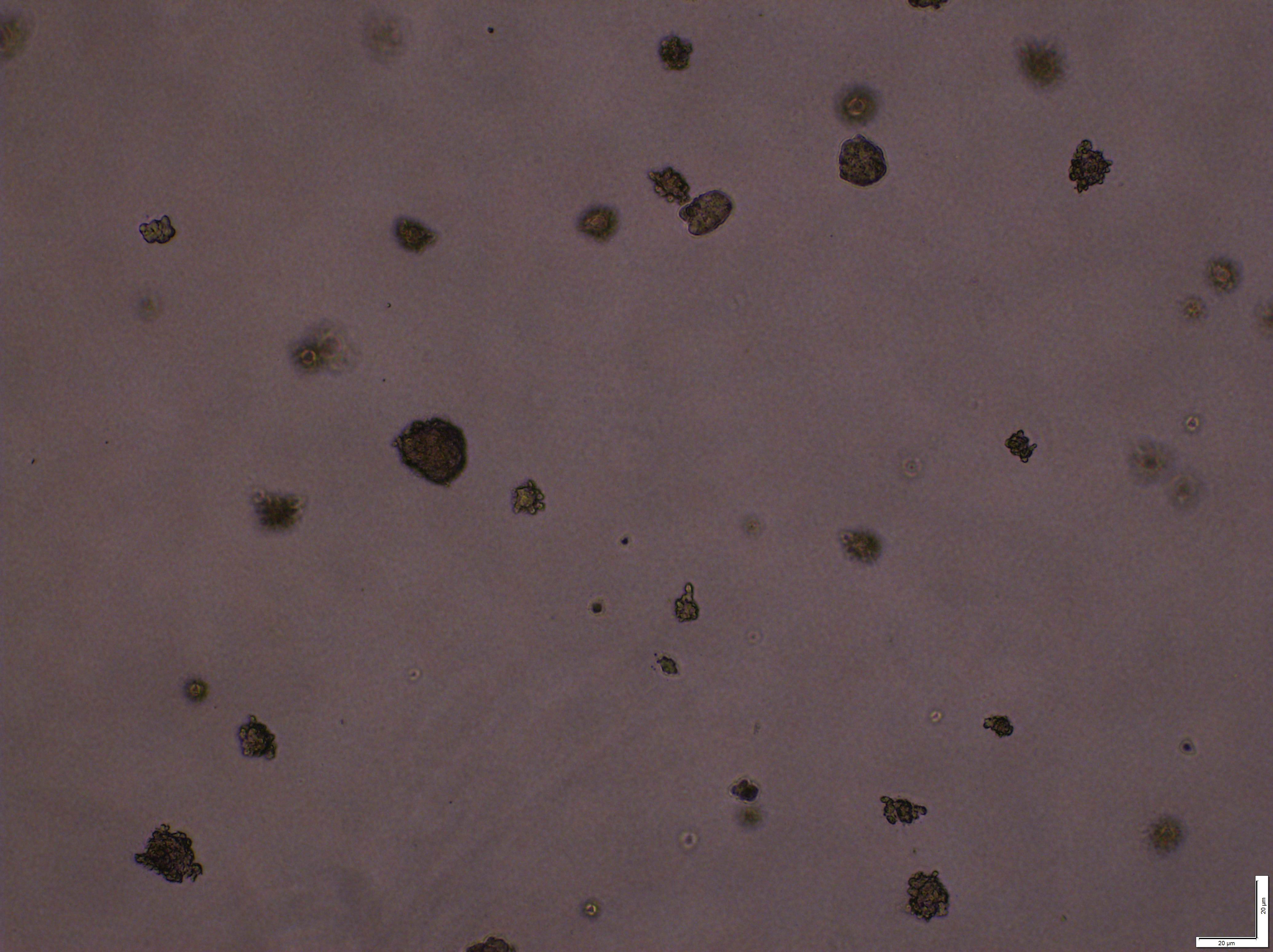

Supplement: Supplementary file 4 [file DataSheet_4.zip › fig 7d. PC-9, p53 (2).jpg]

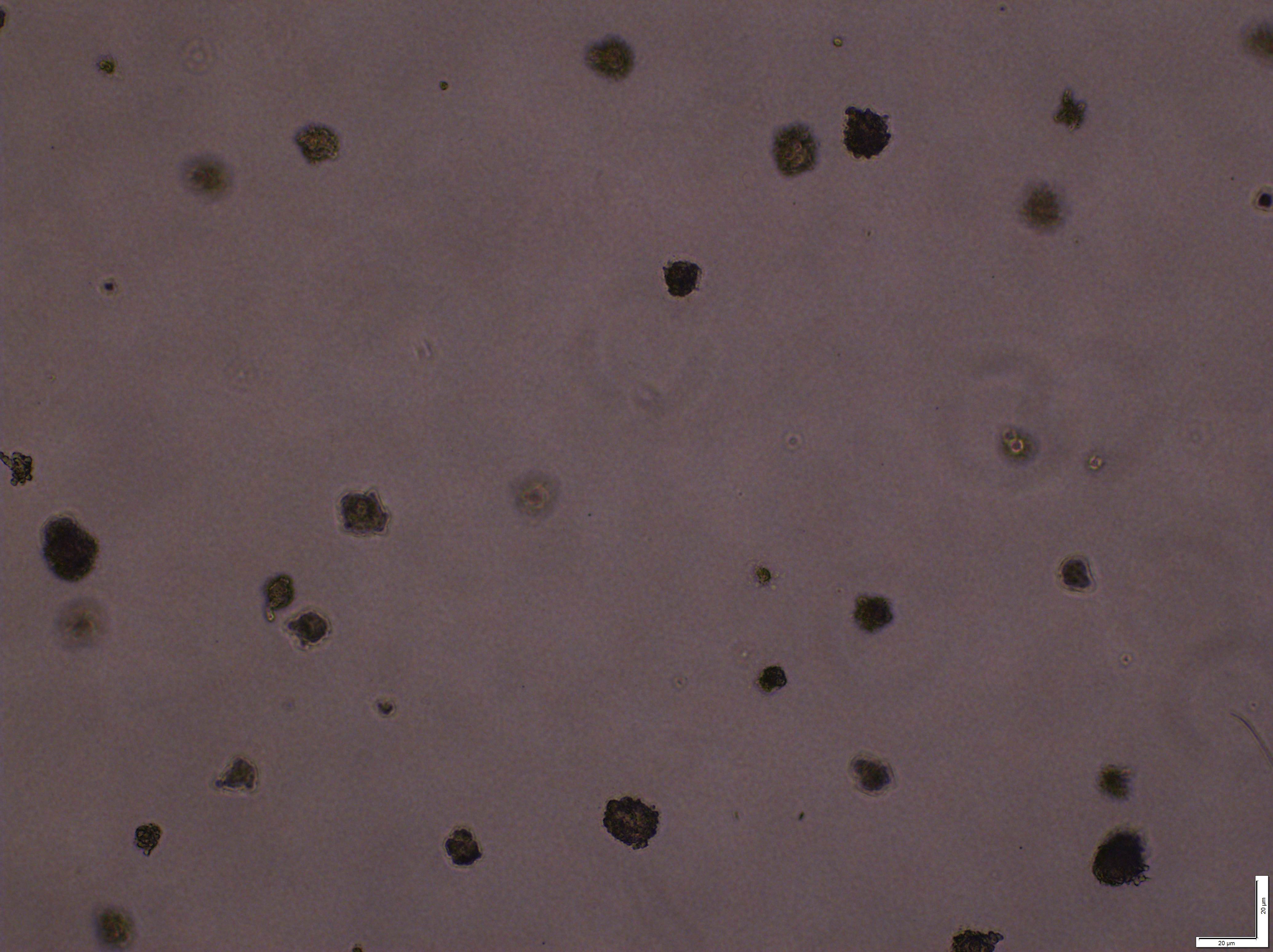

Supplement: Supplementary file 4 [file DataSheet_4.zip › fig 7d. PC-9, p53 (3).jpg]
