# Supplementary figures and images for: RNA Demethylase ALKBH5 Prevents Lung Cancer Progression by Regulating EMT and Stemness via Regulating p53 (part 2 of 2)
Source: Front Oncol. 2022 Apr 22;12:858694. doi: 10.3389/fonc.2022.858694 (PMC9076132; doi:10.3389/fonc.2022.858694)

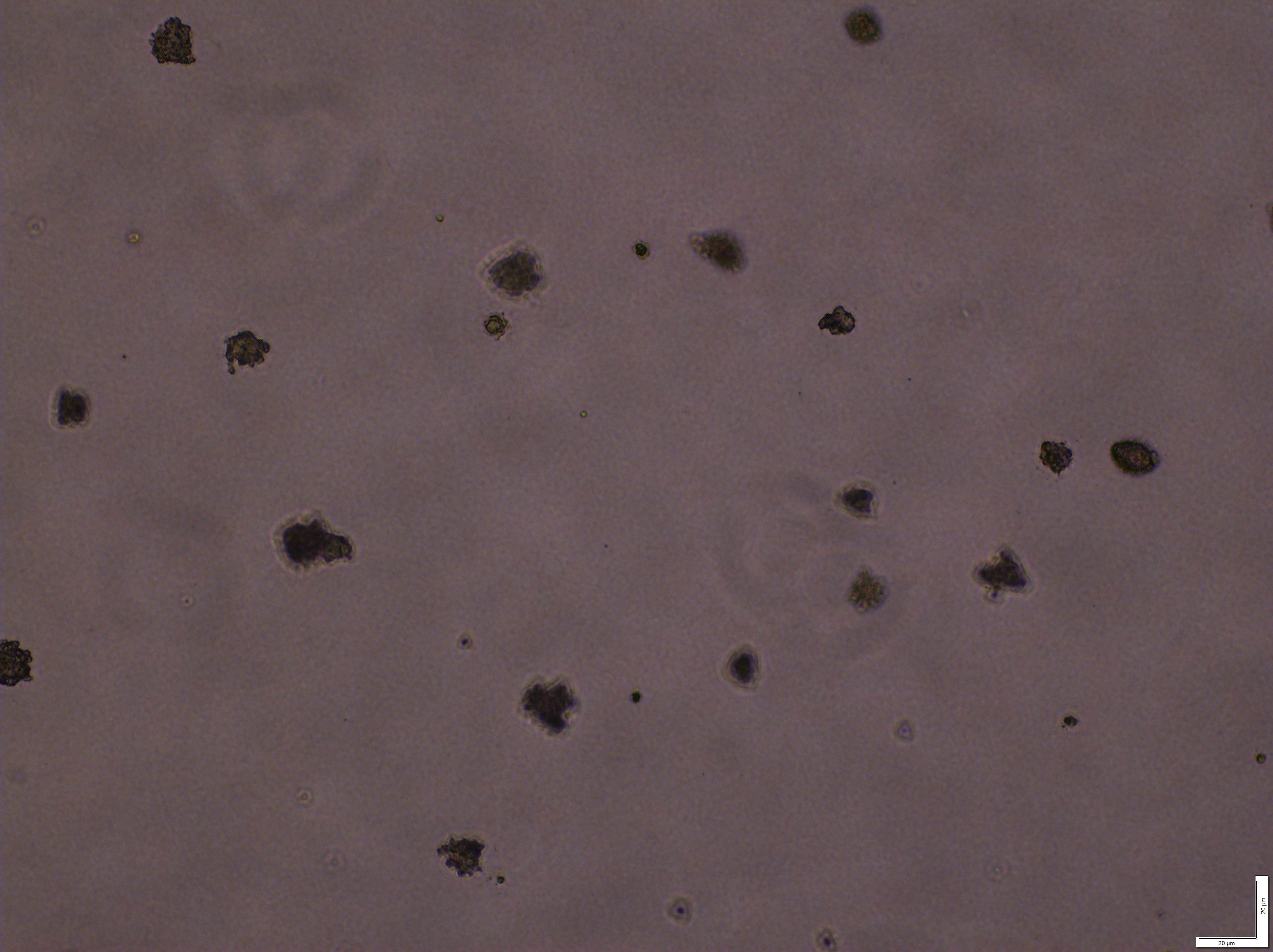

Supplement: Supplementary file 4 [file DataSheet_4.zip › fig 7d. PC-9, p53 (4).jpg]

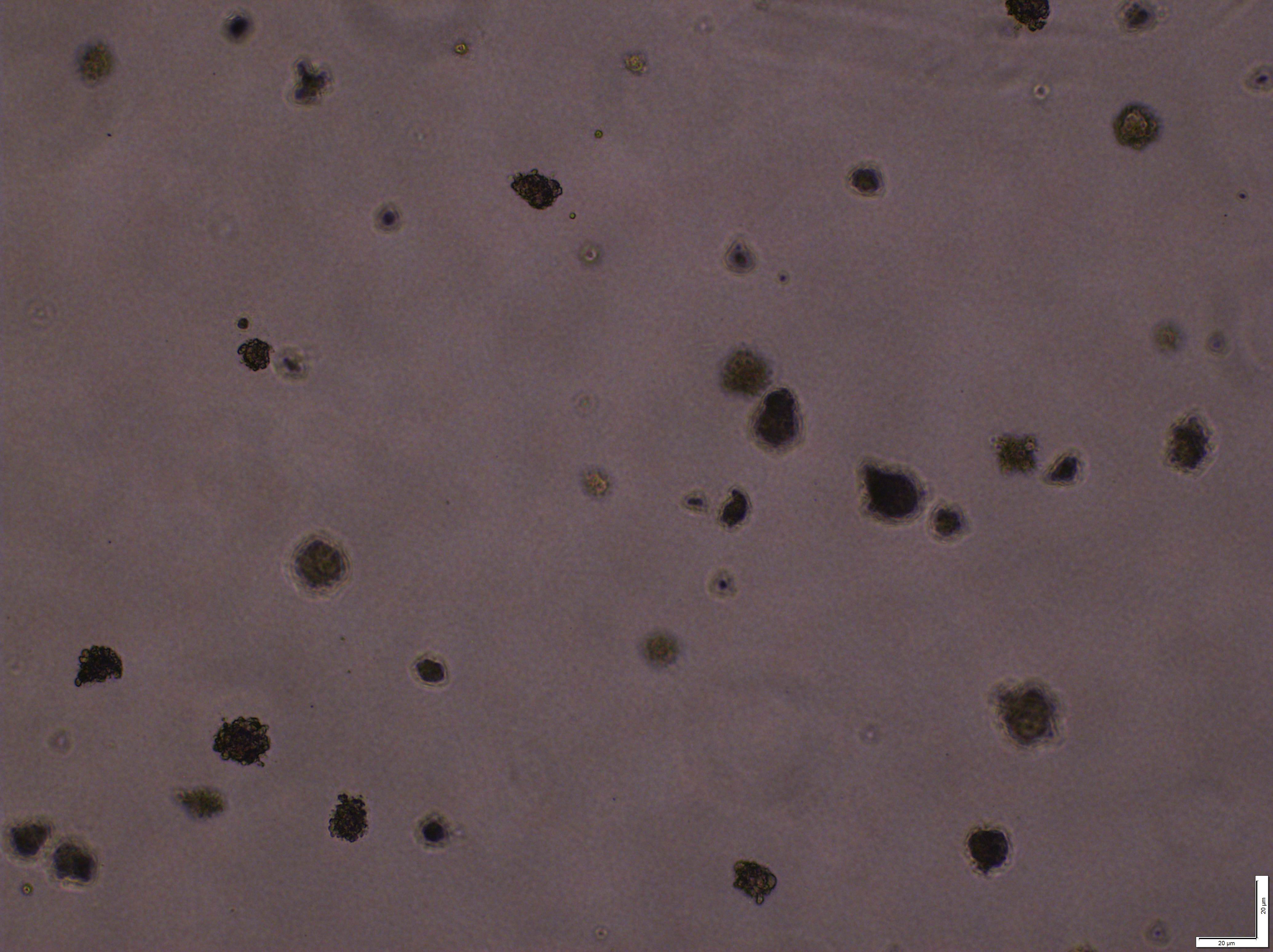

Supplement: Supplementary file 4 [file DataSheet_4.zip › fig 7d. PC-9, p53 (5).jpg]

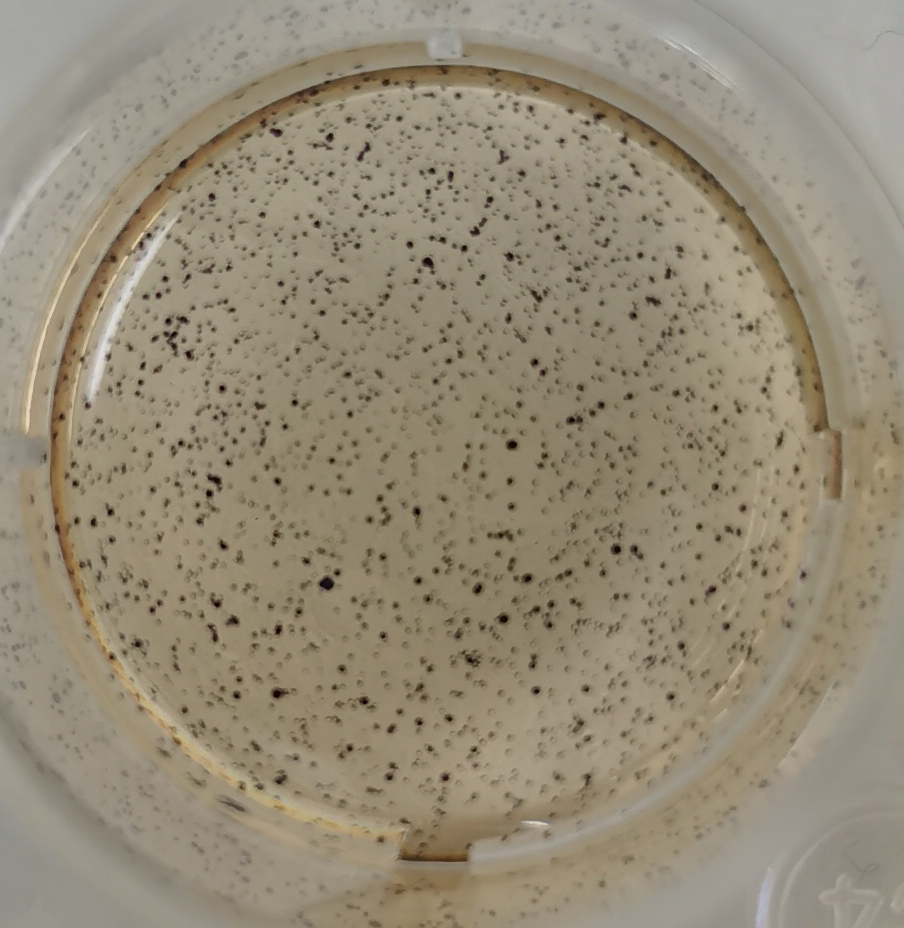

Supplement: Supplementary file 4 [file DataSheet_4.zip › fig 7d. PC-9, p53+PFT-A (1).jpg]

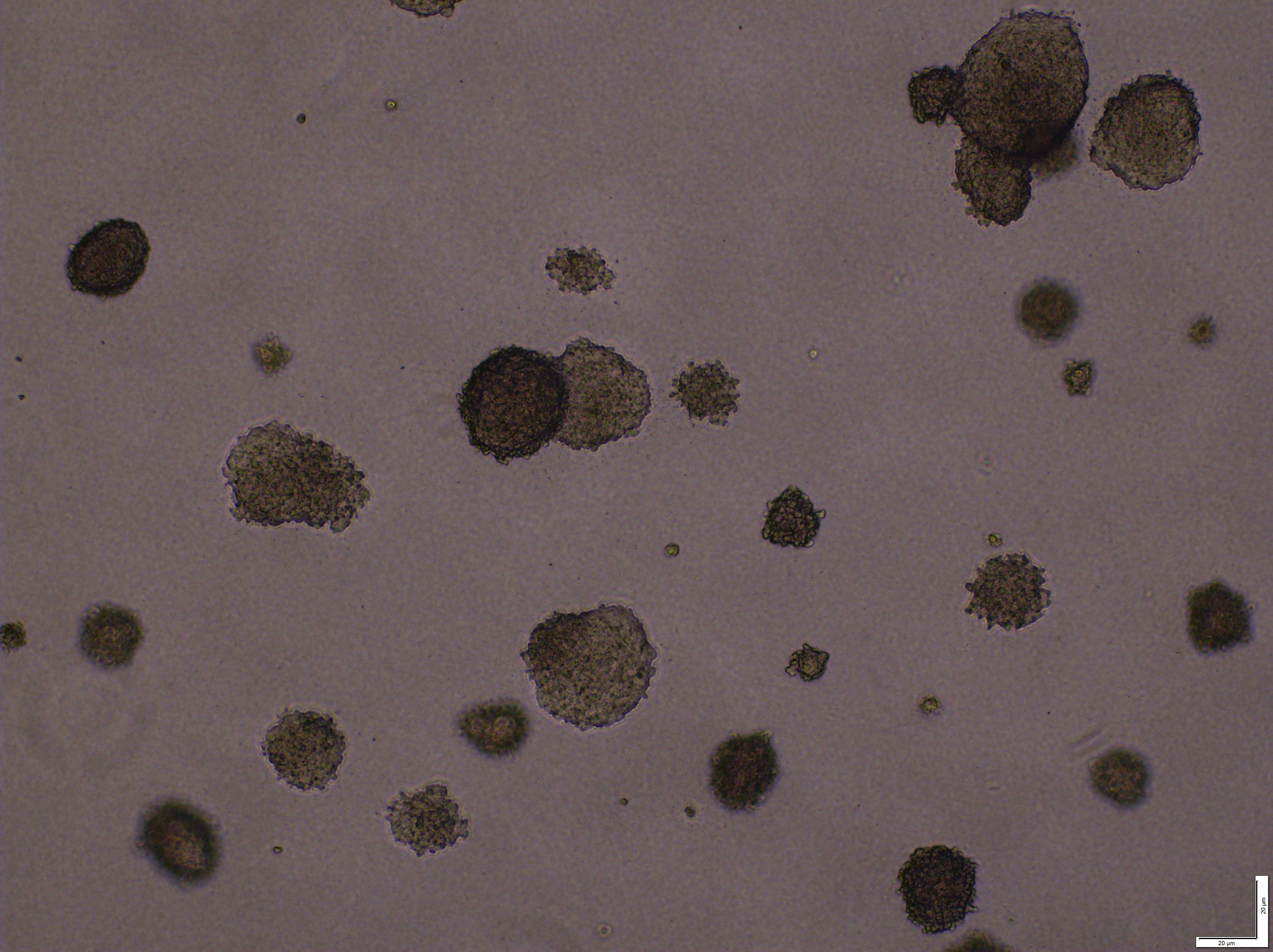

Supplement: Supplementary file 4 [file DataSheet_4.zip › fig 7d. PC-9, p53+PFT-A (2).jpg]

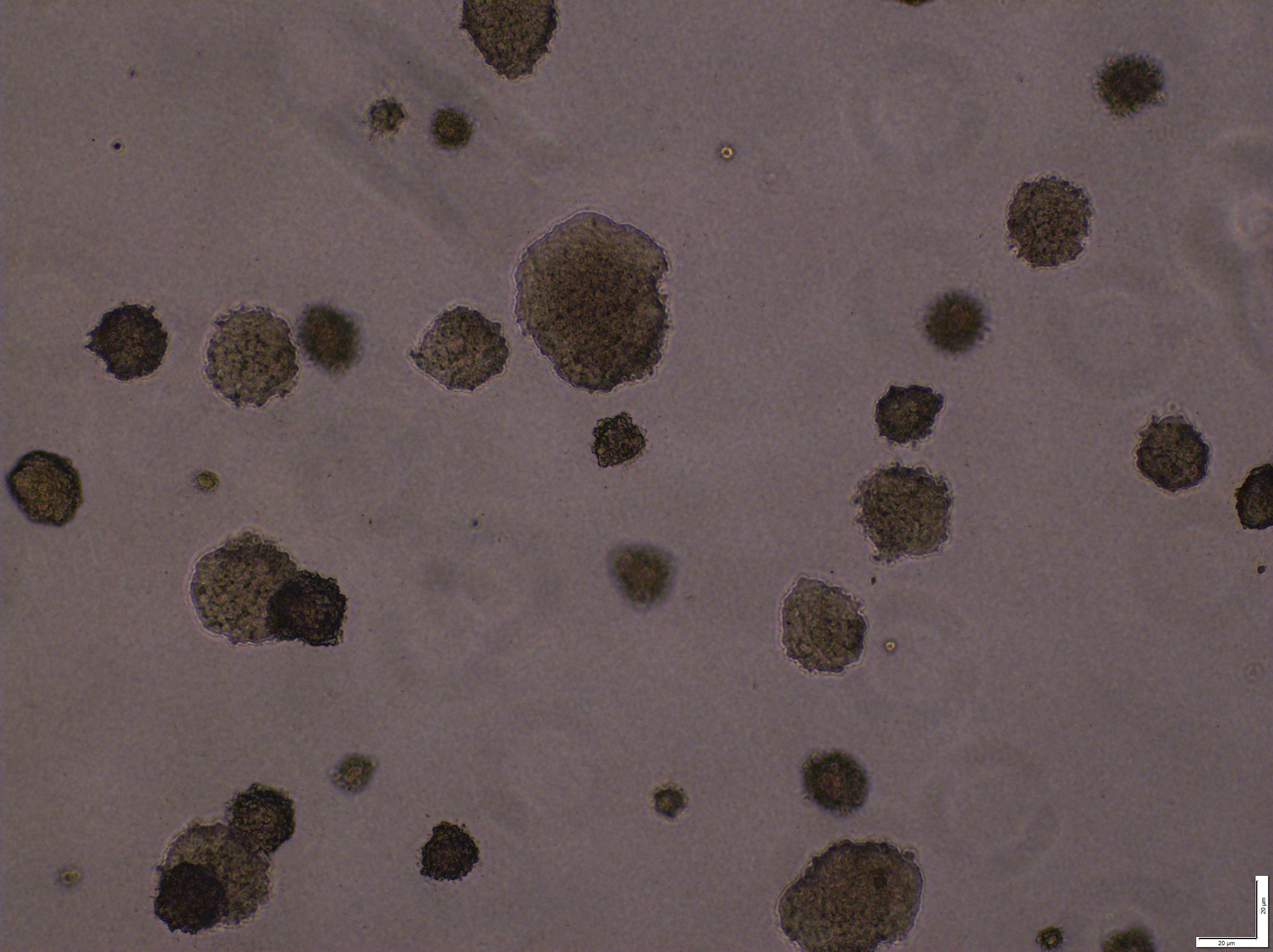

Supplement: Supplementary file 4 [file DataSheet_4.zip › fig 7d. PC-9, p53+PFT-A (3).jpg]

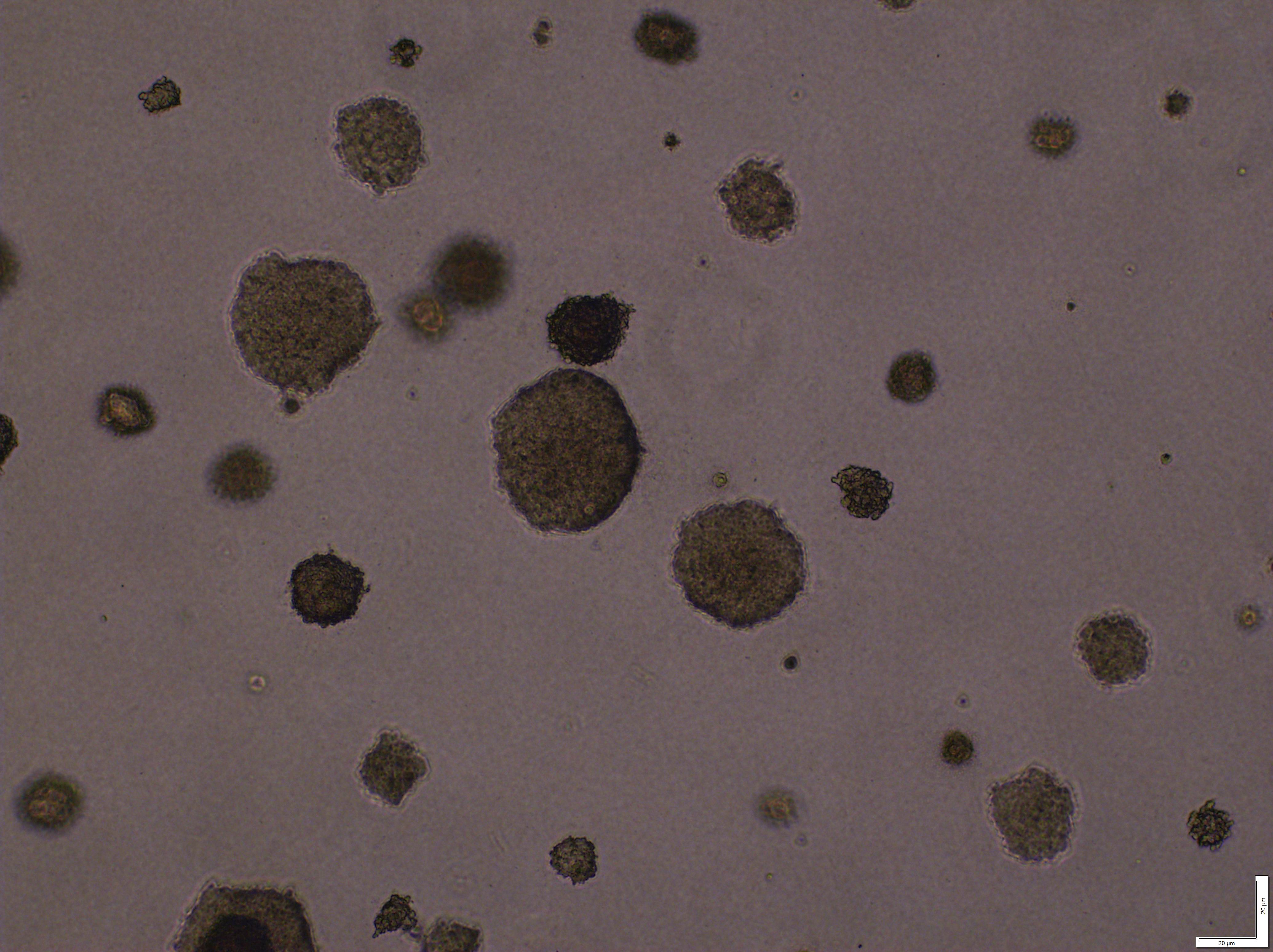

Supplement: Supplementary file 4 [file DataSheet_4.zip › fig 7d. PC-9, p53+PFT-A (4).jpg]

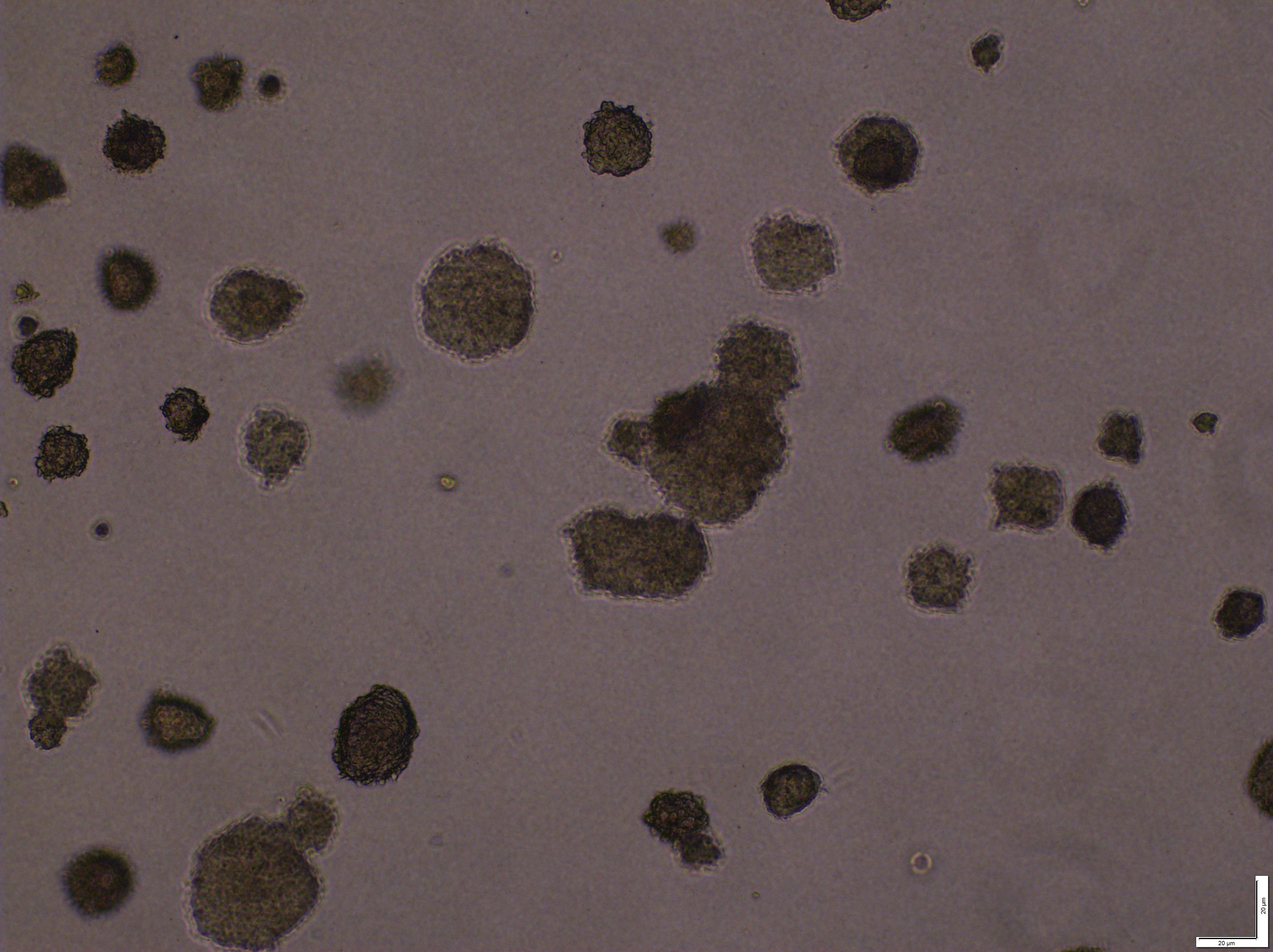

Supplement: Supplementary file 4 [file DataSheet_4.zip › fig 7d. PC-9, p53+PFT-A (5).jpg]

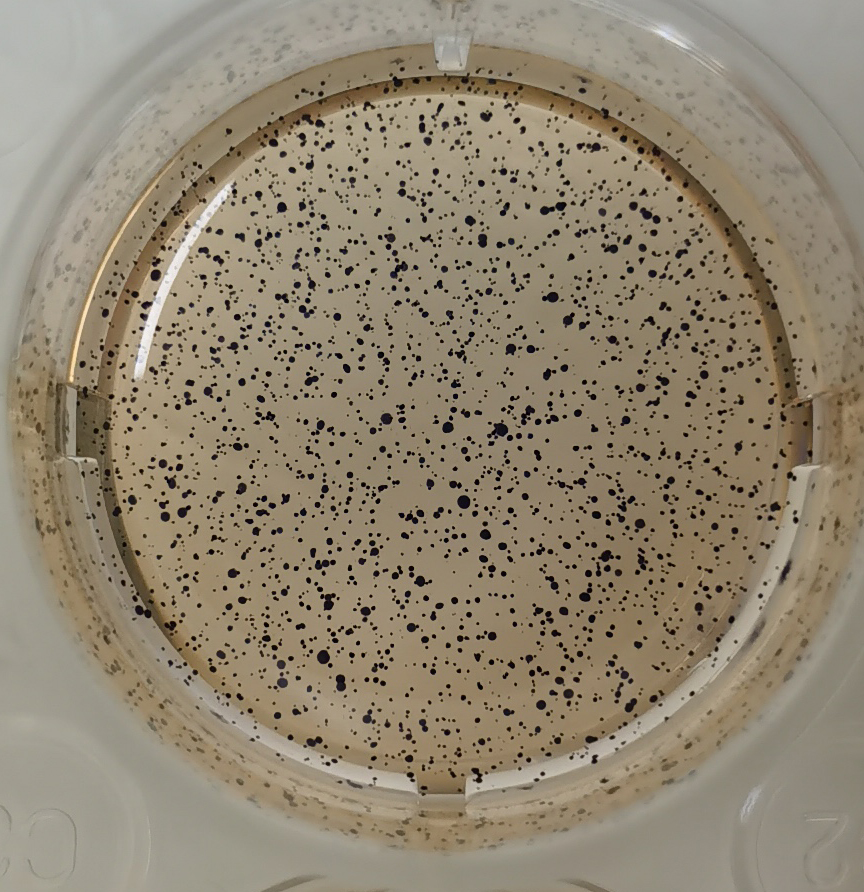

Supplement: Supplementary file 4 [file DataSheet_4.zip › fig 7d. PC-9, vector (1).jpg]

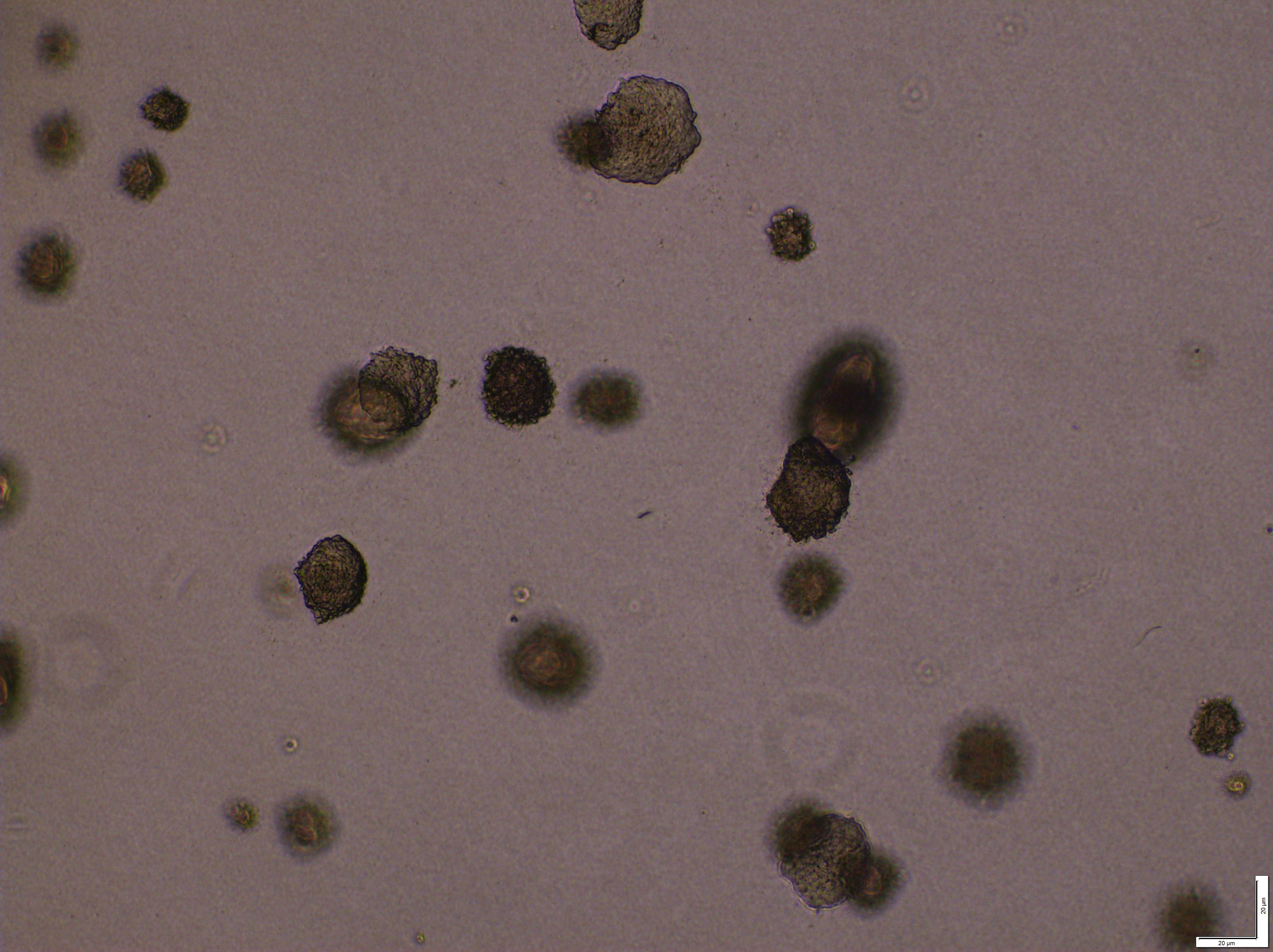

Supplement: Supplementary file 4 [file DataSheet_4.zip › fig 7d. PC-9, vector (2).jpg]

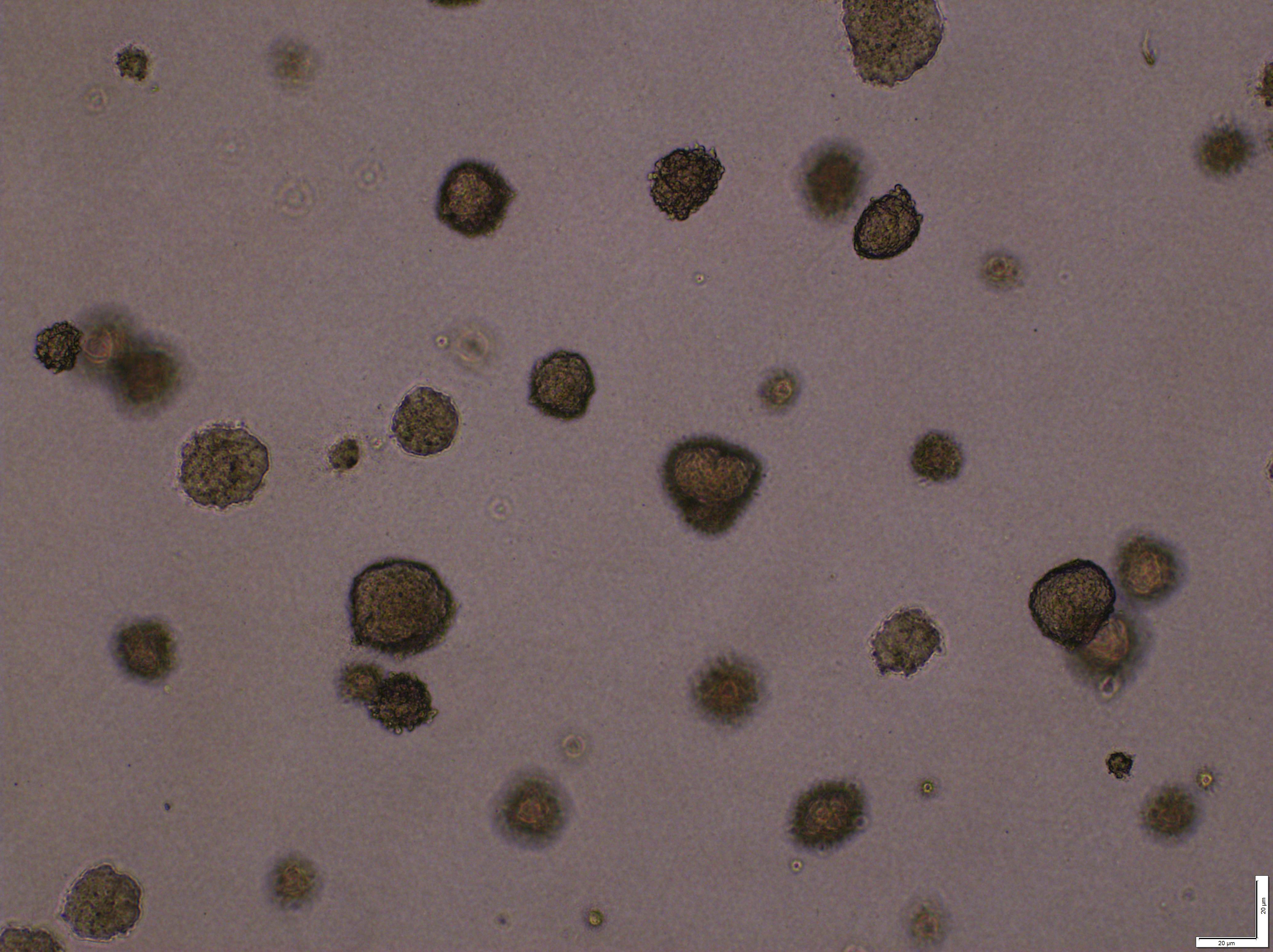

Supplement: Supplementary file 4 [file DataSheet_4.zip › fig 7d. PC-9, vector (3).jpg]

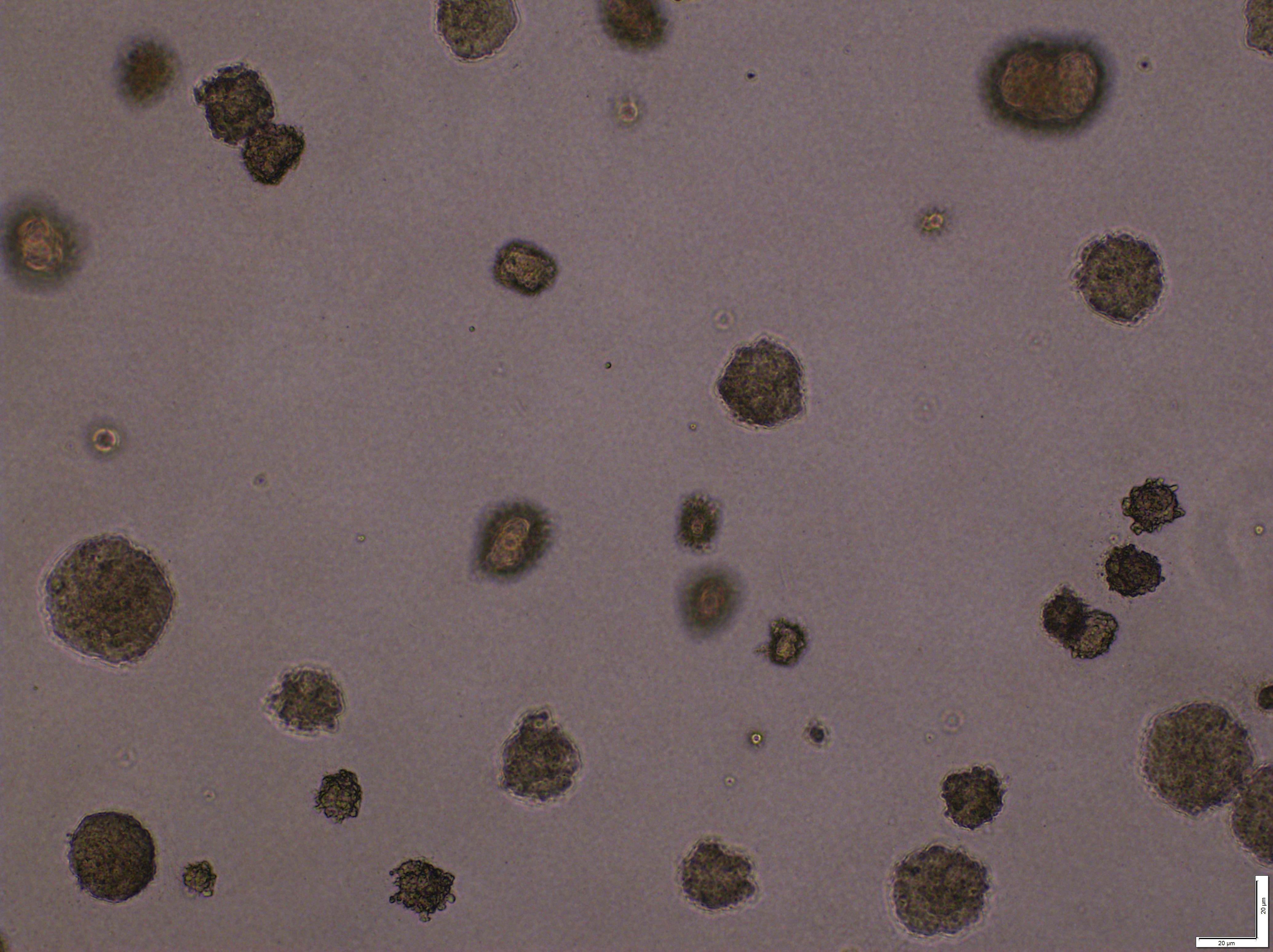

Supplement: Supplementary file 4 [file DataSheet_4.zip › fig 7d. PC-9, vector (4).jpg]

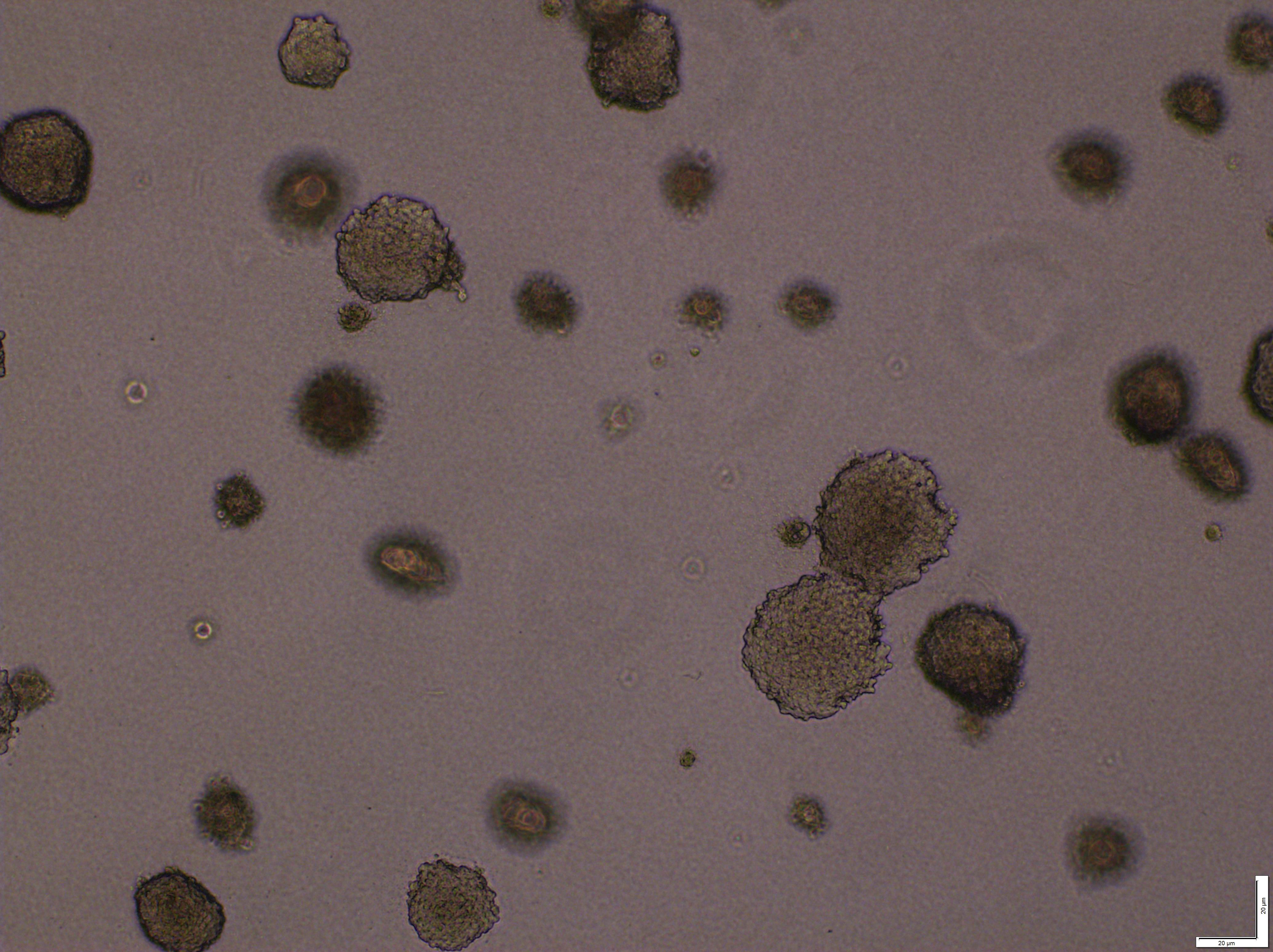

Supplement: Supplementary file 4 [file DataSheet_4.zip › fig 7d. PC-9, vector (5).jpg]

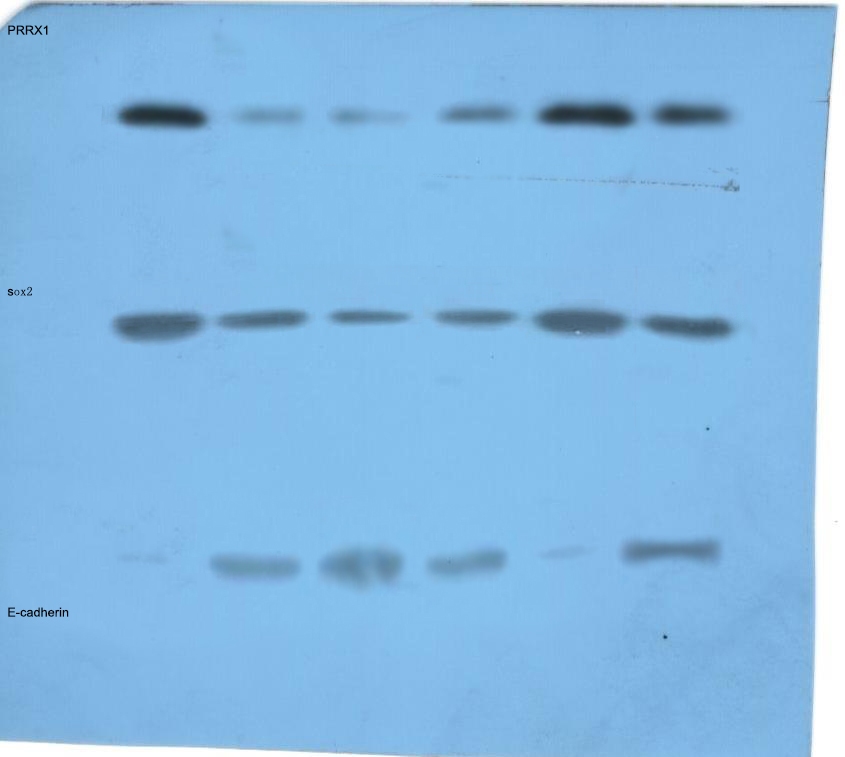

Supplement: Supplementary file 4 [file DataSheet_4.zip › fig 8b. PRRX1.jpg]

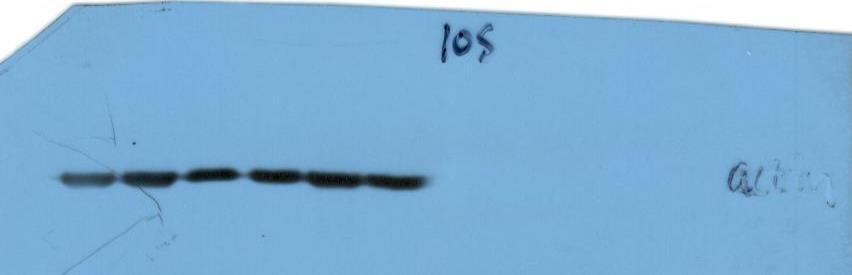

Supplement: Supplementary file 4 [file DataSheet_4.zip › fig 8b. a┬-actin.jpg]

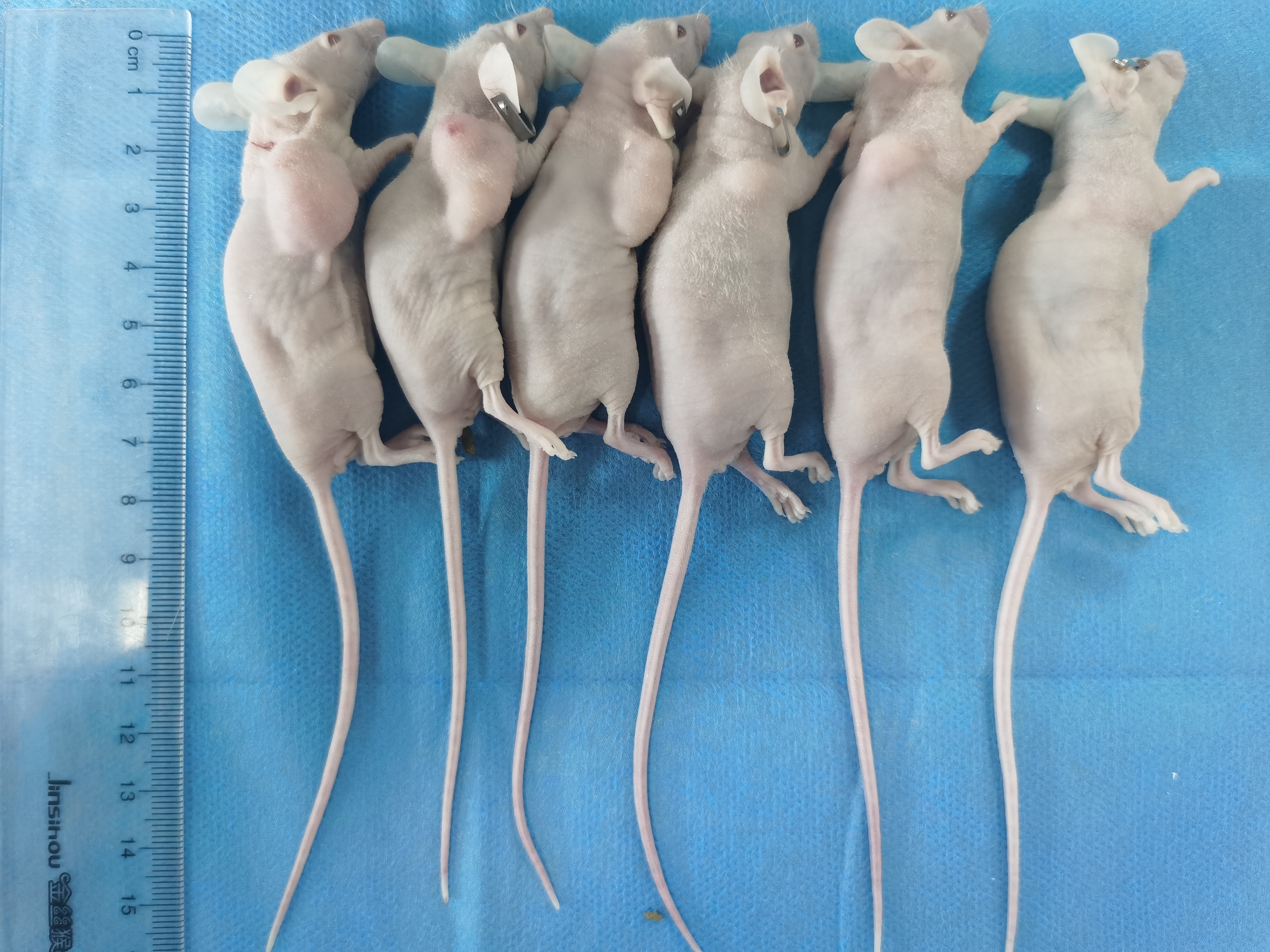

Supplement: Supplementary file 5 [file DataSheet_5.zip › fig 9a. IMG_20210721_113045.jpg]

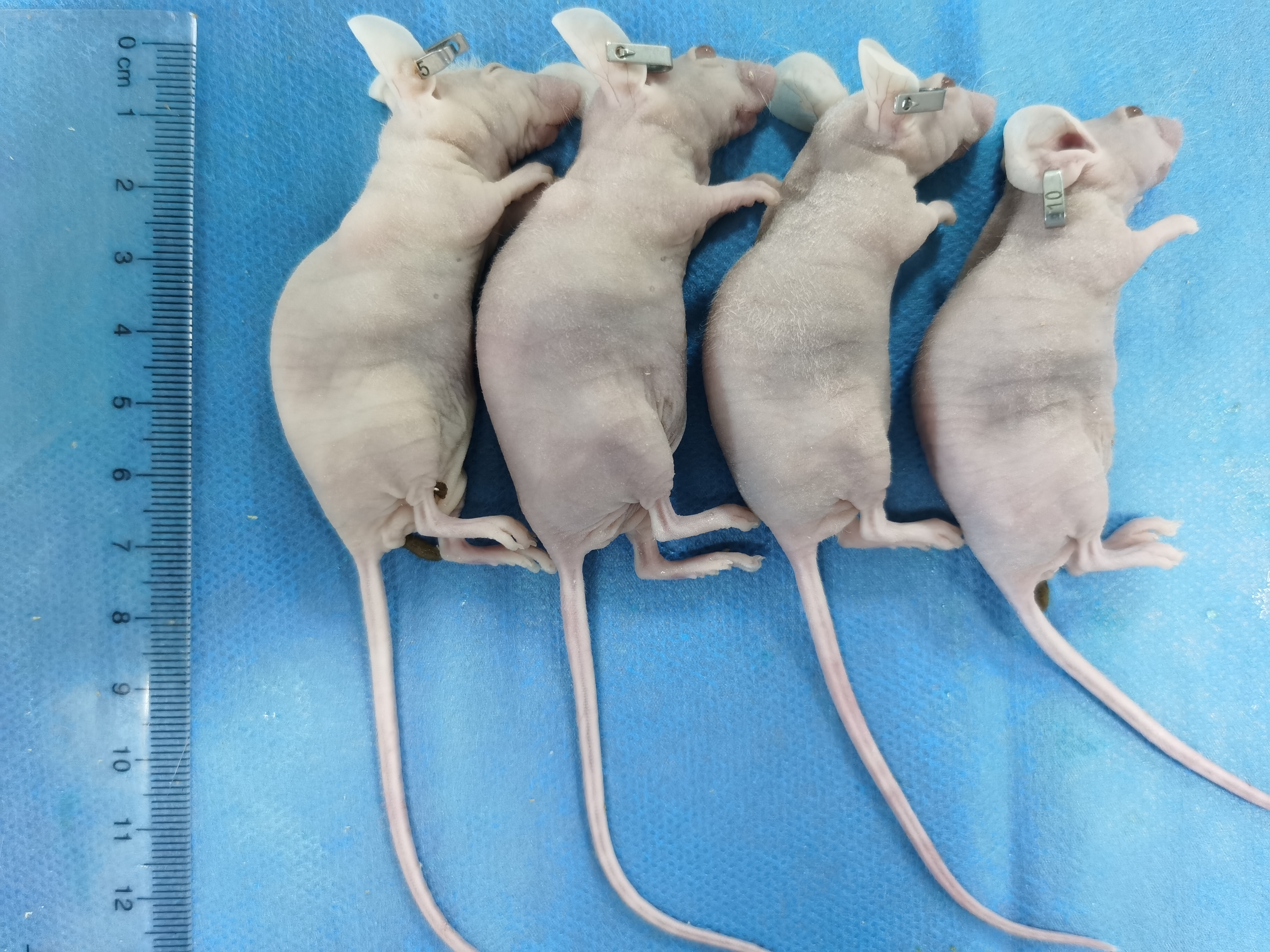

Supplement: Supplementary file 5 [file DataSheet_5.zip › fig 9a. IMG_20210721_122419.jpg]

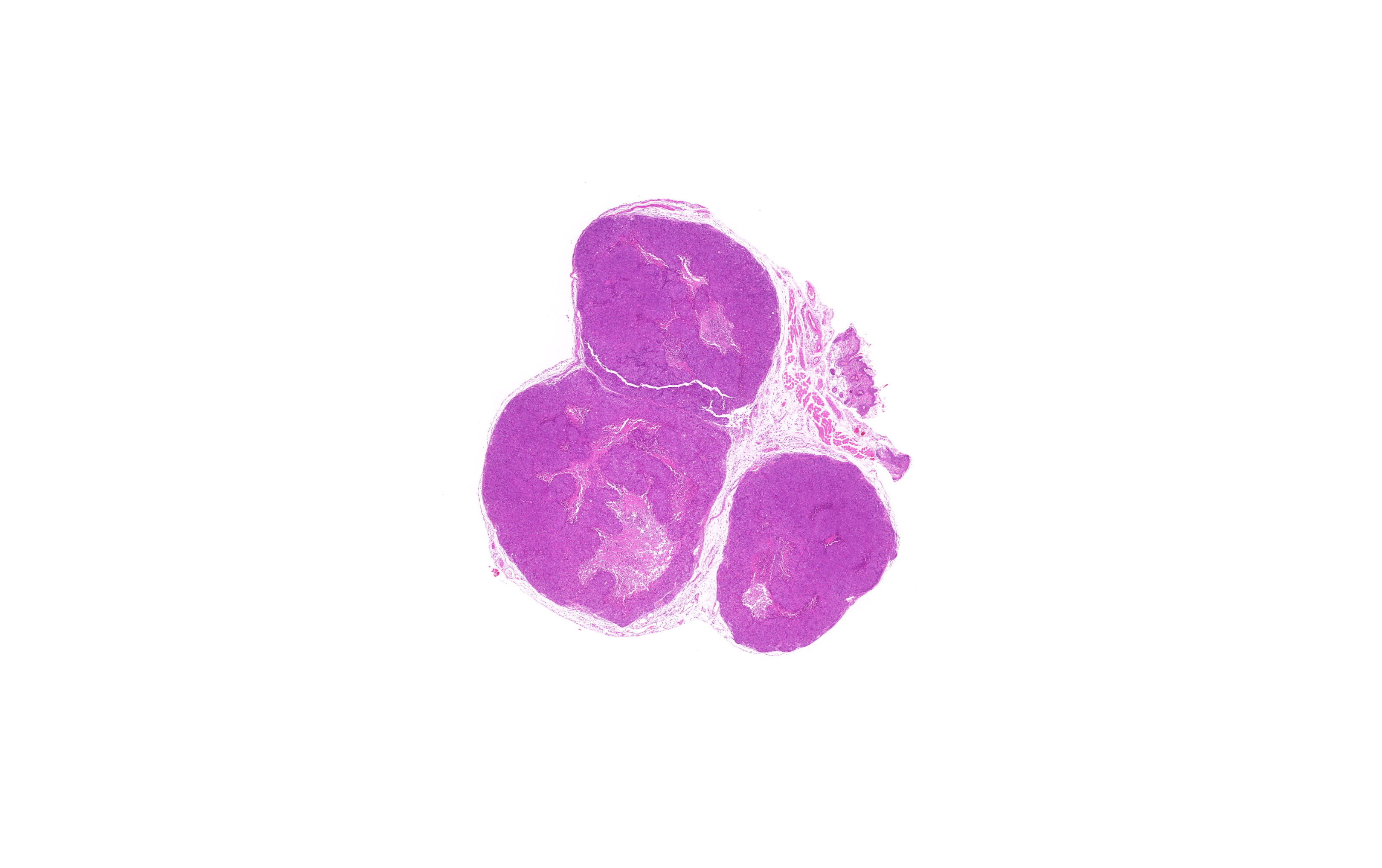

Supplement: Supplementary file 5 [file DataSheet_5.zip › fig 9b. shALKBH5-1_2.0x.jpg]

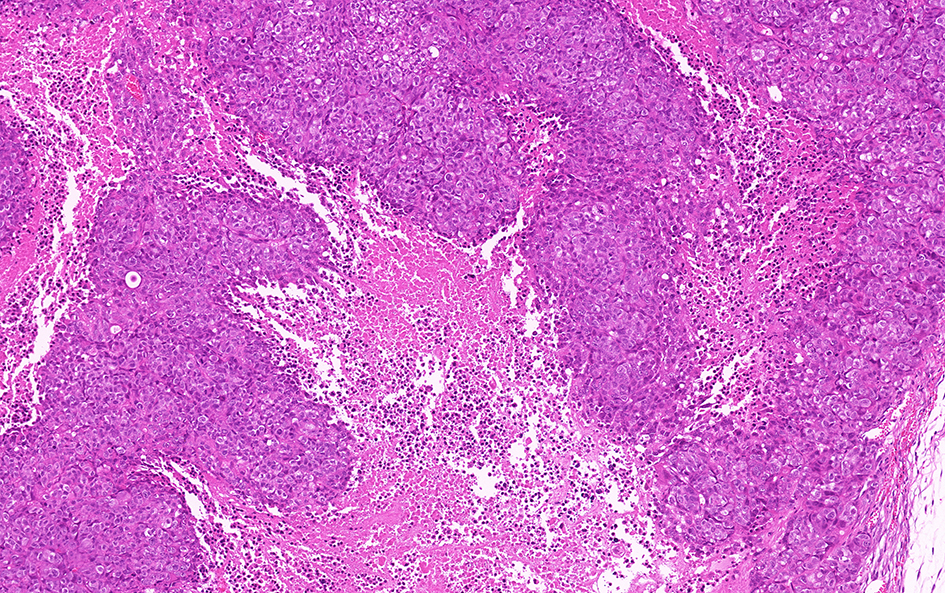

Supplement: Supplementary file 5 [file DataSheet_5.zip › fig 9b. shALKBH5-1_20x.jpg]

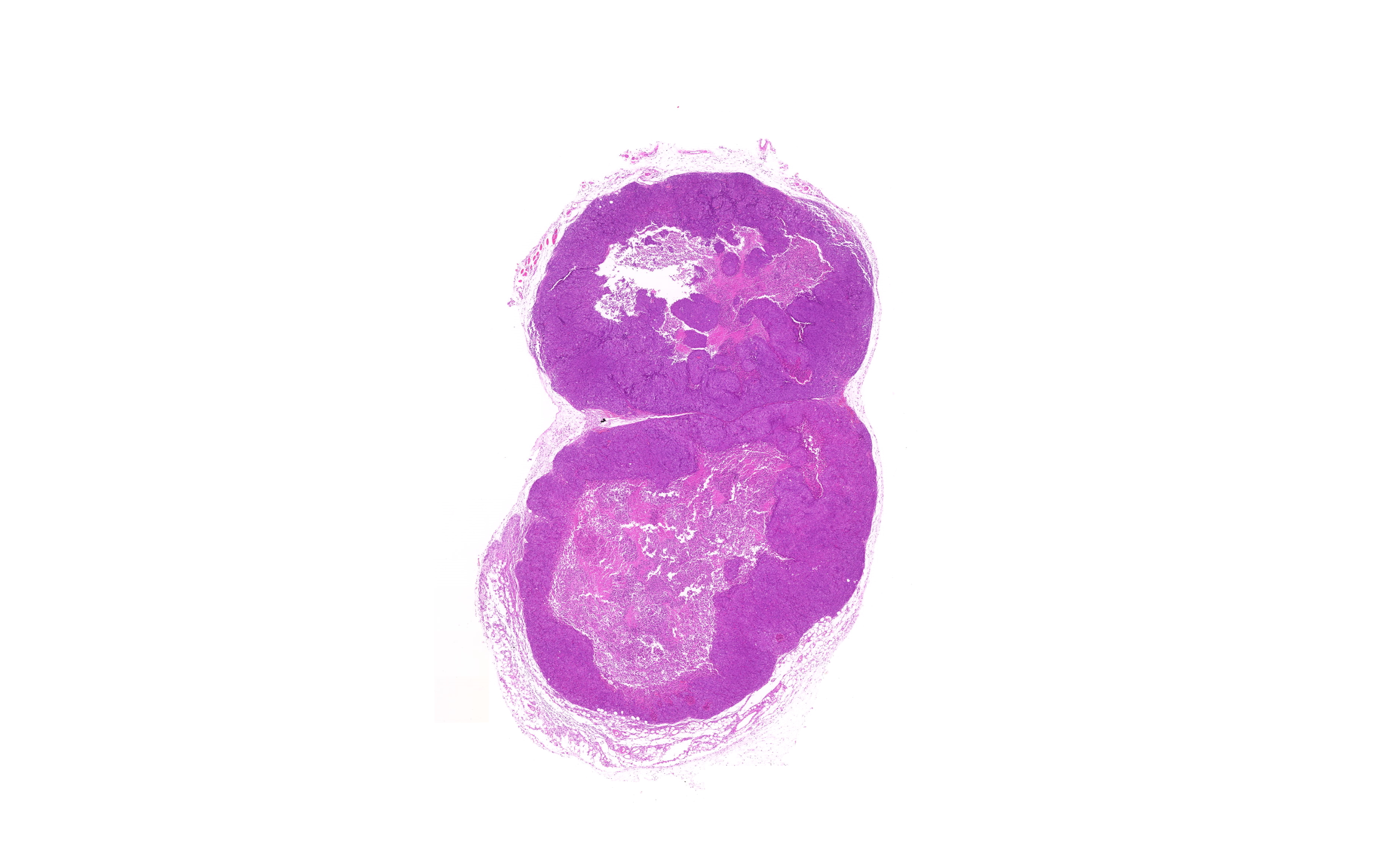

Supplement: Supplementary file 5 [file DataSheet_5.zip › fig 9b. shALKBH5-2_2.0x.jpg]

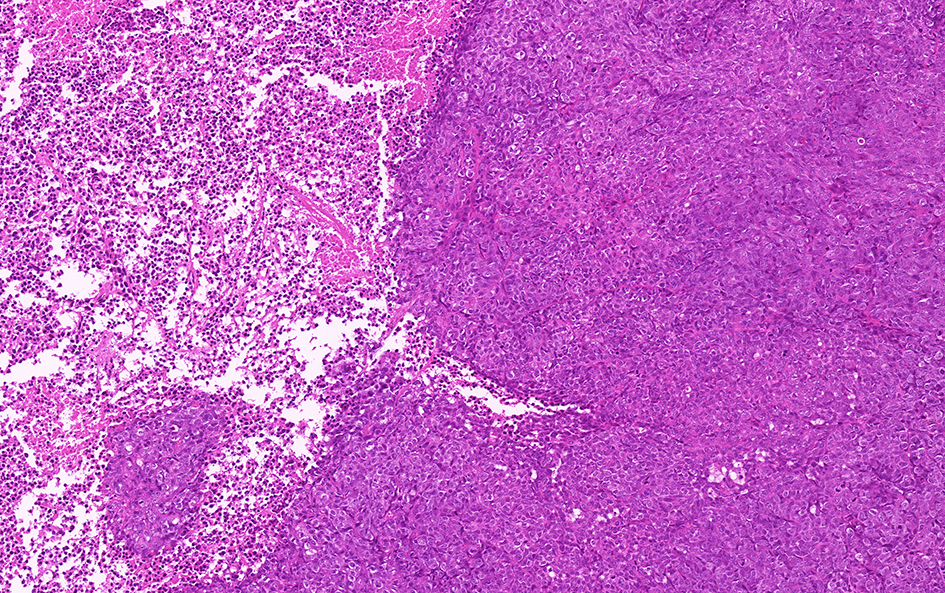

Supplement: Supplementary file 5 [file DataSheet_5.zip › fig 9b. shALKBH5-2_20x.jpg]

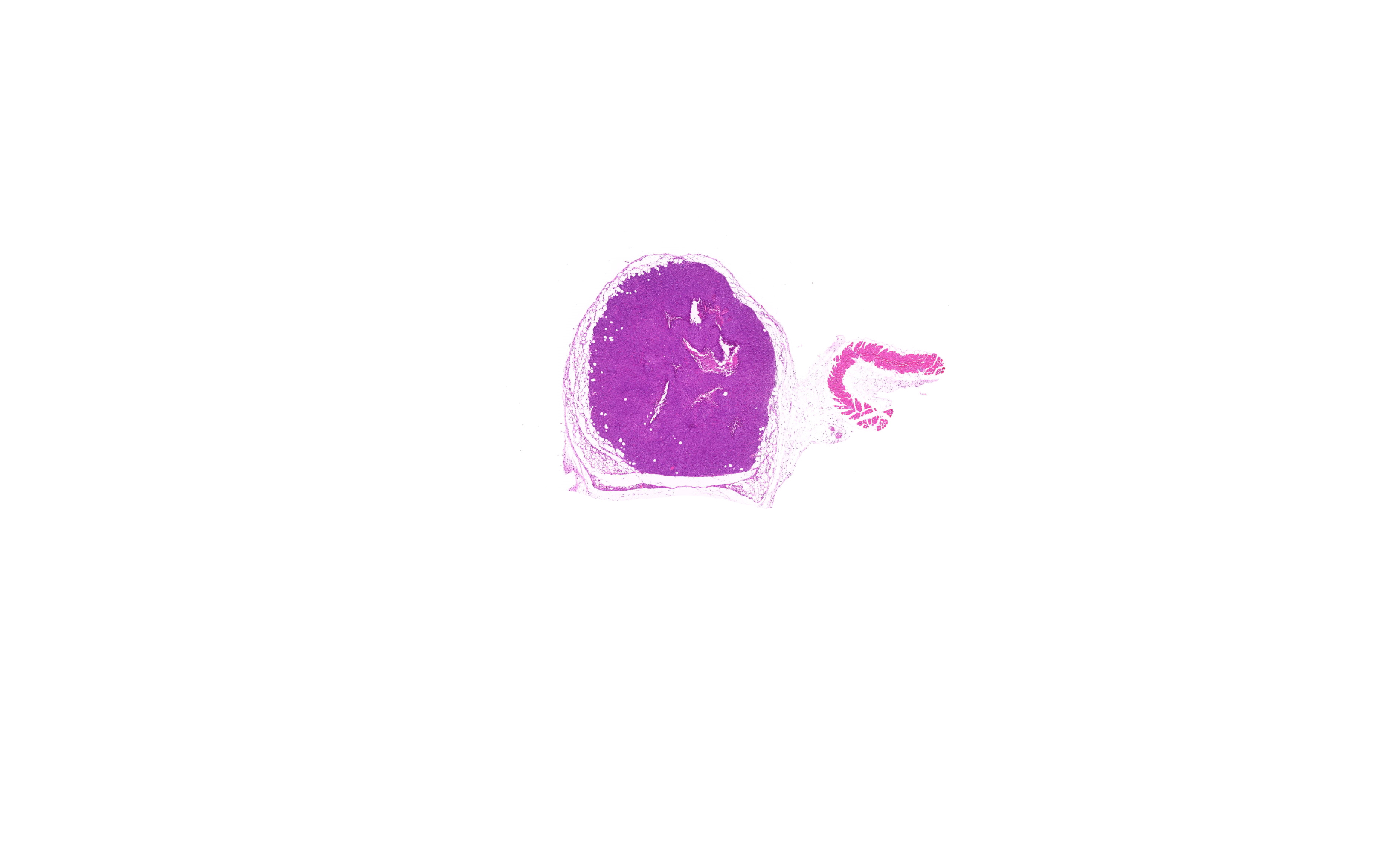

Supplement: Supplementary file 5 [file DataSheet_5.zip › fig 9b. shALKBH5-3_2.0x.jpg]

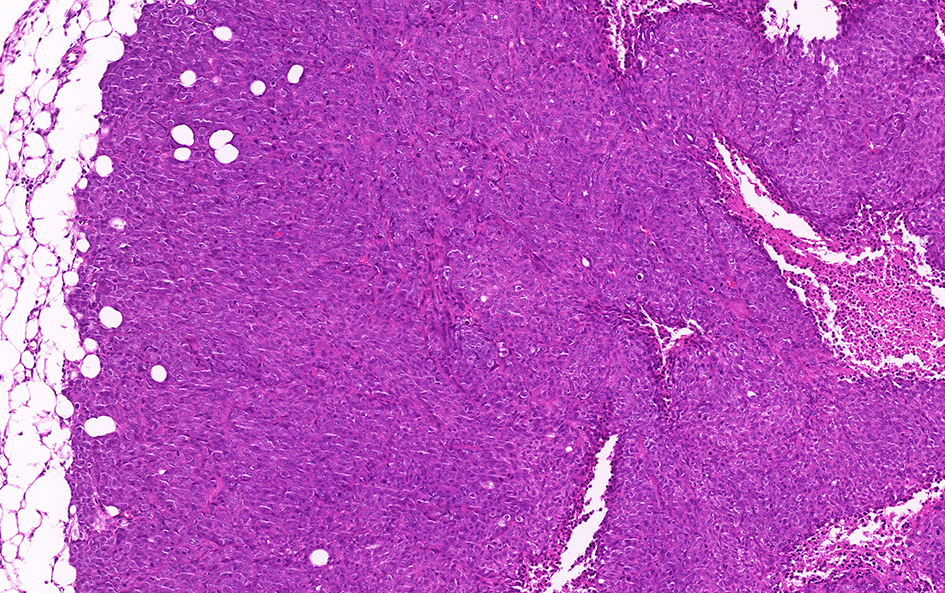

Supplement: Supplementary file 5 [file DataSheet_5.zip › fig 9b. shALKBH5-3_20x.jpg]

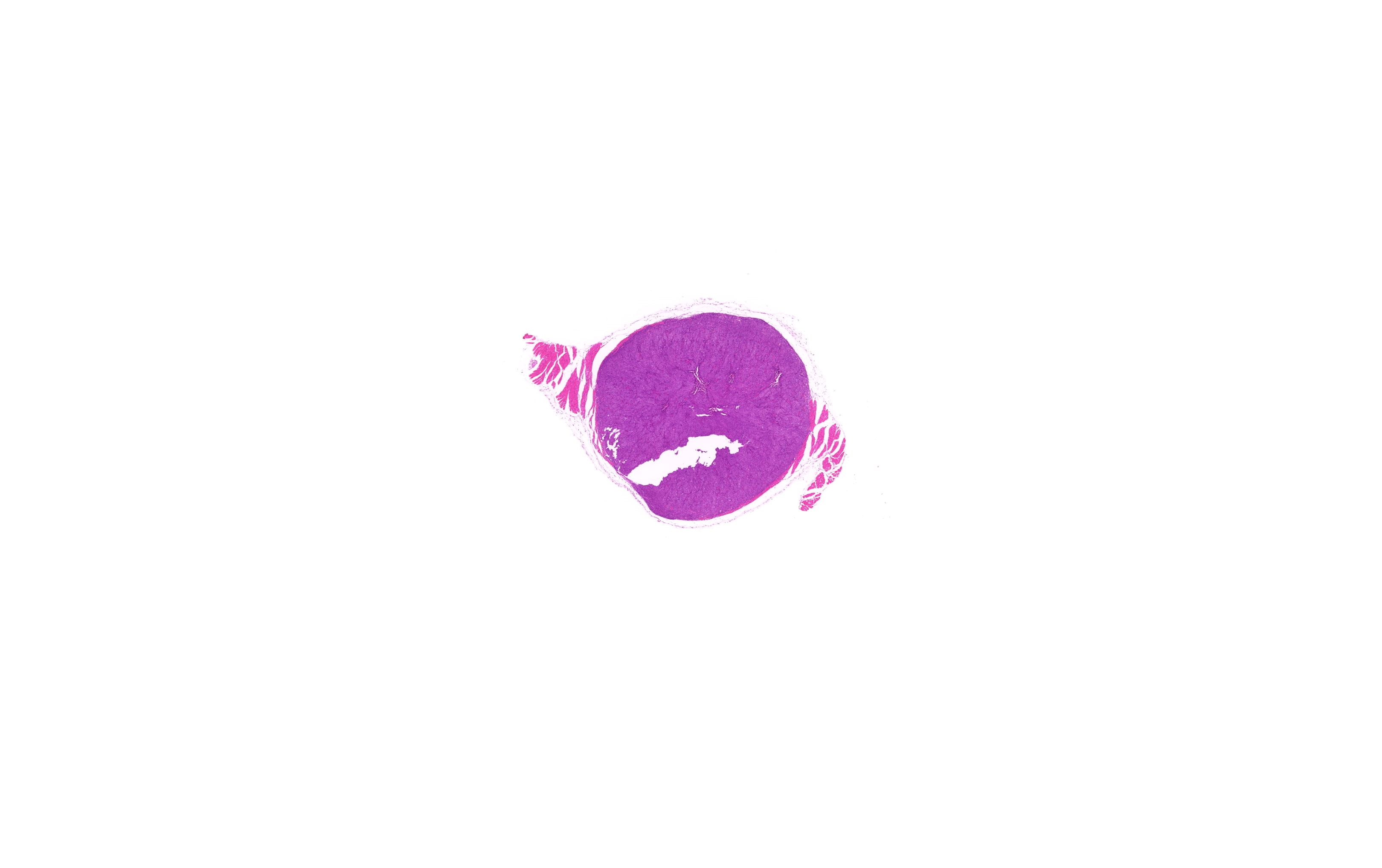

Supplement: Supplementary file 5 [file DataSheet_5.zip › fig 9b. shALKBH5-4_2.0x.jpg]

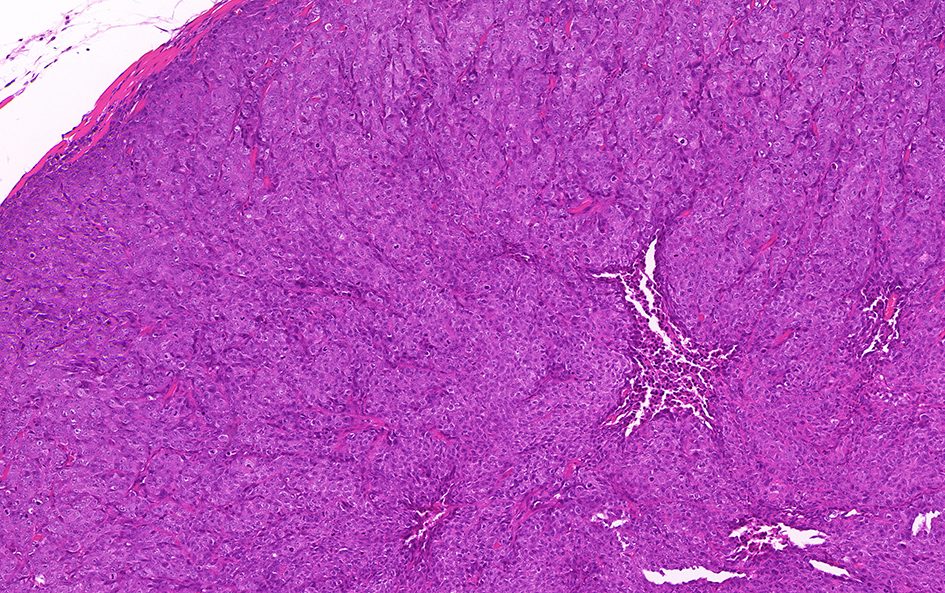

Supplement: Supplementary file 5 [file DataSheet_5.zip › fig 9b. shALKBH5-4_20x.jpg]

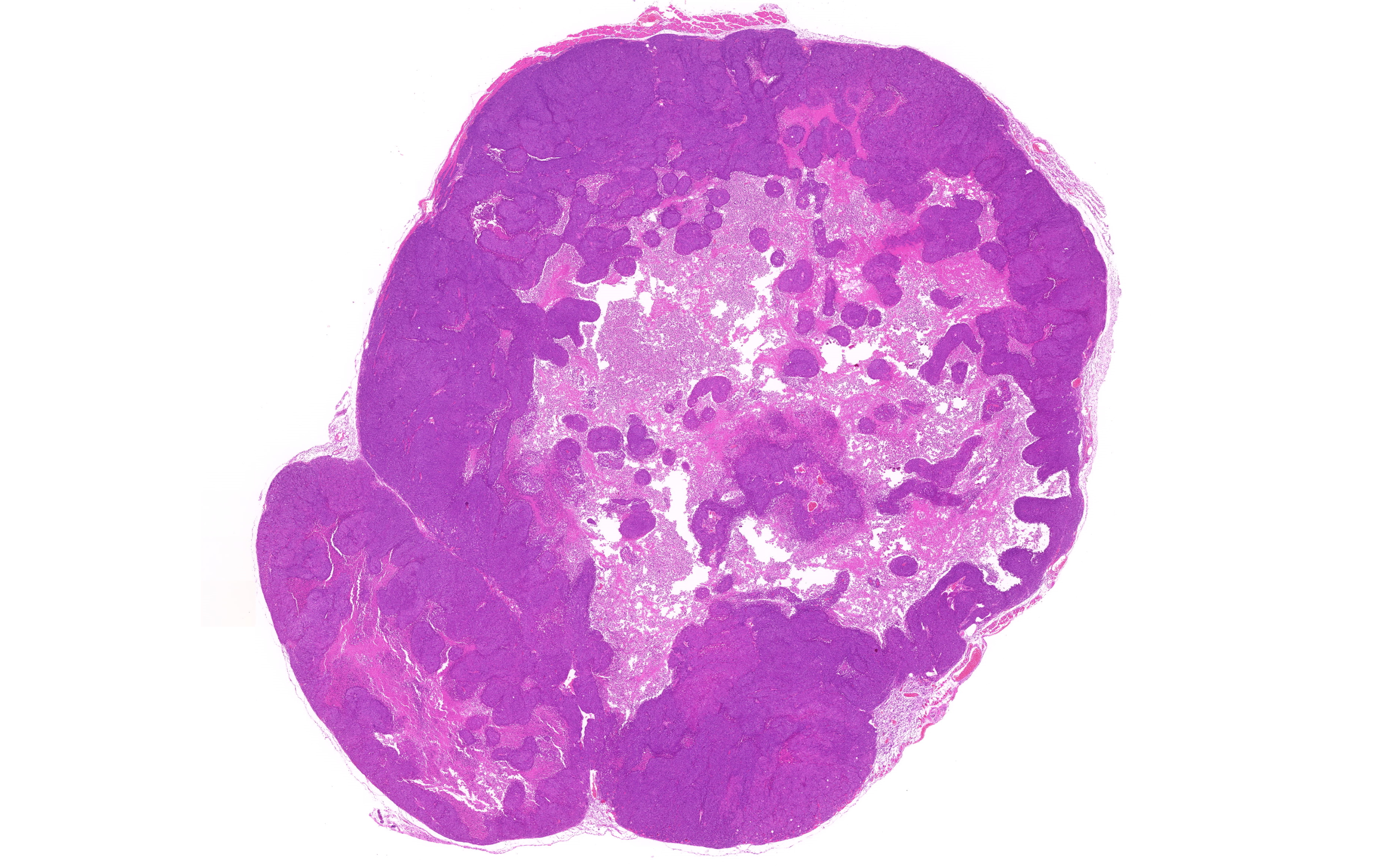

Supplement: Supplementary file 5 [file DataSheet_5.zip › fig 9b. shScrambled-1_2.0x.jpg]

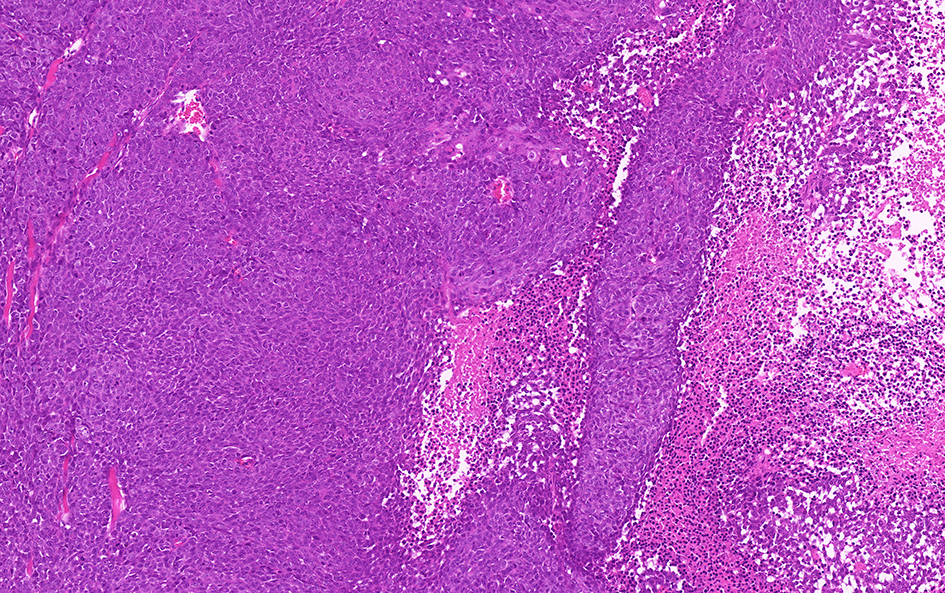

Supplement: Supplementary file 5 [file DataSheet_5.zip › fig 9b. shScrambled-1_20x.jpg]

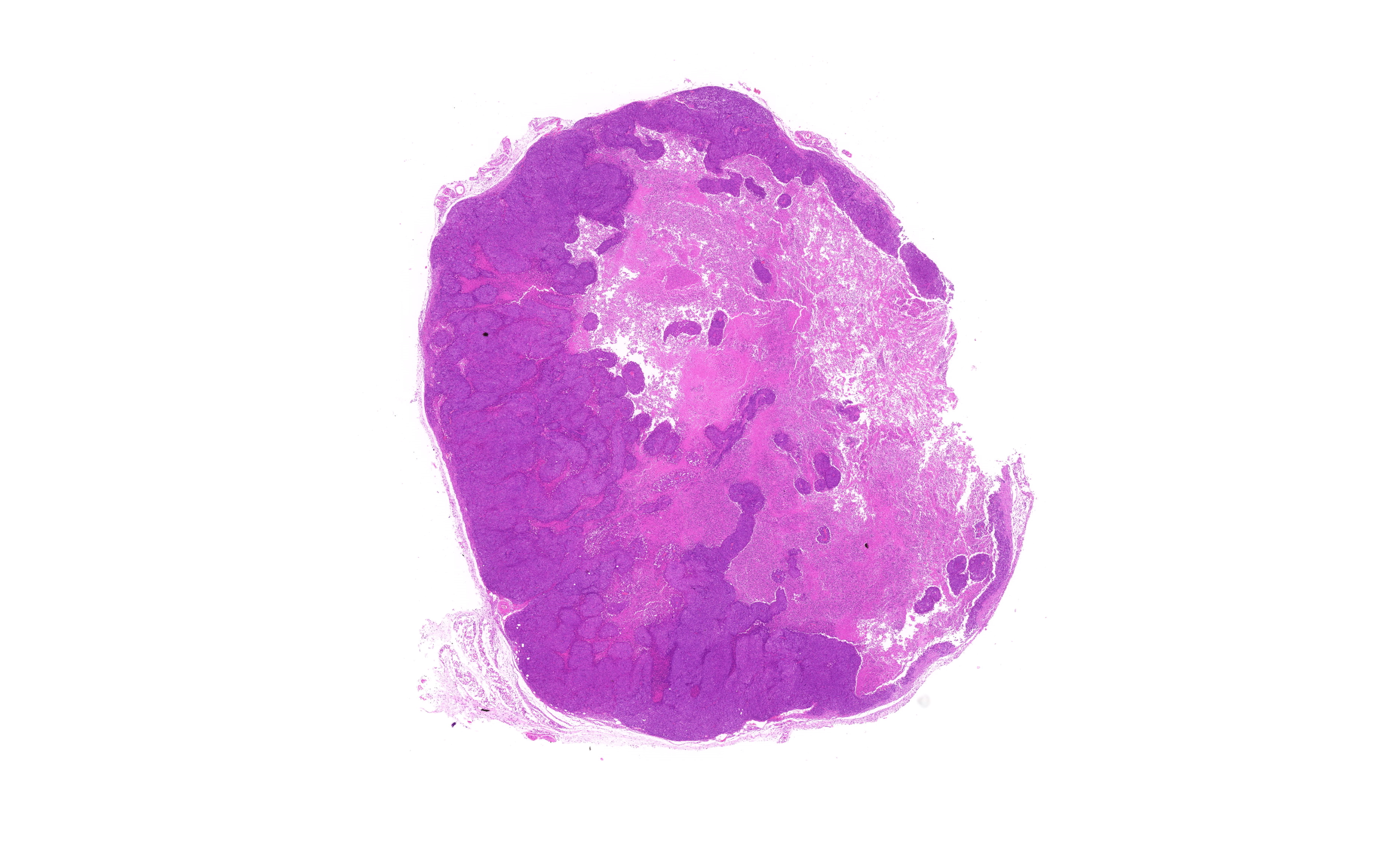

Supplement: Supplementary file 5 [file DataSheet_5.zip › fig 9b. shScrambled-2_2.0x.jpg]

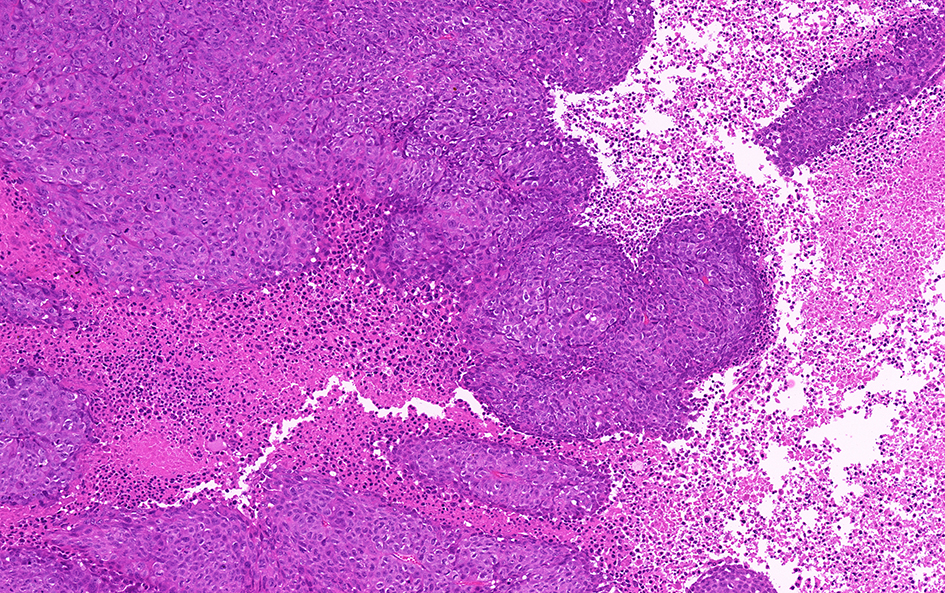

Supplement: Supplementary file 6 [file DataSheet_6.zip › fig 9b. shScrambled-2_20x.jpg]

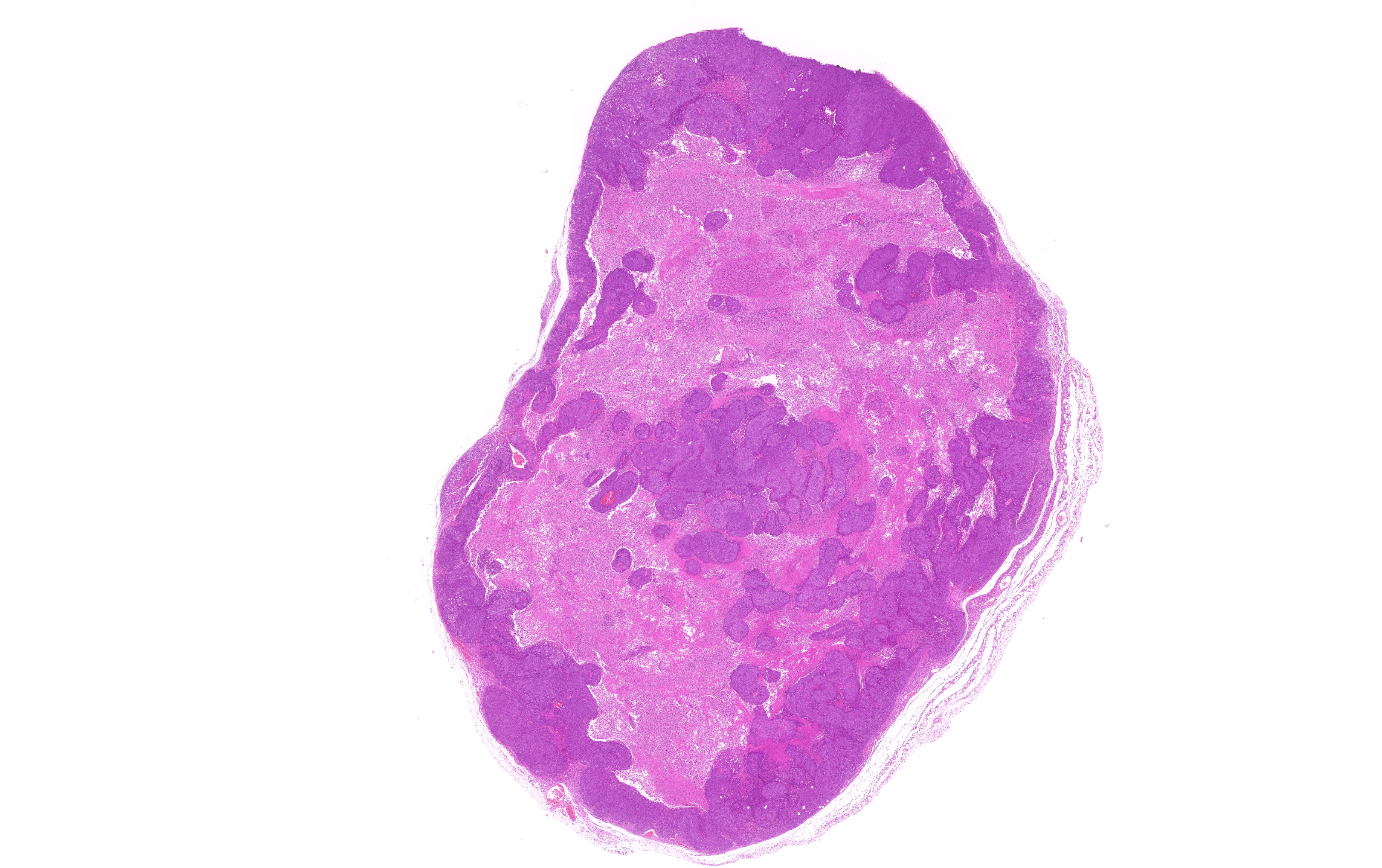

Supplement: Supplementary file 6 [file DataSheet_6.zip › fig 9b. shScrambled-3_2.0x.jpg]

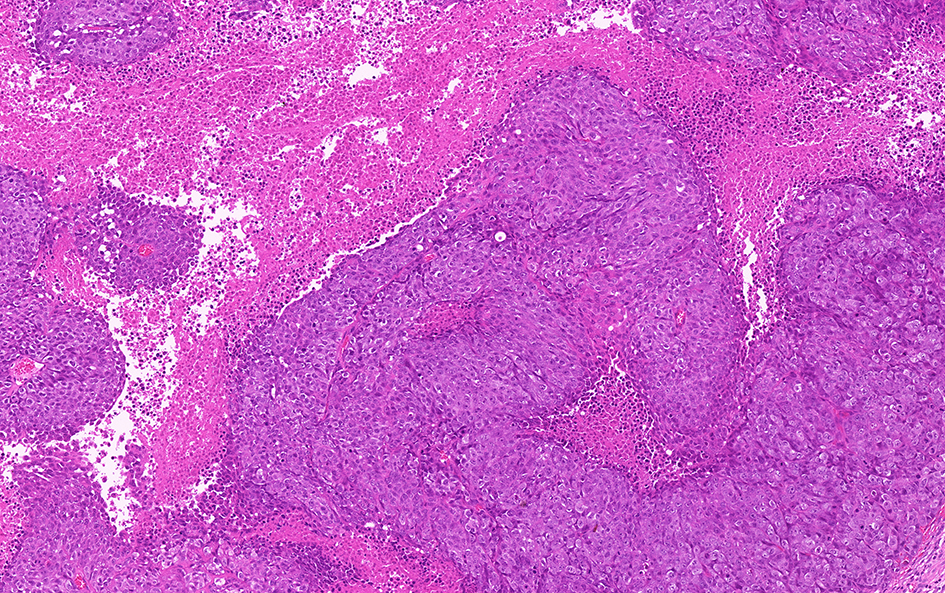

Supplement: Supplementary file 6 [file DataSheet_6.zip › fig 9b. shScrambled-3_20x.jpg]

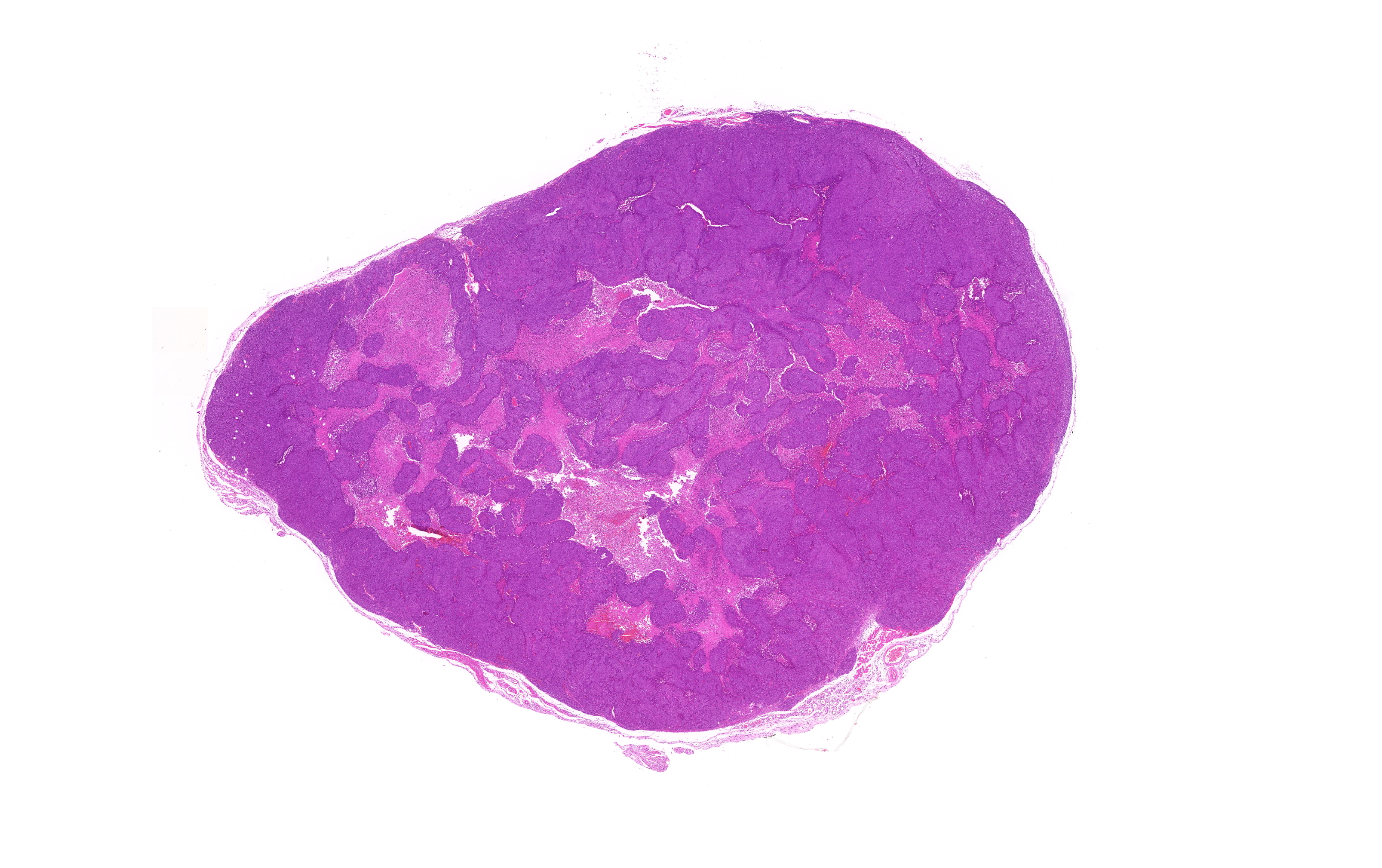

Supplement: Supplementary file 6 [file DataSheet_6.zip › fig 9b. shScrambled-4_2.0x.jpg]

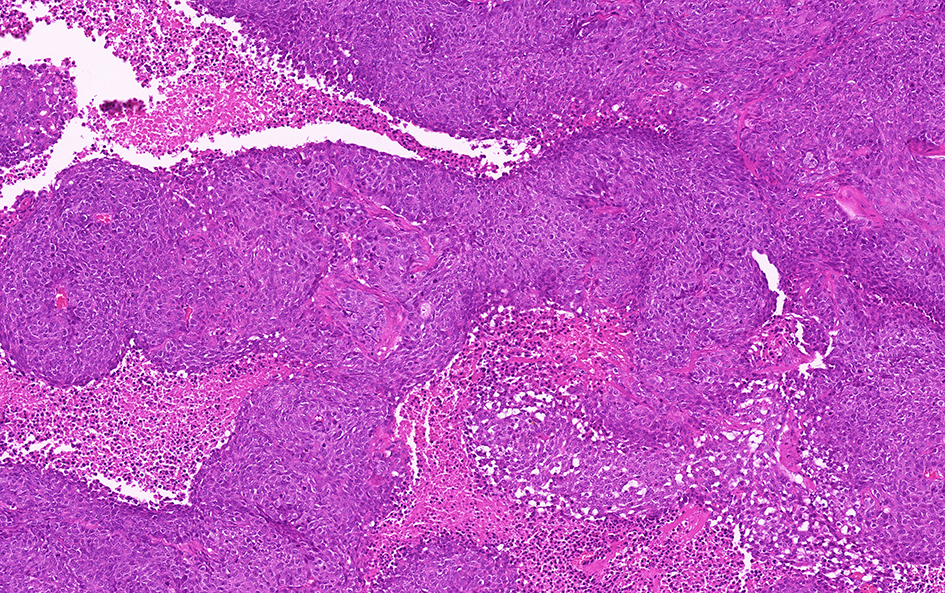

Supplement: Supplementary file 6 [file DataSheet_6.zip › fig 9b. shScrambled-4_20x.jpg]

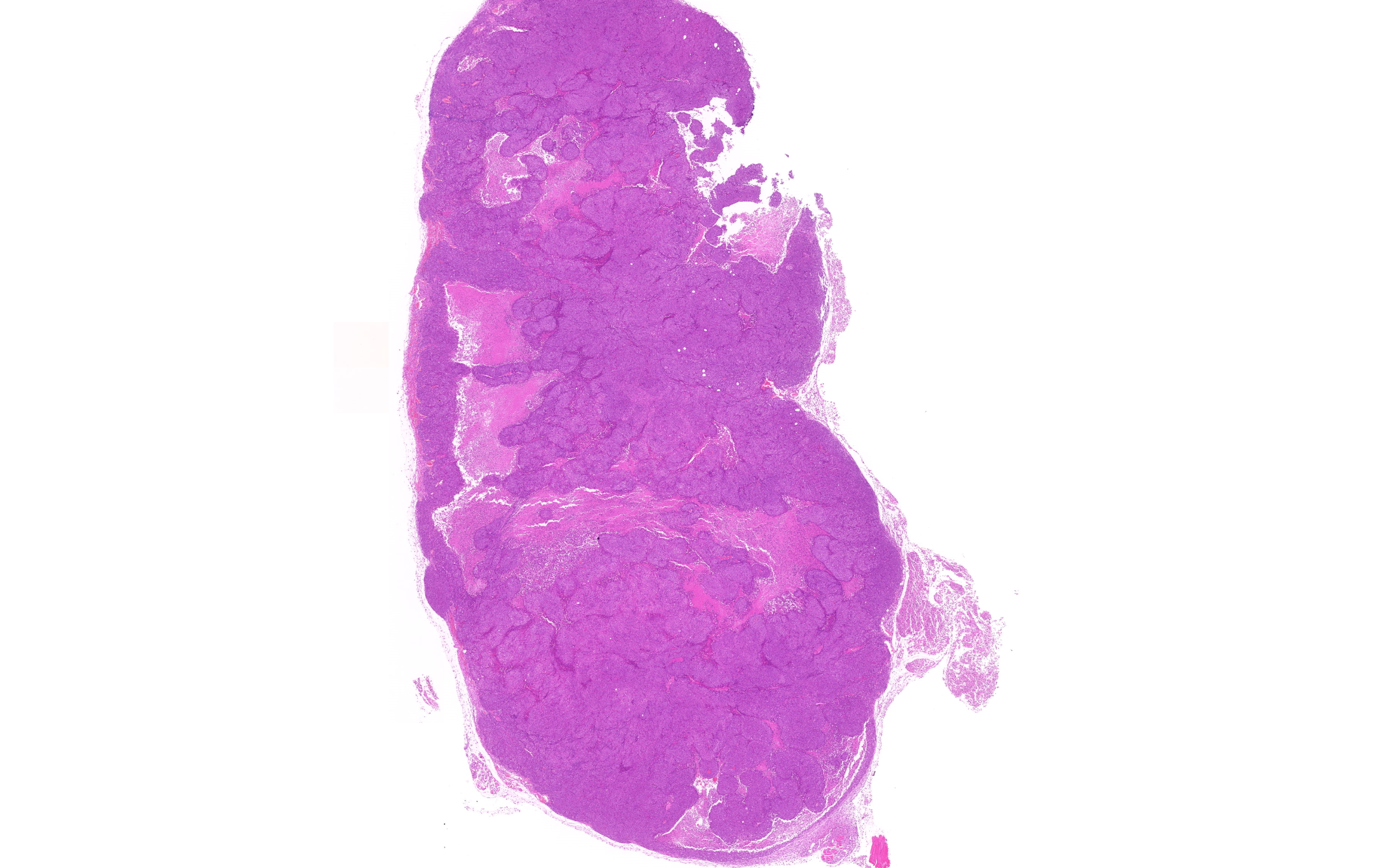

Supplement: Supplementary file 6 [file DataSheet_6.zip › fig 9b. shScrambled-5_2.0x.jpg]

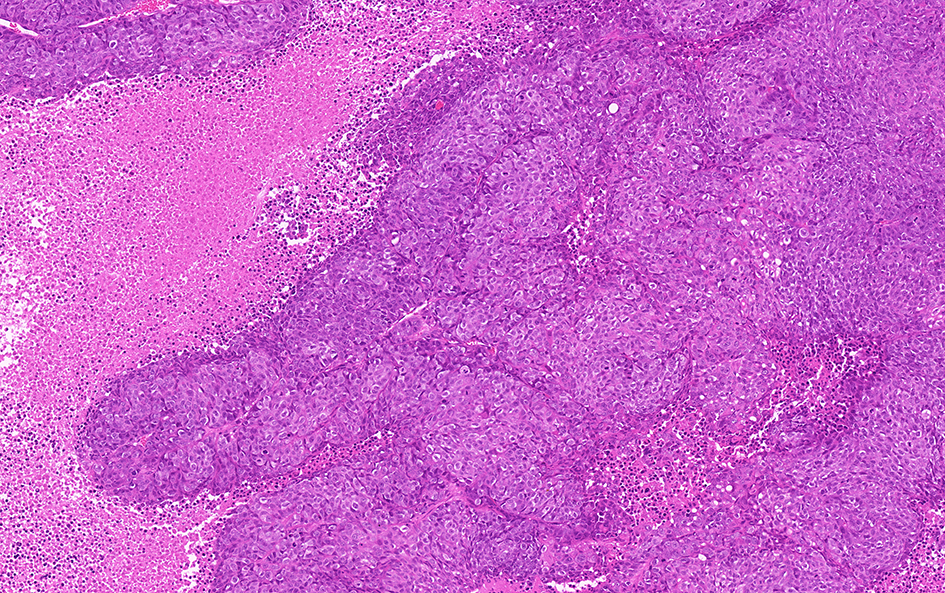

Supplement: Supplementary file 6 [file DataSheet_6.zip › fig 9b. shScrambled-5_20x.jpg]

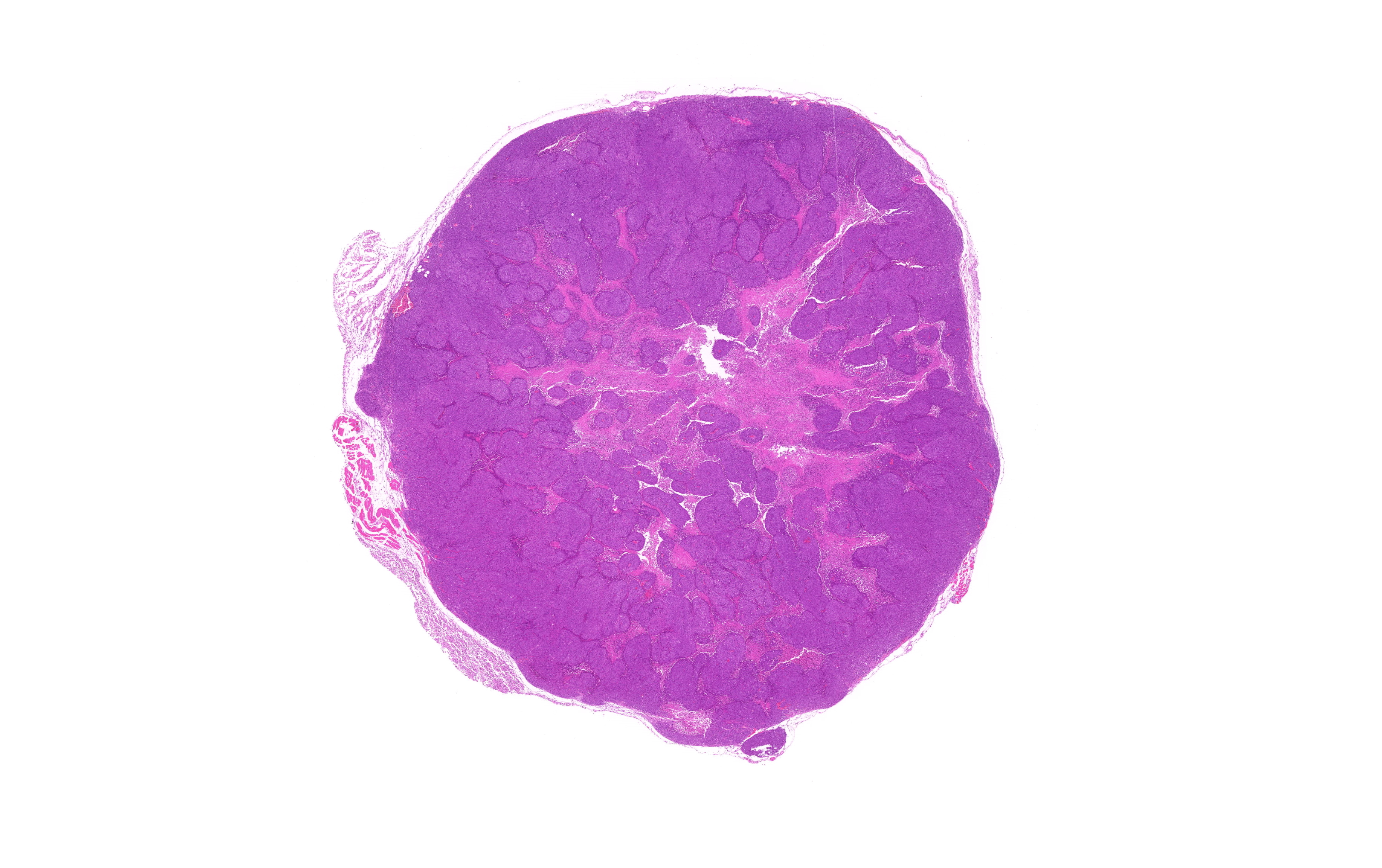

Supplement: Supplementary file 6 [file DataSheet_6.zip › fig 9b. shScrambled-6_2.0x.jpg]

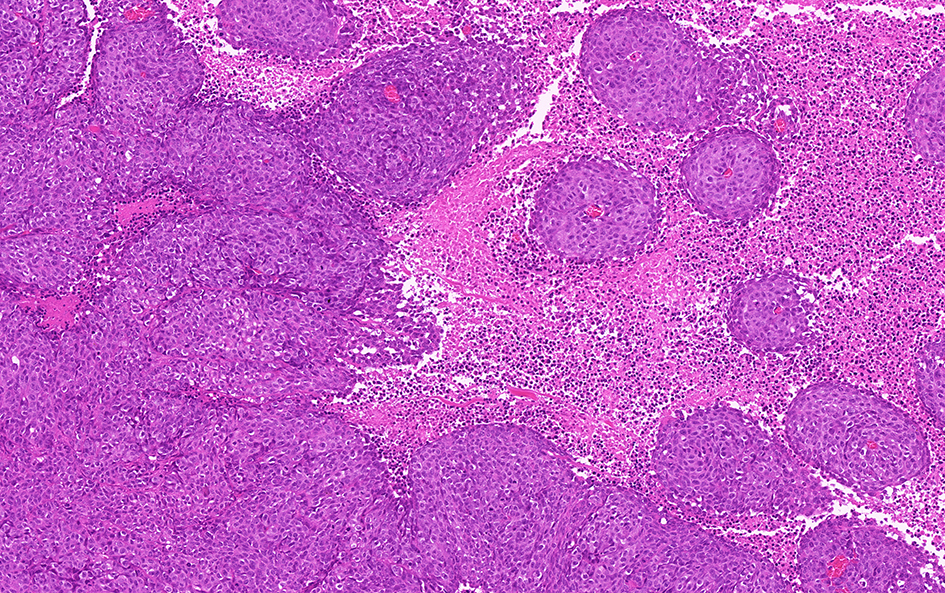

Supplement: Supplementary file 6 [file DataSheet_6.zip › fig 9b. shScrambled-6_20x.jpg]

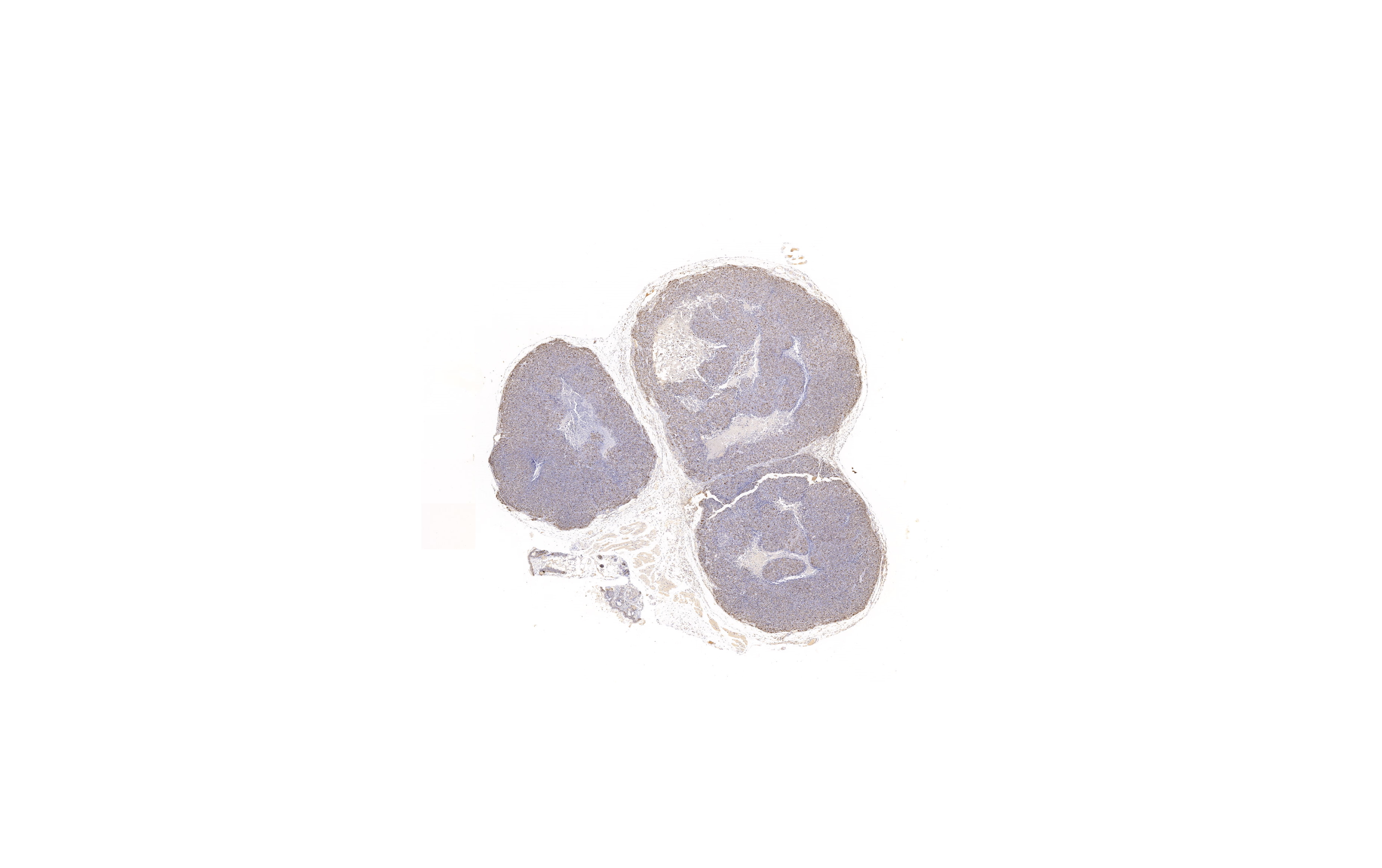

Supplement: Supplementary file 6 [file DataSheet_6.zip › fig 9c. shALKBH5-1_2.0x ki67.jpg]

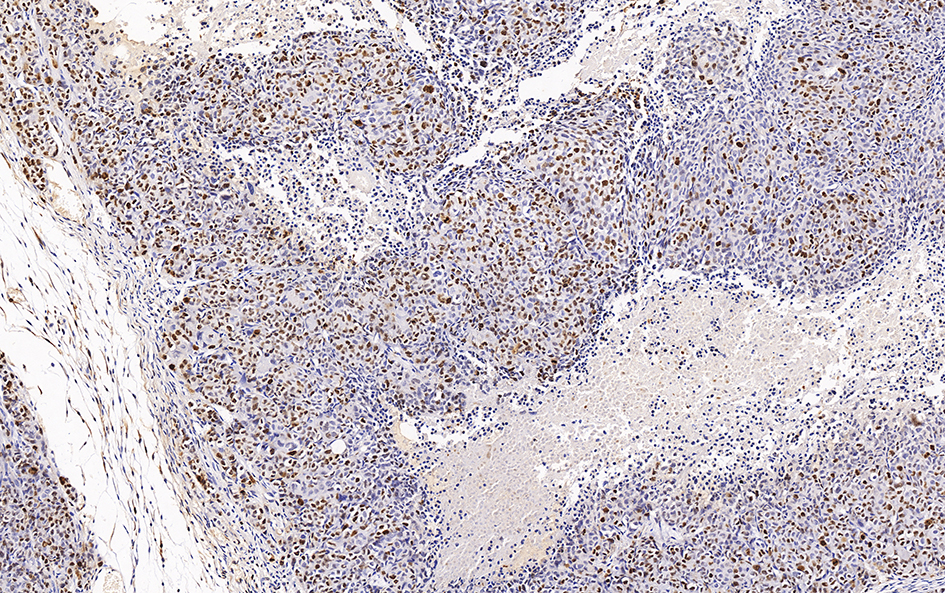

Supplement: Supplementary file 6 [file DataSheet_6.zip › fig 9c. shALKBH5-1_20x ki67.jpg]

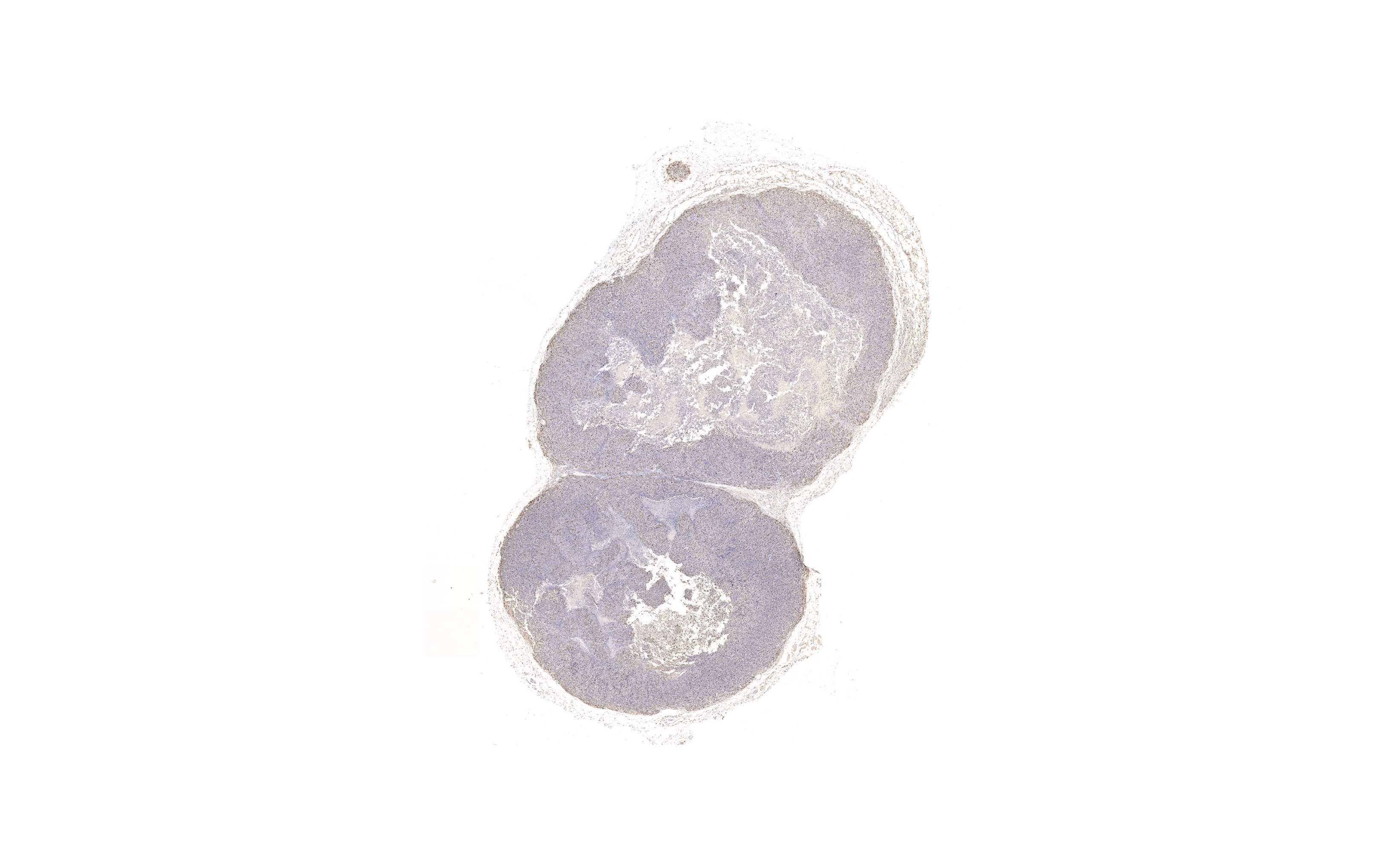

Supplement: Supplementary file 6 [file DataSheet_6.zip › fig 9c. shALKBH5-2_2.0x ki67.jpg]

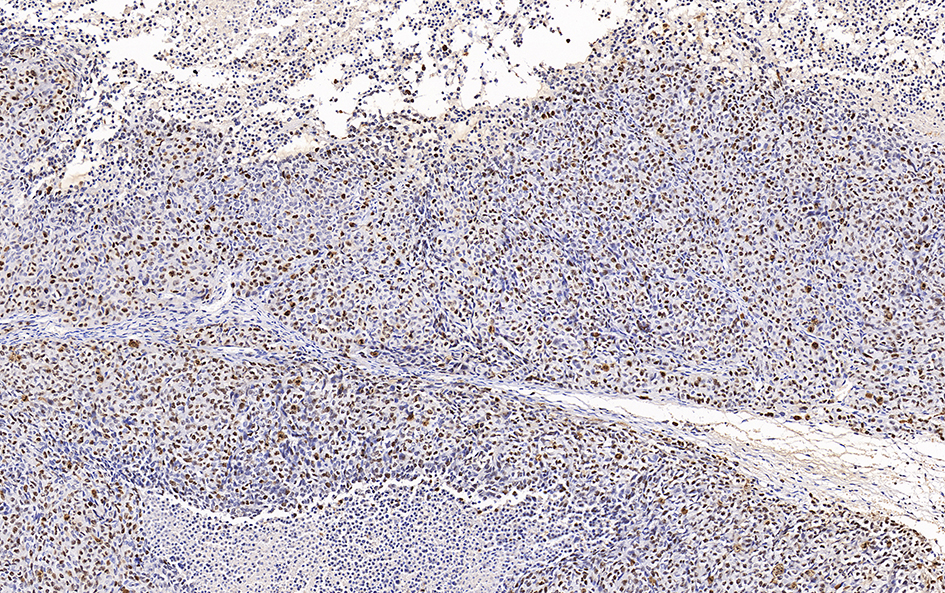

Supplement: Supplementary file 6 [file DataSheet_6.zip › fig 9c. shALKBH5-2_20x ki67.jpg]

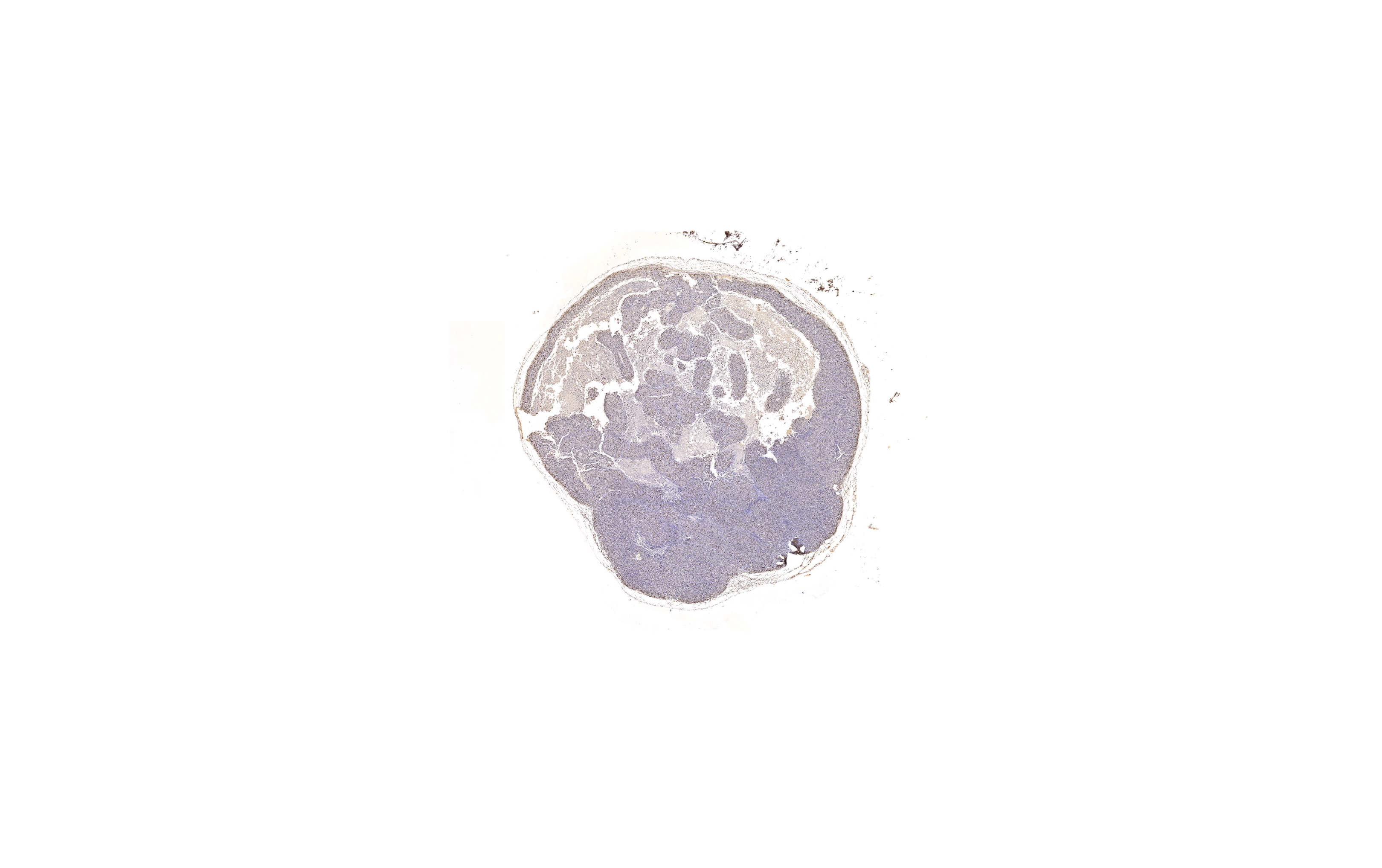

Supplement: Supplementary file 6 [file DataSheet_6.zip › fig 9c. shALKBH5-3_2.0x ki67.jpg]

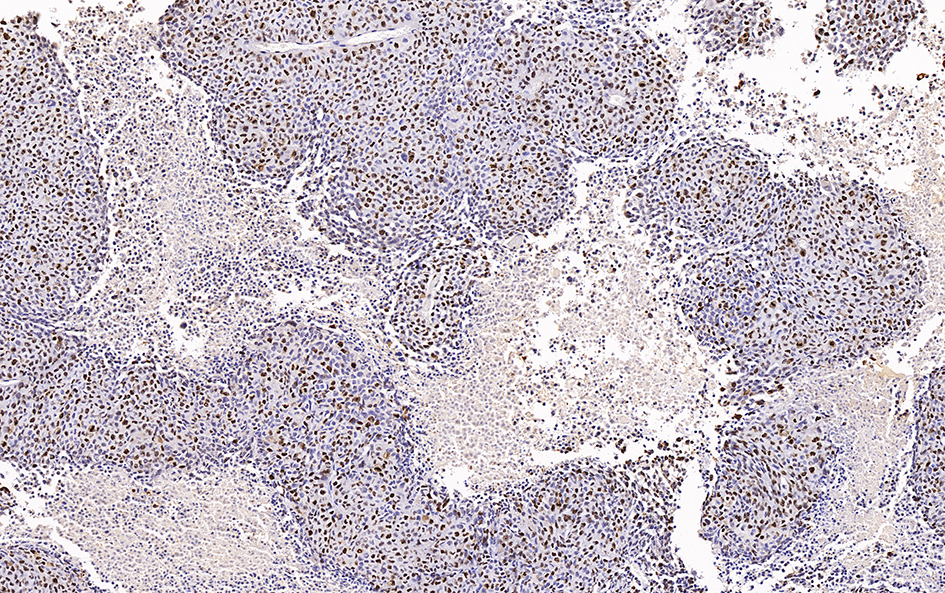

Supplement: Supplementary file 7 [file DataSheet_7.zip › fig 9c. shALKBH5-3_20x ki67.jpg]

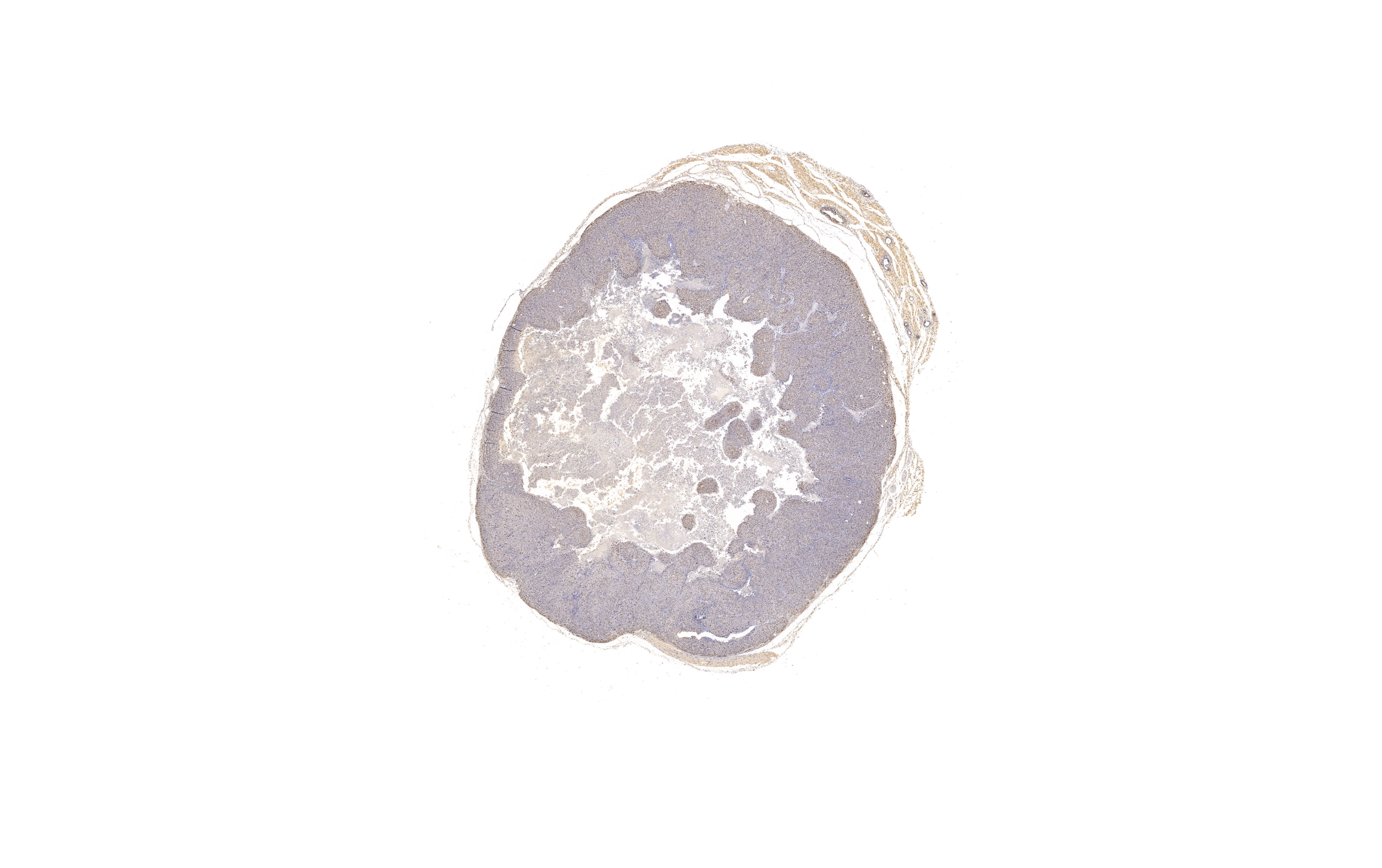

Supplement: Supplementary file 7 [file DataSheet_7.zip › fig 9c. shALKBH5-4_2.0x ki67.jpg]

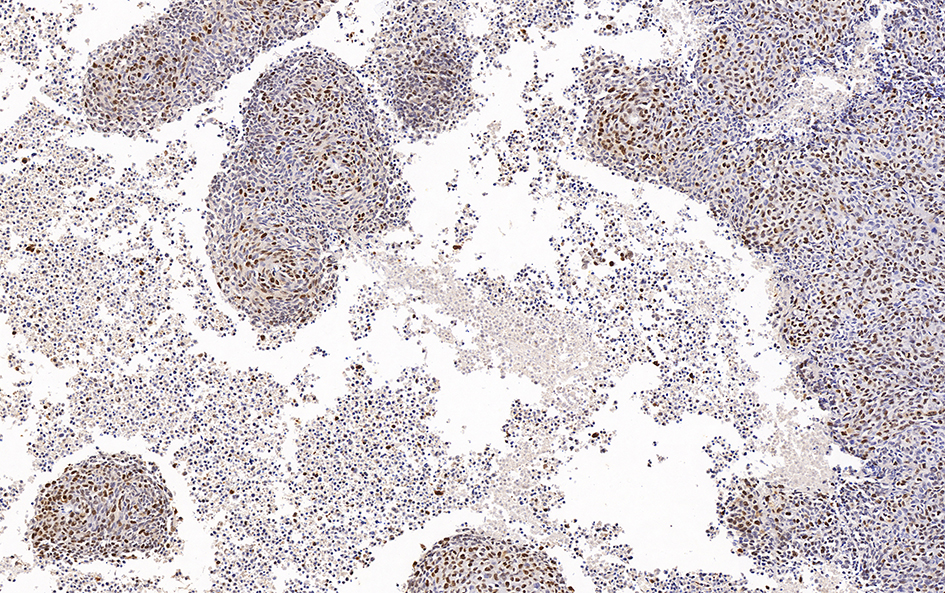

Supplement: Supplementary file 7 [file DataSheet_7.zip › fig 9c. shALKBH5-4_20x ki67.jpg]

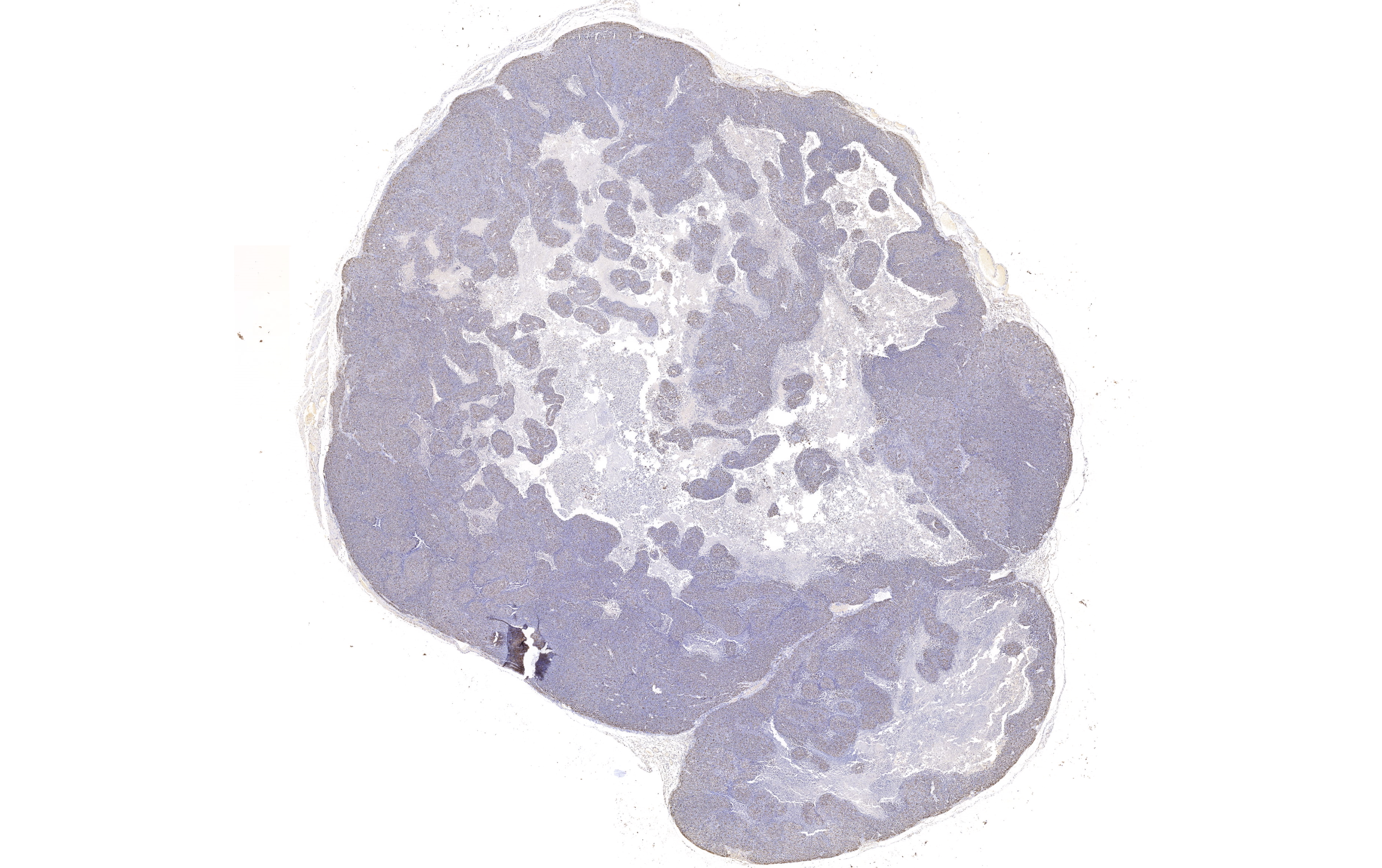

Supplement: Supplementary file 7 [file DataSheet_7.zip › fig 9c. shScrambled-1_2.0x ki67.jpg]

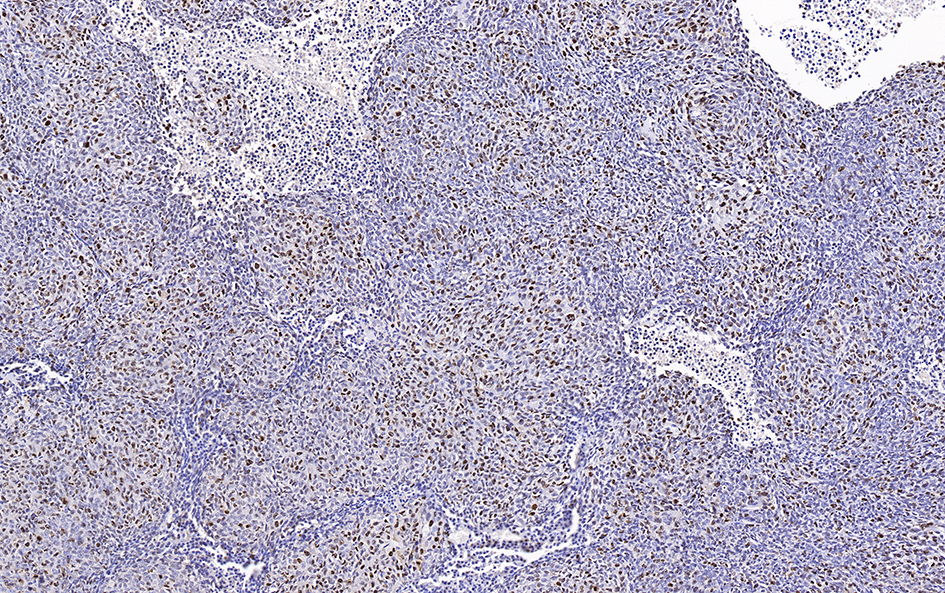

Supplement: Supplementary file 7 [file DataSheet_7.zip › fig 9c. shScrambled-1_20x ki67.jpg]

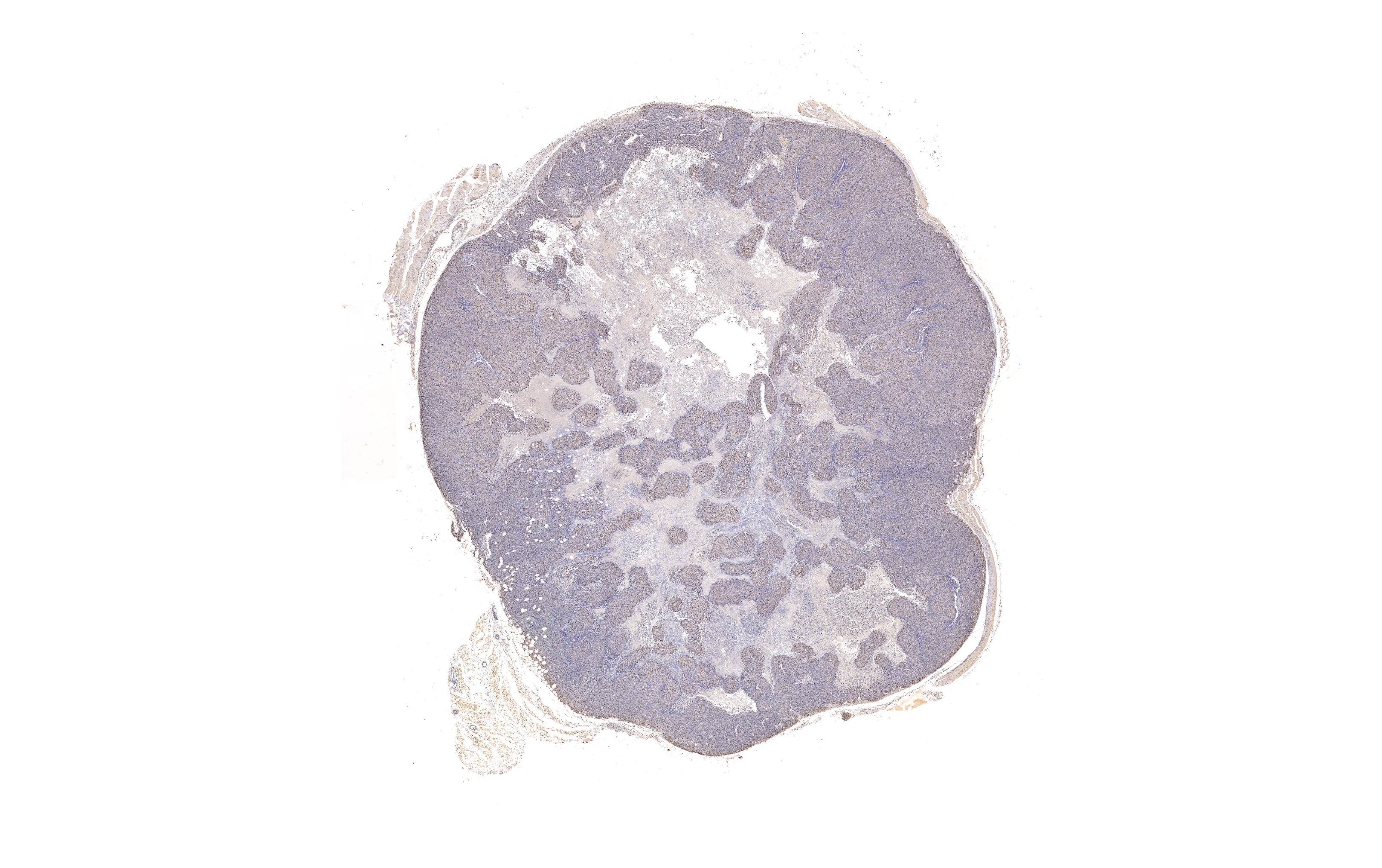

Supplement: Supplementary file 7 [file DataSheet_7.zip › fig 9c. shScrambled-2_2.0x ki67.jpg]

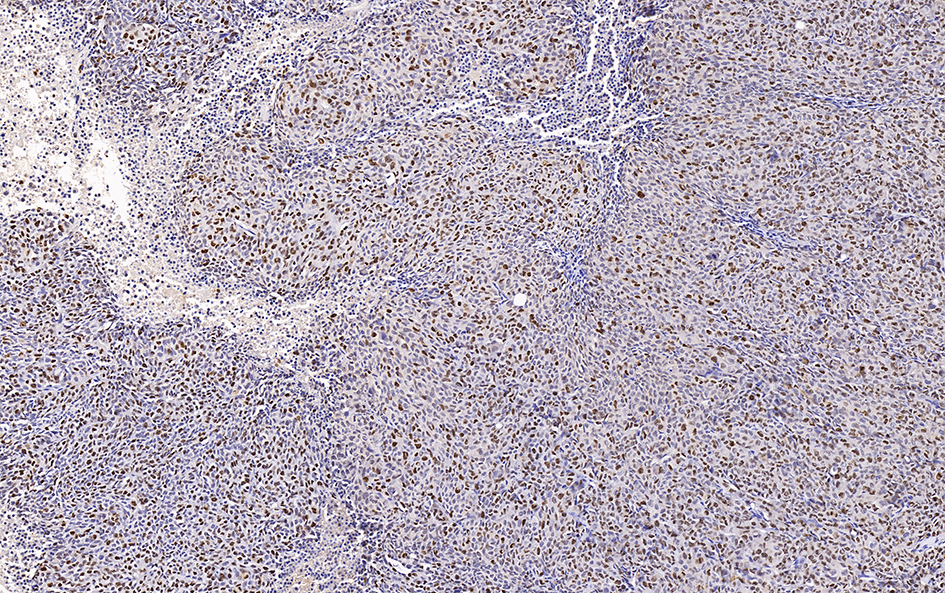

Supplement: Supplementary file 7 [file DataSheet_7.zip › fig 9c. shScrambled-2_20x ki67.jpg]

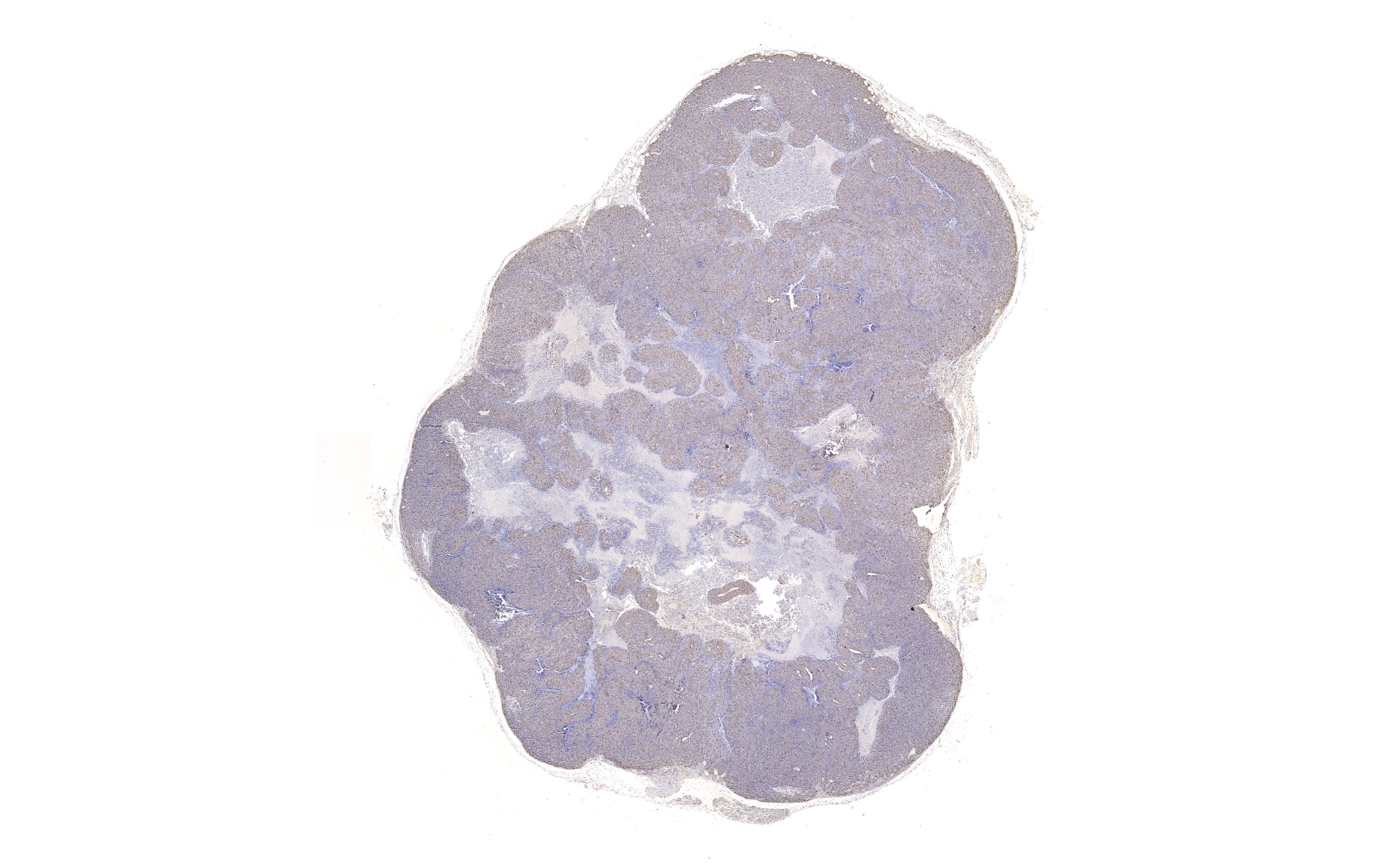

Supplement: Supplementary file 7 [file DataSheet_7.zip › fig 9c. shScrambled-3_2.0x ki67.jpg]

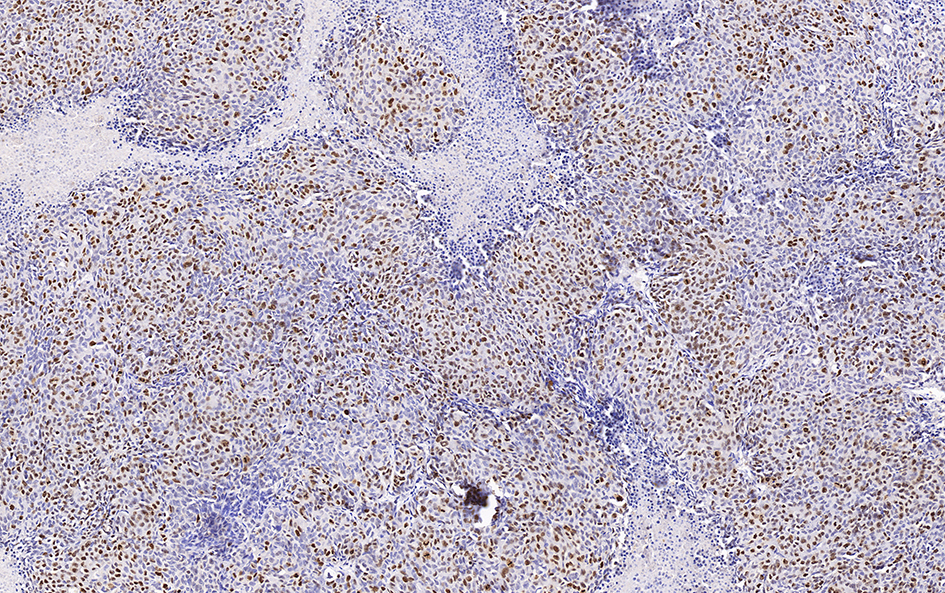

Supplement: Supplementary file 7 [file DataSheet_7.zip › fig 9c. shScrambled-3_20x ki67.jpg]

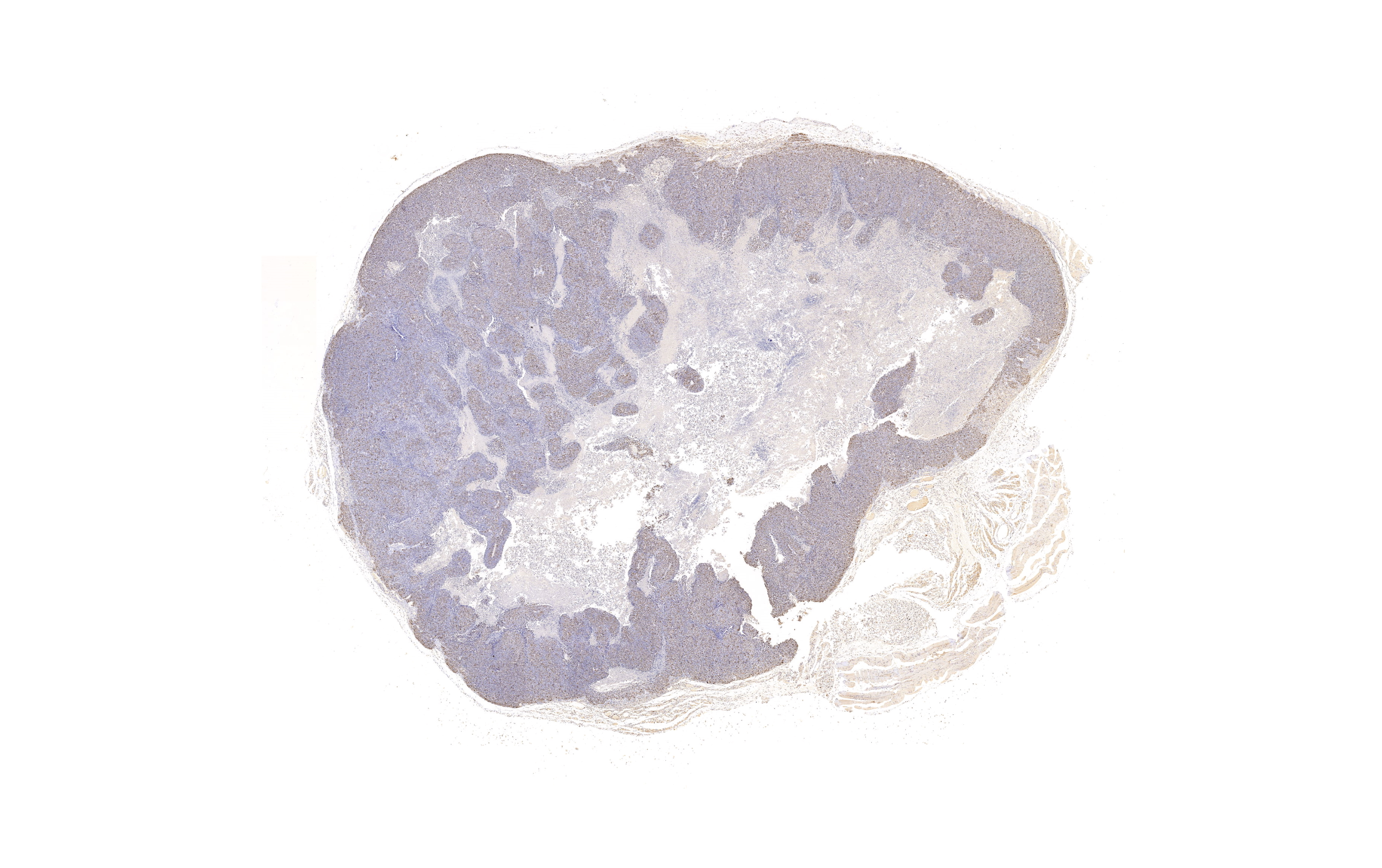

Supplement: Supplementary file 7 [file DataSheet_7.zip › fig 9c. shScrambled-4_2.0x ki67.jpg]

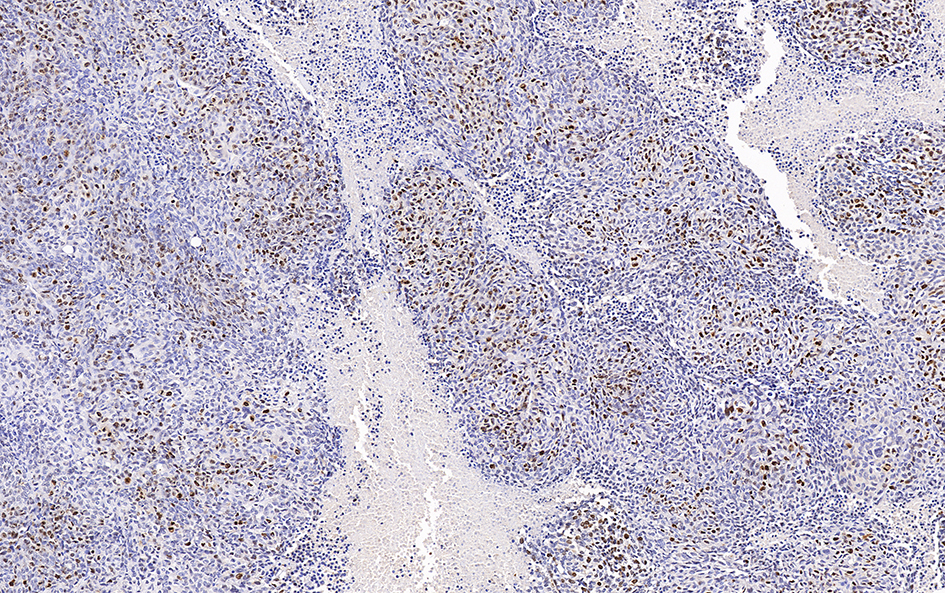

Supplement: Supplementary file 7 [file DataSheet_7.zip › fig 9c. shScrambled-4_20x ki67.jpg]

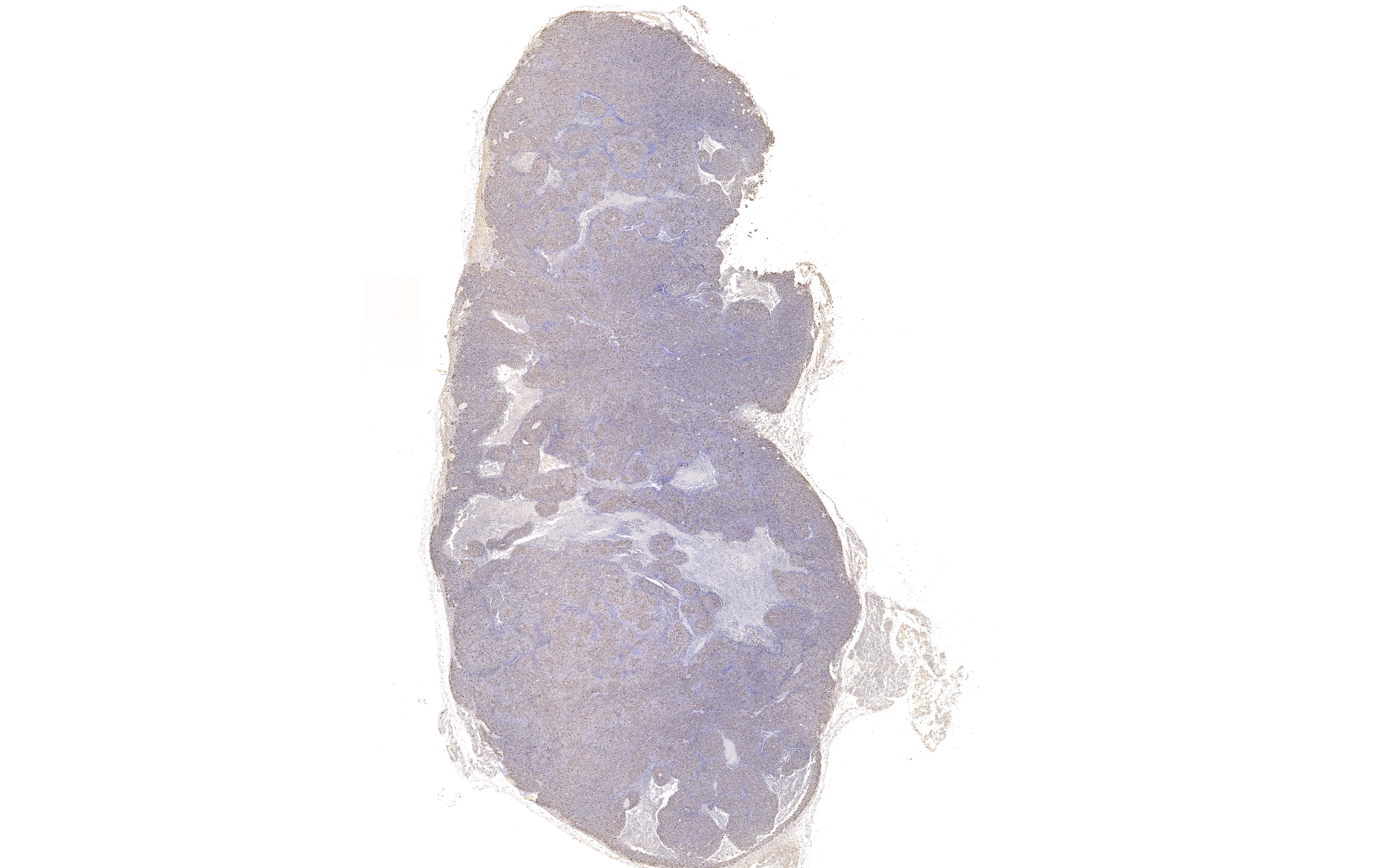

Supplement: Supplementary file 7 [file DataSheet_7.zip › fig 9c. shScrambled-5_2.0x ki67.jpg]

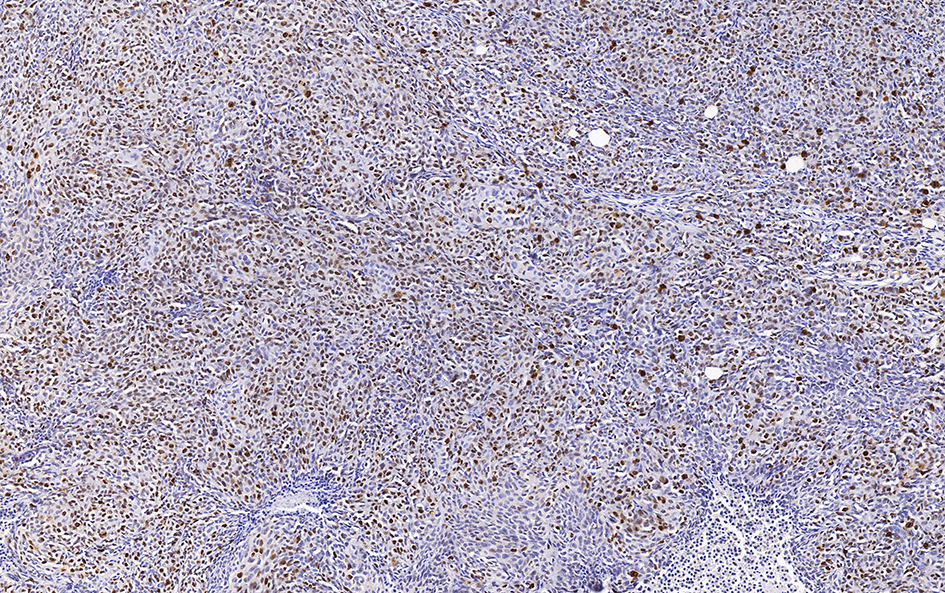

Supplement: Supplementary file 7 [file DataSheet_7.zip › fig 9c. shScrambled-5_20x ki67.jpg]

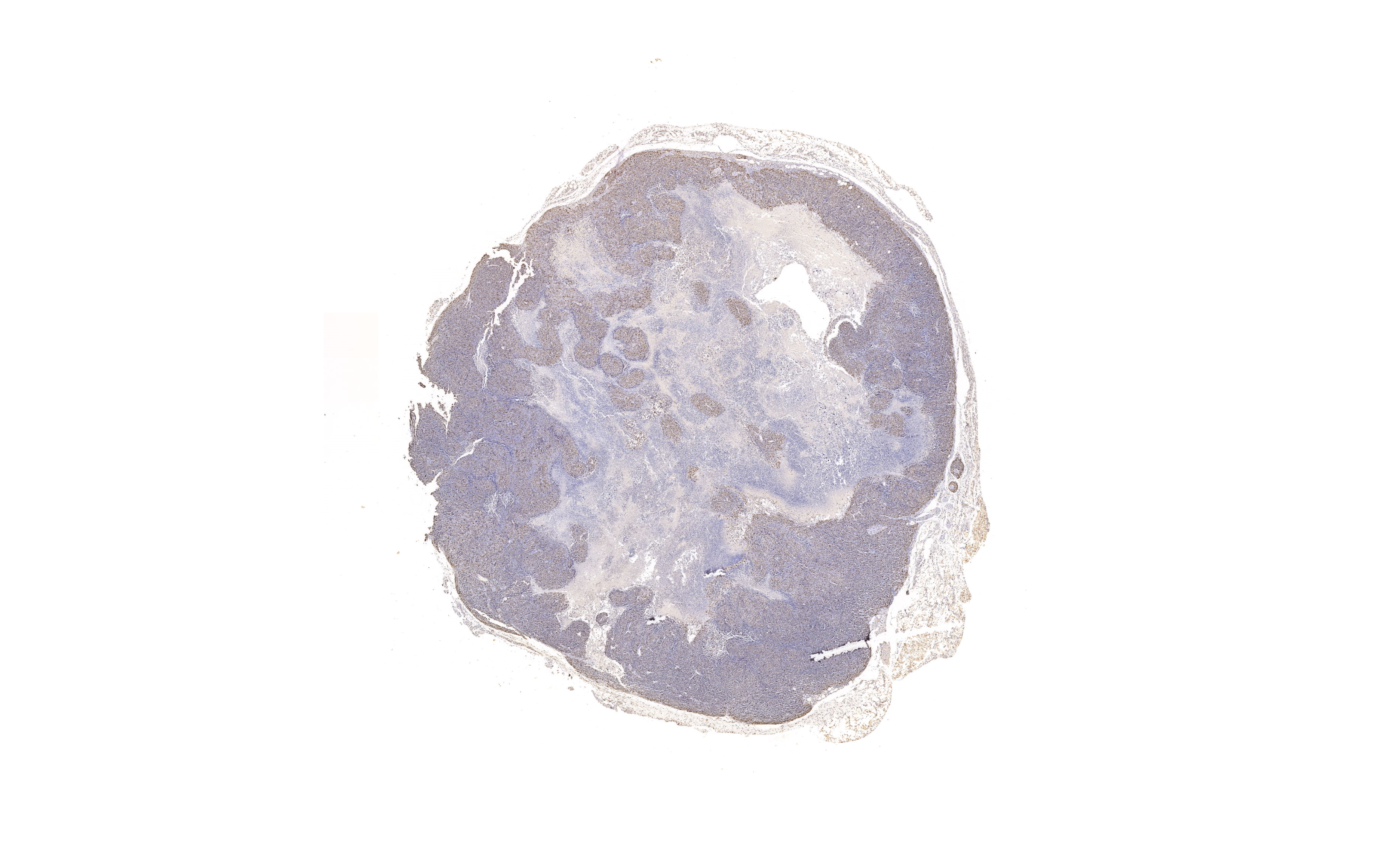

Supplement: Supplementary file 7 [file DataSheet_7.zip › fig 9c. shScrambled-6_2.0x ki67.jpg]

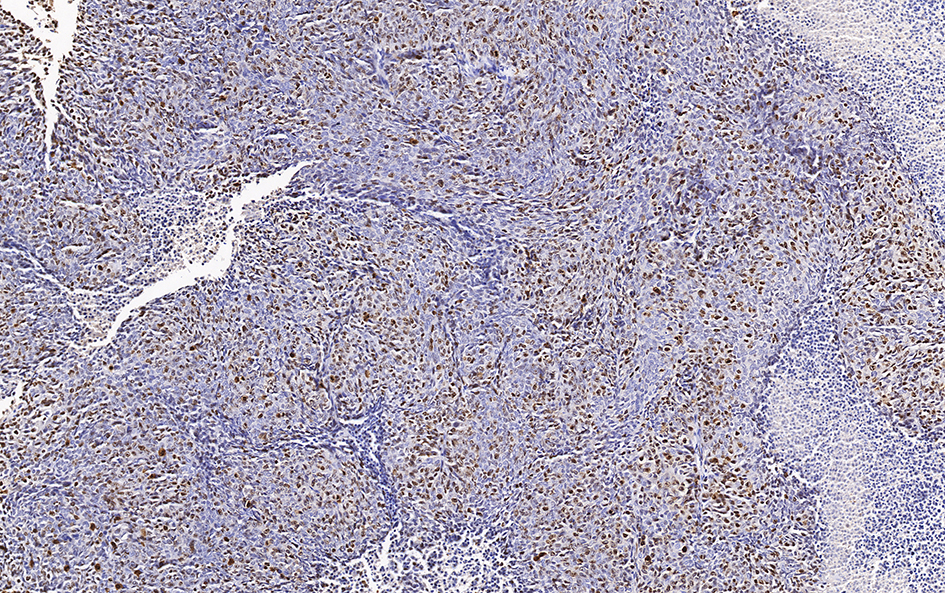

Supplement: Supplementary file 7 [file DataSheet_7.zip › fig 9c. shScrambled-6_20x ki67.jpg]
